# Comparative phylogenetic analyses of recombinant noroviruses based on different protein-encoding regions show the recombination-associated evolution pattern

**DOI:** 10.1038/s41598-017-01640-4

**Published:** 2017-07-10

**Authors:** Liang Xue, Qingping Wu, Ruimin Dong, Weicheng Cai, Haoming Wu, Moutong Chen, Gang Chen, Juan Wang, Jumei Zhang

**Affiliations:** 1 Guangdong Institute of Microbiology, State Key Laboratory of Applied Microbiology Southern China, Guangzhou, P. R. China; 2 Guangdong Provincial Key Laboratory of Microbial Culture Collection and Application, Guangdong Open Laboratory of Applied Microbiology, Guangzhou, P. R. China; 3 Department of Cardiology, The Third Affiliated Hospital, Sun Yat-sen University, Guangzhou, P. R. China; 4 College of Food Science, South China Agricultural University, Guangzhou, P. R. China

## Abstract

Noroviruses are the major cause of acute gastroenteritis worldwide, and recombination is recognized as the important mechanism for its continuous emergence. In this study, for the common GII.P12 and GII.3 recombinants, phylogenetic relationships based on different proteins in three ORFs were comparatively analyzed, focusing on the influence of intergenic recombination. By using newly designed primers, genomes of two GII.P12/GII.3 Guangzhou recombinants were firstly amplified. Combined with other reported sequences of GII.P12_ORF1 (n = 20), GII.3_ORF2 (n = 131), GII.3_ORF3 (n = 36), all GII.P12 and GII.3 strains could be divided into 6, 8, and 7 clusters based on different ORFs, which showed an obvious recombination-associated and temporally sequential evolution pattern (with the exception of GII.P12/GII.13 recombinants). Based on multiple alignments, 126 informative sites were identified in three ORFs (44, 54, and 28), and four proteins (p48, p22, VP1, and VP2) were found under positive selection. Furthermore, by using homology modeling, predicted epitopes were mapped on the P proteins of seven GII.3 representative strains, without one (Epi: 353–361) specific to the GII.4 VA387 strain. In summary, via the genome analyses, phylogenetic relationships of GII.P12 and GII.3 recombinants based on the different proteins presented a special temporally sequential evolution process associated with their recombinant types.

## Introduction

Noroviruses (NoVs) are the most common causative agent of non-bacterial acute gastroenteritis in all age groups over the world. It also causes great economic burden on society due to medical expenses^1^. A great many cases of sporadic NoVs infection and outbreaks have been reported every year. Especially in developing countries, over 200000 deaths of children below five years old were caused by this pathogen^2^. NoV-based acute gastroenteritis has become an important public health concern, but there is still a lack of effective treatments or vaccines available for its infections^3^.

NoVs are classified as a highly diverse genus, a member of the *Caliciviridae* family^4^. The virus has a positive sense, single-stranded RNA genome with a size of 7.5–7.7 kb. The human NoV genome is divided into three open reading frames (ORFs). ORF1 encodes a polyprotein that could be cleaved into six non-structural proteins, namely the N-terminal p48 protein (NS1-2), the nucleoside triphosphatase NTPase (NS3), the 3A-like p22 protein (NS4), the viral genome-linked VPg protein (NS5), the 3C-like protease 3CLpro (NS6), and the RNA-dependent RNA polymerase RdRp (NS7), respectively. ORF2 encodes a major structural protein VP1, which consists of two domains, namely a shell (S) domain and a protruding (P) arm, connected by a flexible hinge. The P arm could be further divided into two subregions of P1 and P2, and the latter one possesses most motifs that are responsible for the host-binding function and the viral antigenicity^5^. ORF3 encodes a minor structural protein VP2, and it may contribute to the expression and stability of the viral capsid^6, 7^. Different viral proteins do not show consistent homology, and four proteins, including p48, p22, VP1 (P2), and VP2, exhibit the highest degree of sequence variability in the genome^8^.

NoV has a great genetic diversity. It could be classified into six genogroups (GI to GVI) based on the full-length sequences of capsid protein VP1, and a tentative GVII has been proposed recently^9, 10^. GI, GII and GIV could infect human. Each genogroup can be subdivided into different genotypes, including at least 9 genotypes in GI and 23 genotypes in GII. GII.4 is the most studied one compared with others, since it is the most prevalent human NoV variant detected and most frequently associated with epidemics (>80%) worldwide during the last two decades^11, 12^. Every two or three years, new GII.4 epidemic variants would appear and cause new infection peaks globally. Some other NoV genotypes could also be classified into different variants during their constant evolutionary process. For example, GII.6 and GII.17 variants could be divided into three GII.6 clusters (GII.6-a to GII.6-c) and four GII.17 clusters (GII.17-a to GII.17-d), respectively^13–15^. The continuous evolution of the non-epidemic NoV variants is also an interesting issue for their potential pandemics^16^.

Antigenic drift and recombination are regarded as the main mechanisms for NoV evolution, and it could cause NoVs immune evasion and the long-term persistence in human populations^5, 17^. In addition to accumulating point mutations associated with error-prone RNA replication, NoVs undergo frequent recombination and generate variants with different genotypes in ORF1 and ORF2. The templates of the RNA polymerase may be switched during the co-infection with different NoV strains, which could generate new viral genomes with both parental sequences^17, 18^. With the exchange of ORF1 and ORF2/ORF3 genes between different NoV lineages, recombination contributes to the genetic diversity and the emergence of new epidemic variants. Kinds of recombinant NoV variants were often detected during the epidemiological surveillance in most countries, including ORF1-based GII.P12 and ORF2-based GII.3, which are prone to recombination^19, 20^. Therefore, it is still essential to monitor the prevalence and emergence of different recombinant variants for public health.

NoV recombination variants were also often detected in most cities in China. Recently, a GII.P12/GII.3 recombinant was once identified as a predominant genotype that compared to the GII.4 epidemic variants in Chongqing, Western China^21^. The same recombinant was also detected in South China in two consecutive cold seasons (2013–2014)^22^. The rising circulation of the NoV recombinant variants illustrated their significance in the viral evolution and epidemic in China. Therefore, it is necessary to collect more comprehensive information on the persistence and characteristics of recombinant variants. In this study, focusing on the characteristics of intergenic recombination, genomes of two NoV recombinant strains in South China were sequenced and comprehensively analyzed with other emerged GII.P12 and GII.3 strains based on the different protein-encoding regions.

## Results

### Genome sequences of Guangzhou NoV recombinant strains

**Figure 1:**
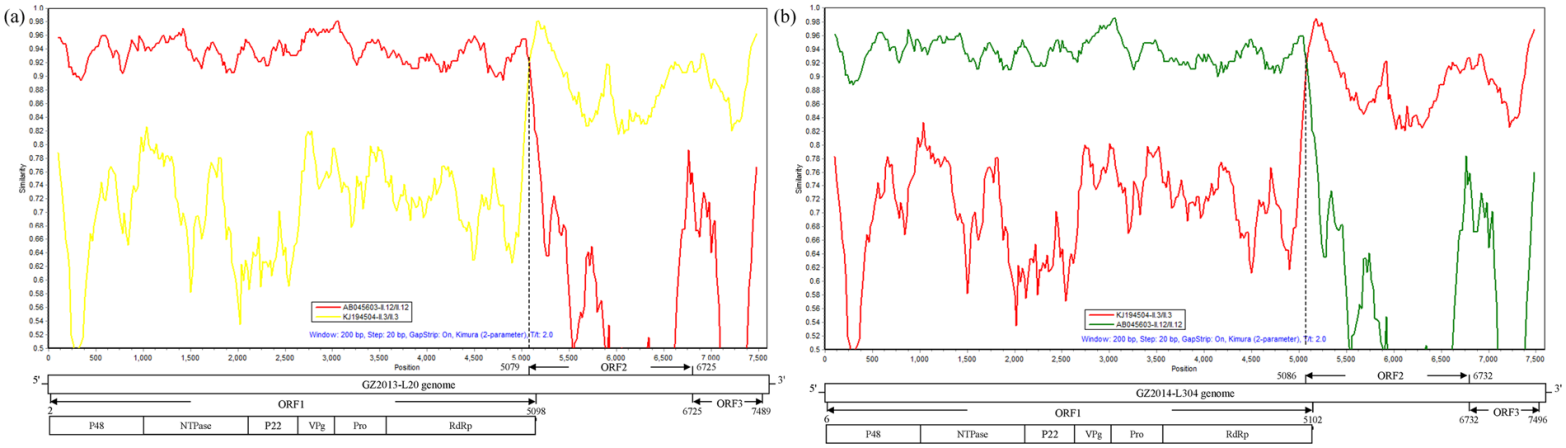
Recombination analyses of NoV Guangzhou strains using Simplot software: (**a**) GZ2013-L20 and (**b**) GZ2014-L304. The assay was performed using standard parameters of the program with a window size of 200 bp, a step size of 20 bp and with the Kimura (2-parameter) model. The y-axis indicates the nucleotide sequence similarity between the recombinant sequence and reference strains. The x-axis indicates the nucleotide position. The site where the two NoV parental strains of different genotypes have equal identity to the recombinant is the predicted site of recombination, which is marked with a black dotted line.

Using the previous “4 + 1 + 1” PCR strategy with newly designed primer pairs (Supplementary Table S1), the genomes of two Guangzhou NoV strains (GZ2013-L20 and GZ2014-L304) were sequenced and assembled (Accession numbers are KY348697-KY348698), which showed a high nucleotide similarity of 99.3%. The detailed composition of different protein-encoding regions in the new genomes was shown in Fig. 1. BLAST results of new genomes identified three sequences with high similarity in GenBank, including GU980585|CBNU1/2006/KOR, GU991355|SH312/2009/CHN and KF306213|Jingzhou/2013402/CHN (query cover = 100%, identity percent >94%).

To verify their recombination and identify the location of the putative recombination breakpoint, SimPlot and RDP analyses were performed with the reference strains GII.12/Gifu-96/JP (AB045603) and GII.3/Amsterdam-1994/NL (KJ194504). Two Guangzhou strains were both confirmed to be GII.P12/GII.3 recombinants with predicted recombination points corresponding to nt position 5068 for GZ2013-L20 and 5075 for GZ2014-L304, slightly upstream of the ORF1/ORF2 overlap region (Fig. 1). In addition, the genotypes of two Guangzhou strains were also confirmed by using the NoV online genotyping tool.

### Comparative analyses of Guangzhou strains with reference strains of the same genotype based on different viral proteins

Complete GII.P12 ORF1 sequences (n = 18), GII.3 ORF2 sequences (n = 129), and GII.3 ORF3 sequences (n = 34) were extracted from GenBank database. The positions of six non-structural protein-encoding regions in ORF1 and subdomains in ORF2 were verified by comparison with representative sequences (GenBank AB045603 for GII.P12 ORF1 and KJ194504 for GII.3 ORF2). An attempt was made to clarify the RdRp genotype for all collected ORF2-based GII.3 strains, but there were still 27/129 strains with no related information.

Based on comparison with above reference sequences, a total of 57 and 2 nucleotide substitutions were introduced in the ORF1 and ORF3 regions by the Guangzhou stains, which resulted in 13 and 2 amino acid changes, respectively. Besides, a deletion mutation was also found in the p22 region of GZ2013-L20 (Supplementary Table S2). However, no nucleotide substitutions or amino acid changes were found in ORF2 of Guangzhou strains.

To understand the variability differences of viral proteins, pairwise identities of six non-structural proteins (p48, NTPase, p22, VPg, 3CL, and RdRp) in GII.P12 ORF1, different domains in GII.3 ORF2, and GII.3 ORF3 were calculated, respectively. Different viral proteins or domains do not show consistent homology. In ORF1, the percentages of variable amino acid sites in six non-structural proteins (p48, NTPase, p22, VPg, 3CL, and RdRp) in ORF1 were identified as 4.55%, 1.91%, 10.61%, 2.26%, 2.21% and 1.18%, respectively; p22 was the most variable protein at the nucleotide (92.44%) and amino acid (94.47%) levels. In ORF2, the P domain was more variable than the S domain, and the P2 subdomain exhibited the highest degree of sequence variability (88.88% at nucleotide level, 91.06% at amino acid level); 59 variable amino acid sites were identified in the capsid protein VP1, and most (40/59) were located in P2 subdomain. No deletion/insertion mutations occurred during the evolution of GII.3 NoVs. Most variable sites were introduced by the Sweden strains (n = 31), which were detected in patients with chronic infection. For ORF3, 36 variable amino acid sites were found by multiple alignments, and the pairwise identity of the nucleotide sequences was almost equal to that of the amino acid sequences (92.35% at the nucleotide level versus 92.78% at the amino acid level), which meant a high ratio of the non-synonymous rate to the synonymous rate (Supplementary Table S3).

### Phylogenetic analyses of recombinant strains based on different protein-encoding regions and subregions

Phylogenetic analyses based on the nucleotide sequences of six non-structural proteins (p48, NTPase, p22, VPg, 3CL, RdRp) in ORF1, VP1 and its subregions (S, P, and P2), and VP2 were conducted. One GII.1 NoV strain GII.1|HCU07611|Hawaii virus was used as the root of the phylogenetic tree, and the most appropriate models were selected using the general time-reversible (GTR) substitution models by jmodeltest 2.

**Figure 2:**
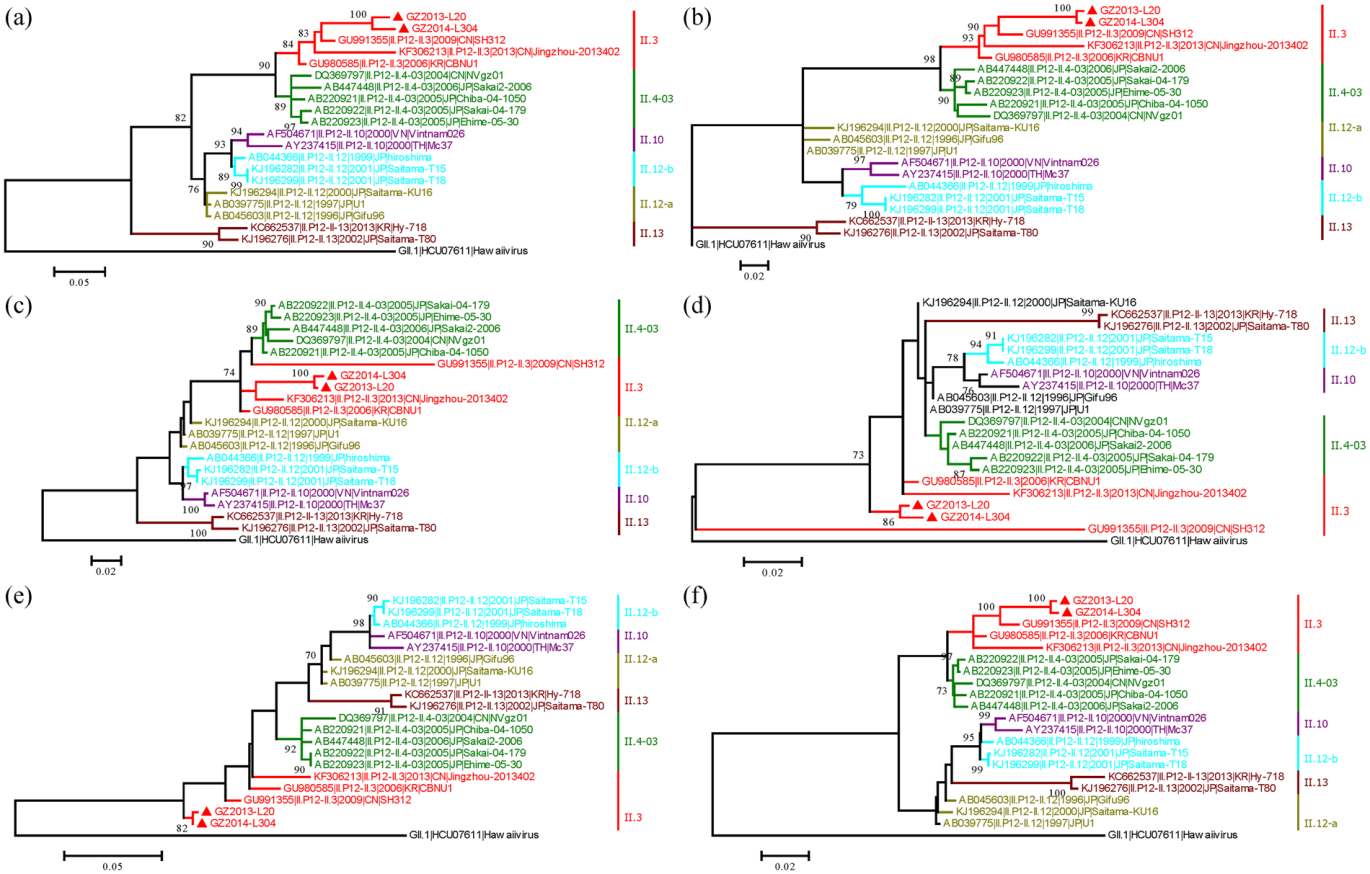
Phylogenetic trees of ORF1-GII.P12 recombinants based on nucleotide sequences of different protein encoding regions in ORF1: (**a**) p48, (**b**) NTPase, (**c**) p22, (**d**) VPg, (**e**) 3C-Pro, and (**f**) RdRp. Maximum likelihood trees were constructed using PhyML 3.0 with the general time-reversible (GTR) substitution models by jmodeltest 2 program. Numbers at the nodes indicate supporting bootstrap values (%) for 1000 resampled datasets, and only values greater than 70% are shown. The scale bar represents the unit for the expected number of substitutions per site. Guangzhou NoV strains are designated by location, year, and identification number (indicated by the black diamond). Reference sequences are indicated by their accession numbers, genotypes, years, countries, and strain names. The reference strain GII.1|HCU07611|Hawaii virus was selected as the root of phylogenetic trees.

In ORF1, based on the nucleotide sequences of six non-structural proteins, all ORF1-based GII.P12 strains (n = 20) strains could be almost subdivided into five clusters, which were associated with their recombination types and switches between ORF2-genotypes, including GII.4-2003, GII.3, GII.10, GII.12 and GII.13 (Fig. 2). However, due to the different homologies of six non-structural proteins, the topologies of six phylogenetic trees were not completely consistent, especially for the conserved VPg protein. With the exception of two strains of the ORF2 GII.13 cluster, the remaining 16 strains of other four clusters exhibited clearly epochal characteristics that were clustered in a temporally sequential pattern. Six GII.P12/GII.12 strains could be divided into subclusters, and two GII.P12/GII.10 strains showed high homology with the ORF2_GII.12 subcluster, which was consisted of AB044366|1999|JP|hiroshima, KJ196282|2001|JP|Saitama/T15, and KJ196299|2001|JP|Saitama/T18.

**Figure 3:**
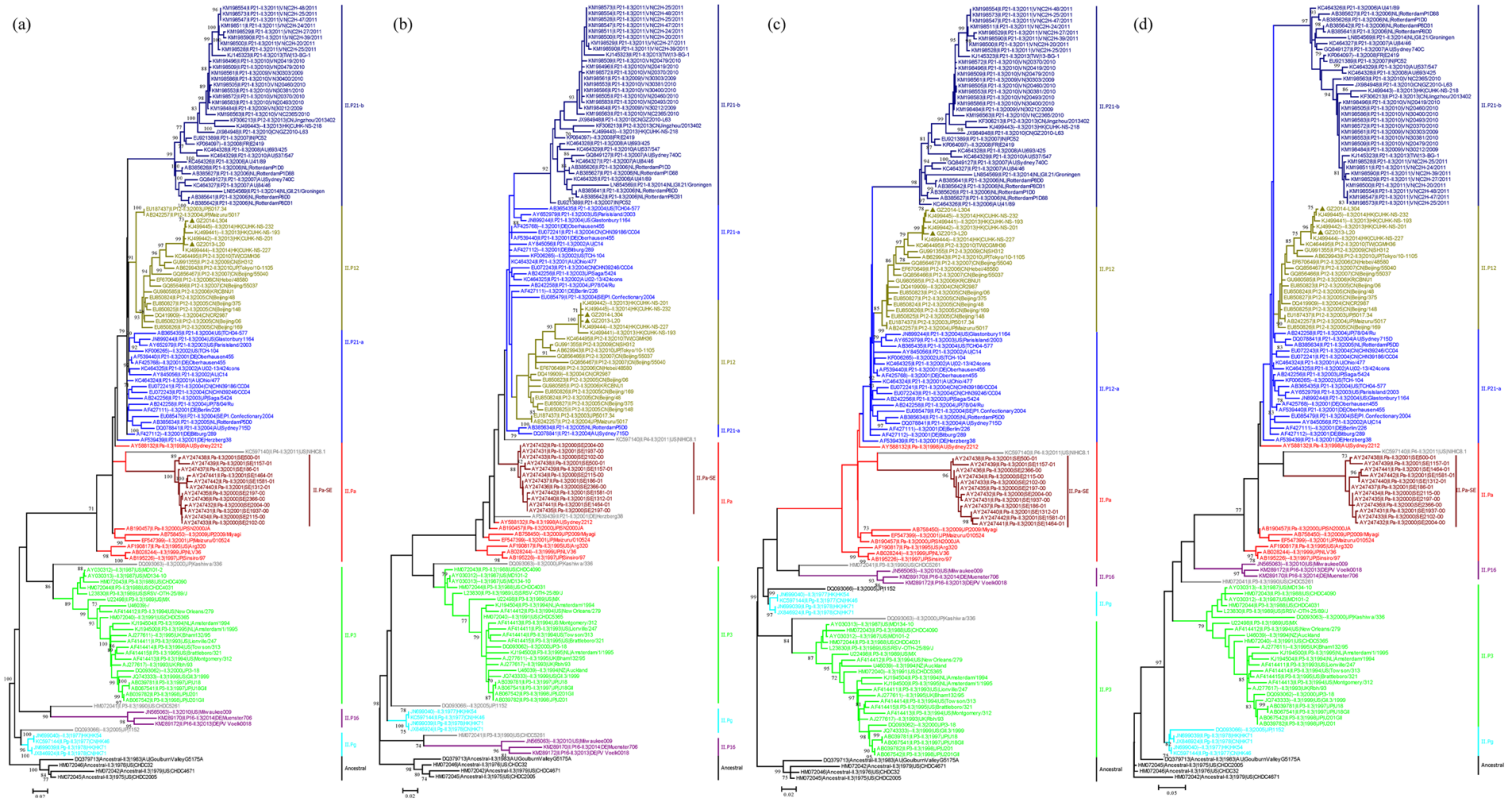
Phylogenetic trees of ORF2-GII.3 recombinants based on nucleotide sequences of the VP1 encoding region and its subregions: (**a**) VP1, (**b**) S domain, (**c**) P domain, and (**d**) P2 subdomain. Maximum likelihood trees were constructed using PhyML 3.0 with the general time-reversible (GTR) substitution models by jmodeltest 2 program. Numbers at the nodes indicate supporting bootstrap values (%) for 1000 resampled datasets, and only values greater than 70% are shown. The scale bar represents the unit for the expected number of substitutions per site. Guangzhou NoV strains are designated by location, year, and identification number (indicated by the black diamond). Reference sequences are indicated by their accession numbers, genotypes, years, countries, and strain names.

In ORF2, based on the nucleotide sequences of the VP1 and its subregions S, P, and P2, all ORF2 GII.3 strains could be subdivided into eight clusters, which were almost corresponded to their ORF1-genotypes GII.P12, GII.P21-a, GII.P21-b, GII.P16, GII.P3, GII.Pg, GII.Pa (including Sweden strains) and Ancestral (Fig. 3). All clusters also emerged in a temporally sequential pattern, with the exception of GII.P16/GII.3 strains. The results are similar to those defined in previous studies only for GII.3 VP1 sequences. In addition, there also existed four strains (DQ093063|–II.3|2000|JP|Kashiwa/336, DQ093066|–II.3|2005|JP|1152, HM072041|II.P3-II.3|1990|US|CHDC5261, and KC597140|II.P4-II.3|2011|US|NIHIC8.1) which showed high genetic distances.

**Figure 4:**
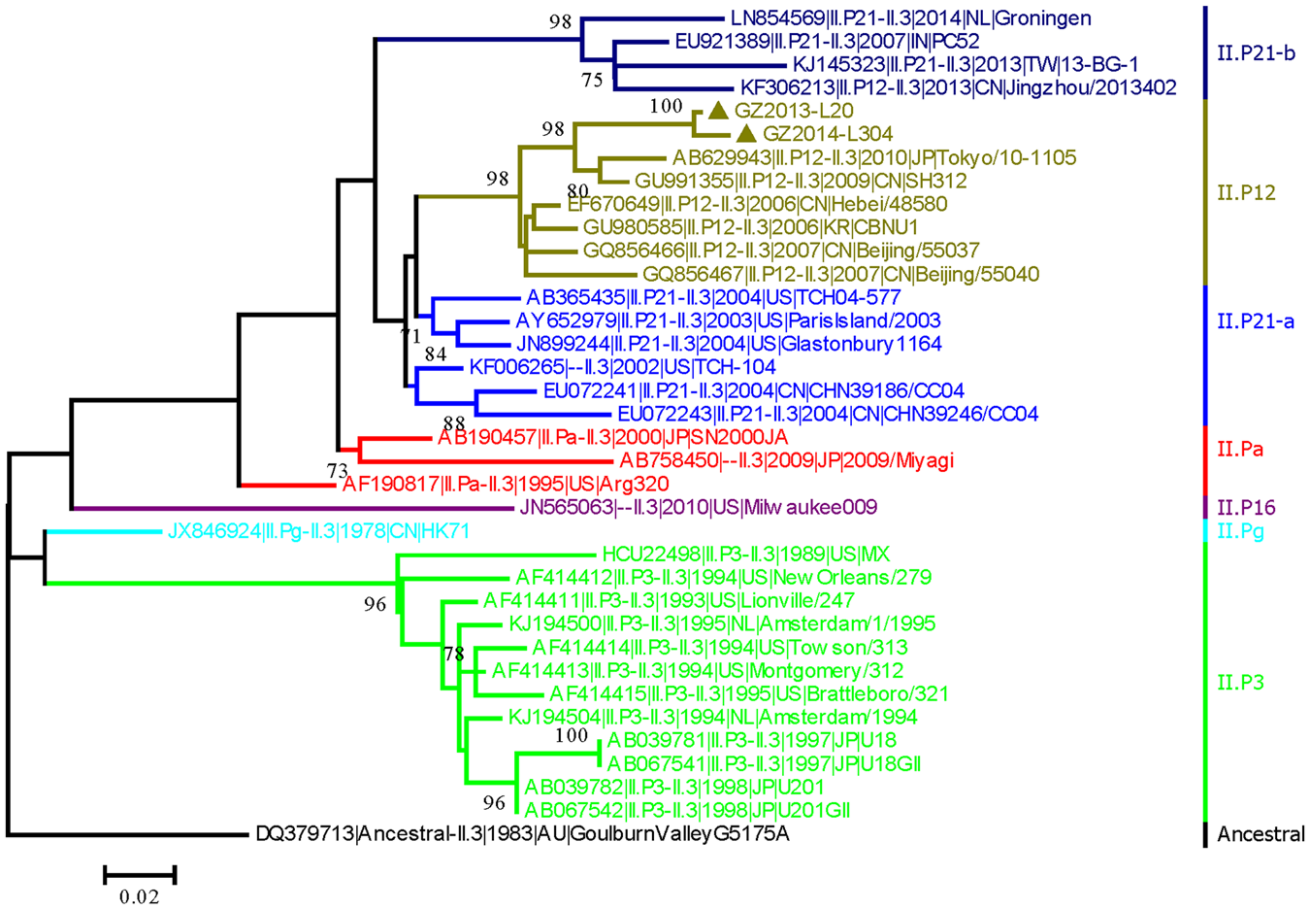
Phylogenetic tree of ORF2-GII.3 recombinants based on nucleotide sequences of VP2 encoding region. The maximum likelihood tree was constructed using PhyML 3.0 with the general time-reversible (GTR) substitution model by jmodeltest 2 program. Numbers at the nodes indicate supporting bootstrap values (%) for 1000 resampled datasets, and only values greater than 70% are shown. The scale bar represents the unit for the expected number of substitutions per site. Guangzhou NoV strains are designated by location, year, and identification number (indicated by the black diamond). Reference sequences are indicated by their accession numbers, genotypes, years, countries, and strain names.

In ORF3, the phylogenetic tree of all ORF2 GII.3 strains with complete GII.3 ORF2 sequences (n = 34) based on the nucleotide sequences of the VP2 is similar to that based on the VP1, and all strains could be subdivided into five clusters (not including ORF1_II.Pg, ORF1_II.P16, and ORF1_Ancestral, which has only one strain). And these clusters were also almost corresponded to their ORF1 genotypes GII.P12, GII.P21-a, GII.P21-b, GII.P3, and GII.Pa, with exception of the strain KF306213|II.P12-II.3|2013|CN|Jingzhou/2013402 (Fig. 4).

### Identification of informative amino acid sites and positive selection analyses

**Figure 5:**
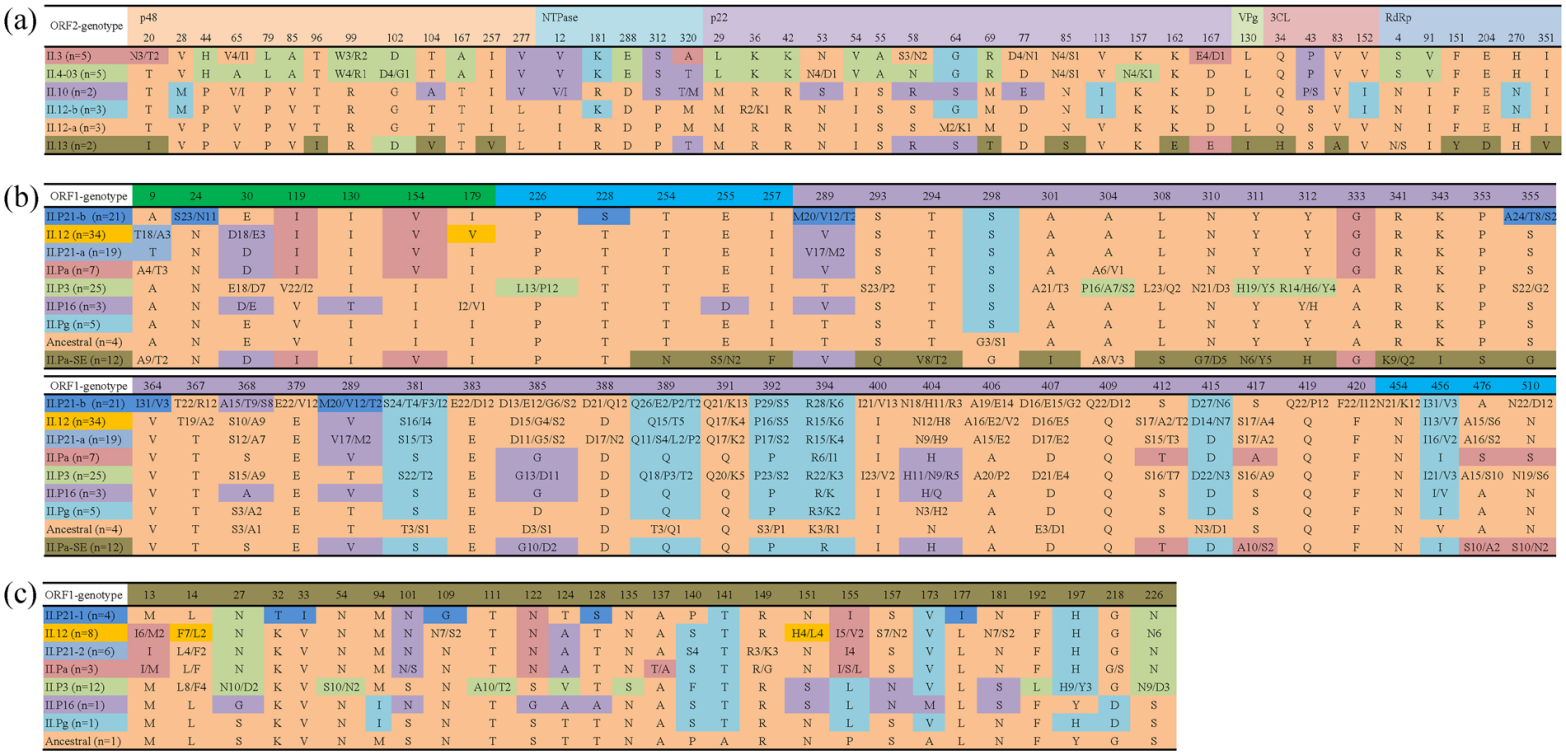
Informative sites of viral proteins among different clusters of ORF1-GII.P12 and ORF2-GII.3 recombinants based on multiple alignments. (**a**) For GII.P12 ORF1, different proteins (p48, NTPase, p22, VPg, 3C-Pro, and RdRp) are presented in light orange, light aqua green, light purple, light green, light pink, and light blue. Amino acids in ORF2-II.12-a cluster are colored in orange, and new amino acids used in other clusters (II.12-b, II.10, II.4-03, II.3, and II.13) were highlighted in aqua green, purple, green, pink, and tawny, respectively. (**b**) For GII.3 ORF2, the N- and C-terminal flanking regions of heterogeneity are in green for the S domain and cyan for the P1 subregion above the amino acids, and sites in P2 subregion are highlighted in light purple. Amino acids in ORF1-ancestral cluster are colored in orange, and new amino acids used in other clusters (II.Pg, II.P16, II.P3, II.Pa, II.P21-a, II.P12, II.P21-b, and II.Pa-SE) are highlighted in aqua green, purple, green, pink, light blue, orange, dark blue, and tawny, respectively. (**c**) For GII.3 ORF3, amino acids in ORF1-ancestral cluster are colored in orange, and new amino acids used in other clusters (II.Pg, II.P16, II.P3, II.Pa, II.P21-a, II.P12, and II.P21-b) are highlighted in aqua green, purple, green, pink, light blue, orange, and dark blue, respectively.

To understand the genetic diversity and key mutations during the evolutionary process of different GII.P12 and GII.3 recombinant variants, multiple amino acid sequence alignments of six non-structural proteins and two capsid proteins (VP1 and VP2) were performed. For six GII.P12 ORF1clusters, a total of 44 concrete differences were identified. P48 and p22 were two regions that had the most informative sites (13 and 15); rather, only one informative site was located in the VPg-encoding region (Fig. 5a). For GII.3 capsid protein VP1, a total of 54 informative sites were found, and most were located in the variable P2 subdomain (n = 38) (Fig. 5b). For GII.3 minor capsid protein VP2, there was also 28 informative sites identified (Fig. 5c).

Positive selection analyses were also performed for six non-structural proteins and two capsid proteins (VP1 and VP2) using three different methods SLAC, FEL and REL (Table 1). In ORF1, only p48 and p22 were the proteins which have the sites under positive selection; in ORF2, four residues (385, 406, 404, and 389) on viral capsid of GII.3 NoVs were identified under the positive selection, which were all located in the P2 domain; in ORF3, three residues (140, 226, and 27) were also identified. All above amino acid sites predicted to be under positive selection were also informative sites during the substitution of different recombinant clusters.

**Table 1:** Positive selection analyses of different viral proteins based on SLAC, FEL, and REL.

Protein	SLAC Method (p < 0.25)	FEL Method (p < 0.15)	REL (Bayes factor value > 50)
p48	**65**, **104** ^a^	**65**, **104**	99, **65**, **104**, 52, 102, 11, 20, 277
NTPase	—	312	—
p22	**58**	**58**, 64, 156	**58**, 156
VPg	—	—	129, 131
3CL	—	—	—
RdRp	—	4	—
VP1	**406**, **385**, **389**, **404**, 304, 297, 547, 510, 6, 9, 392, 372	**385**, **406**, **404**, **389**, 510, 297, 547, 6, 304, 349, 372, 24	**406**, **385**, **404**, **389**
VP2	**140**, **226**, **27**, 232, 148	**140**, **226**, **27**, 76, 57	**140**, **27**, **226**

^a^Positions in bold font style indicate the positive selection sites verified by three methods.

### Homology modeling and structure alignment of GII.3 capsid P proteins

**Figure 6:**
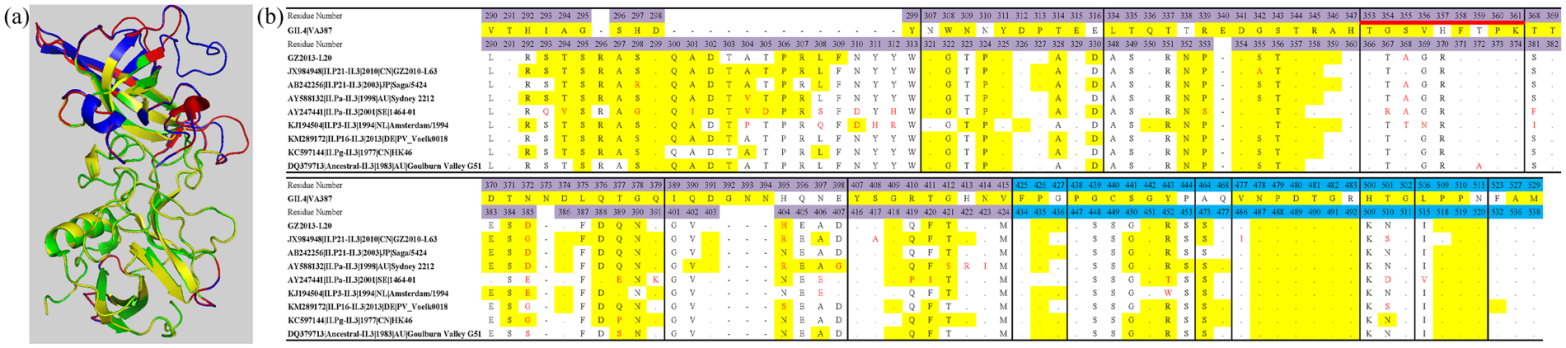
Homology modeling and prediction of potential spatial epitopes of the GII.3 capsid P domain. (**a**) Homology modeling of the P domain of GZ2013-L20. The structural superposition of the predicted structure for GZ2013-L20 P domain (purple) and the crystal structure of GII.4 NoV strain VA387 (green) was performed, which are displayed in cartoon mode. The regions with the major structural differences are highlighted in blue and red for GZ2013-L20 and VA387, respectively. (**b**) Sequence differences between the P domains of representative GII.3 strains and the GII.4 VA387 strain. A black dot indicates the same usage of amino acid as that of the GII.4 strain, and a dash indicates a deletion-mutant. The potential spatial epitopes are highlighted in yellow, and the positions with different amino acids among GII.3 subclusters are colored in red. The predicted epitope region (Epi: 353–361) that is specific to the GII.4 genotype is highlighted with a bold red line below. The sites above the amino acids of the viral capsid protein are highlighted as in Fig. 5b.

Representative capsid protein sequences of nine GII.3 strains (including one Sweden strain) were then selected and compared the sequential and structural differences with the epidemic GII.4 strain VA387. Two insertions (Q300 to Y312, R296 of GZ2013-L10) and three deletions (G392 to N394, E340, N373 of VA387) were found, which all occurred in the P2 subdomain. By using homology modeling, the structural differences of GII.3 strains and VA387 mainly embodied in 5 motifs, which were caused by the former insertions and deletions. The insertion resulted in one expanding loop in the P2 subdomain, whereas the deletion between 392 and 394 truncated the original loop of VA387 (Fig. 6a). The predicted structures were also submitted to SEPPA to calculate possible spatial epitopes (Fig. 6b). In general, no obvious differences in potential conformational epitope regions between the different GII.3 variants and GII.4 NoV were observed, positions of potential conformational epitope regions on sequences are similar or adjacent to each other for GII.4 and different GII.3 strains, but one predicted epitope region (Epi: 353–361) was also found specific to the GII.4 genotype^14, 15^.

## Discussion

NoVs are recognized as the common cause of outbreaks and sporadic cases of nonbacterial acute gastroenteritis worldwide, and recombinant strains are often detected during their epidemic surveillances. However, few studies on NoV recombination based on the full-length viral genome were conducted, and only a few completed recombinant NoV genome sequences are available all over the world. During our ongoing local NoV epidemic surveillance in Guangzhou, China, the recombinant GII.P12/GII.3 was detected in two consecutive winter seasons. In this study, the genomes of new Guangzhou strains were sequenced, and the evolutionary processes of two common recombinants ORF1_GII.P12 and ORF2_GII.3 were both performed based on different viral protein-encoding regions and subregions.

As RNA viruses, the rich genetic diversity is an important factor for their ubiquity, adaptability and uncontrollability. In addition to the fallibility of RNA polymerase, NoV recombination was also reported promoting the mutation rate of viral capsid protein and/or change the viral immunogenicity^23, 24^. Due to the lack of a suitable *in vitro* validation system, genetic information of different NoV strains is necessary to identify their genetic diversity and evolution characteristics. In our previous studies, a “4 + 1 + 1” amplification strategy was used to obtain GII.4 and GII.17 genome sequences, which could be completed in one working day^25, 26^. The next-generation sequencing was employed for assembling of NoV genome recently^8, 27^. Nevertheless, it was time consuming and high cost when compared to our method. Therefore, a set of primers was designed for amplifying six overlapped fragments of GII.P12/GII.3 recombinant genomes based on the same strategy, and RNA genomes of two Guangzhou strains were successfully sequenced. The accumulation of this recombinant genome information is necessary to understand the intact phylogenetic relationships based on different viral proteins.

The basis for epidemiological fitness of different NoV variants, as determined by the incidence and ability to cause pandemics, is partially dependent on their evolution capability. Recombination is a major driving force of viral evolution that allows a substantial exchange of genetic material. GII.P12 and GII.3 are two genotypes that are often identified as recombinant variants, including GII.P12/GII.4-2003, GII.P12/GII.10, GII.P12/GII.13, GII.P12/GII.3, GII.P1/GII.3, GII.P21/GII.3, GII.P16/GII.3, and GII.Pg/GII.3^23, 28, 29^.

Despite the overwhelming predominance of norovirus GII.4 strains in the past two decades, studies on historical stool specimens dated back to the 1970s and 1980s suggested that non-GII.4 norovirus such as GII.3 was commonly detected. In previous studies, evolution of the GII.3 capsid gene from 1975 to 2010 was associated with at least five different RdRp genotypes, and different GII.3 variants were the major causes of pediatric disease worldwide. After 2006, two lineages of GII.3 recombinant evolved simultaneously in distinct geographical locations. In this study, a continuous evolution of GII.3 capsid was observed after 2010. Most GII.3 recombinants emerged as the recombinant types of GII.P12/GII.3 and GII.P21/GII.3, the former one could remain in the original cluster, but the latter one had a serial of new amino acid substitutions, which may be clustered into a new GII.P21/GII.3 variant.

The GII.3 capsid protein VP1 sequences from the immunocompromised patient in Sweden were included in this study^30^. Because the mutations emerging during the chronic infection were the major source of the new epidemic strains^31–33^, and it was reported that novel alterations in the virus capsid are not required to infect naive pediatric hosts. Therefore, comparison between the Sweden strains and other epidemic strains could point out the informative sites for GII.3 evolution. The results showed that there existed 13 new amino acid substitutions special to the Sweden strains, 9 of which were conserved during the evolution of other GII.3 clusters. Besides, the usage of amino acid on the other sites was also different from that of other GII.3 clusters but could be employed in some strains (Y311, H312, and G355). The verification of these amino acid substitutions in the mutants could improve the understanding of its evolution requirements.

Throughout the evolution of the GII.P12 ORF1, at least four recombination events led to the acquisition of ORF2 genes of differing genotypes (Capsid protein switching). Thus, the GII.P12 ORF1 has evolved under the influence of several different capsid proteins. All GII.P12 recombinant strains appeared to be restricted to a specific geographic region, including Japan, China, and South Korea, where it was a major cause of pediatric norovirus disease in 2003 in Asia. A series of different sites within the GII.P12 ORF1 exhibited conserved amino acid substitutions which defined individual lineages and/or different capsid groups (Fig. 5a). The fixation of these substitutions within a lineage indicates an evolutionary advantage in variation at these sites. The conserved amino acids constituted a mutational profile for each lineage, defining mutations that allowed divergence into a new lineage. This suggests that the conserved substitutions within each lineage have been important evolutionary changes that led to fitter versions of previous viruses. In addition, variable amino acid sites could also be identified by their different clusters, but no sites showed continuous variation. It has been reported that RdRp switching may have been a mechanism that allowed increased rates of mutation through acquisition of an RdRp with lower fidelity and/or increased replicative ability. This can lead to the generation of large reservoirs of mutants that enable the virus to adapt to changing environments, improving viral fitness under certain selective pressures. However, the function of capsid protein switching for the virus is still unclear, which needs further investigations.

For most viruses, positively selected sites were mainly located in the exposed regions of viral capsid presumably as a result of selective pressure from neutralizing antibodies. Four sites under positive selection (sites 304, 385, 389, and 406) had been firstly identified in GII.3 capsid protein sequences spanning the years 1975 to 2006, thus they are likely to be major sites of selection at the population level^23^, and another one site 297 was also found when adding new sequences before 2010^24^, which were all located in the P2 domain. A similar result was obtained in this study. Four sites of 406, 385, 389, 404 were identified by three methods, and another seven sites of 304, 297, 547, 510, 6, 9, 392, 372 were also identified by SLAC (P < 0.25). However, these sites did not belong to the informative sites during GII.3 capsid evolution. The relationships of the location of sites under positive selection between GII.3 and other NoVs indicated that the evolution patterns of these genotypes might be different and should be investigated in further studies.

VP2 is one of the variable proteins that could promote the expression and stability of virus particles, and co-evolution of VP1 and VP2 of GII.4 variants was also proved previously^34^. All variable sites of VP2 were also in the predicted interaction regions. It could be known that GII.3 VP2 was consistent with their VP1 genotypes, and all VP2 could be subdivided into different clusters based on their recombinant types. However, the effect of VP2 variation on the epidemic or survival is still unclear. If suitable variation of three different ORFs is the driving force for the emergence of new NoV epidemic strains, the roles of different NoV proteins during viral evolution need to be further explored. Moreover, the recombination could also occur at the junction of ORF2 and ORF3, which would be a complement of the NoV recombination mechanism.

In this study, the evolutionary process of the GII.P12/GII.3 recombinants was analyzed using phylogenetics, with genome sequences of two Guangzhou strains, and an obvious temporally sequential evolution process was found which was associated with their recombinant types. Moreover, no obvious difference was observed between the capsid proteins of different GII.3 clusters, but the lack of a GII.4-specific epitope was identified when compared with the GII.4 VA387 strain. The findings of this study could be utilized for further genetic and evolutionary studies of NoV recombinant variants, which might be also important for effective vaccine design.

## Methods

### Clinical samples

During a continuous NoV surveillance study in Guangzhou, China, clinical specimens were collected and stored at −80 °C for further analyses. Viral RNA was extracted from 10% (w/v) fecal suspension using a QIAamp Viral RNA Mini Kit and then detected by one-step reverse transcription polymerase chain reaction (RT-PCR)^22^. Two NoV strains GZ2013-L10 and GZ2014-L304 were identified as the GII.P12/GII.3 inter-genotype recombinants based on the sequences of regions A and C. The study was approved by the Research Ethics Committee at the Third Affiliated Hospital of Sun Yat-sen University and the Institutional Review Board (IRB) at the Chinese CDC for the protection of human subjects (project approval number 2013(2)-76). Informed patient/guardian consent was obtained from all patients or their parents prior to sample collection. All experiments were performed in accordance with relevant guidelines and regulations.

### Primer designing and genome sequencing

The previous reported “4 + 1 + 1” strategy was employed for primer designing. The full-length and nearly full-length GII.P12 ORF1 and GII.3 ORF2/ORF3 sequences were collected from GenBank. Based on multiple alignments of these reference sequences, six amplifying primer pairs and two sequencing primers for GII.P12/GII.3 NoV genomes were designed by PRIMER PREMIER 5.0 software (Premier Biosoft International, Palo Alto, CA, USA) (Supplementary Table S1). In order to rule out co-infection by two strains of different genotypes, the junction of ORF1/ORF2 and flanking RdRp and capsid regions were contained in one amplicon, which was amplified with the primer pair II.12-4 F/II-4R.

Using viral RNA extracted from the fecal supernatant, six overlapping fragments encompassing the viral genome were amplified by one-step RT-PCR and then sequenced directly (more than three times) using an automated sequencer (ABI 3730XL, Applied Biosystems, Foster City, CA, USA). Viral genomes could be constructed by connecting overlapping sections as a single sequence.

### Multiple alignments and phylogenetic analyses

Positions of different protein-encoding regions in new genomes were verified by compared with those in the representative sequences (GenBank AB045603 for GII.P12 ORF1 and KJ194504 for GII.3 ORF2/ORF3), and all protein-encoding regions of Guangzhou strains and other NoV reference strains of the same genotype were comparatively analyzed to investigate their differences using BioEdit (version 7.0.1) and DNASTAR Lasergene MegAlign program (DNASTAR Inc., WI, USA).

Multiple alignments of different protein sequences were performed using ClustalX for Windows (version 1.83) with the default parameters^35^. Model selection was performed using jmodeltest 2 for nucleotide sequences^36^. The best-fit models for phylogenetic analyses were selected based upon the Akaike information criterion (corrected) (AICc). The maximum likelihood (ML) phylogenetic trees were then conducted in PhyML version 3.0 using the appropriate substitution models^37^. The reliability of clustering results was evaluated using a bootstrap test (1000 replications). The resulting ML trees were visualized using MEGA version 6.0^38^. All nucleotide sequences were also genotyped using the NoV automated online genotyping tool (www.rivm.nl/mpf/norovirus/typingtool)^39^. Information amino acid substitutions during the viral evolutionary process were analyzed by comparison within and between different clusters of the same genotype.

### Positive selection analyses

Using the HyPhy package implemented in the Data Monkey Web Server (http://www.datamonkey.org/), signatures of site-specific positive selection are inferred if the ratio of non-synonymous substitutions per non-synonymous substitutions site (dN) over synonymous substitutions per synonymous sites (dS) among all sequences present in different proteins alignments is statistically higher than the value observed under neutrality. The best-fitting nucleotide substitution model was first determined using the automated tool available. Three codon-based maximum likelihood methods were then used for analyses, including fixed effects likelihood (FEL), single-likelihood ancestor counting (SLAC), and random effects likelihood (REL). The SLAC model is based on the reconstruction of ancestral sequences and counts the number of dS and dN changes at each codon position of the phylogeny. FEL estimates ratios of dN to dS changes for each site in an alignment. REL uses a flexible distribution and allows dS and dN to vary across sites independently. The limit of significance level for SLAC was 0.25, FEL was 0.15, and the minimum Bayes factor value was set at 50.

### Recombination analyses

For recombination analyses, full-length genome sequences of two Guangzhou strains were analyzed using the SimPlot program (version 3.5.1) to investigate the break-point position^40^. The SimPlot analysis was conducted with the reference genome sequences (GenBank AB045603 and KJ194504) by using a window size of 200 bp and a step size of 20 bp. The identification of recombinants was also verified by using the Recombination Detection Program (RDP)^41^.

### Homology modeling

Homology modeling of the viral capsid was performed by generating a Protein Data Bank (PDB) file from the amino acid sequence by using the Swiss Model server of the Swiss Institute of Bioinformatics^42^. Modeling was performed using the published crystal structure of GII.4 NoV strain VA387 (PDB: 2OBT) as a template. Protein structures were visualized and manipulated using PyMOL v1.4.1 (DeLano Scientific, LLC, San Francisco, CA). Possible spatial epitopes were then predicted by submitting modeled 3D structures of capsid proteins to the protein antigen spatial epitope prediction web server (SEPPA) with the default threshold^43^.

### Reference sequences

A total of 18 GII.P12 ORF1 sequences (from 1996 to 2014), 129 GII.3 ORF2 sequences (from 1975 to 2014), and 34 GII.3 ORF3 sequences (from 1978 to 2014) available in the GenBank database were collected for analyzed in this study. All collected sequences comprised the complete ORF sequences (5100 bp for ORF1, 1647bp for ORF2, and 783 bp for ORF3) and their collection information (sampling dates and sites), which were provided in Table S4–S6. An attempt was made to establish the RdRp genotype for all collected strains used in the GII.3 VP1 and GII.3 VP2 evolutionary analyses. For the strains obtained from GenBank, the RdRp genotype was confirmed using the norovirus genotyping tool as above. A literature search was also conducted to determine the assigned RdRp genotype for strains lacking published RdRp sequence data.

## Electronic supplementary material

Supplementary Information

Supplementary Information

## References

[1] Bartsch SM, Lopman BA, Ozawa S, Hall AJ, Lee BY (2016). Global Economic Burden of Norovirus Gastroenteritis. PLoS One.

[2] Ahmed SM (2014). Global prevalence of norovirus in cases of gastroenteritis: a systematic review and meta-analysis. Lancet Infect. Dis..

[3] Riddle MS, Walker RI (2016). Status of vaccine research and development for norovirus. Vaccine.

[4] Cremon, C., De Giorgio, R. & Barbara, G. Norovirus gastroenteritis. *N. Engl. J. Med*. **362**, 557–557, doi:0.1007/s11894-006-0026-4 (2010). 10.1056/NEJMc0911723 20147725

[5] Debbink K, Lindesmith LC, Donaldson EF, Baric RS (2012). Norovirus immunity and the great escape. PLoS Pathog..

[6] Vongpunsawad S, Prasad BVV, Estes MK (2013). Norwalk virus minor capsid protein VP2 associates within the VP1 shell domain. J. Virol..

[7] Bertolotti-Ciarlet A, Crawford SE, Hutson AM, Estes MK (2003). The 3’ end of Norwalk virus mRNA contains determinants that regulate the expression and stability of the viral capsid protein VP1: a novel function for the VP2 protein. J. Virol..

[8] Cotten M (2014). Deep sequencing of norovirus genomes defines evolutionary patterns in an urban tropical setting. J. Virol..

[9] Vinje J (2015). Advances in laboratory methods for detection and typing of norovirus. J. Clin. Microbiol..

[10] Zheng DP (2006). Norovirus classification and proposed strain nomenclature. Virology.

[11] Siebenga JJ (2009). Norovirus illness is a global problem: emergence and spread of norovirus GII.4 variants, 2001-2007. J. Infect. Dis..

[12] Tran TNH, Trainor E, Nakagomi T, Cunliffe NA, Nakagomi O (2013). Molecular epidemiology of noroviruses associated with acute sporadic gastroenteritis in children: Global distribution of genogroups, genotypes and GII.4 variants. J. Clin. Virol..

[13] Chan-It W (2012). Emergence of a new norovirus GII.6 variant in Japan, 2008-2009. J. Med. Virol..

[14] Xue L (2015). Genome characterization of a GII.6 norovirus strain identified in China. Infect. Genet. Evol..

[15] Xue L, Wu QP, Cai WC, Zhang JM, Guo WP (2016). Molecular characterization of new emerging GII. 17 norovirus strains from South China. Infect. Genet. Evol..

[16] de Graaf M (2015). Emergence of a novel GII.17 norovirus - End of the GII.4 era?. Eurosurveillance.

[17] Bull RA, Tanaka MM, White PA (2007). Norovirus recombination. J. Gen. Virol..

[18] White PA (2014). Evolution of norovirus. Clin. Microbiol. Infect..

[19] Phumpholsup T (2015). Human norovirus genogroup II recombinants in Thailand, 2009-2014. Arch. Virol..

[20] Xue L (2013). Genetic analysis of noroviruses associated with sporadic gastroenteritis during winter in Guangzhou, China. Foodborne Pathog. Dis..

[21] Lu QB (2015). An increasing prevalence of recombinant GII norovirus in pediatric patients with diarrhea during 2010-2013 in China. Infect. Genet. Evol..

[22] Xue L (2016). Molecular epidemiology of noroviruses associated with sporadic gastroenteritis in Guangzhou, China, 2013-2015. Arch. Virol..

[23] Boon D (2011). Comparative evolution of GII.3 and GII.4 norovirus over a 31-year period. J. Virol..

[24] Mahar JE, Bok K, Green KY, Kirkwood CD (2013). The importance of intergenic recombination in norovirus GII. 3 evolution. J. Virol..

[25] Xue L, Cai WC, Wu QP, Zhang JM, Guo WP (2016). Direct sequencing and analysis of the genomes of newly emerging GII.17 norovirus strains in South China. J. Appl. Microbiol..

[26] Xue L (2016). Development of a sensitive method for directly sequencing GII.4 norovirus genome. Diagn. Microbiol. Infect. Dis..

[27] Kundu S (2013). Next-generation whole genome sequencing identifies the direction of norovirus transmission in linked patients. Clin. Infect. Dis..

[28] Fumian TM, de Andrade JDR, Leite JPG, Miagostovich MP (2016). Norovirus Recombinant Strains Isolated from Gastroenteritis Outbreaks in Southern Brazil, 2004-2011. PLoS One.

[29] Medici MC (2014). Novel recombinant GII.P16_GII.13 and GII.P16_GII.3 norovirus strains in Italy. Virus Res..

[30] Nilsson M (2003). Evolution of human calicivirus RNA *in vivo*: Accumulation of mutations in the protruding P2 domain of the capsid leads to structural changes and possibly a new phenotype. J. Virol..

[31] Debbink K (2014). Within-Host Evolution Results in Antigenically Distinct GII.4 Noroviruses. J. Virol..

[32] Hoffmann D (2012). Norovirus GII.4 and GII.7 capsid sequences undergo positive selection in chronically infected patients. Infect. Genet. Evol..

[33] Karst SM, Baric RS (2015). What Is the Reservoir of Emergent Human Norovirus Strains?. J. Virol..

[34] Chan MCW (2012). Covariation of major and minor Viral capsid proteins in norovirus genogroup II genotype 4 strains. J. Virol..

[35] Thompson JD, Gibson TJ, Plewniak F, Jeanmougin F, Higgins DG (1997). The CLUSTAL_X windows interface: flexible strategies for multiple sequence alignment aided by quality analysis tools. Nucleic Acids Res.

[36] Darriba D, Taboada GL, Doallo R, Posada D (2012). jModelTest 2: more models, new heuristics and parallel computing. Nat. Meth.

[37] Guindon S (2010). New algorithms and methods to estimate maximum-likelihood phylogenies: assessing the performance of PhyML 3.0. Syst. Biol..

[38] Tamura K, Stecher G, Peterson D, Filipski A, Kumar S (2013). MEGA6: molecular evolutionary genetics analysis version 6.0. Mol. Biol. Evol..

[39] Kroneman A (2011). An automated genotyping tool for enteroviruses and noroviruses. J. Clin. Virol..

[40] Lole KS (1999). Full-length human immunodeficiency virus type 1 genomes from subtype C-infected seroconverters in India, with evidence of intersubtype recombination. J. Virol..

[41] Martin DP (2010). RDP3: a flexible and fast computer program for analyzing recombination. Bioinformatics.

[42] Arnold K, Bordoli L, Kopp J, Schwede T (2006). The SWISS-MODEL workspace: a web-based environment for protein structure homology modelling. Bioinformatics.

[43] Sun J (2009). SEPPA: a computational server for spatial epitope prediction of protein antigens. Nucleic Acids Res.

