# Supplementary material for: Comparative phylogenetic analyses of recombinant noroviruses based on different protein-encoding regions show the recombination-associated evolution pattern
Source: Sci Rep. 2017 Jul 10;7:4976. doi: 10.1038/s41598-017-01640-4 (PMC5504017; doi:10.1038/s41598-017-01640-4)
Supplement: Supplementary file 1 — Supplementary Information [file 41598_2017_1640_MOESM1_ESM.docx]

>AB045603/GII.12/Gifu-96/JP

GTGAATGAAGATGGCGTCTAACGACGCTTCCGCTGCCGCTGCTGCTAACAGCAACAACGACACCGCAAAATCTTCAAGTGACGGAGTGCTTTCTAGCATGGCTGTCACTTTTAAACGAGCCCTCGGGGCACGGCCTAAACAGCCTCCCCCGAGGGAAATACCACAAAGGCCCCCACGACCACCCACCCCAGAACTGGTCAAAAAGATCCCTCCTCCTCCACCCAACGGGGAGGATGAACCAGTGGTTTCTTACAGCGTCAAAGATGGCGTTTCCGGCTTGCCTGAGCTTACCACTGTCAGGCAGCCGGGTGAAACCAACACGGCGTTCAGTGTTCCCCCACTCAACCAAAGGGAGAATAGGGACGCCAAGGAGCCACTAACTGGAACAATCCTGGAAATGTGGGACGGGGAGATCTACCATTACGGCCTGTATGTGGAACGAGGTCTTGTACTTGGTGTGCACAAACCACCGGCTGCCATCAGCCTCGCCAAGGTTGAACTAACACCACTCTCTCTGTTTTGGAGACCAGTGTATACACCACAGTATCTCATCTCTCCAGACACTCTCAGGAGACTGCACGGAGAGTCGTTCCCCTACACAGCCTTTGACAACAACTGCTATGCCTTCTGTTGTTGGGTCCTGGACCTAAACGACTCGTGGTTGTGCAGGAGAATGATCCAGAGGACAACTGGTTTCTTCAGGCCTTACCAAGACTGGAATAGGAAACCCCTTCCCACCATGGATGACTCCAAGTTGAAGAAGGTAGCTAACATATTCTTGTGTGCGCTATCTTCGCTATTCACTAGGCCCATCAAAGACATAATAGGAAAGTTGAGGCCTCTTAACATCCTTAACATCTTGGCCTCATGTGATTGGACTTTTGCAGGCATAGTGGAATCTTTGATCCTCCTGGCAGAGCTCTTTGGAGTTTTCTGGACACCCCCAGATGTGTCTGCGATGATCGCCCCTTTACTAGGTGACTACGAGCTGCAGGGGCCCGAGGACCTTGCAGTGGAACTCGTTCCAATAGTGATGGGGGGGATTGGTTTGGTGCTAGGATTCACTAAAGAGAAGATCGGGAAAATGTTGTCATCTGCCGCATCCACCTTAAGAGCTTGTAAAGACCTTGGTGCATACGGACTGGAAATCTTAAAATTGGTCATGAAGTGGTTCTTCCCAAAGAAAGAGGAAGCAAATGAGCTGGCTATGGTGAGGTCCATCGAGGATGCGGTGCTGGACCTCGAGGCAATTGAGAACAATCACATGACTGCCCTCCTCAAAGACAAAGACAGCCTGGCAACCTATATGAGAACTCTTGACCTCGAGGAGGAGAAAGCCAGGAAGCTTTCAACCAAGTCTGCTTCACCTGATATCGTGGGCACAATCAACGCTCTCCTGGCGAGAATCGCCGCTGCACGTTCCCTGGTGCATCGGGCGAAAGAGGAGCTCTCCAGCAGACCAAGACCTGTTGTTGTTATGATATCAGGCAGGCCAGGGATAGGGAAAACCCACCTTGCCAGGGAATTGGCCAAGAGAATCGCAGCTTCACTCACAGGGGACCAGCGTGTGGGTCTCATCCCTCGCAATGGTGTTGATCACTGGGACGCATATAAGGGAGAAAGAGTCGTCCTATGGGACGATTATGGAATGAGTAATCCCATCCACGACGCCCTCAGGTTGCAAGAACTTGCTGACACCTGCCCCCTCACGCTAAATTGTGACAGGATTGAGAACAAGGGAAAGGTCTTTGACAGTGATGCCATAATCATCACCACTAACCTGGCCAACCCAGCACCACTGGACTATGTCAATTTTGAAGCATGCTCGAGGCGCATCGACTTCCTCGTGTATGCAGATGCCCCTGAGGTTGAGAAGGCAAAACGTGATTTCCCAGGTCAACCTGACATGTGGAAGAACGCTTTCAGTCCTGACTTCTCGCACATAAAACTGATGCTGGCTCCGCAGGGTGGCTTCGACAAGAACGGAAACACCCCACATGGGAAAGGCGTCATGAAAACCCTCACCACTGGTTCCCTCATCGCTCGAGCATCAGGGCTACTCCATGAGAGGTTAGATGAGTACGAGCTGCAGGGCCCAACCCCCACCACCTTCAACTTTGACCGCAACAAGGTGCTTGCGTTCAGACAGCTTGCTGCCGAAAACAAGTACGGGTTGATGGACACAATGAGAGTTGGAAGACAGCTCAAGGATGTCAGGACCATGCCAGAGCTCAAACAAGCACTCAGGAATATCTCAATCAAGAGTTGTCAGATAGTGTATGGTGGCTGCACCTATATGCTTGAGTCTGATGGCAAGGGTGATGTGAAAGTTGACAGAGTTCAGAACGCCACTGTACAGACCAACAATGAACTGGCCGGTGCCCTACACCATCTTAGGTGTGCCAGAATTAGATATTATGTCAAGTGTGTTCAGGAGGCCCTGTATTCCATCATCCAAATTGCTGGAGCTGCATTTGTAACCACGCGCATTGTCAAGCGCATGAACATACAAGACCTTTGGTCCAAGCCACAGGTGGAAGATACAGAGGAGACTGCTAGCAAGGATGGGTGCCCAAAACCCAAGGATGATGACGAGTTCGTTGTCTCATCCGACGACATCAAAACCGAGGGCAAGAAAGGAAAGAACAAGTCTGGCCGTGGTAAGAAGCACACAGCATTCTCAAGCAAAGGTCTCAGTGATGAAGAGTACGATGAGTACAAAAGAATCAGAGAAGAAAGAAACGGCAAATACTCCATAGAGGAATACCTTCAGGACAGAGACAAGTATTATGAGGAGGTGGCCATCGCCAGGGCGACCGAAGAGGACTTCTGTGAAGAAGAAGAGGCCAAGATCCGACAAAGGATTTTCAGGCCAACAAGGAAACAACGCAAAGAGGAGAGGGCCTCTCTCGGTTTGGTCACAGGCTCTGAAATCAGGAAGAGGAACCCAGACGACTTCAAGCCTAAAGGAAAGCTGTGGGCTGATGACGACAGGAGTGTTGACTACAATGAGAGACTCAATTTTGAAGCCCCACCAAGCATTTGGTCGAGGATAGTCAACTTTGGTTCAGGTTGGGGCTTTTGGGTTTCCCCCAGCCTGTTCATAACATCAACTCATGTCATACCCCAGGGCGCACAGGAGTTCTTTGGGGTTTCCATCAAACAAATTCAGATACACAAATCGGGTGAATTCTGTCGCTTAAGGTTTCCAAAACCAATCAGAACTGATGTGACAGGCATGATCCTAGAAGAAGGTGCGCCCGAAGGGACCGTGGTCACACTACTCATCAAGAGGCCAACTGGAGAACTCATGCCCTTGGCAGCCAGAATGGGAACCCATGCAACCATGAAGATTCAAGGGCGCACTGTTGGGGGTCAAATGGGCATGCTCCTAACAGGATCCAACGCCAAGAGTATGGATCTGGGCACCACACCAGGTGACTGTGGCTGTCCCTACATTTACAAGAGGGGGAATGACTACGTAGTCATTGGAGTCCACACGGCTGCTGCCCGTGGAGGGAACACTGTCATATGTGCCACCCAGGGGAGCGAGGGAGAAGCCACACTTGAAGGCGGTGACAACAAGGGAACCTACTGTGGTGCACCAATCTTAGGTCCAGGAAGTGCCCCAAAGCTCAGCACCAAGACTAAGTTTTGGAGATCATCCACAGCACCACTCCCACCTGGTACCTATGAACCAGCCTACCTTGGCGGCAAGGACCCCAGAGTCAAGGGTGGCCCTTCATTGCAACAAGTTATGAGGGACCAGCTGAAACCATTCACTGAGCCCAGGGGTAAACCACCAAAACCAAGTGTGTTAGAGGCTGCCAAGAAAACCATCATCAATGTTCTTGAACAAACAATTGATCCACCTCAAAAATGGTCATTCGCGCAGGCATGCGCATCCCTCGACAAGACCACTTCCAGTGGTCACCCGCACCACATGCGGAAAAACGACTGCTGGAACGGGGAGTCCTTTACAGGCAAATTGGCAGACCAGGCTTCCAAGGCCAACCTGATGTTCGAAGAGGGAAAGAATATGACCCCAGTCTATACAGGTGCGCTTAAGGACGAGCTGGTCAAGACTGACAAAATTTATGGCAGGATCAAAAAGAGGCTTCTCTGGGGCTCGGATCTGGCAACCATGATCCGGTGCGCTCGAGCGTTTGGGGGCCTGATGGAGGAACTCAAAGCACATTGTGTCACACTACCCGTCAGAGTAGGTATGAATATGAATGAGGATGGTCCTATCATCTTTGAGAGACACTCCAGATACAAATACCACTATGATGCTGATTACTCCCGGTGGGACTCAACACAACAAAGAGCCGTGTTAGCAGCAGCCTTAGAAATCATGGTTAAGTTCTCCCCAGAACCGCATCTGGCCCAAAAGGTTGCAGAAGACCTTCTCTCTCCCAGCGTGATGGACGTAGGTGACTTCAAAATATCAATCAATGAGGGCCTCCCCTCCGGGGTGCCCTGCACCTCCCAATGGAATTCCATCGCCCACTGGCTCCTCACTCTCTGTGCACTTTCTGAGGTTACAAACCTGTCCCCTGACATTATCCAGGCCAACTCCCTCTTTTCCTTCTACGGTGATGATGAAATTGTGAGCACAGACATAAAGTTGGACCCAGAGAAGTTGACAGCAAAACTTAAGGAATACGGGTTGAAACCGACCCGCCCTGACAAGACTGAGGGACCCCTTGTTATCTCTGAGGACCTGAATGGCCTAACCTTCCTGCGGAGGACTGTGACCCGCGACCCAGCTGGTTGGTTTGGAAAGTTGGAACAGAGTTCAATACTTAGGCAAATGTATTGGACTAGGGGCCCTAACCATGAAGACCCATCTGAAACAATGATACCACACTCCCAAAGACCCATACAATTGATGTCTTTGCTGGGCGAGGCTGCACTCCACGGCCCAGCATTCTACAGCAAAATCAGCAAGCTGGTCATTGCAGAGCTGAAGGAAGGTGGCATGGATTTTTACGTGCCCAGACAAGAGCCAATGTTCAGATGGATGAGGTTCTCAGATCTGAGCACGTGGGAGGGCGATCGCAATCTGGCTCCCAGTTTTGTGAATGAAGATGGCGTCGAGTGACGCCGCTCCATCTAATGATGGTGCAGCCGGTCTTGTACCAGAGGCTAACAATGAGACCATGGCACTTGAACCGGTGGCTGGGGCTTCAATAGCCGCCCCACTCACCGGTCAAAACAATATTATAGACCCCTGGATTAGATTAAATTTTGTGCAGGCTCCCAATGGAGAGTTCACGGTTTCACCCCGCAACTCGCCCGGGGAAGTCCTATTAAACCTGGAATTAGGCCCCGAACTAAATCCATACCTAGCACACCTTTCTAGAATGTATAATGGTTATGCAGGTGGGGTTGAGGTGCAAGTACTACTGGCTGGGAATGCGTTCACAGCTGGAAAATTGGTGTTTGCCGCAGTTCCCCCTCATTTTCCATTAGAAAACATAAGCCCTGGCCAGATAACTATGTTCCCTCATGTAATTATTGATGTTAGGACTTTAGAACCAGTTTTGTTGCCCCTTCCAGATGTTAGGAATAATTTCTTTCATTATAATCAGCAGAATGAACCGAGGATGAGACTCGTAGCAATGCTTTACACTCCTCTTAGATCTAATGGTTCTGGTGATGATGTGTTTACTGTCTCCTGCAGGGTGCTTACCCGACCTTCCCCTGATTTTGATTTTAATTACTTGGTCCCCCCTACCGTTGAATCTAAAACTAAACCCTTCACACTCCCTATCTTGACTATAGGGGAGTTAACCAACTCCAGGTTCCCTGTGCCCATAGATGAGCTCTACACCAGTCCCAATGAGAGTCTGGTGGTGCAACCCCAGAATGGGAGATGCGCGCTAGATGGGGAGCTGCAGGGCACGACTCAGCTCCTCCCCACGGCGATTTGCTCATTCAGGGGCCGGATCAATCAGAAGGTGAGTGGAGAAAACCATGTTTGGAATATGCAGGTCACCAACATCAACGGGACCCCTTTTGATCCAACAGAGGATGTCCCGGCTCCTCTAGGCACCCCAGATTTCTCTGGCAAGCTCTTTGGTGTACTAAGCCAGAGGGACCATGATAATGCCTGTAGGAGTCATGATGCAGTAATTGCAACCAACTCTGCCAAATTCACTCCAAAATTGGGCGCTATACAAATTGGCACATGGGAAGAAGACGATGTGCACATCAACCAACCTACTAAGTTTACTCCAGTTGGCTTGTTTGAAAATGAAGGTTTCAACCAGTGGACACTCCCCAATTACTCTGGAGCCTTAACACTCAATATGGGGTTGGCCCCTCCTGTGGCCCCCACCTTCCCTGGTGAACAAATCCTTTTCTTTAGATCCCACATTCCTCTTAAAGGAGGTGTGGCGGACCCAGTTATTGATTGTCTCTTGCCCCAAGAGTGGATCCAACATCTTTACCAAGAGTCGGCCCCTTCACAATCAGATGTGGCATTGATTAGGTTTACAAATCCAGACACAGGACGTGTTCTATTTGAAGCAAAATTACACAGGAGTGGTTACATTACAGTGGCCAATACTGGTAGCAGACCGATTGTGGTACCAGCTAATGGTTACTTCAGGTTTGACTCTTGGGTTAATCAATTCTATTCTCTCGCCCCCATGGGAACTGGAAATGGGCGCAGAAGGGTGCAATAATGGCTGGAGCTTTTATAGCAGGGCTTGCTGGTGACATAGTCACCAATGGCATTGGCTCACTTGTGAACGCTGGGGCTAATGCAATAAATCAAAAAGTAGACTTTGAAAATAACAAGCAACTACAGCAGGCTTCTTTCAACCATGATAAAGAGATGCTGCAAGCTCAAGTCCAGGCCACCAAACAGTTGCAGGCTGATATGATTGCAATCAGACAAGGGGTGTTGACCGCGGGCGGCTTCTCCCCCACTGATGCAGCAAGAGGGGCAGTTAATGCACCTATGACTCAGGTCTTAGACTGGAACGGGACCAGGCATTGGGCCCCCGGAGCCACGAAAACCACTGCTCTCTCCGGTGGATTCACCAATGCTTCTCATGCCAGAACTGTCGACCTGACCAAGAAGACACCAGCCACACCAGCCCCTACGTCTGTTTCTAGACCTAGCTCTGTTGCCTCTACAGTCTCCACCCGCTCAACCTTGGTTAGCGGGTCTTTCAATTCTTCTTCTTCAGCTAGGAGTTCTTCTAGTGTTTCTTCTCAACCCACCTCCTCCTCTTCTCGGACCAGTGAGTGGGTGCGCAGCCAAAACAGGGCACTGGAGCCTTACATGAGGGGAGCGCTACACACAGCCTATGTGACGCCTCCCTCTAGTAGAGCTTCTAGTAATGGCACAGTCTCAACCGTGCCAAAAGAGGTTTTGGACTCCTGGACATCTGCATTTAACACCCGCAGACAACCGCTATTCGCTCATCTCCGTCGGAGAGGGGAGTCACAAGTTTAGTGAAAAGATGATCTTTATTTTCTTTCCTTTGAAGATTCTTTTTGTCTTTT

> KJ194504/GII.3/Amsterdam-1994/NL

GTGAATGAAGATGGCGTCTAACGACGCTTCCGTTGCCGTTGCTGGCAAAAACAACAACAACGACAAGGAAAAATCTTCAGGTGACAGCTTGTTTTCTAACATGACTGTCTCCTTTAAGAAAGCCCTCGGGGCGCGGCCTAAACAACCGCCCCCGGGAGAAATAAAACAAACACAAAAACCACCAAGACCACCAACACCGGAACTGGTGAAAAGAATACCCCCACCACCACCCAATGGCGAGGATGAACCAAAGGTGGTATACAGGGTGGGAGAAGGTGTGTCCGGGCTGCCCGACTTAACAACCGTGGCGCAGCCTGACGCACAAGGCACAGCTTATAGTGTACCCCCACTTAGCCAAAGAGAGGTCGGCGAAGCTAAAGAACCGCTACCTGGATCCATTCTGGAGATGTGGGATGGTGAGATCTACCACTATGGGCTGTACGTTGATCGAGGGCACATACTTGGGGTGCATAAACCACCTGCTGCAGTAAGCCTCGCCAAAATTGAACTGACACCACTGTCTCTCTACTGGAGAGTGGTTTACACTCCCCAGTACTTGATAGCCCCAGACACTCTGAAGGGTTTGAATGGAGAGTCATTCCCATACACAGCCTTTGACAACAACTGTTACGCCTTCTGTTGTTGGGTCTTAGATCTCAATGACTCTTGGCTTAGCAGGAGAATGATTCGAAGGACAACCGGTTTCTTCAGACCGTACCAAGATTGGAATAGAAAACCCCTGCCAACCATGGATGAGCCAAAAATCAAGAAGGCCGCAAATGCTATTCTGTGCGCTCTCTCCTCACTCTTCACTAGACCCATTAAGGACATCATTGGGAAGCTCAAACCAATGAACATCCTCAATATATTGGCAACTTGTGACTGGACTTTTGCAGGCATAGTGGAGTCCCTTATTCTTCTTGCTGAACTTTTTGGGGTGTTCTGGACACCCCCAGATGTGTCTGCGATGATTGCTCCCTTACTCGGTGACTACGAGATGCAAGGCCCAGAAGACCTGGCCATGGAACTTGTACCCGTGGTAATGGGAGGGATAGGTTTGGTGTTGGGATTCACCAAGGAGAAGATCGGCAAGATGCTTTCATCAGCTGCATCCACGCTTAAGGCCTGCAAAGATCTAGGAGCCTATGGATTGGAGATACTCAAATTGGTCATGAAGTGGTTCTTCCCTAAGAAGGAAGAGGCTAACGAGTTAGCCTTGGTAAGGGCCATCGAGGATGCAGTCCTGGATTTAGAAGCAATAGAAAACAACCACATGACCACCCTCCTCAAGGACAAAGACAGTCTTGCTACATACATGAGGACTCTTGACCTAGAGGAAGAGAAAGCAAGAAAACTGTCCACCAAATCCGCATCACCTGACATTGTGGGCACAATAAATGCATTATTGGCAAGGATTGCAGCCGCTAGGTCATTAGTCCACAAGGCTAAGGAGGAGCTCTCAAGCAGACAGAGGCCTGTCGTCGTGATGATATCTGGCAGACCAGGCATAGGGAAGACCCATCTCGCCAGAGAGCTAGCCAAGAAAATTGCATCAACCCTGTCGGGTGACCAAAGGATTGGACTGGTGCCTAGAAACGGTGTAGACCACTGGGACGCCTACAAGGGGGAAAGAGTGGTACTATGGGATGACTATGGCATGAGCAACCCCATACAGGATGCACTGAGGCTTCAAGAGTTGGCTGATACCTGTCCTCTAACTCTAAACTGTGACAGAATTGAAAACAAGGGAAAAGTATTTGACAGTGATGTCATAATCTTAACAACCAACCTCGCAAACCCAGCACCGCTGGACTATGTCAACTTTGAGGCTTGCTCCAGACGCATAGACTTTTTAGTGTATGCAGATGCACCTGACATTGAGAAAGCCAAGCGTGACTTTCCCGGCCAACCAGATATGTGGAAAGATCACTACAGACCAGACTTTACACACATCAAACTTCAGCTAGCACCACAGGGAGGTTTTGATAAGAATGGCAACACCCCACATGGTAAAGGTGTAGTGAAGTCTCTGACGCTTGGGTCATTGATCGCCAGGGCTTCCGGTTTGCTTCACGAGAGAATGGATGAATTCGAGCTCCAAGGCCCTAACCTACCAACCTTCAATTTTGACCGCAACAAGATCGCCGCCTTTAGGCAATTGGCAGCTGAAAACAAATATGGTATGGTGGACACACTGAGGGTCGGTAATCAACTGAAGAGTGTTAAGACCATGGATGAGCTCAAACAGGCTATAAAGAATATCACCATTAAAAAGTGCCAAATAGTGTACAATGGGTCTACCTACACAATGGAGTCTGATGGAAGAGGCAAGGTTTCAGTTGACAAGGTGCAAAACGCAACGGTCCAAACTAACAATGAGCTTTCAGGAGCGCTGCACCACTTAAGATCTGCAAGAATAAGATATTATGTTAAATGTTTCCAGGAAGCTGTCTATTCTTTACTACAAATTGCTGGTGCGGCTTTTGTCACCTCACGCATTGTGAGGCGCATGAACATCTCGAGCCTCTGGTCGAAGCCACCTGTTGAAGAGAGTGATGAATCTGAAGACAAAGAAGGGTGCCCCAAGCCCCGAGATGAAGATGACCTCACTATCGATTCCAAGGACATTAAAGTGGAAGGAAAGAAAGGCAAGAACAAGTCCGGCCGAGGTAAGAAACACACAGCCTTCTCATCTAAGGGCCTCAGTGATGAAGAGTACGATGAGTACAAGAGAATCAGGGACGAAAGGAATGGCAAATATTCAATAGAGGAGTACCTCCAAGACAGGGACAGATACTATGAAGAGCTAGCCATTGCTAAGGCCACTGAAGAGAACTTCTGTGAAGAGGAGGAGATCAAGATTCGTCAGAGAATCTTCCGCCCCACCAAAAAGCAGCGGAAAGAGGAAAGAGCCACACTTGGGCTCGTCACAGGGTCGGAGATCAGAAAGAGGAATCCAGATGACTTCAAGCCAAAAGGGAAACTGTGGGCTGATGATAGCAGGAGTGTGGACTACAATGAGAGGATAGATTTTGAAGCGCCCCCTAGCGTTTGGTCAAGGATAGTCAACTTTGGCACAGGCTGGGGATTCTGGGTTTCTCCAAGTCTCTTCATAACCTCGACACACGTGATACCAAAAGGAATCACGGAGGCATTTGGGGTGCCCATGAACCAAATCCAAATTCATAAATCAGGGGAATTCTGCCGCTTGCGGTTCCCAAAACCAATCAGGCCAGACGTGAGTGGGATGATTTTGGAAGAGGGTGCCCCTGAAGGAACTGTTGCGTCCATCCTCATCAAAAGAACAACAGGGGAGTTGATGCCCCTTGCAGCCAGAATGGGAACCCACGCCACAATGAAAATCCAAGGAAGAACGGTGGGTGGCCAGATGGGCATGTTACTCACAGGTTCAAATGCCAAAAGCATGGACCTAGGCACAACACCAGGTGACTGTGGGTGCCCCTACGTATACAAGAGGGGCAATGACTTTGTGGTCATTGGCGTGCACACTGCAGCAGCACGTGGAGGAAACACAGTCATATGTGCCACGCAAGGAAGCGAAGGTGAAGCCACTCTTGAGGGAGGTGACGACAAGGGCACCTATTGTGGAGCCCCCATTTTAGGACCTGGTAATGCACCAAAATTGAGCACAAAAACAAAATTCTGGAGATCATCCAACGCACCGCTCCCACCAGGCACCTATGAACCAGCATACCTAGGTGGGAAAGATCCACGTGTGAAGGGCGGTCCGTCTCTGCAACAGGTCATGAGAGACCAACTTAAACCTTTCACAGAGCCCAGAGGGAAACCACCAAACCCGAGTGTCCTAGAATCAGCAAAGAAAACTATCATCAATGTTCTAGAGCAGGTCATTGACCCCCCCCAGAAGTGGTCTTATGCCCAAGCTTGTGCATCCCTTGACAAAACAACCTCCAGTGGAAACCCGCACCACGTTCGGAAAAATGATTACTGGAATGGTGAATCCTTCACAGGAAAACTTGCAGACCAGGCTTCAAAAGCAAATCTCATGTATGAAGAAGGCAAACACATGCCACCGGTTTACACCGCAGCGCTCAAGGATGAGCTGGTGAAAACTGACAAAATCTATGGCAAAATTAAGAAAAGGCTCCTGTGGGGTTCTGACCTCTCCACCATGATTCGGTGCGCCAGAGCATTTGGTGGACTCATGGATGAGCTCAAAGCAAATTGTATCACACTTCCTGTCAGAGTTGGCATGAACATGAATGAAGATGGTCCCATAATATTTGAGAAACATTCCAGATACAGATACCACTATGATGCAGATTACTCCCGCTGGGACTCCACGCAGCAGCGGGCAGTGTTGGCAGCAGCACTTGAAATCATGGTGAGGTTCTCTGCTGAACCACAGCTAGCACAAATAGTGGCTGAAGACCTGCTAGCACCAAGTGTGGTTGATGTGGGTGACTTCAAGATCACCATTAATGAAGGCCTACCTTCTGGTGTGCCTTGCACCTCACAGTGGAACTCCATTGCCCACTGGTTGCTTACTTTGTGTGCCCTTTCTGAGGTGACAGGATTAGGCCCCGACATCATACAAGCTAATTCCATGTACTCTTTCTATGGTGATGATGAGATTGTGAGCACTGACATTAAATTGGACCCAGAGAAACTGACTGCAAAACTCAAAGAGTACGGCCTCAAACCCACCCGGCCCGACAAGACCGAAGGGCCGCTGGTGATCAGTGAAGACTTGAATGGTTTAACGTTCCTCCGTCGAACAGTCACCCGTGACCCAGCAGGTTGGTTTGGAAAACTGGAGCAAAGTTCCATCCTCAGGCAGCTATACTGGACAAGGGGACCTAACCATGAAGACCCCAGTGAAACCATGATACCACATGCGCAGAGACCCGTGCAGCTCATGGCACTACTGGGAGAATCCTCCCTACATGGACCCTCATTTTACAGCAAGGTCAGCAAGCTGGTTATATCTGAACTTAAGGAGGGAGGAATGGATTTTTATGTGCCCAGACAAGAGTCAATGTTCAGGTGGATGAGGTTCTCAGATCTAAGCACATGGGAGGGCGATCGCAATCTGGCTCCCAGTTTTGTGAATGAAGATGGCGTCGAATGACGCTGCTCCATCTAATGATGGTGCCGCCGGCCTCGTCCCAGAGATCAACAATGAGGCAATGGCGCTAGAGCCAGTGGCGGGTGCAGCGATAGCAGCGCCCCTCACTGGCCAGCAAAATATAATTGATCCCTGGATTATGAATAATTTTGTGCAAGCACCTGGTGGTGAGTTTACAGTGTCACCTAGGAATTCCCCTGGTGAAGTGCTTCTCAATTTGGAATTAGGCCCAGAAATAAATCCCTATTTGGCTCATCTTGCTAGAATGTACAATGGTTATGCAGGTGGATTTGAAGTGCAAGTGGTCCTAGCTGGAAATGCGTTTACAGCAGGAAAGGTTATCTTTGCAGCTATACCCCCTAATTTCCCTATTGACAATCTGAGCGCGGCACAGATCACAATGTGTCCGCACGTGATTGTGGATGTCAGGCAGTTGGAACCAATCAATCTCCCGATGCCTGATGTCCGCAACAATTTCTTTCATTATAATCAAGGTTCTGATTCAAGATTACGTTTGGTTGCAATGCTGTATACACCTCTTAGGGCAAATAATTCTGGAGATGATGTTTTCACTGTGTCTTGTAGGGTGTTAACTAGGCCCAGCCCTGATTTCTCATTCAATTTTCTTGTCCCACCCACTGTGGAATCAAAGACAAAGCCTTTTACCCTCCCCATTTTAACCATCTCTGAAATGTCCAATTCCAGGTTTCCAGTTCCAATTGACTCTCTGCACACCAGCCCAACTGAGAATATAGTTGTCCAGTGCCAAAATGGGCGCGTCACTCTTGACGGTGAGTTAATGGGCACCACCCAACTCTTACCGAGCCAAATATGTGCTTTCAGGGGCACACTCACTAGATCAACAAGCAGGGCCAGTGACCAAGCCGACACACCAACCCCCAGGCAATTCGACCATCGTTGGCACATACAATTGGATAATCTAAATGGAACTCCCTACGACCCTGCAGAGGACATACCAGCTCCTTTGGGCACACCAGACTTCCGGGGTAAGGTCTTTGGCGTGGCCAGCCAGAGAAACCCCGACGGCACAACAAGGGCACATGAAGCAAAAGTGGACACAACAACTAACCGCTTCACCCCAAAATTGGGCTCCTTAGAAATAATCACTGAATCTGAAGACTTTGACACAAACCAGTCAACAAAATTCACCCCAGTTGGCGTCGGAGTTGACAATGAGGAAGAATTCCAACAATGGTCCTTACCCAACTATTCTGGTCAGTTTACTCATAATATGAACTTAGCCCCAGCTGTCGCCCCCAATTTTCCTGGTGAACAGCTACTTTTCTTCCGGTCACAGCTGCCATCCTCTGGTGGGTGGTCTAACGGGATTCTAGACTGCCTGGTCCCCCAGGAATGGGTTCAACACTTCTACCAGGAATCAGCCCCCGCCCAAACGCAGGTGGCCCTGGTTAGGTATGTCAACCCTGACACTGGCAGAGTGCTATTTGAGGCCAAGCTACACAAATTGGGTTTTATGACTATAGCAAAGAATGGTGACTCCCCAATAACTGTCCCTCCAAATGGATACTTTAGATTTGAATCTTGGGTTAACCCCTTTTATACACTTGCCCCCATGGGAACTGGAAACGGGCGTAGAAGGATTCAATAATGGCTGGAGCTTTTGTAGCAGGATTGGCTGGTGACATGCTCACAAACACTGTAGGGTCTTTGGTTAATGCAGGGGCCAATGCTATTAATCAAAAAGTTGATTTTGAAAATAATAAATATTTGCAAAATGCTTCTTTTAATCATGATAAGGAGATGTTAAGTGCACAAATTGAGGCAACAAAGAGGCTGCAGGCTGACATGATTGCAATCAAACAAGGGGTCTTGACCGCTGGCGGCTTTTCCCCCACTGATGCAGCACGTGGGGCAATTAATGCCCCAGTGACAAAAGTTTTAGATTGGAGTGGAACAAGGTACTGGGCACCAAACGCCGCCTCCACAACTTCAATGTCAGGTGGCTTCACAAGCCAAGTTGTGCACAGAACCACACCAAATTTCAAAACGAGCCAGGCCCCCAAATTCACACCCAGCAGTGGGTCTTCAGTGAGATCAAGCTCAACCCAACTCACCAACTTGAGCTCACACTCATCCGGGTCGTCCCGATCTAGCGGGTCTACGATTGTCAGCTCGCTGCCGTCCTCCAGTAGGACTAGGGACTGGGTCAACCAACAAAATCTCAATTTGGAACCACACATGCCTGGATCTCTCAGGACAGCTTTTGTCACTCCACCATCTAGCACAGCCTCTAGTTCAGGCACAGTCTCAACCGTGCCCAAAAATGTTTTGGACTCCTGGACATCTGCGTTTAACACGCGCAGACAGCCGCTGTTTGCACACCTTCGTAGAAGGGGGGAGTCAAATGTTTAGTGAAAAGATTATTCTTAAATTTGATTTTGAA
