# Supplementary material for: Comparative phylogenetic analyses of recombinant noroviruses based on different protein-encoding regions show the recombination-associated evolution pattern
Source: Sci Rep. 2017 Jul 10;7:4976. doi: 10.1038/s41598-017-01640-4 (PMC5504017; doi:10.1038/s41598-017-01640-4)
Supplement: Supplementary file 2 — Supplementary Information [file 41598_2017_1640_MOESM2_ESM.docx]

Contents:

- 18 GII.P12 ORF1 sequences (Table S4):
- 129 GII.3 ORF2 sequences (Table S5)
- 34 GII.3 ORF3 sequences (Table S6)

# 18 GII.P12 ORF1 sequences (Table S4):

>AB039775|II.12/II.12|1997|JP|U1

ATGAAGATGGCGTCTAACGACGCTTCCGCTGCCGCTGCTGCTAACAGCAACAACGACACCGCAAAATCTTCAAGTGACGGAGTGCTTTCTAGCATGGCTGTCACTTTTAAACGAGCCCTCGGGGCACGGCCTAAACAGCCTCCCCCGAGGGAAATACCACAAAGGCCCCCACGACCCCCCACCCCAGAACTGGTCAAAAAGATCCCTCCTCCTCCACCCAACGGGGAGGATGAACCAGTGGTTTCTTACAGCGTCAAAGATGGCGTTTCCGGCTTGCCTGAGCTTACCACTGTCAGGCAGCCGGGTGAAACCAACACGGCGTTCAGTGTTCCCCCACTCAACCAAAGGGAGAATAGGGACGCCAAGGAGCCACTAACTGGAACAATCCTGGAAATGTGGGACGGGGAGATCTACCATTACGGCCTGTATGTGGAACGAGGTCTTGTACTTGGTGTGCACAAACCACCGGCTGCCATCAGCCTCGCCAAGGTTGAACTAACACCACTCTCTCTGTTTTGGAGACCAGTGTATACACCACAGTATCTCATCTCTCCAGACACTCTCAGGAGACTGCACGGAGAGTCGTTCCCCTACACAGCCTTTGACAACAACTGCTATGCCTTCTGTTGTTGGGTCCTGGACCTAAACGACTCGTGGTTGTGCAGGAGAATGATCCAGAGGACAACTGGTTTCTTCAGGCCTTACCAAGACTGGAATAGGAAACCCCTTCCCACCATGGATGACTCCAAGTTGAAGAAGGTAGCTAACATATTCTTGTGTGCGCTATCTTCGCTATTCACTAGGCCCATCAAAGACATAATAGGAAAGTTGAGGCCTCTTAACATCCTTAACATCTTGGCCTCATGTGATTGGACTTTTGCAGGCATAGTGGAATCTTTGATCCTCTTGGCAGAGCTCTTTGGAGTTTTCTGGACACCCCCAGATGTGTCTGCGATGATCGCCCCTTTACTAGGTGACTACGAGCTGCAGGGGCCCGAGGACCTTGCAGTGGAACTCGTTCCAATAGTGATGGGGGGGATTGGTTTGGTGCTAGGATTCACCAAAGAGAAGATCGGGAAAATGTTGTCATCTGCTGCATCCACCTTAAGAGCTTGTAAAGACCTTGGTGCATACGGACTGGAAATCTTAAAATTGGTCATGAAGTGGTTCTTCCCAAAGAAAGAGGAAGCAAATGAGCTGGCTATGGTGAGGTCCATCGAGGATGCGGTGCTGGACCTCGAGGCAATTGAGAACAATCACATGACTGCCCTCCTCAAAGACAAAGACAGCCTGGCAACCTATATGAGAACTCTTGACCTCGAGGAGGAGAAAGCCAGGAAGCTTTCAACCAAGTCTGCTTCACCTGATATCGTGGGCACAATCAACGCTCTCCTGGCGAGAATCGCCGCTGCACGTTCCCTGGTGCATCGGGCGAAAGAGGAGCTCTCCAGCAGACCAAGACCTGTTGTTGTTATGATATCAGGCAGGCCAGGGATAGGGAAAACCCACCTTGCCAGGGAATTGGCCAAGAGAATCGCAGCTTCTCTCACAGGGGACCAGCGTGTGGGTCTCATCCCTCGCAATGGTGTTGATCACTGGGACGCATATAAGGGAGAAAGAGTCGTCCTATGGGACGATTATGGAATGAGTAATCCCATCCACGACGCCCTCAGGTTACAAGAACTTGCTGACACCTGCCCCCTCACGCTAAATTGTGACAGGATTGAGAACAAAGGAAAGGTCTTTGACAGTGATGCCATAATCATCACCACTAACCTGGCCAACCCAGCACCACTGGACTATGTCAATTTTGAAGCATGCTCGAGGCGCATCGACTTCCTCGTGTATGCAGATGCCCCTGAGGTCGAGAAGGCAAAGCGTGATTTCCCAGGTCAACCTGACATGTGGAAGAACGCTTTCAGTCCTGACTTCTCGCACATAAAACTGATGCTGGCTCCGCAGGGTGGCTTCGACAAGAACGGAAACACCCCACATGGGAAAGGCGTCATGAAAACCCTCACCACTGGTTCCCTCATCGCTCGAGCATCAGGGCTACTCCATGAGAGGTTAGATGAGTACGAGCTGCAG>GGCCCAACCCCCACCACCTTCAACTTTGACCGCAACAAGGTGCTTGCGTTCAGACAGCTTGCTGCCGAAAACAAGTACGGGTTGATGGACACAATGAGAGTTGGAAGACAGCTCAAGGATGTCAGGACCATGCCAGAGCTCAAACAAGCACTCAAGAATATCTCAATCAAGAGTTGTCAGATAGTGTATGGTGGCTGCACCTATATGCTTGAGTCTGATGGCAAGGGTGATGTGAAAGTTGACAGAGTTCAGAACGCCACTGTACAGACCAACAATGAACTTGCCGGTGCCCTACACCATCTTAGGTGTGCCAGAATTAGATACTATGTCAAGTGTGTTCAGGAGGCCCTGTATTCCATCATCCAAATTGCTGGAGCTGCATTTGTAACCACGCGCATTGTCAAGCGCATGAACATACAAGACCTTTGGTCCAAGCCACAGGTGGAAGATACAGAGGAGACTGCTAGCAAGGATGGGTGCCCAAAACCCAAGGATGATGACGAGTTCGTTGTCTCATCCGACGACATCAAAACCGAGGGCAAGAAAGGGAAGAACAAGTCTGGCCGTGGTAAGAAGCACACAGCATTCTCAAGCAAAGGTCTCAGTGATGAAGAGTACGATGAGTACAAAAGAATCAGAGAAGAAAGAAACGGCAAATACTCCATAGAGGAATACCTTCAGGACAGAGACAAGTATTATGAGGAGGTGGCCATCGCCAGGGCGACCGAAGAGGACTTCTGTGAAGAAGAAGAGGCCAAGATCCGACAAAGGATTTTCAGGCCAACAAGGAAACAACGCAAAGAGGAGAGGGCCTCTCTCGGTTTGGTCACAGGCTCTGAAATCAGGAAGAGGAACCCAGACGACTTCAAGCCTAAAGGAAAGCTGTGGGCTGATGACGACAGGAGTGTTGACTACAATGAGAGACTCAATTTTGAAGCCCCACCAAGCATCTGGTCGAGGATAGTCAACTTTGGATCAGGTTGGGGCTTTTGGGTTTCCCCCAGCCTGTTCATAACATCAACTCATGTCATACCCCAGGGCGCACAGGAGTTCTTTGGGGTTTCCATCAAACAAATTCAGATACACAAATCGGGTGAATTCTGTCGCTTGAGGTTTCCAAAACCAATCAGAACTGACGTGACAGGCATGATCCTAGAAGAAGGTGCGCCCGAAGGGACCGTGGTCACACTACTCATCAAGAGGCCAACTGGAGAACTCATGCCCTTGGCAGCCAGAATGGGAACCCATGCAACCATGAAGATTCAAGGGCGCACTGTTGGGGGTCAAATGGGCATGCTCCTAACAGGATCCAACGCCAAGAGTATGGATCTGGGCACCACACCAGGTGACTGTGGCTGTCCCTACATTTACAAGAGGGGGAATGACTACGTAGTCATTGGAGTCCACACGGCTGCTGCCCGTGGAGGAAACACTGTCATATGTGCCACCCAGGGGAGCGAGGGAGAAGCCACACTTGAAGGCGGTGACAACAAGGGAACCTACTGTGGTGCACCAATCTTAGGTCCAGGAAGCGCCCCAAAGCTCAGCACCAAGACTAAGTTCTGGAGATCATCCACAGCACCACTCCCACCTGGTACCTATGAGCCAGCCTACCTTGGCGGCAAGGACCCCAGAGTCAAGGGTGGCCCTTCATTGCAACAAGTTATGAGGGACCAGCTGAAACCATTCACCGAGCCCAGGGGTAAACCACCAAAACCAAGTGTGTTAGAGGCTGCCAAGAAAACCATCATCAATGTTCTTGAACAAACAATTGATCCACCTCAAAAATGGTCATTCGCGCAGGCATGCGCATCCCTCGACAAGACCACTTCCAGTGGTCACCCGCACCACATGCGGAAAAACGACTGCTGGAACGGGGAGTCCTTTACAGGCAAATTGGCAGACCAGGCTTCCAAGGCCAACCTGATGTTCGAAGAGGGAAAGAACATGACCCCAGTCTACACAGGTGCGCTTAAGGACGAGCTGGTCAAGACTGACAAAATTTATGGCAAGATCAAAAAGAGGCTTCTCTGGGGCTCGGATCTGGCGACCATGATCCGGTGCGCTCGAGCGTTTGGGGGCCTGATGGAGGAACTCAAAGCACATTGTGTCACACTACCCGTCAGAGTAGGTATGAATATGAATGAGGATGGTCCTATCATCTTTGAGAGACACTCCAGATACAAATACCATTATGATGCTGATTACTCCCGGTGGGACTCAACACAACAAAGAGCCGTGTTAGCAGCAGCCTTAGAAATCATGGTTAAGTTCTCCCCAGAACCGCATCTGGCCCAAAAGGTTGCAGAAGACCTTCTCTCTCCCAGCGTGATGGACGTAGGTGACTTCAAAATATCAATCAATGAGGGCCTCCCCTCCGGGGTGCCCTGCACCTCCCAATGGAATTCCATCGCCCACTGGCTCCTCACTCTCTGTGCACTTTCTGAGGTTACAAACCTGTCCCCTGACATTATCCAGGCCAACTCTCTCTTTTCCTTCTACGGTGATGATGAAATTGTGAGCACAGACATAAAGTTGGACCCAGAAAAGTTGACAGCAAAACTTAAGGAATACGGGTTGAAACCGACCCGCCCTGATAAGACTGAGGGACCCCTTGTTATCTCTGAGGACCTGAATGGCCTAACCTTCCTGCGGAGGACTGTGACCCGCGACCCAGCTGGCTGGTTTGGAAAGTTGGAACAGAGTTCAATACTCAGGCAAATGTATTGGACTAGGGGCCCTAACCATGAAGACCCATCTGAAACAATGATACCACACTCCCAAAGACCCATACAATTGATGTCTTTGCTGGGCGAGGCTGCACTCCACGGCCCAGCATTCTACAGCAAAATCAGCAAGCTGGTCATTGCAGAGCTGAAGGAAGGTGGCATGGATTTTTACGTGCCCAGACAAGAGCCAATGTTCAGATGGATGAGGTTCTCAGATCTGAGCACGTGGGAGGGCGATCGCAATCTGGCTCCCAGTTTTGTGAATGAAGATGGCGTCGAG

>AB044366|II.12/II.12|1999|JP|hiroshima

ATGAAGATGGCGTCTAACGACGCTTCCGCTGCCGCTGCTGCTAACAGCAACAACGACACCGCAAAATCTTCAAGTGACGGAATGCTTTCTAGTATGGCTGTCACATTTAAACGAGCCCTCGGGGCACGGCCTAAACAGCCTCCCCCGAGGGAAATACCACAAAGACCCCCACGACCACCCACCCCAGAACTGGTCAAAAAGATCCCTCCTCCTCCACCCAACGGGGAGGATGAACCAGTGGTTTCTTACAGCGTCAAAGATGGCGTTTCCGGCTTGCCTGAGCTTACCACTGTCAGGCAGCCGGGTGAAACCAACACGGCGTTCAGTGTTCCCCCACTCAATCAAAGGGAGAATAGGGACGCCAAGGAGCCACTAACTGGAACAATCCTGGAAATGTGGGACGGGGAGATCTACCATTACGGCCTGTATGTGGAACGAGGTCTTGTACTTGGTGTGCACAAACCACCGGCTGCCATCAGCCTCGCCAAGGTTGAACTAACACCACTCTCTCTGTTTTGGAGACCAGTGTATACACCACAGTATCTCATCTCTCCGGACACTCTCAGGAGACTGCACGGAGAGTCGTTTCCCTACACAGCCTTTGATAACAACTGCTATGCCTTCTGTTGTTGGGTCCTGGACCTAAACGACTCGTGGTTGTGCAGGAGAATGATCCAGAGGACAACTGGTTTCTTCAGGCCCTACCAAGACTGGAATAGGAAACCCCTTCCCACCATGGATGACTCCAAGTTGAAGAAGGTAGCTAACATATTCTTGTGCGCGCTATCTTCGCTATTCACTAGGCCCATCAAAGACATAATAGGAAAGTTGAGGCCTCTCAACATCCTTAACATCTTGGCTTCATGTGATTGGACTTTTGCAGGCATAGTGGAATCTTTGATTCTCTTGGCAGAGCTCTTTGGAGTTTTCTGGACACCCCCAGATGTGTCTGCGATGATCGCCCCTTTACTAGGTGACTACGAGCTGCAGGGGCCCGAGGACCTTGCAGTGGAACTCGTTCCAATAGTGATGGGGGGGATTGGTTTGGTGCTAGGGTTCACCAAAGAGAAGATCGGGAAAATGTTGTCATCTGCTGCATCCACCTTAAGAGCTTGTAAAGACCTTGGTGCATACGGACTGGAAATCTTAAAATTGGTCATGAAGTGGTTCTTCCCAAAGAGAGAGGAAGCAAATGAGCTGGCTATGGTGAGGTCCATCGAGGATGCGGTGTTGGACCTCGAGGCAATTGAAAACAACCACATGACTGCCCTCCTCAAAGACAAAGACAGCCTGGCAACCTATATGAGAACTCTTGACCTCGAGGAGGAGAAAGCCAGAAAGCTTTCGACCAAGTCTGCTTCACCTGATATCGTGGGCACAATCAACGCTCTCCTGGCGAGAATCGCCGCTGCACGCTCCCTGGTGCATCGGGCGAAAGAGGAGCTCTCCAGCAGACCAAGACCTGTTGTTGTGATGATATCAGGTAAGCCAGGGATAGGGAAAACCCACCTTGCCAGGGAATTGGCCAAGAAAATCGCAGCTTCTCTCACAGGGGACCAGCGTGTGGGTCTTATCCCGCGCAATGGTGTTGATCACTGGGACGCATATAAGGGAGAAAGAGTCGTTCTATGGGACGACTATGGAATGAGTAACCCCATCCACGACGCCCTCAGGTTACAAGAACTTGCTGACACCTGCCCCCTCACGCTAAATTGTGATAGGATTGAGAATAAAGGAAAGGTCTTTGACAGTGATGCCATAATCATCACCACTAACCTGGCCAACCCAGCACCACTGGACTATGTCAATTTTGAAGCATGCTCGAGGCGTATCGACTTCCTCGTGTATGCAGATGCCCCTGAAGTCGAGAAGGCGAAACGTGATTTCCCAGGTCAACCTGACATGTGGAAGAACGCTTTCAGTCCTGACTTCTCGCACATAAAACTGATGCTGGCTCCGCAGGGTGGCTTCGACAAGAACGGAAACACCCCACATGGGAAAGGCGTCATGAAAACCCTCACTACTGGTTCCCTCATCGCTCGAGCATCAGGGCTACTCCATGAGAGGTTAGATGAGTACGAGTTACAG>GGCCCAACCCCCACTACCTTCAACTTTGACCGCAACAAGGTGCTTGCGTTCAGACAGCTTGCTGCTGAAAACAAGTACGGGTTGATGGACACAATGAGAGTCGGAAAACAGCTCAAGGATGTCAGGACCATGCCAGAGCTCAGACAAGCACTCAAGAACATCTCAATCAAGAGTTGCCAGATAGTGTATGGTGGCTGCACCTATATGCTTGAGTCTGATGGCAAGGGTGATGTGAAAGTTGACAGAGTTCAGAACGCCACTGTGCAGACCAACAATGAACTGGCCGGCGCCCTACACCATCTTAGGTGTGCCAGGATTAGATATTATGTCAAGTGCATTCAGGAGGCCCTGTATTCCATCATCCAAATTGCTGGAGCTGCATTTGTCACCACGCGCATTGTCAAGCGCATGAACATACAAGACCTTTGGTCCAAGCCACAGGTGGAAGATACAGAGGAGACTGCTAGCAAGGATGGGTGCCCAAAACCCAAGGATGATGACGAGTTCGTTGTTTCATCCGACGACATCAAAACCGAGGGCAAGAAAGGAAAGAACAAGTCTGGCCGTGGTAAGAAGCACACAGCATTCTCAAGCAAAGGCCTCAGTGATGAGGAGTACGATGAGTACAAAAGAATCAGAGAAGAAAGAAACGGCAAGTACTCTATAGAGGAATACCTTCAGGACAGAGATAAGTATTATGAGGAGGTGGCCATCGCCAGGGCGACCGAAGAGGACTTCTGTGAAGAAGAAGAGGCCAAGATCCGACAAAGGATTTTTAGGCCAACAAGGAAGCAACGCAAAGAGGAGAGGGCCTCTCTCGGCTTGGTCACAGGTTCTGAAATCAGGAAGAGGAACCCAGACGACTTCAAGCCTAAAGGAAAGCTGTGGGCTGATGACGACAGGAGTGTTGACTACAATGAGAGACTCAATTTTGAAGCCCCACCAAGCATTTGGTCGAGGATAGTCAACTTTGGTTCAGGTTGGGGTTTTTGGGTTTCCCCCAGCCTGTTCATAACATCAACTCATGTCATACCCCAGGGCGCACAGGAGTTCTTTGGGGTTTCCATCAAGCAAATTCAGATACACAAATCGGGTGAATTCTGTCGCTTGAGGTTTCCAAAACCAATCAGAACTGATGTGACAGGCATGATCCTAGAAGAAGGTGCGCCCGAAGGGACCGTGGTCACATTACTCATCAAGAGACCAACTGGAGAACTCATGCCCTTGGCAGCCAGAATGGGAACCCATGCAACCATGAAGATACAAGGGCGCACTGTTGGGGGTCAAATGGGCATGCTCCTAACAGGATCTAACGCCAAGAGTATGGACCTGGGCACCACACCAGGTGACTGTGGCTGTCCCTACATTTACAAGAGAGGGAATGACTACATAGTCATTGGAGTCCACACGGCTGCTGCCCGTGGAGGAAACACTGTCATATGTGCCACCCAGGGGAGCGAGGGGGAAGCCACACTTGAAGGCGGTGACAACAAGGGAACCTACTGCGGTGCACCAATCTTAGGTCCAGGGAGTGCCCCAAAGCTCAGCACCAAGACTAAGTTTTGGAGATCATCCACAGCACCACTCCCACCTGGTACCTATGAACCAGCCTATCTTGGCGGCAAGGACCCCAGAGTCAAGGGTGGCCCCTCATTGCAACAAGTTATGAGGGACCAGCTGAAACCATTCACTGAGCCCAGGGGCAAACCACCAAAACCAAGTGTGTTAGAGGCTGCCAAGAAAACCATCATCAATGTTCTTGAACAAACAATTGACCCACCTCAAAAATGGTCATTCGCGCAGGCATGCGCATCCCTCGACAAGACCACTTCCAGCGGCCACCCGCACCACATGCGGAAGAACGACTGCTGGAACGGGGAGTCCTTCACAGGCAAATTGGCAGACCAGGCTTCCAAGGCTAACCTGATGTTCGAAGAGGGAAAGAACATGACCCCAGTCTACACAGGTGCGCTTAAGGACGAGCTGGTCAAGACTGACAAAATTTATGGCAAGATCAAAAAGAGGCTTCTCTGGGGCTCGGATCTGGCAACCATGATCCGGTGTGCTCGAGCGTTTGGAGGCCTGATGGAGGAACTCAAAGCACATTGTGTCACACTACCCGTCAGAGTAGGTATGAATATGAATGAGGATGGCCCTATCATCTTTGAGAGGCACTCCAGATATAAGTATCATTATGATGCTGATTACTCCCGGTGGGACTCAACACAACAAAGAGCCGTGTTAGCAGCAGCCTTAGAAATCATGGTTAAGTTCTCCCCAGAACCGAATCTGGCCCAAAAGGTTGCAGAAGACCTTCTCTCTCCCAGCGTGATGGACGTAGGTGACTTCAAAATATCAATCAATGAGGGCCTCCCCTCCGGGGTGCCCTGCACCTCCCAATGGAATTCCATCGCCCACTGGCTCCTCACCCTCTGTGCGCTTTCTGAGGTTACAAACCTGTCCCCTGACATTATCCAGGCTAATTCCCTCTTTTCCTTCTACGGTGATGATGAAATTGTGAGCACAGACATAAAATTGGACCCAGAGAAGTTGACAGCAAAACTTAAGGAATACGGGTTGAAACCGACCCGCCCTGACAAGACTGAGGGACCCCTTGTTATCTCTGAGGACCTGGATGGCCTAACCTTCCTGCGGAGGACTGTGACCCGCGACCCAGCTGGCTGGTTTGGAAAGCTGGAACAGAGCTCAATACTTAGGCAAATGTATTGGACTAGGGGCCCTAACCATGAAGACCCATCTGAAACAATGATACCACACTCCCAAAGACCCATACAATTGATGTCTTTGCTGGGCGAGGCTGCACTCCACGGCCCAGCATTCTACAGCAAAATCAGCAAGCTGGTCATTGCAGAGCTGAAGGAAGGTGGCATGGATTTTTACGTGCCCAGACAAGAGCCAATGTTCAGATGGATGAGGTTTTCAGATCTGAGCACGTGGGAGGGCGATCGCAATCTGGCTCCCAGTTTTGTGAATGAAGATGGCGTCGAG

>AB045603|II.12/II.12|1996|JP|Gifu'96

ATGAAGATGGCGTCTAACGACGCTTCCGCTGCCGCTGCTGCTAACAGCAACAACGACACCGCAAAATCTTCAAGTGACGGAGTGCTTTCTAGCATGGCTGTCACTTTTAAACGAGCCCTCGGGGCACGGCCTAAACAGCCTCCCCCGAGGGAAATACCACAAAGGCCCCCACGACCACCCACCCCAGAACTGGTCAAAAAGATCCCTCCTCCTCCACCCAACGGGGAGGATGAACCAGTGGTTTCTTACAGCGTCAAAGATGGCGTTTCCGGCTTGCCTGAGCTTACCACTGTCAGGCAGCCGGGTGAAACCAACACGGCGTTCAGTGTTCCCCCACTCAACCAAAGGGAGAATAGGGACGCCAAGGAGCCACTAACTGGAACAATCCTGGAAATGTGGGACGGGGAGATCTACCATTACGGCCTGTATGTGGAACGAGGTCTTGTACTTGGTGTGCACAAACCACCGGCTGCCATCAGCCTCGCCAAGGTTGAACTAACACCACTCTCTCTGTTTTGGAGACCAGTGTATACACCACAGTATCTCATCTCTCCAGACACTCTCAGGAGACTGCACGGAGAGTCGTTCCCCTACACAGCCTTTGACAACAACTGCTATGCCTTCTGTTGTTGGGTCCTGGACCTAAACGACTCGTGGTTGTGCAGGAGAATGATCCAGAGGACAACTGGTTTCTTCAGGCCTTACCAAGACTGGAATAGGAAACCCCTTCCCACCATGGATGACTCCAAGTTGAAGAAGGTAGCTAACATATTCTTGTGTGCGCTATCTTCGCTATTCACTAGGCCCATCAAAGACATAATAGGAAAGTTGAGGCCTCTTAACATCCTTAACATCTTGGCCTCATGTGATTGGACTTTTGCAGGCATAGTGGAATCTTTGATCCTCCTGGCAGAGCTCTTTGGAGTTTTCTGGACACCCCCAGATGTGTCTGCGATGATCGCCCCTTTACTAGGTGACTACGAGCTGCAGGGGCCCGAGGACCTTGCAGTGGAACTCGTTCCAATAGTGATGGGGGGGATTGGTTTGGTGCTAGGATTCACTAAAGAGAAGATCGGGAAAATGTTGTCATCTGCCGCATCCACCTTAAGAGCTTGTAAAGACCTTGGTGCATACGGACTGGAAATCTTAAAATTGGTCATGAAGTGGTTCTTCCCAAAGAAAGAGGAAGCAAATGAGCTGGCTATGGTGAGGTCCATCGAGGATGCGGTGCTGGACCTCGAGGCAATTGAGAACAATCACATGACTGCCCTCCTCAAAGACAAAGACAGCCTGGCAACCTATATGAGAACTCTTGACCTCGAGGAGGAGAAAGCCAGGAAGCTTTCAACCAAGTCTGCTTCACCTGATATCGTGGGCACAATCAACGCTCTCCTGGCGAGAATCGCCGCTGCACGTTCCCTGGTGCATCGGGCGAAAGAGGAGCTCTCCAGCAGACCAAGACCTGTTGTTGTTATGATATCAGGCAGGCCAGGGATAGGGAAAACCCACCTTGCCAGGGAATTGGCCAAGAGAATCGCAGCTTCACTCACAGGGGACCAGCGTGTGGGTCTCATCCCTCGCAATGGTGTTGATCACTGGGACGCATATAAGGGAGAAAGAGTCGTCCTATGGGACGATTATGGAATGAGTAATCCCATCCACGACGCCCTCAGGTTGCAAGAACTTGCTGACACCTGCCCCCTCACGCTAAATTGTGACAGGATTGAGAACAAGGGAAAGGTCTTTGACAGTGATGCCATAATCATCACCACTAACCTGGCCAACCCAGCACCACTGGACTATGTCAATTTTGAAGCATGCTCGAGGCGCATCGACTTCCTCGTGTATGCAGATGCCCCTGAGGTTGAGAAGGCAAAACGTGATTTCCCAGGTCAACCTGACATGTGGAAGAACGCTTTCAGTCCTGACTTCTCGCACATAAAACTGATGCTGGCTCCGCAGGGTGGCTTCGACAAGAACGGAAACACCCCACATGGGAAAGGCGTCATGAAAACCCTCACCACTGGTTCCCTCATCGCTCGAGCATCAGGGCTACTCCATGAGAGGTTAGATGAGTACGAGCTGCAG>GGCCCAACCCCCACCACCTTCAACTTTGACCGCAACAAGGTGCTTGCGTTCAGACAGCTTGCTGCCGAAAACAAGTACGGGTTGATGGACACAATGAGAGTTGGAAGACAGCTCAAGGATGTCAGGACCATGCCAGAGCTCAAACAAGCACTCAGGAATATCTCAATCAAGAGTTGTCAGATAGTGTATGGTGGCTGCACCTATATGCTTGAGTCTGATGGCAAGGGTGATGTGAAAGTTGACAGAGTTCAGAACGCCACTGTACAGACCAACAATGAACTGGCCGGTGCCCTACACCATCTTAGGTGTGCCAGAATTAGATATTATGTCAAGTGTGTTCAGGAGGCCCTGTATTCCATCATCCAAATTGCTGGAGCTGCATTTGTAACCACGCGCATTGTCAAGCGCATGAACATACAAGACCTTTGGTCCAAGCCACAGGTGGAAGATACAGAGGAGACTGCTAGCAAGGATGGGTGCCCAAAACCCAAGGATGATGACGAGTTCGTTGTCTCATCCGACGACATCAAAACCGAGGGCAAGAAAGGAAAGAACAAGTCTGGCCGTGGTAAGAAGCACACAGCATTCTCAAGCAAAGGTCTCAGTGATGAAGAGTACGATGAGTACAAAAGAATCAGAGAAGAAAGAAACGGCAAATACTCCATAGAGGAATACCTTCAGGACAGAGACAAGTATTATGAGGAGGTGGCCATCGCCAGGGCGACCGAAGAGGACTTCTGTGAAGAAGAAGAGGCCAAGATCCGACAAAGGATTTTCAGGCCAACAAGGAAACAACGCAAAGAGGAGAGGGCCTCTCTCGGTTTGGTCACAGGCTCTGAAATCAGGAAGAGGAACCCAGACGACTTCAAGCCTAAAGGAAAGCTGTGGGCTGATGACGACAGGAGTGTTGACTACAATGAGAGACTCAATTTTGAAGCCCCACCAAGCATTTGGTCGAGGATAGTCAACTTTGGTTCAGGTTGGGGCTTTTGGGTTTCCCCCAGCCTGTTCATAACATCAACTCATGTCATACCCCAGGGCGCACAGGAGTTCTTTGGGGTTTCCATCAAACAAATTCAGATACACAAATCGGGTGAATTCTGTCGCTTAAGGTTTCCAAAACCAATCAGAACTGATGTGACAGGCATGATCCTAGAAGAAGGTGCGCCCGAAGGGACCGTGGTCACACTACTCATCAAGAGGCCAACTGGAGAACTCATGCCCTTGGCAGCCAGAATGGGAACCCATGCAACCATGAAGATTCAAGGGCGCACTGTTGGGGGTCAAATGGGCATGCTCCTAACAGGATCCAACGCCAAGAGTATGGATCTGGGCACCACACCAGGTGACTGTGGCTGTCCCTACATTTACAAGAGGGGGAATGACTACGTAGTCATTGGAGTCCACACGGCTGCTGCCCGTGGAGGGAACACTGTCATATGTGCCACCCAGGGGAGCGAGGGAGAAGCCACACTTGAAGGCGGTGACAACAAGGGAACCTACTGTGGTGCACCAATCTTAGGTCCAGGAAGTGCCCCAAAGCTCAGCACCAAGACTAAGTTTTGGAGATCATCCACAGCACCACTCCCACCTGGTACCTATGAACCAGCCTACCTTGGCGGCAAGGACCCCAGAGTCAAGGGTGGCCCTTCATTGCAACAAGTTATGAGGGACCAGCTGAAACCATTCACTGAGCCCAGGGGTAAACCACCAAAACCAAGTGTGTTAGAGGCTGCCAAGAAAACCATCATCAATGTTCTTGAACAAACAATTGATCCACCTCAAAAATGGTCATTCGCGCAGGCATGCGCATCCCTCGACAAGACCACTTCCAGTGGTCACCCGCACCACATGCGGAAAAACGACTGCTGGAACGGGGAGTCCTTTACAGGCAAATTGGCAGACCAGGCTTCCAAGGCCAACCTGATGTTCGAAGAGGGAAAGAATATGACCCCAGTCTATACAGGTGCGCTTAAGGACGAGCTGGTCAAGACTGACAAAATTTATGGCAGGATCAAAAAGAGGCTTCTCTGGGGCTCGGATCTGGCAACCATGATCCGGTGCGCTCGAGCGTTTGGGGGCCTGATGGAGGAACTCAAAGCACATTGTGTCACACTACCCGTCAGAGTAGGTATGAATATGAATGAGGATGGTCCTATCATCTTTGAGAGACACTCCAGATACAAATACCACTATGATGCTGATTACTCCCGGTGGGACTCAACACAACAAAGAGCCGTGTTAGCAGCAGCCTTAGAAATCATGGTTAAGTTCTCCCCAGAACCGCATCTGGCCCAAAAGGTTGCAGAAGACCTTCTCTCTCCCAGCGTGATGGACGTAGGTGACTTCAAAATATCAATCAATGAGGGCCTCCCCTCCGGGGTGCCCTGCACCTCCCAATGGAATTCCATCGCCCACTGGCTCCTCACTCTCTGTGCACTTTCTGAGGTTACAAACCTGTCCCCTGACATTATCCAGGCCAACTCCCTCTTTTCCTTCTACGGTGATGATGAAATTGTGAGCACAGACATAAAGTTGGACCCAGAGAAGTTGACAGCAAAACTTAAGGAATACGGGTTGAAACCGACCCGCCCTGACAAGACTGAGGGACCCCTTGTTATCTCTGAGGACCTGAATGGCCTAACCTTCCTGCGGAGGACTGTGACCCGCGACCCAGCTGGTTGGTTTGGAAAGTTGGAACAGAGTTCAATACTTAGGCAAATGTATTGGACTAGGGGCCCTAACCATGAAGACCCATCTGAAACAATGATACCACACTCCCAAAGACCCATACAATTGATGTCTTTGCTGGGCGAGGCTGCACTCCACGGCCCAGCATTCTACAGCAAAATCAGCAAGCTGGTCATTGCAGAGCTGAAGGAAGGTGGCATGGATTTTTACGTGCCCAGACAAGAGCCAATGTTCAGATGGATGAGGTTCTCAGATCTGAGCACGTGGGAGGGCGATCGCAATCTGGCTCCCAGTTTTGTGAATGAAGATGGCGTCGAG

>AB220921|II.12/II.4-03|2005|JP|Chiba/04-1050

ATGAAGATGGCGTCTAACGACGCTTCCGCTGCCGCTGCTGCTAACAGCAACAACGACACCGCAAAATCTTCAAGTGACGGAGTGCTTTCTAGCATGGCTGTCACTTTTAAACGAGCCCTCGGGGCGCGGCATAAACAGCCTCCCCCGAGGGACATACCACAAAGACCTCCACGACCACCCACCCCGGAACTTGCCAAAAAGATCCCTCCTCCCCCACCCAACGGGGAGGATGAACTAGTGGTTTCTTACAGCGCCAAAGATGGCGTTTCCGGTTTGCCTGAGCTTACCACTGTCTGGCAGCCGGATGAAACCAACACGGCGTTCAGTGTTCCCCCACTCAACCAGAGGGAGAATAGGGACGCCAAGGAGCCGCTAACCGGAACAATCCTGGAAATGTGGGACGGGGAGATCTATCATTACGGTCTGTATGTGGAACGAGGCCTTGTACTTGGTGTGCACAAACCACCAGCTGCCATCAGCCTCGCCAAGGTTGAGCTAGCACCACTCTCTCTGTTTTGGAGACCAGTGTATACACCACAGTATCTCATCTCCCCAGACACTCTCAGGAGACTGCATGGAGAGTCGTTCCCCTACACAGCCTTTGACAACAACTGCTATGCCTTCTGTTGTTGGGTCCTGGATCTAAACGACTCGTGGTTGTGCAGGAGAATGATCCAGAGGACAACTGGTTTCTTCAGACCTTACCAAGACTGGAACAGGAAACCCCTCCCCACCATGGATGACTCCAAATTGAAGAAGGTAGCCAACATATTCTTGTGTGCGCTATCTTCGCTATTCACTAGGCCCATCAAAGACATAATAGGAAAGGTGAGACCGCTTAACATCCTTAACATCTTGGCCTCGTGTGATTGGACTTTTGCAGGCATAGTGGAATCTTTGATCCTCTTGGCAGAGCTCTTTGGAGTTTTCTGGACACCCCCAGATGTGTCTGCGATGATCGCCCCCTTACTAGGTGACTACGAACTGCAGGGGCCCGAGGACCTTGCAGTGGAACTCGTTCCAGTAGTGATGGGGGGGATTGGTTTGGTGCTAGGATTCACTAAAGAGAAGATCGGAAAAATGTTGTCATCTGCTGCATCCACCTTAAGGGCTTGTAAAGACCTTGGTGCATACGGATTGGAAATCCTAAAATTGGTCATGAAGTGGTTCTTCCCAAAGAAAGAGGAAGCAAATGAGTTGGCTATGGTGAGGTCCATCGAGGATGCGGTGCTGGACCTCGAAGCAATTGAGAACAATCACATGACTGCCCTCCTCAAAGACAAAGACAGCTTGGCAACCTACATGAGAACCCTTGACCTCGAGGAGGAGAAGGCCAGGAAGCTTTCAACCAAGTCTGCTTCACCTGACATCGTGGGCACAATCAACGCTCTCCTGGCGAGAATCGCCGCTGCACGTTCCCTGGTGCATCGGGCGAAAGAGGAACTCTCCAGCAGACCAAGACCTGTTGTTGTTATGATATCAGGCAGGCCAGGGATAGGGAAAACTCACCTTGCTAGGGAACTGGCCAAGAAAATCGCAGCTTCGCTTACAGGGGACCAGCGTGTGGGTCTCATCCCTCGCAATGGCGTTGATCACTGGGATGCATATAAGGGGGAAAGAGTCGTCCTGTGGGACGATTATGGAATGAGTAATCCCATCCACGACGCCCTCAGGTTACAGGAACTTGGTGACACCTGCCCCCTCACGCTAAACTGTGACAGGATTGAGAACAAAGGAAAGGTCTTTGACAGTGATGCCATAATCATCACCACTAACCTGGCCAACCCAGCACCATTGGACTATGTCAACTTTGAAGCATGCTCGAGGCGCATCGACTTCCTCGTGTACGCAGAAGCCCCTGAGGTCGAGAAGGCAAAGCGTGATTTCCCAGGTCAACCTGACATGTGGAAGAATGCATTCAGTTCTGACTTCTCGCACATAAAACTGACGCTGGCTCCGCAAGGTGGCTTCGACAAGAACGGAAACACCCCACCCGGGAAAGGCGTCATGAAAACCCTCACCACTGGTTCCCTCATCGCCCGAGCATCAGGGCTACTCCATGAGAGGTTAGATGAGTATGAGCTGCAG>GGCCCAACCCCCACCACCTTTAACTTTGACCGCAACAAGGTGCTTGCGTTCAGACAGCTTGCTGCCGAAAACAAGTACGGGTTGTTGGACACAATGAGAGTTGGAAAACAGCTCAAGGATGTCAAGACCATGCCAGAGCTCAAACAAGCGCTCAAAAATGTCGCAATCAAGAATTGTCAGATAGTGTATGGTGGCTGCACTTACAGGCTTGAGTCTGATGGCAAGGGTGATGTGAAGGTTGACAGAGTTCAGAACGCCACTGTACAGACCAACAATGAGCTTGCCGGCGCCCTACATCATCTTAGGTGTGCCAGAATCAGATATTATGTCAAGTGTGTCCAGGAGGCCCTGTATTCCATCATCCAAATTGCTGGAGCTGCATTTGTAACCACGCGCATTGTCAAGCGCATGAACATACAAGACCTCTGGTCCAAGCCACAGGTGGAGGACACAGAGGAGACCGCTAGCAAAGATGGGTGCCCAAAACCCAAGGATGATGACGAGTTCGTTGTCTCATCCGATGACATCAAAACCGAGGGCAAGAAAGGGAAGAACAAGTCTGGCCGTGGTAAGAAGCACACAGCATTCTCAAGCAAAGGTCTCAGTGATGAAGAGTACGATGAGTACAAAAGAATCAGAGAAGAAAGGAACGGCAAATACTCCATAGAAGAATACCTTCAGGACAGAGATAAGTATTATGAGGAGGTGGCCATCGCCAGGGCGACCGAAGAGGACTTCTGTGAAGAAGAAGAGGCCAAGATCCGACAAAGGATTTTCAGGCCAACAAGGAAACAACGCAAAGAGGAGAGGGCCTCTCTCGGTTTGGTCACAGGCTCTGAAATCAGGAAGAGGAACCCAGACGACTTCAAGCCTAAAGGAAAGCTGTGGGCTGATGATGACAGGAGTGTTGACTACAATGAGAGACTCAATTTTGAAGCCCCACCAAGCATCTGGTCGAGGATAGTCAATTTTGGTTCGGGTTGGGGCTTTTGGGTTTCCCCCAGCCTGTTCATAACATCAACTCATGTCATACCCCAGGGCGCACAGGAGTTCTTTGGTGTTCCCATCAAACAAATTCAGATACACAAATCGGGAGAATTCTGTCGTTTGAGGTTTCCAAAACCAATCAGAACTGACGTGACAGGCATGATCTTAGAAGAAGGTGCACCCGAAGGGACCGTGGTCACACTACTCATTAAGAGGCCAACTGGAGAACTCATGCCCTTGGCAGCCAGAATGGGAACCCATGCAACCATGAAGATTCAAGGGCGCACTGTTGGAGGTCAAATGGGCATGCTCCTAACAGGATCCAACGCCAAGAGTATGGACCTGGGCACCACACCAGGTGACTGTGGCTGTCCCTACATTTACAAGAGGGGGAATGACTATGTAGTCATTGGAGTCCACACAGCTGCCGCCCGTGGAGGAAACACTGTCATATGTGCCACCCAAGGGAGCGAGGGAGAAGCCACACTTGAGGGCGGTGACAGCAAGGGGACCTACTGCGGTGCACCAATCTTAGGTCCAGGAAGTGCCCCAAAGCTCAGCACCAAGACTAAATTCTGGAGATCATCCACGGCACCACTCCCACCTGGTACCTATGAGCCAGCCTACCTCGGCGGCAAGGACCCCAGAGTTAAGGGTGGCCCTTCATTGCAACAAGTTATGAGGGACCAGCTGAAACCATTCACTGAACCCAGGGGTAAACCACCAAAACCAAGTGTGTTAGAAGCTGCCAAGAAAACCATCGTCAATGTCCTTGAACAAACAATTGATCCACCTCAAAAATGGTCATTTGCGCAGGCATGCGCATCCCTTGACAAGACCACTTCCAGTGGTCACCCGCACCACATGCGGAAAAACGACTGCTGGAACGGGGAATCCTTTACAGGCAAATTGGCAGACCAGGCTTCCAAGGCCAACCTGATGTTCGAGGAGGGAAAGAACATGACCCCAGTTTACACGGGTGCGCTTAAGGACGAGCTAGTCAAGACTGATAAAATTTATGGCAAGATCAAAAAGAGGCTTCTCTGGGGTTCGGACCTGGCGACCATGATCCGGTGCGCTCGAGCATTCGGGGGCCTGATGGAGGAACTCAAAGCACATTGTGTCACACTACCTGTCAGAGTGGGTATGAATATGAATGAGGATGGTCCTATCATCTTTGAGAGACACTCCAGATATAAATATCATTATGATGCTGATTACTCCCGGTGGGACTCAACACAACAAAGAGCCGTGTTAGCAGCGGCTTTAGAAATCATGGTCAAGTTCTCCCCAGAGCCGCATCTGGCCCAAAAGGTTGCAGAAGACCTTCTTTCTCCCAGCGTGATGGACGTGGGTGATTTCAAAATAACAATCAATGAGGGTCTCCCCTCCGGGGTGCCCTGCACCTCCCAATGGAATTCCATCGCCCACTGGCTCCTCACTCTATGTGCACTCTCTGAGGTTACAAACCTGTCCCCTGACATTATCCAGGCCAATTCTCTCTTTTCTTTCTACGGTGATGATGAAATTGTGAGCACAGACATAAAATTGGACCCAGAAAAGCTGACAGCCAAACTCAAGGAATACGGGTTGAAACCGACCCGCCCTGACAAGACTGAGGGACCCCTTGTTATCTCTGAAGACCTGAATGGCCTAACCTTCCTGCGGAGGACCGTGACCCGCGACCCAGCAGGCTGGTTTGGAAAGTTGGAACAGAGTTCAATACTCAGACAAATGTATTGGACTAGGGGCCCCAACCATGAAGACCCATCTGAAACAATGATACCACACTCCCAGAGGCCCATACAATTGATGTCTTTGCTGGGTGAGGCTGCACTCCACGGCCCAGCATTCTACAGCAAAATCAGCAAACTGGTCATCGCAGAGTTGAAGGAAGGTGGCATGGATTTTTACGTGCCAAGACAAGAGCCAATGTTCAGATGGATGAGATTCTCGGATCTGAGCACGTGGGAGGGCGATCGCAATCTGGCTCCCAGTTTTGTGAATGAAGATGGCGTCGAA

>AB220922|II.12/II.4-03|2005|JP|Sakai/04-179

CTTCCGCTGCCGCTGCTGCTAACAGCAACAACGACACCGCAAAATCTTCAAGTGACGGAGTGCTTTCTAGCATGGCTGTCACTTTTAAACGAGCCCTCGGGGCGCGGCATAAACAGCCTCCCCCGAGGGAAATGCCACAAAGACCTCCACGACCACCCACCCCGGAACTTGCCAAAAAGATCCCTCCCCCCCCACCCAACGGGGAGGATGAACTAGTGGTTTCTTACAGCGCCAAAGATGGCGTTTCCGGTTTGCCTGAGCTTACCACTGTCTGGCAGCCGGATGAAACCAACACGGCGTTCAGTGTTCCCCCACTCAACCAGAGGGAGAATAGGGACGCCAAGGAGCCGCTAACCGGAACAATACTGGAAATGTGGGACGGGGAAATCTATCATTACGGTCTGTATGTGGAACGAGGCCTTGTACTTGGTGTGCACAAACCACCAGCTGCCATCAGCCTCGCTAAGGTTGAGCTAGCACCACTCTCTCTGTTTTGGAGACCAGTGTATACACCACAGTATCTCATCTCTCCAGACACTCTCAGGAGACTGCATGGAGAGTCGTTCCCCTACACAGCCTTTGACAACAACTGCTATGCCTTCTGTTGTTGGGTCCTGGACCTAAACGACTCGTGGTTGTGCAGGAGAATGATCCAGAGGACAACTGGTTTCTTCAGACCTTACCAAGACTGGAACAGGAAACCCCTCCCCACCATGGATGACTCCAAATTGAAGAAGGTAGCCAACATATTCTTGTGTGCGCTATCTTCGCTATTCACTAGGCCCATCAAAGACATAATAGGAAAGGTGAGACCGCTTAACATCCTTAACATCTTGGCCTCATGTGATTGGACTTTTGCAGGCATAGTGGAATCTTTGATCCTCTTGGCAGAGCTCTTTGGAGTTTTCTGGACACCCCCAGATGTGTCTGCGATGATCGCTCCCTTACTAGGTGACTACGAACTGCAGGGTCCCGAGGACCTTGCAGTGGAACTCGTTCCAGTAGTGATGGGGGGGATTGGTTTGGTACTAGGATTCACCAAAGAGAAGATTGGAAAAATGTTGTCATCTGCTGCATCCACCTTAAGGGCTTGTAAAGACCTTGGTGCATACGGACTGGAAATCCTAAAATTGGTCATGAAGTGGTTCTTCCCAAAGAAAGAGGAAGCAAATGAGTTGGCTATGGTGAGGTCCATCGAGGATGCGGTGCTGGACCTCGAAGCGATTGAGAACAATCACATGACTGCCCTCCTCAAAGACAAAGACAGCTTGGCAACCTACATGAGAACCCTTGACCTCGAGGAGGAGAAGGCCAGGAAGCTTTCAACCAAGTCTGCTTCACCTGACATCGTGGGCACAATCAACGCTCTCCTGGCGAGAATCGCCGCTGCACGCTCCCTGGTGCATCGGGCGAAAGAGGAACTCTCCAGCAGACCAAGACCTGTTGTTGTTATGATATCAGGCAGGCCAGGGATAGGGAAAACTCACCTTGCTAGGGAACTGGCCAAGAAAATCGCAGCTTCGCTTACAGGGGACCAGCGTGTGGGTCTCATCCCTCGCAATGGCGTTGACCACTGGGATGCATATAAGGGGGAAAGAGTCGTCCTATGGGACGATTATGGAATGAGTAATCCCATCCACGACGCCCTCAGGTTACAGGAACTTGCTGACACCTGCCCCCTCACGCTAAACTGTGACAGGATTGAGAACAAAGGAAAGGTCTTTGACAGTGATGCCATAATCATCACCACTAATCTGGCCAACCCAGCACCATTGGACTATGTCAACTTTGAAGCATGCTCGAGGCGCATCGACTTCCTCGTGTACGCAGAAGCCCCTGAGGTCGAGAAGGCAAAGCGTGATTTCCCAGGTCAACCTGACATGTGGAAGAACGCATTCAGTTCTGACTTCTCGCACATAAAACTGACGCTGGCTCCGCAAGGTGGCTTCGACAAGAACGGAAACACCCCACACGGGAAAGGCGTCATGAAAACCCTCACCACTGGTTCCCTCATCGCCCGAGCATCAGGGCTACTCCATGAGAGGTTAGATGAGTATGAGCTGCAG>GGCCCAACCCCCACCACCTTTAACTTTGACCGCAACAAGGTGCTTGCGTTCAGACAGCTTGCTGCCGAAAACAAGTACGGGTTGTTGGACACAATGAGAGTTGGGAAACAGCTCAAGGATGTCAAGACCATGCCAGAGCTCAAACAAGCGCTCAAAAATGTCGCAATCAAGAATTGTCAGATAGTGTATGGTGGCTGCACTTACAGGCTTGAGTCTGATGGCAAGGGTGATGTGAAAGTTGACAGAGTTCAGAACGCCATTGTACAGACCAACAATGAGCTTGCCGGCGCCCTACATCATCTTAGGTGTGCCAGAATCAGATATTATGTCAAGTGTGTCCAGGAGGCCCTGTATTCCATCATCCAAATTGCTGGAGCTGCATTTGTAACCACGCGCATTGTCAAGCGCATGAACATACAAGACCTCTGGTCCAAGCCACAGGTGGAGGACACAGAGGAGACCGCTAGCAATGATGGTTGCCCAAAACCCAAGGATGATGACGAGTTCGTTGTCTCATCCGATGACATCAAAACCGAGGGCAAGAAAGGGAAAAACAAGTCTGGCCGTGGTAAGAAGCACACAGCATTCTCAAGCAAAGGTCTCAGTGATGAAGAGTACGATGAGTACAAAAGAATCAGAGAAGAAAGGAATGGCAAATACTCCATAGAGGAATACCTTCAGGACAGAGATAAGTATTATGAGGAGGTGGCCATCGCCAGGGCAACCGAAGAGGACTTCTGTGAAGAAGAAGAGGCCAAGATCCGACAAAGGATTTTCAGGCCAACAAGGAAACAACGCAAAGAGGAAAGGGCCTCTCTCGGTTTGGTCACAGGCTCTGAAATCAGGAAGAGGAACCCAGACGACTTCAAGCCTAAAGGAAAGCTGTGGGCTGATGATGACAGGAGTGTTGACTACAATGAGAAACTCAATTTTGAAGCCCCACCAAGCATCTGGTCGAGGATAGTCAATTTTGGTTCGGGTTGGGGTTTTTGGGTTTCCCCCAGCCTGTTCATAACATCAACTCATGTCATACCCCAGGGCGCACAGGAGTTCTTTGGTGTTCCCATCAAACAAATTCAGATACACAAATCGGGTGAATTCTGTCGTTTGAGGTTTCCAAAACCAATCAGAACTGACGTGACAGGCATGATCTTAGAAGAAGGTGCACCCGAAGGGACCGTGGTCACACTACTCATTAAGAGGCCAACTGGAGAACTCATGCCCTTGGCAGCCAGAATGGGAACCCATGCAACCATGAAGATTCAAGGGCGCACTGTTGGAGGTCAAATGGGCATGCTCCTAACAGGATCCAACGCCAAGAGTATGGACCTGGGCACCACACCAGGTGACTGTGGCTGTCCCTATATTTACAAGAGGGGGAATGACTATGTAGTCATTGGAGTCCACACAGCTGCCGCCCGTGGAGGAAACACTGTCATATGTGCCACCCAAGGGAGCGAGGGAGAAGCCACACTTGAAGGCGGTGACAGCAAGGGGACCTACTGCGGTGCACCAATCTTAGGTCCAGGAAGTGCCCCAAAGCTCAGCACCAAGACTAAATTCTGGAGATCATCCACGGCACCACTCCCACCTGGTACCTATGAGCCAGCCTACCTCGGCGGCAAGGACCCCAGAGTTAAGGGTGGCCCTTCATTGCAACAAGTTATGAGGGACCAGCTGAAACCATTCACTGAACCCAGGGGTAAGCCACCAAAACCAAGTGTGTTAGAAGCTGCCAAGAAAACCATCGTCAATGTCCTTGAACAAACAATTGATCCACCTCAAAAATGGTCATTTGCGCAGGCATGCGCATCCCTTGACAAGACCACTTCCAGTGGCCACCCGCACCACATGCGGAAAAACGACTGCTGGAACGGGGAATCCTTTACAGGCAAATTGGCAGACCAGGCTTCCAAGGCCAACCTGATGTTTGAGGAGGGAAAGAACATGACCCCAGTCTACACGGGTGCGCTTAAGGACGAGCTGGTCAAGACTGACAAAATTTATGGCAAGATCAAAAAGAGGCTTCTCTGGGGTTCGGACCTGGCGACCATGATCCGGTGCGCTCGAGCATTCGGGGGCCTGATGGAGGAACTCAAAGCACATTGTGTCACACTACCTGTCAGAGTGGGTATGAATATGAATGAGGATGGTCCTATCATCTTTGAGAGACACTCCAGATATAAATACCATTATGATGCTGATTACTCCCGGTGGGACTCAACACAACAAAGAGCCGTGTTAGCAGCGGCTTTAGAAATCATGGTCAAGTTCTCCCCAGAGCCGCATCTGGCCCAAAAGGTTGCAGAAGACCTTCTTTCTCCCAGCGTGATGGACGTGGGTGATTTCAAAATATCAATTAATGAGGGTCTCCCCTCCGGGGTGCCCTGCACCTCCCAATGGAATTCCATCGCCCACTGGCTCCTCACTCTATGTGCACTCTCTGAGGTTACAAACCTGTCCCCTGACATTATCCAGGCCAACTCTCTCTTTTCTTTCTACGGTGATGATGAAATTGTGAGCACAGACATAAAATTGGACCCAGAAAAGCTGACAGCAAAACTCAAAGAATACGGGTTGAAACCGACCCGCCCTGACAAGACTGAGGGACCCCTTGTTATCTCTGAAGACCTGAATGGCCTAACCTTCCTGCGGAGGACCGTGACCCGCGATCCAGCAGGCTGGTTTGGAAAGTTGGAACAGAGTTCAATACTCAGACAAATGTATTGGACTAGGGGCCCCAACCATGAAGACCCATCTGAAACAATGATACCACACTCCCAGAGGCCCATACAATTGATGTCTTTGCTGGGTGAGGCTGCACTCCACGGCCCAGCATTCTACAGCAAAATCAGCAAACTGGTCATTGCAGAGTTGAAGGAAGGTGGCATGGATTTTTACGTGCCAAGACAGGAGCCAATGTTCAGATGGATGAGATTCTCGGATCTGAGCACGTGGGAGGGCGATCGCAATCTGGCTCCCAGTTTTGTGAATGAAGATGGCGTCGAA

>AB220923|II.12/II.4-03|2005|JP|Ehime/05-30

CTTCCGCTGCCGCTGCTGCTAACAGCAACAACGACACCGCAAAATCTTCAAGTGACGGAGTGCTTTCTAGCATGGCTGTCACTTTTAAACGAGCCCTCGGGGCGCGGCATAAACAGCCTCCCCCGAGGGAAATGCCACAAAGACCTCCACGACCACCCACCCCGGAACTTGCCAAAAAGATCCCTCCCCCCCCACCCAACGGGGAGGATGAACTAGTGGTTTCTTACAGCGCCAAAGATGGCGTTTCCGGTTTGCCTGAGCTTACCACTGTCTGGCAGCCGGGTGAGACCAACACGGCGTTCAGTGTTCCCCCACTCAACCAGAGGGAGAATAGGGACGCCAAGGAGCCGCTAACCGGAACAATCCTGGAAATGTGGGACGGGGAGATCTATCATTACGGTCTGTATGTGGAACGAGGCCTTGTACTTGGTGTGCACAAACCACCAGCTGCCATCAGCCTCGCTAAGGTTGAGCTAGCACCACTCTCTCTGTTTTGGAGACCAGTGTATACACCACAGTATCTCATCTCTCCAGACACTCTCAGGAGACTGCATGGAGAGTCGTTCCCCTACACAGCCTTTGACAACAACTGCTATGCCTTCTGTTGTTGGGTCCTGGACCTAAACGACTCGTGGTTGTGCAGGAGAATGATCCAGAGGACAACTGGTTTCTTCAGACCTTACCAAGACTGGAACAGGAAACCCCTCCCCACCATGGATGACTCCAAATTGAAGAAGGTAGCCAACATATTCTTGTGTGCGCTATCTTCGCTATTCACTAGGCCCATCAAAGACATAATAGGAAAGGTGAGACCGCTTAACATCCTTAACATCTTGGCCTCATGTGATTGGACTTTTGCAGGCATAGTGGAATCTTTGATCCTCTTGGCAGAGCTCTTTGGAGTTTTCTGGACACCCCCAGATGTGTCTGCGATGATCGCCCCCTTACTAGGTGACTACGAACTGCAGGGGCCCGAGGACCTTGCAGTGGAACTCGTTCCAGTAGTGATGGGGGGGATTGGTTTGGTACTAGGATTCACCAAAGAGAAGATTGGAAAAATGTTGTCATCTGCTGCATCCACCTTAAGGGCTTGTAAAGACCTTGGTGCATACGGACTGGAAATCCTAAAATTGGTCATGAAGTGGTTCTTCCCAAAGAAAGAGGAAGCAAATGAGTTGGCTATGGTGAGGTCCATCGAGGATGCGGTGCTGGACCTCGAAGCGATTGAGAACAATCACATGACTGCCCTCCTCAAAGACAAAGACAGCTTGGCAACCTACATGAGAACCCTTGACCTCGAGGAGGAGAAGGCCAGGAAGCTTTCAACCAAGTCTGCTTCACCTGACATCGTGGGCACAATCAACGCTCTCCTGGCGAGAATCGCCGCTGCACGCTCCCTGGTGCATCGGGCGAAAGAGGAACTCTCCAGCAGACCAAGACCTGTTGTTGTTATGATATCAGGCAGGCCAGGGATAGGGAAAACTCACCTTGCTAGGGAACTGGCCAAGAAAATCGCAGCTTCGCTTACAGGGGACCAGCGTGTGGGTCTCATCCCTCGCAATGGCGTTGATCACTGGGATGCATATAAGGGGGAAAGAGTCGTCCTATGGGACGATTATGGAATGAGTAATCCCATCCACGACGCCCTCAGGTTACAGGAACTTGCTGACACCTGCCCCCTCACGCTAAACTGTGACAGGATTGAGAACAAAGGAAAGGTCTTTGACAGTGATGCCATAATCATCACCACTAATCTGGCCAACCCAGCACCATTGGACTATGTCAACTTTGAAGCATGCTCGAGGCGCATCGATTTCCTCGTGTACGCAGAAGCCCCTGAGGTCGAGAAGGCAAAGCGTGATTTCCCAGGTCAACCTGACATGTGGAAGAACGCATTCAGTTCTGACTTCTCGCACATAAAACTGACGCTGGCTCCGCAAGGTGGCTTCGACAAGAACGGAAACACCCCACACGGGAAAGGCGTCATGAAAACCCTCACCACTGGTTCCCTCATCGCCCGAGCATCAGGGCTACTCCATGAGAGGTTAGATGAGTATGAGCTGCAG>GGCCCAACCCCCACCACCTTTAACTTTGACCGCAACAAGGTGCTTGCGTTCAGACAGCTTGCTGCCGAAAACAAGTACGGGTTGTTGGACACAATGAGAGTTGGAAAACAGCTCAAGGATGTCAAGACCATGCCAGAGCTCAAACAAGCGCTCAAAAATGTCGCAATCAAGAATTGCCAGATAGTGTATGGTGGCTGCACTTACAGGCTTGAGTCTGATGGCAAGGGTGATGTGAAAGTTGACAGAGTTCAGAACGCCATTGTACAGACCAACAATGAGCTTGCCGGCGCCCTACATCATCTTAGGTGTGCCAGAATCAGATATTATGTCAAGTGTGTCCAGGAGGCCCTGTATTCCATCATCCAAATTGCTGGAGCTGCATTTGTAACCACGCGCATTGTCAAGCGCATGAACATACAAGACCTCTGGTCCAAGCCACAGGTGGAGGACACAGAGGAGACCGCTAGCAATGATGGTTGCCCAAAATCCAAGGATGATGACGAGTTCGTTGTCTCATCCGATGACATCAAGACCGAGGGCAAGAAAGGGAAGAACAAGTCTGGCCGTGGTAAGAAGCACACAGCATTCTCAAGCAAAGGTCTCAGTGATGAAGAGTACGATGAGTACAAAAGAATCAGAGAAGAAAGGAACGGCAAATACTCCATAGAGGAATACCTTCAGGACAGAGATAAGTATTATGAGGAGGTGGCCATCGCCAGGGCAACCGAAGAGGACTTCTGTGAAGAAGAAGAGGCCAAGATCCGACAAAGGATTTTCAGGCCAACAAGGAAACAACGCAAAGAGGAAAGGGCCTCTCTCGGTTTGGTCACAGGCTCTGAAATCAGGAAGAGGAACCCAGACGACTTCAAGCCTAAAGGAAAGCTGTGGGCTGATGATGACAGGAGTGTTGACTACAATGAGAAACTCAGTTTTGAAGCCCCACCAAGCATCTGGTCGAGGATAGTCAATTTTGGTTCGGGTTGGGGTTTTTGGGTTTCCCCCAGCCTGTTCATAACATCAACTCATGTCATACCCCAGGGCGCACAGGAGTTCTTTGGTGTTCCCATCAAACAAATTCAGATACACAAATCGGGTGAATTCTGTCGTTTGAGGTTTCCAAAACCAATCAGAACTGACGTGACAGGCATGATCTTAGAAGAAGGTGCACCCGAAGGGACCGTGGTCACACTACTCATTAAGAGGCCAACTGGAGAACTCATGCCCTTGGCAGCCAGAATGGGAACCCATGCAACCATGAAGATTCAAGGGCGCACTGTTGGAGGTCAAATGGGCATGCTCCTAACAGGATCCAACGCCAAGAGTATGGACCTGGGCACCACACCAGGTGACTGTGGCTGTCCCTATATTTACAAGAGGGGGAATGACTATGTAGTCATTGGAGTCCACACAGCTGCCGCCCGTGGAGGAAACACTGTCATATGTGCCACCCAAGGGAGCGAGGGAGAAGCCACACTTGAAGGCGGTGACAGCAAGGGGACCTACTGCGGTGCACCAATCTTAGGTCCAGGAAGTGCCCCAAAGCTCAGCACCAAGACTAAATTCTGGAGATCATCCACGGCACCACTCCCACCTGGTACCTATGAGCCAGCCTACCTCGGCGGCAAAGACCCCAGAGTTAAGGGTGGCCCTTCATTGCAACAAGTTATGAGGGACCAGCTGAAACCATTCACTGAACCCAGGGGTAAACCACCAAAACCAAGTGTGTTAGAAGCTGCCAAGAAAACCATCGTCAATGTCCTTGAACAAACAATTGATCCACCTCAAAAATGGTCATTTGCGCAGGCATGCGCATCCCTTGACAAGACCACTTCCAGTGGTCACCCGCACCACATGCGGAAAAACGACTGCTGGAACGGGGAATCCTTTACAGGCAAATTGGCAGACCAGGCTTCCAAGGCCAACCTGATGTTTGAGGAGGGAAAGAACATGACCCCAGTCTACACGGGTGCGCTTAAGGACGAGCTGGTCAAGACTGACAAAATTTATGGCAAGATCAAAAAGAGGCTTCTCTGGGGTTCGGACCTGGCGACCATGATCCGGTGCGCTCGAGCATTCGGGGGCCTGATGGAGGAACTCAAAGCACATTGTGTCACACTACCTGTCAGAGTGGGTATGAATATGAATGAGGATGGTCCTATCATCTTTGAGAGACACTCCAGATATAAATACCATTATGATGCTGATTACTCCCGGTGGGACTCAACACAACAAAGAGCCGTGTTAGCAGCGGCTTTAGAAATCATGGTCAAGTTCTCCCCAGAGCCGCATCTGGCCCAAAAGGTTGCAGAAGACCTTCTTTCTCCCAGCGTGATGGACGTGGGTGATTTCAAAATATCAATCAATGAGGGTCTCCCCTCCGGGGTGCCCTGCACCTCCCAATGGAATTCCATCGCCCACTGGCTCCTCACTCTATGTGCACTCTCTGAGGTTACAAACCTGTCCCCTGACATTATCCAGGCCAACTCTCTCTTTTCTTTCTACGGTGATGATGAAATTGTGAGCACAGACATAAAATTGGACCCAGAAAAGCTGACAGCAAAACTCAAAGAATACGGGTTGAAACCGACCCGCCCTGACAAGACTGAGGGACCCCTTGTTATCTCTGAAGACCTGAATGGCCTAACCTTCCTGCGGAGGACCGTGACCCGCGATCCAGCAGGCTGGTTTGGAAAGTTGGAACAGAGTTCAATACTCAGACAAATGTATTGGACTAGGGGCCCCAACCATGAAGACCCATCTGAAACAATGATACCACACTCCCAGAGGCCCATACAATTGATGTCTTTGCTGGGTGAGGCTGCACTCCACGGCCCAGCATTCTACAGCAAAATCAGCAAACTGGTCATTGCAGAGTTGAAGGAAGGTGGCATGGATTTTTACGTGCCAAGACAAGAGCCAATGTTCAGATGGATGAGATTCTCGGATCTGAGCACGTGGGAGGGCGATCGCAATCTGGCTCCCAGTTTTGTGAATGAAGATGGCGTCGAA

>AB447448|II.12/II.4-03|2006|JP|Sakai2/2006

ATGAAGATGGCGTCTAACGACGCTTCCGCTGCCGCTGTTGCTAACAGCAACAACGACACCGCAAAATCTTCAAGTGACGGAGTGCTTTCTAGCATGGCTGTCACTTTTAAACGAGCCCTCGGGGCGCGGCACAAACAGCCTCCCCCGAGGGAAACACCACAAAGACCTCCACGACCACCCACCCCGGAACTTGCCAAAAAGATCCCTCCTCCCCCACCCAACGGGGAGGATGAACTAGTGGTTTCTTACAGCGCCAAAGATGGCGTTTCCGGTTTGCCTGAGCTTACCACTGTCCGGCAGCCGGATGAAACCAACACGGCGTTCAGTGTTCCCCCACTCAACCAGAGGGAGAATAGGGACGCCAAGGAGCCGCTAACCGGAACAATCCTGGAAATGTGGGACGGGGAGATCTATCATTACGGTCTGTATGTGGAACGAGGCCTTGTACTTGGTGTGCACAAACCACCAGCTGCCATCAGCCTCGCCAAGGTTGAGCTAGCACCACTCTCTCTGTTTTGGAGACCAGTGTATACACCACAGTATCTCATCTCTCCAGACACTCTCAGGAGACTGCATGGAGAGTCGTTCCCCTACACAGCCTTTGACAACAACTGCTATGCCTTCTGCTGTTGGGTCCTGGACCTAAACGACTCGTGGTTGTGCAGGAGGATGATCCAGAGGACAACTGGTTTCTTCAGACCTTACCAAGACTGGAACAGGAAACCCCTCCCCACCATGGATGACTCCAAACTGAAGAAGGTGGCCAACATATTCTTGTGTGCGCTATCTTCACTATTCACTAGGCCCATCAAAGACATAATAGGAAAGGTGAGACCGCTTAACATCCTTAACATCTTGGCCTCATGTGATTGGACTTTTGCAGGCATAGTGGAATCTTTGATCCTCTTGGCAGAGCTCTTTGGAGTTTTCTGGACACCCCCAGATGTGTCTGCGATGATCGCCCCCTTACTAGGTGACTACGAACTGCAGGGGCCCGAGGACCTTGCAGTGGAACTCGTTCCAGTAGTGATGGGGGGGATTGGTTTGGTGCTAGGATTCACCAAAGAGAAGATCGGAAAAATGTTGTCATCTGCTGCATCCACCTTAAGGGCTTGTAAAGACCTTGGTGCATACGGACTGGAAATCCTAAAATTGGTCATGAAGTGGTTCTTCCCAAAGAAAGAGGAAGCAAATGAGTTGGCTATGGTGAGGTCCATCGAGGATGCGGTGCTGGACCTCGAAGCAATTGAGAACAATCACATGACTGCCCTCCTCAAAGACAAAGACAGCTTGGCAACCTATATGAGAACCCTTGACCTCGAGGAGGAGAAGGCCAGGAAGCTTTCGACCAAGTCTGCTTCACCTGACATCGTGGGCACAATCAACGCTCTCCTGGCGAGAATCGCCGCTGCACGCTCCCTGGTGCATCGGGCGAAAGAGGAACTCTCCAGCAGACCAAGACCTGTTGTTGTTATGATATCAGGCAGGCCAGGGATAGGGAAAACTCACCTTGCTAGGGAACTGGCCAAGAAAATCGCAGCTTCGCTTACAGGGGACCAGCGTGTGGGTCTCATCCCTCGCAATGGCGTTGACCACTGGGATGCATATAAGGGGGAAAGAGTCGTCCTATGGGACGATTATGGAATGAGTAATCCCATCCACGACGCCCTCAGGTTACAGGAACTTGCTGACACCTGCCCCCTCACGCTAAACTGTGACAGGATTGAGAACAAAGGAAAGGTCTTTGACAGTGATGCCATAATCATCACCACTAATCTGGCCAACCCAGCACCATTGGACTATGTCAACTTTGAAGCGTGCTCGAGGCGCATCGACTTCCTCGTGTACGCAGAAGCCCCTGAGGTCGAGAAGGCAAAGCGTGATTTCCCAGGTCAACCTGACATGTGGAAGAACGCATTCAGTTCTGACTTCTCGCACATAAAACTGACGCTGGCTCCGCAAGGTGGCTTCGACAAGAACGGAAACACCCCACACGGGAAAGGCGTCATGAAAACCCTCACCACTGGTTCTCTCATCGCCCGAGCATCAGGGCTACTCCATGAGAGGTTAGATGAGTATGAGCTGCAG>GGCCCAACCCCTACCACCTTTAACTTTGACCGCAACAAGGTGCTTGCGTTCAGACAGCTTGCTGCCGAAAACAAGTACGGGTTGTTGGACACAATGAGAGTTGGAAAACAGCTCAAGGATGTCAAGACCATGCCAGAGCTCAAACAAGCGCTCAAAGATGTCGCAATCAAGAATTGTCAGATAGTGTATGGTGGCTGCACTTACAGGCTTGAGTCTGATGGCAAGGGTGATGTGAAAGTTGACAGAGTTCAGAGCGCCACTGTACAGACCAACAATGAGCTTGTCGGCGCCCTACATCATCTTAGGTGTGCCAGAATCAGATATTATGTCAAGTGTGTCCAGGAGGCCCTGTATTCCATCATCCAAATTGCTGGAGCTGCATTTGTAACCACGCGCATTGTCAAGCGCATGAACATACAAGACCTCTGGTCCAAGCCACAGGTGGAGGACACAGAGGAGACTGCTAGCAATGATGGGTGCCCAAAACCTAAGGATGACGACGAGTTCGTGGTCTCATCCGATGACATCAAAACCGAGGGCAAGAAAGGGAAGAACAAGTCTGGCCGTGGTAAGAAGCACACAGCATTCTCAAGCAAAGGTCTCAGTGATGAAGAGTACGATGAGTACAAAAGAATCAGAGAAGAAAGGAACGGCAAATACTCCATAGAGGAATACCTTCAGGACAGAGATAAGTATTATGAGGAGGTGGCCATCGCCAGGGCGACCGAAGAGGACTTCTGTGAAGAAGAAGAGGCCAAGATCCGACAAAGGATTTTCAGGCCAACAAGGAAACAACGCAAAGAGGAGAGGGCCTCTCTCGGTTTGGTCACAGGCTCTGAAATCAGGAAGAGGAACCCAGACGACTTCAAGCCTAAAGGAAAGCTGTGGGCTGATGATGACAGGAGTGTTGACTACAATGAGAGACTCAATTTTGAAGCCCCACCAAGCATCTGGTCGAGGATAGTCAATTTTGGTTCGGGTTGGGGCTTTTGGGTTTCCCCCAGCCTGTTCATAACATCAACTCATGTGATACCCCAGGGCGCACAGGAGTTCTTTGGTGTTCCCATCAAACAAATTCAGATACACAAATCGGGTGAATTCTGTCGTTTGAGGTTTCCAAAACCAATCAGAACTGACGTGACAGGCATGATCTTAGAAGAAGGTGCACCCGAAGGGACCGTGGTCACACTACTCATTAAGAGGCCAACTGGAGAACTCATGCCCTTGGCAGCCAGAATGGGAACCCATGCAACCATGAAGATTCAAGGGCGCACTGTTGGAGGTCAAATGGGCATGCTCCTAACAGGATCCAACGCCAAGAGTATGGACCTGGGCACCACACCAGGTGACTGTGGTTGTCCCTACATTTACAAGAGGGGGAATGACTATGTAGTCATTGGAGTCCACACAGCTGCCGCCCGTGGAGGAAACACTGTCATATGTGCCACCCAAGGGAGCGAGGGAGAAGCCACACTTGAAGGCGGTGACAGCAAGGGGACCTACTGCGGTGCACCAATCTTAGGTCCAGGAAGTGCCCCGAAGCTCAGCACCAAGACTAAATTCTGGAGATCATCCACGGCACCACTCCCACCTGGTACCTATGAGCCAGCCTACCTCGGCGGCAAGGACCCCAGAGTTAAGGGTGGCCCTTCATTGCAACAAGTTATGAGGGACCAGCTGAAACCATTCACTGAACCCAGGGGTAAACCACCAAAACCAAGTGTGTTAGAAGCTGCCAAGAAAACCATCGTCAATGTCCTTGAACAAACAATTGATCCACCTCAAAAATGGTCATTTGCGCAGGCATGCGCATCCCTTGACAAGACCACTTCCAGTGGTCACCCGCACCACATGCGGAAAAACGACTGCTGGAACGGGGAATCCTTTACAGGCAAATTGGCAGACCAGGCTTCCAAGGCCAACCTGATGTTCGAGGAGGGAAAGAACATGACTCCAGTCTACACGGGTGCGCTTAAGGACGAGCTGGTCAAGACTGACAAAATTTATGGCAAGATCAAAAAGAGGCTTCTCTGGGGTTCGGACCTGGCGACCATGATCCGGTGCGCTCGAGCATTTGGGGGCCTGATGGAGGAACTCAAAGCACATTGTGTCACACTACCTGTCAGAGTGGGTATGAATATGAATGAGGATGGTCCTATCATCTTTGAGAGACACTCTAGATATAAATATCATTATGATGCTGATTACTCCCGGTGGGACTCAACACAACAAAGAGCCGTGTTAGCAGCGGCTTTAGAAATCATGGTCAAGTTCTCCCCAGAGCCGCATCTGGCCCAAAAGGTTGCAGAAGACCTTCTTTCTCCCAGCGTGATGGACGTGGGTGATTTCAAAATATCAATCAATGAGGGTCTCCCCTCCGGGGTGCCCTGCACCTCCCAATGGAATTCCATCGCCCACTGGCTCCTCACTCTATGTGCACTCTCTGAGGTTACAAACCTGTCCCCTGACATTATCCAGGCCAACTCTCTCTTTTCTTTCTACGGTGATGATGAAATTGTGAGCACAGACATAAAATTGGACCCAGAAAAGCTGACAGCAAAACTCAAGGAATACGGGTTAAAACCGACCCGCCCTGACAAGACTGAGGGACCCCTTGTCATCTCTGAAGACCTGAATGGCCTAACCTTCCTGCGGAGAACCGTGACCCGCGACCCAGCAGGCTGGTTTGGAAAGTTGGAACAGAGTTCAATACTCAGACAAATGTATTGGACTAGAGGCCCCAACCATGAAGACCCATCTGAAACAATGATACCACACTCCCAGAGGCCCATACAATTGATGTCTTTGCTGGGTGAGGCTGCACTCCACGGCCCAGCATTCTACAGCAAAATCAGCAAACTGGTCATTGCAGAGTTGAAGGAAGGTGGCATGGATTTTTACGTGCCAAGACAAGAGCCAATGTTCAGATGGATGAGATTCTCGGATCTGAGCACGTGGGAGGGCGATCGCAATCTGGCTCCCAGTTTTGTGAATGAAGATGGCGTCGAA

>AF504671|II.12/II.10|2000|VN|Vintnam026

ATGAAGATGGCGTCTAACGACGCTTCCGCTGCCGCTGCTGCTAACAGCAACAACGACACCGCAAAATCTTCAAGTGACGGAATGCTTTCTAGCATGGCTGTCACTTTTAAACGAGCCCTCGGGGCACGGCCTAAACAGCCTCCCCCGAGGGAAATACCACAAAGACCCCCACGACCACCCACCCCAGAACTGATCAAAAAGATCCCTCCTCCCCCACCCAACGGGGAGGATGAACCAGTGGTTTCTTACAGCGTCAAAGATGGCGTTTCCGGCTTGCCTGAGCTTACTACTGTCAGGCAGCCGGGTGAAGCCAACACGGCGTTCAGTGTTCCCCCACTCAACCAAAGGGAAAATAGGGATGCCAAGGAGCCACTAACTGGAACAATCCTGGAAATGTGGGACGGGGAGATCTACCATTACGGCCTGTATGTGGAACGAGGTCTTGTACTTGGTGTGCACAAACCACCGGCTGCCATCAGCCTCGCCAAGGTTGAACTAACACCACTCTCTCTGTTTTGGAGACCAGTGTATACACCACAGTATCTCATCTCTCCGGACACTCTCAGGAGACTGCACGGGGAGTCGTTTCCCTACACAGCCTTTGACAACAACTGCTATGCCTTCTGTTGTTGGGTCCTGGACCTAAACGACTCGTGGTTGTGCAGGAGAATGATCCAGAGGACAACTGGTTTCTTCAGGCCCTACCAAGACTGGAATAGGAAACCCCTTCCCACCATGGATGACTCCAAGTTGAAGAAGGTAGCTAACATATTCTTGTGCGCGCTATCTTCGCTATTCACTAGGCCCATCAAAGACATAATAGGAAAGGTGAGGCCTCTCAACATCCTTAACATCTTGGCCTCGTGTGATTGGACTTTTGCAGGCATAGTGGAATCTTTGATTCTCTTGGCAGAGCTCTTTGGAGTTTTCTGGACACCCCCAGATGTGTCTGCGATGATCGCCCCTTTACTAGGTGACTACGAGCTGCAGGGACCCGAGGACCTTGCAGTGGAACTCGTTCCAATAGTGATGGGGGGGATTGGTTTGGTGCTAGGATTCACCAAAGAGAAGATCGGGAAAATGTTGTCATCTGCTGCATCCACCTTAAGAGCTTGTAAAGATCTTGGTGCATACGGACTGGAAATCTTAAAATTGGTCATGAAGTGGTTCTTCCCAAAGAAAGAGGAAGCAAATGAGCTGGCTATGGTGAGGTCCATCGAGGATGCGGTGCTGGACCTCGAGGCAATTGAGAACAACCACATGACTGCCCTCCTCAAAGACAAAGACAGCCTGGCAACCTACATGAGAACTCTCGACCTCGAGGAGGAGAAAGCCAGAAAGCTTTCAACCAAGTCTGCTTCACCTGATATCGTGGGCACAATCAACGCTCTCCTGGCGAGAATCGCCGCTGCACGCTCCCTGGTGCATCGGGCGAAAGAGGAGCTCTCCAGCAGACCAAGACCTGTTGTTGTGATGATATCAGGCAGGCCAGGGATAGGGAAAACCCACCTTGCCAGGGAATTGGCCAAGAGAATCGCTGCTTCTCTTACAGGGGACCAGCGTGTGGGTCTTATCCCGCGCAATGGTGTTGATCACTGGGACGCATATAAGGGAGAAAGAGTCGTTCTATGGGACGACTATGGAATGAGTAACCCCATCCACGACGCCCTCAGGTTACAAGAACTTGCTGACACCTGCCCCCTCACGCTAAATTGTGATAGGATTGAGAACAAAGGAAAGGTTTTTGACAGTGATGCCATAATCATCACCACTAACCTGGCCAACCCAGCACCACTGGACTATGTCAATTTTGAAGCATGCTCGAGGCGCATTGACTTCCTCGTGTATGCAGATGCTCCTGAGGTCGAGAAGGCAAAACGTGACTTTCCAGGTCAACCTGACATGTGGAAGAACGCTTTCAGTTCTGACTTCTCGCACATAAAACTGATGCTGGCCCCGCAGGGTGGCTTCGACAAGAACGGAAACACCCCACATGGGAAAGGCGTCATGAAAACCCTCACCACTGGTTCCCTCATCGCTCGAGCATCAGGGCTACTCCATGAGAGGTTAGATGAGTACGAGCTGCAG>GGCCCAACCCCCACCACCTTCAACTTTGACCGCAACAAGGTGCTTGCGTTCAGACAGCTTGCTGCTGAAAACAAGTACGGGTTGATGGACACAATGAGAGTCGGAAGACAGCTCAAAGATGTCAGGACCATGCCAGAACTCAAACAAGCACTCAAGAGTATCTCAATCAAGAGATGCCAGATAGTGTATAGTGGCTGCACCTATATGCTTGAGTCTGATGGCAAGGGTGAAGTGAAAGTTGACAGAGTTCAGAACGCCACTGTGCAGACCAACAATGAACTGGCCGGTGCCCTACACCATCTTCGGTGTGCCAGAATTAGATACTATGTCAAGTGCATTCAGGAGGCCCTGTATTCCATCATCCAAATTGCTGGAGCTGCATTTGTCACCACGCGCATTGTCAAGCGCATGAACATACAAGACCTTTGGTCCAAGCCACAGGTGGAAGATACAGAGGAGACTGTTAGCAAGGATGGGTGCCCAAAACCCAAGGATGATGACGAGTTCGTTGTTTCATCCGACGACATCAAAACCGAGGGCAAGAAAGGAAAGAACAAGTCTGGCCGTGGTAAGAAGCACACAGCATTCTCAAGCAAAGGTCTCAGTGATGAAGAGTACGATGAGTACAAAAGAATCAGAGAAGAAAGAAACGGCAAGTACTCTATAGAGGAGTACCTTCAGGACAGAGACAAGTATTATGAGGAGGTGGCCATCGCCAGGGCGACCGAAGAGGACTTCTGTGAAGAAGAAGAAGCCAAGATCCGACAAAGGATTTTTAGGCCAACAAGGAAACAACGCAAAGAGGAGAGGGCCTCTCTCGGTTTGGTCACAGGTTCTGAAATCAGGAAGAGGAACCCAGACGACTTCAAGCCTAAAGGAAAGCTGTGGGCTGATGACGACAGGAGTGTTGACTACAATGAGAGACTTAATTTTGAAGCCCCACCAAGCATTTGGTCGAGGATAGTCAACTTTGGTTCAGGTTGGGGTTTTTGGGTTTCCCCCAGCCTGTTCATAACATCAACTCACGTCATACCCCAGGGCGCACAGGAGTTCTTTGGGGTTTCCATCAAGCAAATTCAGATACACAAATCGGGTGAATTCTGTCGCTTGAGGTTTCCAAAACCAATCAGAACTGATGTGACAGGCATGATTCTAGAAGAAGGTGCGCCCGAAGGGACCGTAGTCACATTACTCATCAAGAGACCAACTGGAGAACTCATGCCCTTGGCAGCCAGAATGGGAACCCATGCAACCATGAAGATACAAGGGCGCACTGTTGGGGGTCAAATGGGCATGCTCCTAACAGGATCTAACGCCAAGAGTATGGACCTGGGCACCACACCAGGTGACTGTGGCTGTCCCTACATTTACAAGAGGGGGAATGACTACATAGTCATTGGAGTCCACACGGCTGCTGCCCGTGGAGGAAACACTGTCATATGTGCCACCCAGGGGAGCGAGGGAGAAGCCACACTTGAAGGCGGTGACAACAAAGGAACCTATTGCGGTGCACCAATCTTAGGTCCAGGGAGTGCCCCAAAGCTCAGCACCAAGACTAAGTTTTGGAGATCATCCACAGCACCACTCCCACCTGGTACCTATGAACCAGCCTACCTTGGCGGTAAGGACCCCAGAGTCAAGGGTGGCCCCTCATTGCAACAAGTCATGAGGGACCAGCTGAAACCATTCACTGAGCCCAGGGGTAAACCACCAAAACCAAGTGTGTTAGAGGCTGCCAAGAAAACCATCATCAATGTTCTCGAACAAACAATTGATCCACCTCAAAAATGGTCATTCGCGCAGGCATGCGCATCCCTCGACAAGACCACTTCCAGTGGCCACCCGCACCACATGCGGAAAAACGACTGCTGGAACGGGGAGTCCTTCACAGGCAAATTGGCAGACCAGGCTTCCAAGGCTAACCTGATGTTCGAAGAGGGAAAGAACATGACTCCAGTCTACACAGGTGCGCTTAAGGACGAGCTGGTCAAGACTGACAAAATTTATGGCAAGATCAAAAAGAGGCTTCTCTGGGGCTCGGATCTGGCGACCATGATCCGGTGTGCTCGAGCGTTTGGAGGCCTGATGGAGGAACTCAAAGCACATTGTGTCACACTACCCGTCAGAGTAGGTATGAATATGAATGAGGATGGCCCTATCATCTTTGAGAGACACTCCAGATATAAGTATCATTATGATGCTGATTACTCCCGGTGGGACTCAACACAACAAAGAGCCGTGTTAGCAGCAGCCTTAGAAATCATGGTTAAGTTCTCCCCAGAACCGAATCTGGCCCAAAAGGTTGCAGAAGACCTTCTCTCTCCCAGCGTGATGGACGTTGGTGACTTCAAAATATCAATCAATGAGGGCCTCCCCTCCGGGGTGCCCTGCACCTCCCAATGGAATTCTATCGCCCACTGGCTCCTCACCCTCTGTGCGCTTTCTGAGGTTACAAACCTGTCCCCTGACATTATCCAGGCTAATTCCCTCTTTTCCTTCTACGGTGATGATGAAATTGTGAGCACAGACATAAAATTGGACCCAGAGAAGCTGACAGCAAAACTTAAGGAATACGGGTTGAAACCGACCCGCCCTGATAAGACTGAGGGACCCCTTGTTATCTCTGAGGACCTGGATGGCCTAACCTTCCTGCGGAGGACTGTGACCCGCGACCCAGCTGGCTGGTTTGGAAAGCTGGAACAGAGTTCAATACTTAGGCAAATGTATTGGAGTAGGGGCCCCAACCATGAAGACCCATCTGAAACAATGATACCACACTCCCAAAGACCCATACAATTGATGTCTTTGCTGGGCGAGGCTGCACTCCACGGCCCAGCATTCTACAGCAAAATCAGCAAGCTGGTCATTGCAGAGCTGAAGGAAGGTGGCATGGATTTTTACGTGCCCAGACAAGAGCCTATGTTCAGATGGATGAGGTTTTCAGATCTGAGCACGTGGGAGGGCGATCGCAATCTGGCTCCCAGTTTTGTGAATGAAGATGGCGTCGAA

>AY237415|II.12/II.10|2000|TH|Mc37

ATGAAGATGGCGTCTAACGACGCTTCCGCTGCCGCTGCTGCTAATAGCAACAACGACACCGCAAAATCTTCAAGTGACGGAATGCTTTCTAGCATGGCTGTCACTTTTAAACGAGCCCTCGGGGCACGGCCTAAACAGCCTCCCCCGAGGGAAATACCACAAAGACCCCCACGACCACCCACCCCAGAACTGGTCAAAAAGATCCCTCCTCCCCCACACAACGGGGAGGATGAACCAGTGGTTACTTACAGCGTCAAAGATGGCGTTTCCGGCTTGCCTGAGCTCACTACTGTCAGGCAGCCGGGTGAAGCCAACACGGCGTTCAGTGTTCCCCCACTCAACCAAAGGGAGAATAGGGATGCCAAGGAGCCACTAACTGGAACAATCCTGGAAATGTGGGACGGGGAGATCTACCATTACGGCCTGTATGTGGAACGAGGTCTTGTACTTGGTGTGCACAAACCACCGGCTGCCATCAGCCTCGCCAAGGTTGAACTAACACCACTCTCTCTGTTTTGGAGACCAGTGTATACACCACAGTATCTCATCTCCCCGGACACTCTCAGGAAACTGCACGGAGAGTCGTTTCCCTACACAGCCTTTGATAACAACTGCTATGCCTTCTGTTGTTGGGTCCTGGACTTAAATGACTCGTGGTTGTGCAGGAGAATGATCCAGAGGACAACTGGTTTCTTCAGGCCCTACCAAGACTGGAATAGGAAACCCCTTCCCACCATGGATGACTCCAAGTTGAAGAAGGTAGCTAACATATTCTTGTGCGCGCTATCTTCGCTATTCACTAGGCCCATCAAAGACATAATAGGAAAGGTGAGGCCTCTCAACATCCTTAACATCTTGGCCTCATGTGATTGGTCTTTCGCAGGCATAGTGGAATCTTTGATTCTCTTGGCAGAGCTCTTTGGAGTTTTCTGGACACCCCCAGATGTGTCTGCGATGATCGCCCCTTTACTAGGTGACTACGAGCTGCAGGGACCCGAGGACCTTGCAGTGGAACTCGTTCCAGTAGTAATGGGGGGGATTGGTTTGGTGCTAGGATTCACCAAAGAGAAGATCGGGAAAATGTTGTCATCTGCTGCATCCACCTTAAGAGCTTGTAAAGATCTTGGTGCATACGGACTGGAAATCTTAAAATTGGTCATGAAGTGGTTCTTCCCAAAGAAAGAGGAAGCAAATGAGCTGGCTATGGTGAGGTCCATCGAGGATGCGGTGCTGGACCTCGAGGCAATTGAGAACAACCACATGACTGCCCTCCTCAAAGACAAAGACAGCCTGGCAACCTACATGAGAACTCTTGACCTCGAGGAGGAGAAAGCCAGAAAGCTTTCAACCAAGTCTGCTTCACCTGATATCGTGGGCACAATCAACGCTCTCCTGGCGAGAATCGCCGCTGCACGCTCCCTGGTGCATCGGGCGAAAGAGGAGCTCTCCAGCAGACCAAGACCTGTTGTTGTGATGATATCAGGCAGGCCAGGTATAGGGAAAACCCACCTTGCCAGGGAATTGGCCAAGAGAATCGCTGCTTCTCTCACAGGGGACCAGCGTGTGGGTCTCATCCCGCGCAATGGTGTTGATCACTGGGACGCATATAAGGGAGAAAGAGTCGTTCTATGGGACGACTATGGAATGAGTAACCCCATCCACGACGCCCTCAGGTTACAAGAACTTGCTGACACCTGCCCCCTCACGCTAAATTGTGATAGGATTGAGAACAAAGGAAAGGTCTTTGACAGTGATGCCATAATCATCACCACTAACCTGGCCAACCCAGCACCACTGGACTATGTCAATTTTGAAGCGTGCTCGAGGCGCATCGACTTCCTCGTGTATGCAGATGCCCCTGAGGTCGAGAAGGCAAAACGTGACTTTCCAGGTCAACCTGACATGTGGAAGAACGCTTTCAGTTCTGACTTCTCGCACATAAAACTGACGCTGGCTCCGCAGGGTGGCTTCGACAAGAACGGAAACACCCCACATGGGAAAGGCGTCATGAAAACTCTCACTACTGGTTCCCTCATCGCTCGAGCATCAGGGCTACTCCATGAGAGGTTAGATGAGTACGAGCTGCAG>GGCCCAACCCCCACCACCTTCAACTTTGACCGCAACAAGGTGCTTGCGTTCAGACAGCTTGCTGCTGAAAACAAGTACGGGTTGATGGACACAATGAGAGTCGGAAGACAGCTCAAAGATGTCAGGACCATGCCAGAACTCAAACAAGCACTCAAGAGTATCTCAATCAAGAGATGCCAGATAGTGTATAGTGGCTGCACCTATATGCTTGAGTCTGATGGCAAGGGTGAAGTGAAAGTTGACAGAGTTCAGAACGCCACTGTGCAGACCAACAATGAACTGGCCGGTGCCCTACACCATCTTCGGTGTGCCAGAATTAGATACTATGTCAAGTGCATTCAGGAGGCCCTGTATTCCATCATCCAAATTGCTGGAGCTGCATTTGTTACCACGCGCATTGTCAAGCGCATGAACATACAAGACCTTTGGTCCAAGCCACAGGTGGAAGATACAGAGGAGACTGCTAGCAAGGATGGGTGCCCAAAACCCAAGGATGATGACGAGTTCGTTGTTTCATCCGATGACATCAAAACTGAGGGCAAGAAAGGAAAGAACAAATCTGGCCGTGGTAAGAAGCACACAGCATTCTCAAGCAAAGGTCTCAGTGATGAAGAGTACGATGAGTACAAAAGAATCAGAGAAGAAAGAAACGGCAAGTACTCTATAGAGGAGTACCTTCAGGACAGAGACAAGTATTATGAGGAGGTGGCCATCGCCAGGGCGACCGAAGAGGACTTCTGTGAAGAAGAAGAGGCCAAGATCCGGCAAAGGATTTTTAGGCCAACAAGGAAACAACGCAAAGAGGAGAGGGCCTCTCTCGGTTTGGTCACAGGTTCTGAAATCAGGAAGAGAAACCCAGACGACTTCAAGCCTAAAGGAAAGCTGTGGGCTGATGACGACAGGAGTGTTGACTACAATGAGAAACTTAGTTTTGAAGCCCCACCAAGCATCTGGTCGAGGATAGTCAACTTTGGTTCAGGTTGGGGTTTTTGGGTTTCCCCCAGCCTGTTCATAACATCAACTCATGTCATACCCCAGGGCGCACAGGAGTTCTTCGGGGTTCCCATCAAGCAAATTCAGATACACAAATCGGGTGAATTTTGTCGCTTGAGGTTTCCAAAACCAATCAGAACTGATGTGACAGGCATGATCCTTGAAGAAGGTGCGCCCGAAGGGACCGTGGTCACATTACTCATCAAGAGACCAACTGGAGAACTCATGCCCTTGGCAGCCAGAATGGGGACCCATGCAACCATGAAGATACAAGGGCGCACTGTTGGGGGTCAAATGGGCATGCTCCTAACAGGATCTAACGCCAAGAGTATGGACCTGGGCACCACACCAGGTGACTGTGGCTGTCCCTACATTTACAAGAGGGGGAATGACTACATAGTCATTGGAGTCCACACGGCTGCTGCCCGTGGAGGAAACACTGTCATATGTGCCACCCAGGGGAGTGAGGGAGAAGCCACACTTGAAGGCGGTGACAACAAGGGAACCTATTGCGGTGCACCCATCTTAGGTCCAGGGAGTGCCCCAAAGCTCAGCACCAAGACTAAGTTTTGGAGGTCATCCACAGCACCACTCCCACCTGGCACCTACGAACCAGCCTACCTTGGCGGCAAGGACCCCAGAGTCAAGGGTGGCCCCTCATTGCAACAAGTCATGAGGGACCAGCTGAAACCATTCACTGAGCCCAGGGGTAAACCACCAAAACCAAGTGTGCTAGAGGCTGCCAAGAAAACCATCATCAATGTTCTTGAACAAACAATTGATCCACCTCAAAAATGGTCATTCGCGCAGGCATGCGCATCCCTCGACAAGACCACTTCCAGTGGCCACCCGCACCACATGCGGAAAAACGACTGCTGGAACGGGGAGTCCTTCACAGGCAAATTGGCAGACCAGGCTTCCAAGGCTAACCTGATGTTTGAGGAGGGAAAGAACATGACCCCAGTCTACACAGGTGCGCTTAAGGACGAGCTGGTCAAGACTGACAAAATTTATGGCAAGATCAAAAAGAGGCTCCTCTGGGGCTCGGATCTGGCGACCATGATCCGGTGTGCCCGAGCGTTTGGAGGCCTGATGGAGGAACTCAAAGCACATTGTGTCACACTACCCGTCAGAGTAGGTATGAATATGAATGAGGATGGCCCTATCATCTTTGAGAGACACTCCAGATATAAATATCATTATGATGCTGATTACTCCCGGTGGGACTCAACACAACAAAGAGCCGTGTTAGCAGCAGCCCTAGAAATCATGGTCAAGTTCTCCCCAGAACCGAATCTGGCCCAAAAGGTTGCAGAAGACCTTCTCTCTCCCAGCGTGATGGACGTTGGCGACTTCAAAATATCAATCAATGAGGGCCTCCCCTCCGGGGTGCCCTGCACCTCTCAATGGAATTCTATCGCCCACTGGCTCCTCACCCTCTGTGCGCTTTCTGAGGTTACAAACCTGTCCCCTGACATTATCCAGGCTAATTCCCTCTTTTCCTTCTACGGTGATGATGAAATTGTGAGCACAGACATAAAACTGGACCCAGAGAAGCTGACAGCAAAACTTAAGGAATACGGGTTGAAACCGACTCGCCCTGACAAGACTGAGGGACCCCTTGTTATCTCTGAGGACCTGGATGGCCTAACCTTCCTGCGGAGGACTGTGACCCGCGACCCAGCTGGCTGGTTTGGAAAGCTGGAACAGAGTTCAATACTTAGGCAAATGTATTGGACTAGGGGCCCCAACCATGAAGACCCATCTGAAACAATGATACCACACTCCCAAAGACCCATACAATTGATGTCTTTGCTGGGCGAGGCTGCACTCCACGGCCCAGCATTCTACAGCAAAATCAGCAAGCTGGTCATTGCAGAGCTGAAGGAAGGTGGCATGGATTTTTACGTGCCCAGACAAGAGCCTATGTTCAGATGGATGAGGTTTTCAGATCTGAGCACGTGGGAGGGCGATCGCAATCTGGCTCCCAGTTTTGTGAATGAAGATGGCGTCGAA

>DQ369797|II.12/II.4-03|2004|CN|NVgz01

ATGAAGATGGCGTCTAACGACGCTTCCGCTGTCGCTGCTGCTAACAGCAACAACGACACCGCAAAATCTTCAAGTGACGGAGTGCTTTCTAGCATGGCTGTCACTTTTAAACGAGCCCTCGGGGCGCGGCATAAACAGCCTCCCCCGAGGGAAATACCACAAAGACCTCCACGACCACCCACCCCGGAACTTGCCAAAAAGATCCCTCCTCCCCCACCCAACGGGGAGGATGAACTAGTGGTTTCTTACAGCGCCAAAGATGGCGTTTCCGGTTTGCCTGAGCTTACCACTGTCTGGCAGCCGGATGAAACCAACACGGCGTTCAGTGTTCCCCCACTCAACCAGAGGGAGAATAGGGACGCCAAGGAGCCGCTAACCGGAACAACCCTGGAAATGTGGGACGGGGAGATCTATCATTACGGTCTGTATGTGGAGCGAGGCCTTGTACTTGGTGTGCACAAACCACCAGCTGCCATCAGCCTCGCCAAGGTTGAGCTAGCACCACTCTCTCTGTTTTGGAGACCAGTGTATACACCACAGTATCTCATCTCTCCAGACACTCTCAGGAGACTGCATGGAGAGTCGTTCCCCTACACAGCCTTTGACAACAACTGCTATGCCTTCTGTTGTTGGGTCCTGGACCTAAACGACTCGTGGTTGTGCAGGAGAATGATCCAGAGGACAACTGGTTTCTTCAGACCTTACCAAGACTGGAACAGGAAACCCCTCCCCACCATGGATGACTCCAAATTGAAGAAGGTAGCCAACATATTCTTGTGTGCGCTATCTTCGCTTATCACTAGGCCCATCAAAGACATAATAGGAAAGGTGAGGCCGCTTAACATCCTTAACATCTTGGCCTCATGTGATTGGACTTTTGCAGGCATAGTGGAATCTTTGATCCTCTTGGCAGAGCTCTTTGGAGTTTTCTGGACACCCCCAGATGTGTCTGCGATGATCGCCCCCTTACTAGGTGACTACGAACTGCAGGGGCCCGAGGACCTTGCAGTGGAACTCGTTCCAGTAGTGATGGGGGGGATTGGTTTGGTGCTAGGATTCACTAAAGAGAAGATCGGAAAAATGTTGTCATCTGCTGCATCCACCTTAAGGGCTTGTAAAGACCTTGGTGCATACGGACTGGAAATCCTAAAATTGGTCATGAAGTGGTTCTTCCCAAAGAAAGAGGAAGCAAATGAGTTGGCTATGGTGAGGTCCATCGAGGATGCGGTGCTGGACCTCGAAGCAATTGAGAACAATCACATGACTGCCCTCCTCAAAGACAAAGACAGCTTGGCAACCTACATGAGAACCCTTGACCTCGAGGAGGAGAAGGCCAGGAAGCTTTCAACCAAGTCTGCTTCACCTGACATCGTGGGCACAATCAACGCTCTCCTGGCGAGAATCGCCGCTGCACGCTCCCTGGTGCATCGGGCGAAAGAGGAACTCTCCAGCAGACCAAGACCTGTTGTTGTTATGATATCAGGTAGGCCAGGGATAGGGAAAACTCACCTTGCTAGGGAACTGGCCAAGAAAATCGCAGCTTCGCTTACAGGAGACCAGCGTGTGGGTCTCACCCCTCGCAATGGCGTTGGTCACTGGGATGCATATAAGGGGGAAAGAGTCGTCCTATGGGACGATTATGGAATGAGTAATCCCATCCACGACGCCCTCAGATTACAGGAACTTGCTGACACCTGCCCCCTCACACTAAACTGTGACAGGATTGAGAACAAAGGAAAGGTCTTTGACAGTGATGCCATAATCATCACCACTAATCTGGCCAACCCAGCACCATTGGACTATGTCAACTTTGAAGCATGCTCGAGGCGCATCGACTTCCTCGTGTACGCAGAAGCCCCTGAGGTCGAGAAGGCAAAGCGTGATTTCCCAGGTCAACCTGACATGTGGAAGAACGCATTCAGTTCTGACTTCTCGCACATAAAACTGACGCTGGCTCCGCAAGGTGGCTTCGACAAGAACGGAAACACCCCACACGGGAAAGGCGTCATGAAAACCCTCACCACTGGTTCCCTCATCGCCCGAGCACCAGGGTTACTCCATGAGAGGTTAGATGAGTATGAGCTGCAG>GGCCCAACCCCTACCACCTTTAACTTTGACCGCAACAAGGTGCTTGCGTTCAGACAGCTTGCTGCCGAAAACAAGTACGGGTTGTTGGACACAATGAGAGTTGGAAAACAGCTCAAGGATGTCAAGACCATGCCAGAGCTCAAACAAGCGCTCAAAAATGTTGCAATCAAAAATTGTCAGATAGTGTATGGTGGCTGCACTTACAGGCTTGAGTCTGATGGCAAGGGTGATGTAAAAGTTGACAGAGTCCAGAACGCCACTGTACAGACCAACAATGAGCTTGCCGGCGCCCTACATCATCTTAGGTGTGCCAGAATCAGATATTATGTCAAGTGTGTCCAGGAGGCCCTGTATTCCATCATCCAAATTGCTGGAGCAGCATTTGTAACCACGCGCATTGTCAAGCGCATGAACATACAAGACCCCTGGTCCAAGCCACAGGTGGAGGACACAGAGGAGACCGCCAGCAATGACGGGTGCCCAAAACCCAAGGATGATGACGAGTTCGTTGTCTCATCCGATGACATCAAAACCGAGGGCAAGAAAGGGAAGAACAAGTCTGGCTGTGGCAAGAAGCACACAGCCTTCTCAAGCAAAGGTCTCAGTGATGAAGAGTACGATGAGTACAAAAGAATCAGAGAAGAAAGGAACGGCAAATACTCCATAGAGGAATACCTTCAGGACAGAGACAAGTATTATGAGGAGGTGGCCATCGCCAGGGCGACCGAAGAGGACTTCTGTGAAGAAGAAGAGGCCAAGATCCGACAAAGGATTTTCAGGCCAACAAGGAAACAACGCAAAGAGGAGAGGGCCTCTCTCGGTTTGGTCACAGGCTCTGAAATCAGGAAGAGGAACCCAGACGACTTCAAGCCTAAAGGAAAGCTGTGGGCTGATGATGACAGGAGTGTTGACTACAATGAGAGACTCAATTTTGAAGCCCCACCAAGCATCTGGTCGAGGATAGTCAATTTTGGTTCGGGTTGGGGCTTTTGGGTCTCCCCCAGCCTGTTCATAACATCAACTCATGTCATACCCCAGGGTGCACAGGAGTTCTTTGGTGTTCCCATCAAACAAATTCAGATACACAAATCGGGTGAATTCTGTCGTTTGAGGTTTCCAAAACCAATCAGAACTGACGTAACAGGCATGATCTTAGAAGAAGGTGCACCCGAAGGGACCGTGGTCACACTACTCATTAAAGGGCCAACTGGAGAACTCATGCCCTTGGCAGCCAGAATGGGAACCCATGCAACCATGAAGATTCAAGGGCGCACTGTTGGAGGTCAAATGGGCATGCTCCTAACAGGATCCAACGCCAAGAGTATGGACCTGGGCACCACACCAGGTGACTGTGGCCGTCCCTACATTTACAAGAGGGGGAATGACTATGTGGTCATTGGAGTCCACACAGCTGCCGCCCGTGGAGGAAACACTGTCATATGTGCCACCCAAGGGAGCGAGGGAGAAGCCACACTTGAAGGCGGTGACAGCAAGGGGACCTACTGCGGTGCACCAATCTTAGGTCCAGGGAGTGCCCCAAAGCTCAGCACCAAGACTAAATTCTGGAGATCATCCACGGCACCACTCCCACCTGGTACCTATGAGCCAGCCTACCTCGGCGGCAAGGACCCTAGAGTTAAGGGTGGCCCTTCATTGCAACAAGTTATGAGGGACCAGCTGAAACCATTCACTGAACCCAGGGGTAAACCACCAAAGCCAAGTGTGTTGGAAGCTGCCAAGAAAACCATCGTCAATGTCCTTGAACAAACAATTGATCCACCTCAAAAATGGTCATTTGCGCAGGCATGCGCATCCCTTGACAAGACCACTTCCAGTGGTCACCCGCACCACATGCGGAAAAACGACTGCTGGAACGGAGAATCCTTTACAGGCAAATTGGCAGACCAGGCTTCCAAGGCCAACCTGATGTTCGAGGAGGGAAAGAACATGACCCCAGTCTACACGGGTGCGCTTAAGGACGAGCTGGTCAAGACTGACAAAATTTATGGCAAGATCAAAAAGAGGCTTCTCTGGGGTTCGGACCTGGCGACCATGATCCGGTGCGCTCGAGCATTCGGGGGCCTGATGGAGGAACTCAAAGCACATTGTGTCACACTACCTGTCAGAGTGGGTATGAATATGAATGAGGATGGTCCTATCATCTTTGAGAGACACTCCAGATATAAATATCATTATGATGCTGATTACTCCCGGTGGGACTCAACACAACAAAGAGCCGTGTTAGCAGCGGCTTTAGAAATCATGGTCAAGTTCTCCCCAGAGCCGCATCTGGCCCAAAAAGTTGCAGAAGACCTTCTTTCTCCCAGCGTGATGGACGTGGGTGATTTCAAAATATCAATCAATGAGGGCCTCCCCTCCGGGGTGCCCTGCACCTCCCAATGGAATTCCATCGCCCACTGGCTCCTCACTCTATGTGCACCCTCTGAGGTTACAAACCTGTCCCCTGACATTATCCAGGCCAACTCTCTCTTTTCTTTCTACGGTGATGATGAAATTGTGAGCACAGACATAAAATTGGACCCAGAAAAGTTGACAGCAAAACTCAAGGAATACGGGTTGAAACCGACCCGCCCTGACAAGACTGAGGGACCCCTTGTTATCTCTGAAGACCTGAATGGCCTAACCTTCCTGCGGAGGACCGTGACCCGCGACCCAGCAGGCTGGTTTGGAAAGTTGGAACAGAGTTCAATACTCAGACAAATGTATTGGACTAGGGGCCCCAACCATGAAGACCCATCTGAAACAATGATACCACACTCCCAGAGGCCCATACAATTGATGTCTTTGCTGGGTGAGGCTGCACTCCACGGCCCAGCATTCTACAGCAAAATCAGCAAACTGGTCATTGCAGAGTTGAGGGAAGGTGGCATGGATTTTTACGTGCCAAGACAAGAGCCAATGTTCAGATGGATGAGATTCTCGGATCTGAGCACGTGGGAGGGCGATCGCAATCTGGCTCCCAGTTTTGTGAATGAAGATGGCGCCGAA

>GU980585|II.12/II.3|2006|KR|CBNU1

ATGAAGATGGCGTCTAACGACGCTTCCGCTGCCGCTGCTGCTGACAGCAACAACGACACCGCAAAATCTTCAAGTGACGGAGTGCTTTCTAGCATGGCTGTCACTTTTAAACGAGCCCTCGGGGCGCGGCATAAACAGCCTCCCCCGAGGGAAATACCACAAAGGCCTCCACGACCACCCACCCCAGAACTGGTCAAAAAGATCCCTCCTCCCCCACCCAACGGGGAGGATGAACTAGTGGTTTCTTACAGCGCCAAAGATGGCGTTTCTGGTTTGCCTGAGCTTACCACTGTCTGGCAGCCGGATGAAACCAATACGGCGTTCAGTGTTCCCCCACTCAACCAGAGGGAGAACAGGGACGCCAAGGAGCCGCTAACCGGAACAATCCTGGAAATGTGGGACGGGGAGATCTATCATTACGGTCTGTATGTGGAGCGAGGTCTTGTACTTGGTGTGCACAAACCACCAGCTGCCATCAGCCTCGCCAAGGTTGAACTAGCACCACTCTCTCTGTTTTGGAGGCCAGTGTACACACCACAGTATCTCATCTCTCCAGACACTCTCAGGAGACTGCATGGAGAGTCGTTCCCCTACACAGCCTTTGACAACAACTGCTATGCCTTCTGTTGTTGGGTTCTGGACCTAAACGACTCGTGGTTGTGCAGGAGAATGATCCAGAGGACAACTGGTTTCTTCAGACCTTACCAAGACTGGAACAGGAAACCCCTCCCCACCATGGATGACTCCAAATTGAAGAAGGTAGCCAACATATTCTTGTGTGCGCTATCTTCGCTATTCACTAGGCCCATCAAAGACATAATAGGAAAGGTGAGACCGCTTAACATCCTTAACATCTTGGCCTCATGTGATTGGACTTTTGCAGGCATAGTGGAATCTTTGATCCTCTTGGCAGAGCTCTTTGGAGTTTTCTGGACACCCCCAGATGTGTCTGCGATGATCGCCCCCTTACTAGGTGACTACGAACTGCAGGGGCCTGAGGACCTTGCAGTGGAACTCGTACCAGTAGTGATGGGGGGGATTGGTTTGGTGCTAGGATTCACCAAAGAGAAGATCGGAAAAATGTTGTCATCTGCTGCATCCACCTTAAGAGCTTGTAAAGACCTTGGTGCATACGGACTGGAAATCCTAAAATTGGTCATGAAGTGGTTCTTCCCAAAGAAAGAGGAAGCAAATGAGCTGGCTATGGTGAGGTCCATCGAGGATGCGGTGCTGGACCTCGAAGCAATTGAGAACAACCACATGACTGCCCTCCTCAAAGACAAAGACAGCTTGGCAACCTACATGAGAACTCTTGACCTCGAGGAGGAGAAGGCCAGGAAGCTTTCAACCAAGTCTGCTTCACCTGACATCGTGGGCACAATCAACGCTCTCCTGGCGAGAATCGCCGCTGCACGTTCCCTGGTGCATCGGGCGAAAGAGGAACTCTCTAGCAGACCAAGACCTGTTGTTGTTATGATATCAGGCAGGCCAGGGATAGGGAAAACTCACCTTGCCAGGGAGCTAGCCAAGAAAATCGCAGCTTCGCTTACAGGGGACCAGCGTGTGGGTCTCATCCCTCGCAATGGCGTTGATCACTGGGATGCATATAAGGGGGAAAGAGTCGTCCTATGGGACGATTATGGAATGAGTAATCCCATCCACGACGCCCTCAGGTTACAGGAACTTGCTGACACCTGCCCCCTTACGCTAAACTGTGACAGGATTGAGAACAAAGGAAAGGTCTTTGACAGTGATGCCATAATCATCACCACTAATCTGGCCAACCCAGCACCATTGGACTATGTCAACTTTGAAGCATGCTCGAGGCGCATCGACTTCCTCGTGTACGCAGAAGCCCCTGAGGTCGAGAAGGCAAAGCGTGATTTCCCAGGTCAACCTGACATGTGGAAGAACGCATTTAGTTCTGACTTCTCGCACATAAAACTGGCGCTGGCTCCGCAAGGTGGCTTCGACAAGAACGGAAACACCCCACACGGGAAAGGCGTTATGAAAACCCTCACCACTGGTTCCCTCATCGCCCGAGCATCAGGGTTACTCCATGAGAGGTTAGATGAGTATGAGCTGCAG>GGCCCAACCCCCACCACCTTTAACTTTGACCGCAACAAGGTGCTTGCGTTCAGACAGCTTGCTGCCGAAAACAAGTACGGATTGTTGGACACAATGAGAGTTGGAAAACAGCTCAAGGATGTCAAGACCATGCCAGAGCTCAAACAAGCACTCAAGAATGTCGCAATCAAGAGTTGTCAGATAGTGTATGGTGGCTGCACTTATAGGCTTGAGTCTGACGGCAAGGGTGATGTGAAAGTTGACAGAGTTCAGAACGCCACTGTACAGACTAACAATGAACTTGCCGGCGCCCTACATCATCTTAGGTGTGCCAGAATCAGATATTATGTCAAGTGTGTCCAGGAGGCCCTGTATTCCATCATCCAAATTGCTGGAGCTGCATTTGTAACCACGCGCATTGTCAAGCGCATGAACATACAAGATCTCTGGTCCAAGCCACAGGTGGAGGATACAGAGGAGACCGCTAGCAAGGATGGGTGCCCAAAACCCAAGGATGATGACGAGTTCGTTGTCTCATCCGATGACATCAAAACCGAGGGCAAGAAAGGGAAGAACAAGTCTGGCCGTGGTAAGAAGCACACAGCATTCTCAAGTAAAGGTCTCAGTGATGAAGAGTACGATGAGTACAAAAGAATCAGAGAAGAAAGAAATGGCAAATACTCCATAGAGGAATACCTCCAGGACAGGGACAAGTATTATGAGGAGGTGGCCATCGCCAGGGCGACCGAAGAGGACTTCTGTGAAGAAGAAGAGGCCAAGATCCGACAAAGGATTTTCAGGCCAACAAGGAAACAACGCAAAGAGGAGAGGGCCTCTCTCGGTTTGGTCACAGGCTCTGAGATCAGGAAGAGGAACCCAGACGACTTCAAGCCTAAAGGAAAGCTGTGGGCTGATGACGACAGGAGTGTTGACTACAATGAGAGACTCAATTTTGAAGCCCCACCAAGCATCTGGTCGAGGATAGTCAGCTTTGGTTCAGGTTGGGGCTTTTGGGTTTCCCCCAGCCTGTTCATAACATCAACCCATGTCATACCCCAGGGCGCACAGGAGTTCTTTGGTGTTCCCATCAAACAAATTCAGATACACAAATCGGGTGAATTCTGTCGTTTGAGGTTTCCAAAGCCGATCAGAACTGACGTGACAGGCATGATCTTAGAAGAAGGTGCACCCGAAGGGACCGTGGTCACACTACTCATCAAGAGGCCAACTGGAGAACTCATGCCCCTGGCAGCCAGAATGGGAACCCATGCAACCATGAGGATTCAAGGGCGCACTGTTGGAGGTCAAATGGGCATGCTTCTAACAGGATCCAACGCTAAGAGTATGGACCTGGGCACCACACCAGGTGACTGTGGTTGTCCCTACATTTACAAGAGGGGGAATGACTACGTAGTCATTGGAGTCCACACAGCTGCCGCCCGTGGAGGAAACACCGTCATTTGTGCCACCCAGGGGAGCGAGGGAGAAGCCACACTTGAAGGCGGTGACAGCAAGGGGACCTACTGCGGTGCACCAATCTTAGGTCCAGGAAGTGCCCCAAAGCTCAGCACCAAGACTAAATTCTGGAGGTCATCCACGGCACCACTCCCACCTGGTACCTATGAGCCAGCCTACCTCGGCGGCAAGGACCCCAGAGTTAAGGGTGGCCCCTCATTGCAACAAGTTATGAGGGACCAACTGAAACCATTCACTGAGCCCAGGGGTAAACCACCAAAACCAAGTGTGTTAGAAGCTGCCAAGAAAACCATCGTCAATGTCCTTGAACAAACAATTGATCCACCTCAAAAATGGTCGTTTGCGCAGGCATGCGCATCCCTTGACAAGACCACTTCCAGCGGTCACCCGCACCATATGCGGAAAAACGACTGCTGGAACGGGGAATCCTTTACAGGAAAATTGGCAGACCAGGCTTCCAAGGCCAACCTGATGTTCGAGGAGGGAAAGAACATGACCCCAGTCTACACGGGTGCGCTCAAGGACGAGCTGGTCAAGACTGACAAGATTTATGGCAAGATCAAAAAGAGGCTTCTCTGGGGTTCGGACCTGGCGACCATGATTCGGTGCGCTCGAGCATTCGGAGGCCTGATGGAGGAACTCAAAGCACATTGCGTCACACTACCTGTCAGAGTGGGCATGAATATGAATGAGGATGGTCCTATCATCTTTGAGAGACACTCCAGATATAAATACCATTATGATGCTGATTACTCCCGGTGGGACTCGACACAACAAAGAGCCGTGTTAGCAGCAGCCTTAGAAATCATGGTCAAGTTCTCCCCAGAGCCGCATCTGGCCCAAAAGGTTGCAGAAGACCTTCTTTCTCCCAGCGTGATGGACGTGGGTGATTTCAAAATATCAATTAATGAGGGTCTCCCCTCCGGGGTGCCCTGCACCTCCCAATGGAATTCCATCGCCCACTGGCTCCTCACTCTCTGTGCACTCTCTGAGGTTACAAACCTGTCCCCTGACATTATTCAGGCTAACTCTCTCTTTTCTTTCTACGGTGATGATGAAATTGTGAGTACAGACATAAAATTGGACCCAGAAAAACTGACAGCAAAACTCAAGGAATACGGGTTGAAACCGACCCGCCCTGACAAGACTGAAGGACCCCTTGTCATCTCTGAAGACCTGAATGGCCTAACCTTCCTGCGGAGGACCGTGACCCGCGACCCAGCAGGCTGGTTTGGAAAGTTGGAACAGAGTTCAATACTCAGACAAATGTATTGGACTAGGGGCCCCAACCATGAAGACCCATCTGAAACAATGATACCACACTCCCAGAGGCCCATACAATTGATGTCTTTGCTGGGTGAGGCTGCACTCCACGGCCCAGCATTCTACAGCAAAATCAGTAAACTGGTCATTGCAGAGTTGAAGGAAGGTGGCATGGATTTTTACGTGCCAAGACAAGAGCCAATGTTCAGATGGATGAGGTTCTCGGATCTGAGCACGTGGGAGGGCGATCGCAATCTGGCTCCCAGTTTTGTGAATGAAGATGGCGTCGAA

>GU991355|II.12/II.3|2009|CN|SH312

ATGAAGATGGCGTCTAACGACGCTTCCGCTGCCGCTGCTGCTAACAGCAACAACGACAACGCAAAATCTTCAAGTGACGGAGTGCTTTCTAGCATGGCTGTCACTTTTAAACGAGCCCTCGGGGCGCGGCATAAACAGCCTCCCCCGAGGGAAATACCACAAAGGCCTCCACGGCCACCCACCCCAGAACTGGTCAAAAAGATCCCTCCTCCCCCACCCAACGGGGAGGATGAACTAGTGGTCTCTTACAGCGCCAAAGATGGCGTTTCTGGTTTGCCTGAGCTTACCACTGTCTGGCAGCCGGATGAAACCAATACGGCGTTCAGTGTTCCCCCACTCAATCAGAGGGAGAACAGGGACGCCAAGGAGCCGCTGACCGGAACAATCCTGGAAATGTGGGACGGGGAGATCTATCATTACGGTCTGTATGTGGAGCGAGGCCTTGTACTTGGTGTGCACAAACCACCAGCTGCCATCAGCCTCGCCAAGGTTGAGCTAGCACCACTCTCTCTGTTTTGGAGGCCAGTGTACACACCACAGTATCTCATCTCTCCAGACACTCTCAGGAGACTGCATGGAGAGTCGTTCCCCTACACAGCCTTTGACAACAACTGCTATGCCTTCTGTTGTTGGGTTCTGGACCTAAACGACTCGTGGTTGTGCAGGAGAATGATCCAGAGAACAACTGGTTTCTTCAGACCTTACCAAGACTGGAACAGGAAACCCCTCCCCACCATGGATGACTCCAAATTAAAGAAGGTAGCCAACATATTCTTGTGTGCACTATCTTCGCTATTCACTAGGCCCATCAAAGACATAATAGGAAAGGTGAGACCGCTCAACATCCTTAACATCTTGGCCTCATGTGATTGGACTTTTGCAGGCATAGTGGAATCTTTGATCCTCTTGGCAGAGCTCTTTGGAGTTTTCTGGACACCCCCAGATGTGTCTGCGATGATCGCCCCCTTACTAGGTGACTACGAACTGCAGGGGCCTGAGGACCTTGCAGTGGAACTCGTACCAGTAGTGATGGGGGGGATTGGTTTGGTGTTAGGATTCACCAAAGAGAAGATCGGAAAAATGTTGTCATCTGCTGCATCCACCTTAAGAGCTTGTAAAGACCTTGGTGCATACGGACTGGAAATCCTAAAATTGGTCATGAAGTGGTTCTTCCCAAAGAAAGAGGAAGCAAATGAGCTGGCTATGGTGAGGTCCATCGAGGATGCGGTGCTGGACCTCGAAGCAATTGAGAACAACCACATGACTGCCCTCCTCAAAGACAAAGACAGCTTGGCAACCTACATGAGAACCCTTGACCTCGAGGAGGAGAAAGCCAGGAAGCTTTCAACCAAGTCTGCTTCACCTGACATCGTGGGCACAATCAACGCTCTCCTGGCGAGAATCGCCGCTGCACGTTCCCTGGTGCATCGGGCGAAAGAGGAACTCTCCAGCAGACCAAGACCTGTTGTTGTTATGATATCAG-CAG-CCAGG-ATAGGGAAAACTCACCTTGCCAGGGAGCTAGCCAAGAAAATCGCAGCTTCGCTTACAGGGGACCAGCGCGTGGGTCTCATCCCTCGCAATGGCGTTGATCACTGGGATGCATACAAGGAAGAAAGAGTCGTCCTATGGGACGATTATGGAATGAGTCATCCCATC-ACGACGCCCTCAGGTTACAGGAACTTGCTGACACCTGCCCCCTTACGCTAAATTGTGACAGGATTGAGAACAAAGGAAAGGTCTTTGGCAGTGATGCCATAATCATCACCACTAATCTGGCCAACCCAGCACCATTGGACTATGTCAACTTTGAAGCATGCTCGAGGCGCATCGACTTCCTCGTGTACGCAGAAGCCCCTGAGGTCGAGAAAGCAAAGCGTGATTTCCCAGGTCAACCTGACATGTGGAAGAACGCATTTAGTTCTGACTTCTCGCACATAAAACTGGCGCTGGCTCCGCAAGGTGGCTTCGACAAGAACGGAAACACCCCACACGGGAAAGGCGTTATGAAAACCCTCACCACTGGTTCCCTCATCGCCCGAGCGTCAGGGCTACTCCATGAGAGGTTAGATGAGTATGAGCTGCAG>GGCCCAACCCCCACCACCTTTAACTTTGACCGCAACAAGGTGCTTGCGTTCAGACAGCTTGCTGCCGAAAACAAGTACGGATTGTTGGACACAATGAGAGTTGGAAAACAGCTCAAGGATGTCAAGACCATGCCAGAGCTCAAACAAGCACTCAAAAATGTCGCAATCAAGAATTGTCAGATAGTGTATGGTGGCTGCACTTATAGGCTTGAGTCTGATGGCAAGGGTAATGTGAAAGTTGACAGAGTTCAGAGCACCTCCGTTCAGACCAACAATGAGCTGGCTGGCGCCCTGCACCATCTAAGGTGCGCCAGAATCAGGTACTATGTCAAGTGTGTTCAGGAGGCCCTGTATTCTATCATCCAGATTGCTGGGGCTGCATTTGTCACCACGCGCATCATCAAGCGTGTGAACATTCAAGACTTATGGTCCAAGCCACAAGTGGAAAACACAGAGGAGGCTACCGACAAGGACGGGTGCCCAAAACCCAAAGATATTGAGGAGTTCGTCATTTCATCTGACGACATTAAAACTGAGGGTAAGAAAGGGAAGAACAAGGCTGGCCGTGGCAAGAAGCACACAGCCTTCTCAAGTAAAGGTCTCAGTGATGAAGAGTATGATGAGTACAAGAGAATTAGAGAGGAAAGGAATGGCAAGTACTCCATAGAAGAGTACCTACAGGACAGGGACAAATACTATGAGGAGGTGGCCATTGCCAGGGCGACCGAGGAAGACTTCTGTGAAGAGGAGGAGGCCAAGATCCGGCAAAGGATCTTCAGGCCAACAAGGAAACAACGCAAGGAAGAAAGAGCTTCTCTCGGTTTAGTCACAGGTTCTGAAATTAGGAAAAGGAACCCAGAAGACTTCAAGCCTAAGGGGAAACTATGGGCCGACGATGACAGAAATGTGGACTACAATGAAAAACTCAGCTTTGAGGCCCCACCAGGCATCTGGTCAAGGATAGTCAACTTTGGTTCAGGTTGGGGCTTTTGGGTTTCCCCCAGCCTGTTCATAACATCAACCCATGTCATACCCCAGGGCGCACAGGAGTTCTTTGGTGTTCCCATCAAACAAATTCAGATACACAAATCGGGTGAATTCTGTCGCTTGAGGTTTCCAAAGCCGATCAGAACTGACGTGACAGGCATGATCTTAGAAGAAGGTGCACCCGAAGGGACCGTGGTCACATTACTCATCAAGAGGCCAACTGGAGAGCTCATGCCCCTGGCAGCCAGAATGGGAACCCACGCAACCATGAAGATTCAAGGGCGCACTGTTGGAGGTCAAATGGGCATGCTCCTAACAGGGTCCAACGCCAAGAGTATGGACCTGGGCACCACACCAGGTGACTGTGGCTGCCCCTACATTTACAAGAGGGGGAATGACTACGTAGTCATTGGAGTCCACACAGCTGCCGCCCGTGGAGGAAACACTGTCATTTGTGCCACCCAGGGGAGCGAGGGAGAAGCCACACTTGAAGGCGGTGACAGCAAGGGGACCTACTGTGGTGCACCAATCTTAGGTCCAGGAAGTGCCCCAAAGCTCAGCACCAAGACTAAGTTCTGGAGGTCATCCACGGCACCACTCCCACCTGGTACCTATGAGCCAGCCTACCTCGGCGGCAAGGACCCCAGAGTTAAGGGTGGCCCTTCATTGCAACAAGTTATGAGGGACCAACTGAAACCATTCACTGAGCCTAGGGGTAAACCACCAAGACCAAGTGTGCTAGAAGCCGCCAAGAAAACCATCGTCAATGTCCTTGAACAGACAATTGATCCACCTCAAAAATGGTCGTTTGCGCAGGCATGCGCATCCCTTGACAAGACCACTTCCAGCGGTCACCCGCACCATATGCGGAAAAACGACTGCTGGAACGGGGAATCCTTTACAGGAAAATTGGCAGACCAGGCTTCCAAGGCCAACCTGATGTTCGAGGAGGGAAAGAGCATGACCCCAGTTTACACGGGTGCGCTTATGGACGAGCTGGTCAAGACTGACAAGATTTATGGCAAGATCAAAAAGAGGCTTCTCTGGGGTTCGGACCTGGCGACCATGATTCGGTGCGCCCGAGCATTCGGAGGCCTGATGGAGGAACTCAAAGCACATTGTGTCACACTACCTGTCAGAGTGGGTATGAATATGAATGAGGATGGTCCTATCATCTTTGAGAGACACTCCAGATATAAATATCATTATGATGCTGATTACTCCCGGTGGGACTCAACACAACAAAGAGCCGTGCTAGCAGCAGCCTTGGAAATCATGGTCAAGTTCTCCCCAGAGCCGCACCTGGCCCAAAAGGTTGCAGAAGACCTTCTTTCTCCCAGCGTGATGGACGTGGGTGATTTCAAAATATCAATCAATGAGGGTCTCCCCTCCGGAGTGCCCTGCACCTCCCAATGGAATTCCATCGCCCACTGGCTCCTCACTCTCTGTGCACTCTCCGAGGTTACAAACCTGTCTCCTGACATCATTCAGGCCAACTCTCTCTTTTCTTTCTACGGTGATGATGAAATTGTGAGTACAGACATAAAATTGGACCCAGAAAAACTGACAGCAAAACTCAAGGAATACGGGTTGAAACCGACCCGCCCTGACAAGACTGAAGGGCCTCTTGTCATCTCCGAAGACCTGAATGGCCTGACCTTCCTGCGGAGGACCGTGACCCGCGACCCAGCAGGCTGGTTTGGAAAGTTGGAACAGAGCTCAATACTCAGACAAATGTACTGGACTAGGGGCCCCAACCATGAAGATCCATCTGAAACAATGATACCACACTCCCAGAGGCCCATACAATTGATGTCTTTGCTGGGTGAGGCAGCACTCCACGGCCCAGCATTCTACAGCAAAATCAGTAAACTGGTCATTGCAGAGTTGAAGGAAGGTGGCATGGATTTTTACGTGCCAAGACAAGAGCCAATGTTCAGATGGATGAGATTCTCGGATCTGAGCACGTGGGAGGGCGATCGCAATCTGGCTCCCAGTTTTGTGAATGAAGATGGCGTCGAA

>KC662537|II.12/II/13|2013|KR|Hy-718

ATGAAGATGGCGTCTAACGACGCTTCCGCTGCCGCTGCTGCCAACAGCAACAACGACATCGCAAAATCTTCAAGTGACGGAGTGCTTTCTAGCATGGCTGTCACTTTTAAACGAGCCCTCGGGGCGCGGCCTAAACAGCCTCCCCCGAGGGAAATACCACAGAGACCCCCACGACCACCCACACCAGAACTGGTCAAAAAGATCCCTCCTCCCCCGCCCAACGGGGAGGATGAACCAGTGGTTTCTTATAGCGTCAAAGATGGTGTTTCCGGCTTGCCTGAGCTTATCACTGTCAGGCAGCCGGACGAAGTCAACACGGCGTTCAGTGTTCCCCCACTCAACCAAAGGGAGAATAGGGACGCCAAGGAACCACTAACTGGAACAATTCTGGAAATGTGGGACGGGGAGATCTACCATTACGGCCTGTATGTGGAACGAGGTCTAGTACTTGGCGTGCACAGACCACCTGCTGCCATCAGCCTTGCCAAGGTTGAACTAACACCACTCTCTCTGTTTTGGAGGCCAGTGTACACACCTCAGTATCTCATTTCTCCAGACACTCTTAGGAGGCTGCACGGAGAATCGTTCCCCTATACGGCCTTTGACAACAATTGCTATGCCTTCTGTTGTTGGGTCCTGGACCTAAACGACTCGTGGTTGTGTAGGAGAATGATCCAGAGGACAACTGGTTTCTTTAGGCCCTACCAAGACTGGAATAGGAAACCCCTTCCTACTATGGATGATTCCAAGTTGAAGAAGGTAGCTAACGTATTCCTGTGCGCGCTTTCTTCACTATTCACCAGGCCCATCAAAGACATAATAGGGAAGCTGAGACCTCTTAACATCCTTAACATCTTGGCCTCATGTGATTGGACTTTTGCAGGCATAGTGGAATCCTTGATACTCTTGGCAGAGCTCTTTGGAGTTTTCTGGACACCCCCAGATGTGTCTGCGATGATCGCCCCCTTACTGGGTGAATACGAGCTGCAGGGGCCTGAGGACCTTGCAGTGGAACTCGTCCCAATAGTGATGGGGGGGATTGGTTTGGTGCTAGGATTTACCAAAGAGAAGATTGGGAAAATGTTGTCATCTGCTGCATCCACCTTAAGAGCTTGCAAAGACCTTGGTGCATACGGGCTGGAAATCTTGAAATTAGTCATGAAGTGGTTCTTCCCAAAGAAAGAGGAAGCAAATGAGCTGGCTATGGTGAGATCCATCGAGGACGCGGTGCTGGACCTCGAGGCAATTGAGAACAACCACATGACTGCCCTTCTTAAAGACAAAGATAGCTTGGCAACCTATATGAGAACCCTCGACCTCGAGGAGGAGAAGGCCAGGAAGCTCTCAACCAAGTCTGCTTCACCTGACATCGTGGGTACAATCAATGCTCTTCTGGCGAGAATCGCCGCTGCGCGCTCCCTGGTGCATCGGGCGAAGGAGGAGCTCTCTAGCAGACCAAGACCTGTTGTTGTGATGATATCAGGCAGACCAGGGATAGGGAAAACCCACCTTGCCAGGGAATTGGCCAAGAGAATTGCAGCTTCCCTCACAGGGGACCAGCGTGTGGGTCTCATCCCTCGCAATGGCGTTGACCATTGGGACGCATACAAGGGAGAAAGAGTCGTCCTGTGGGACGATTATGGAATGAGTAATCCCATCCATGATGCCCTCAGATTGCAAGAACTTGCTGACACCTGCCCCCTCACGCTAAATTGTGACAGGATTGAGAATAAAGGAAAGGTATTTGACAGTGATGCCATAATCATCACCACTAACCTGGCCAACCCAGCACCACTGGACTATGTTAACTTTGAAGCATGCTCGAGGCGCATTGACTTCCTCGTGTATGCAGATGCCCCTGAGGTCGAAAAGGCAAAACGTGACTTCCCAGGTCAACCTGACATGTGGAAGAACGCTTTTAGTCCTGACTTCTCGCACATAAAACTGACGCTGGCTCCGCAGGGAGGTTTCGACAAGAACGGAAACACCCCACATGGGAAAGGCGTCATGAAAACTCTCACCACTGGCTCTCTCATCGCCCGAGCATCAGGGCTACTCCATGAGAGGTTGGATGAGTACGAGCTGCAG>GGCCCAACCCCCACCACCTTCAACTTCGACCGCAACAAGGTGCTTGCTTTTAGACAGCTTGCTGCTGAAAACAAGTACGGGTTAATGGACACAATGAGAGTTGGTAGACAACTCAAGGATGTCAGGACTATGCCAGAGCTCAAACAGGCACTCAAGAATATCTCAATCAAGAGGTGTCAGATAGTGTACAGTGGCTGCACCTACACGCTTGAATCTGATGGCAAGGGTGACGTGAAAGTTGATAGAGTTCAGAGCGCCACTGTGCAGACCAACAATGAGCTGGCTGGCGCCCTACACCATCTAAGGTGTGCCAGAATTAGATACTATGTCAAGTGTGTTCAGGAGGCCCTGTACTCCATCATCCAAATTGCTGGTGCTGCATTTGTCACCACGCGCATCGTCAAGCGCATGAACATACAAGATCTTTGGTCCAAACCACAGGTTGAAGATACAGAGGAGACCGCTAGCAAGGATGGGTGCCCAGAACCCAAAGATGATGAAGAGTTTGTCGTCTCATCCGACGACATCAAAACTGAGGGCAAGAAAGGGAAGAACAAATCTGGCCGTGGCAAGAAGCATACAGCATTCTCAAGTAAAGGTCTCAGTGATGAAGAGTACGATGAGTACAAGAGAATCAGAGAAGAAAGAAACGGCAAATACTCCATAGAAGAGTACCTTCAGGACAGAGACAAGTATTATGAGGAGGTGGCCATCGCCAGGGCGACCGAAGAGGACTTCTGTGAGGAAGAAGAAGCCAAGATCCGACAAAGGATCTTCAGGCCAACAAGGAAACAACGCAAGGAGGAGAGAGCCTCTCTCGGCCTGGTCACAGGCTCTGAAATCAGAAAAAGAAACCCAGACGATTTCAAGCCTAAAGGGAAGCTGTGGGCTGATGACGACAGGAGTGTTGACTACAACGAGAGAATCAATTTTGAAGCCCCACCAAGCATCTGGTCGAGGATAGTCAACTTTGGCTCAGGTTGGGGCTTTTGGGTCTCCCCCAGTCTGTTCATAACATCAACTCATGTCATACCCCATGGCGCACAGGAGTTCTTTGGGGTTTCTATCAAACAGATTCAGATACACAAATCGGGTGAATTCTGTCGCTTGAGGTTTCCAAAACCAATCAGGACTGACGTGACAGGCATGATCTTGGAAGAAGGTGCGCCCGAAGGGACCGTGGCCACATTACTCATCAAGAGGCCAACTGGAGAACTCATGCCCTTGGCGGCCAGAATGGGAACCCATGCAACCATGAAGATCCAAGGGCGCACCGTTGGAGGTCAAATGGGCATGCTTCTAACAGGATCCAACGCCAAGAGTATGGATCTGGGCACCACACCGGGTGACTGTGGTTGTCCCTACATTTACAAGAGGGGGAATGACTACGTAGTCATTGGAGTCCACACGGCTGCCGCCCGTGGAGGAAACACTGTCATATGTGCCACCCAGGGGAGTGAGGGAGAAGCCACACTTGAAGGTGGTGACAACAAGGGAACCTACTGTGGTGCACCGATCTTAGGTCCAGGTAGTGCCCCAAAACTCAGTACCAAGACTAAATTTTGGAGATCATCCACAGCACCACTCCCACCTGGTACCTATGAACCAGCCTACCTTGGCGGCAAAGACCCCAGAGTCAAGGGTGGTCCTTCATTGCAACAAGTTATGAGGGACCAGCTGAAACCATTTACTGAACCCAGGGGCAAACCACCAAAACCAAGTGTGTTAGAGGCTGCCAAGAAAACCATCATCAATGTCCTTGAGCAAACAATTGATCCACCCCAAAAATGGTCATTCGCGCAAGCATGCGCGTCCCTCGACAAGACCACCTCTAGTGGCCACCCGCACCACATGCGGAAAAACGACTGCTGGAACGGGGAGTCCTTCACAGGCAAATTGGCAGACCAGGCTTCCAAGGCTAACCTGATGTACGAAGAGGGAAAGAACATGACTCCAGTTTACACGGGTGCGCTTAAGGACGAGCTGGTCAAGACTGACAAAATTTATGGCAAAATCAAAAAGAGGCTTCTCTGGGGCTCGGACCTGGCGACCATGATCCGGTGTGCTCGGGCTTTTGGGGGCCTGATGGATGAACTTAAAGCACATTGTGTCACACTCCCCGTCAGAGTGGGTATGAATATGAATGAGGATGGTCCTATCATCTTTGAAAGACATTCCAGATACAAATATCACTATGATGCTGATTACTCTCGGTGGGACTCAACACAACAGCGGGCCGTACTAGCAGCAGCCTTAGAAATCATGGTTAAGTTCTCCCCAGAACCTCATCTGGCCCAAAAGGTTGCAGAAGACCTTCTCTCTCCCAGCGTGATGGATGTAGGTGATTTCAAAATATCAATTAATGAGGGCCTCCCCTCTGGGGTACCCTGCACCTCCCAATGGAATTCCATCGCCCACTGGCTCCTCACCCTCTGTGCACTTTCTGAGGTCACAAACCTGTCCCCTGACATTATCCAGGCCAATTCCCTCTTCTCCTTCTATGGTGATGATGAAATTGTGAGCACAGACGTAAAGCTGGACCCAGAGAAGCTGACAGCAAAACTCAAGGAATACGGGCTGAAACCAACCCGCCCTGACAAGACTGAGGGGCCCCTTGTTATCTCTGAGGACCTGAATGGCTTGACTTTCCTGCGGAGGACTGTAACCCGCGATCCAGCTGGCTGGTTTGGAAAACTGGAACAGAGTTCAATACTTAGGCAAATGTACTGGACTAGGGGCCCTAATCATGAAGACCCATCTGAAACAATGATACCACACTCCCAAAGACCCATACAATTAATGTCTTTGCTGGGCGAGGCTGCCCTCCACGGCCCAGCGCTCTACAGCAAAATCAGCAAGTTAGTTATTGCAGAACTAAAGGAAGGTGGCATGGATTTCTACGTGCCCAGACAAGAGCCAATGTTCAGATGGATGAGATTCTCAGATCTGAGCACGTGGGAGGGCGATCGCAATCTGGCTCCCAGTTTTGTGAATGAAGATGGCGTCGAA

>KF306213|II.12/II.3|2013|CN|Jingzhou/2013402

ATGAAGATGGCCTCTAACGACGCTTCCGCTGCCGCTGCTGCTAACAGCAACAACGACACCGCAAAATCTTCAAGTGACGGAGTGCTTTCTAGCATGGCTGTCACTTTTAAACGAGCCCTCGGGGCGCGGCATAAACAGCCTCCCCCGAGGGAAATACCACAAAGGCCTCCACGGCCACCCACCCCAGAATTGATCAAAAAGATCCCTCCTCCCCCACCCAACGGGGAGGATGAACTAGTGGTCTCTTACAGCGCCAAAGATGGCGTTTCTGGTTTGCCTGAGCTCACCACTGTCAGGCAGCCGGATGAAACCAACACGGCGTTCAGTGTTCCCCCACTCAACCAGAGGGAGAACAGGGACGCCAAGGAGCCACTGACCGGAACAATCCTGGAAATGTGGGATGGGGAGATCTATCATTATGGTCTGTATGTGGAGCGAGGCCTTGTGCTTGGTGTGCACAAACCACCAGCTGCCATCAGCCTCGCCAAGGTTGAACTCGCACCACTTTCTCTATTTTGGAGGCCAGTGTACACACCACAGTATCTCATCTCTCCAGACACTCTTAGGAGGCTGCATGGAGAGTCGTTCCCTTACACAGCCTTTGACAACAACTGCTATGCCTTCTGTTGTTGGGTTCTTGACCTAAACGACTCGTGGTTGTGCAGGAGAATGATCCAAAGGACAACTGGTTTCTTCAGACCTTACCAAGACTGGAACAGGAAACCCCTCCCCACTATGGATGACTCCAAATTAAAGAAGGTAGCCAACATATTCTTGTGTGCGCTATCTTCGCTATTCACTAGGCCTATCAAAGATATAATAGGAAAGGTGAGACCGCTCAACATCCTTAACATCTTGGCCTCATGTGATTGGACTTTTGCAGGCATAGTAGAATCTTTGATTCTCTTGGCAGAGCTCTTTGGAGTTTTCTGGACACCCCCAGATGTGTCTGCGATGATCGCCCCCTTACTAGGTGACTACGAACTGCAGGGGCCTGAGGACCTTGCAGTGGAACTCGTACCAGTAGTGATGGGGGGGATTGGTTTGGTGCTAGGATTCACCAAAGAGAAGATCGGAAAAATGTTGTCATCTGCTGCATCCACCTTAAGAGCTTGTAAAGATCTTGGTGCATATGGATTGGAAATCCTAAAATTGGTCATGAAGTGGTTCTTCCCAAAGAAAGAGGAGGCAAATGAGCTGGCCATGGTGAGGTCCATTGAGGATGCGGTGCTGGACCTCGAAGCAATTGAGAACAACCACATGACTGCCCTCCTCAAAGACAAAGACAGTTTGGCAACCTACATGAGAACCCTTGACCTCGAGGAGGAGAAAGCCAGGAAACTTTCAACCAAGTCTGCTTCACCTGACATCGTGGGCACAATCAACGCCCTACTGGCGAGAATCGCCGCTGCACGTTCCCTGGTGCATCGGGCGAAAGAGGAACTCTCCAGCAGGCCAAGACCTGTTGTTGTTATGATATCAGGCAGGCCAGGGATAGGGAAAACTCACCTTGCCAGGGAGCTAGCCAAGAAAATCGCGGCTTCGCTTACAGGGGACCAGCGTGTGGGCCTCATCCCTCGCAATGGCGTTGATCACTGGGATGCATATAAGGGTGAAAGAGTCGTCCTATGGGATGATTATGGAATGAGTAATCCCATCCACGACGCCCTCAGGTTACAGGAACTTGCTGACACCTGCCCCCTTACGCTGAACTGTGACAGGATTGAGAACAAGGGAAAGGTCTTTGACAGTGATGCCATAATCATCACCACTAATCTGGCCAACCCAGCACCATTGGACTATGTCAACTTTGAAGCATGCTCGAGACGCATTGACTTCCTCGTGTATGCAGAAGCCCCTGAGGTCGAGAAGGCAAAGCGTGATTTCCCAGGTCAACCTGACATGTGGAAGAACGCATTTAGTTCTGACTTCTCGCACATAAAATTGGCGCTGGCTCCGCAGGGTGGTTTCGACAAGAACGGAAACACCCCACACGGGAAAGGCGTTATGAAAACCCTTACCACTGGTTCCCTCATCGCCCGAGCATCAGGGCTACTCCATGAGAGGTTAGATGAGTATGAGCTGCAG>GGCCCAACTCCCACCACCTTTAACTTTGACCGCAATAAGGTGCTTGCGTTCAGACAGCTTGCTGCCGAAAACAAGTACGGATTGTTGGACACAATGAGAGTTGGAAAGCAGCTCAAGGATGTCAAGACCATGCCAGAGCTCAAACAGGCACTCAAGAATGTCGCAATCAAGAATTGTCAGATAGTGTATGGTGGCTGCACTTATAGGCTTGAGTCTGATGGCAAGGGTGATGTGAAAGTTGACAGAGTTCAGAACGCCACTGTACAGACCAACAATGAACTTGCCGGCGCCCTACACCACCTTAGGTGTGCCAGAATCAGATATTATGTCAAGTGTGTCCAAGAGGCCCTGTATTCCATCATCCAAATTGCTGGAGCTGCATTTGTGACCACGCGCATTGTCAAGCGCATGAACATACAAGACCTCTGGTCCAAGCCACAGGTAGAGGATACAGAGGAAACCGTCAGCAAGGATGGGTGCCCAAAACCCAAGGATGATGAAGAGTTCGTTGTCTCATCCGATGACATCAAAACCGAGGGCAAGAAGGGGAAGAACAAATCTGGCCGTGGTAAGAAGCACACGGCATTCTCAAGTAAAGGTCTCAGTGATGAGGAGTACGATGAGTACAAAAGGATCAGGGAAGAAAGAAATGGAAAATATTCCATAGAGGAATACCTCCAGGACAGAGACAAGTATTATGAGGAGGTGGCCATCGCCAGGGCGACCGAAGAGGACTTCTGTGAAGAGGAAGAGGCCAAGATCCGACAAAGGATTTTCAGGCCAACAAGGAAACAACGCAAAGAGGAGAGGACCTCCCTCGGTTTGGTCACAGGCTCTGAAATCAGGAAGAGGAACCCAGACGACTTCAAACCTAAAGGAAAGCTGTGGGCTGATGACGACAGGAGTGTTGACCACAATGAGAGACTCAATTTTGAAGCCCCGCCAAGCATCTGGTCGAGGATAGTCAACTTTGGTTCAGGTTGGGGCTTTTGGGTTTCCCCCAGCCTGTTCATAACATCAACCCATGTCATACCCCAGGGCGCACAGGAGTTCTTTGGTGTCCCCATCAAACAGATTCAGATACACAAATCGGGTGAATTCTGCCGTTTGAGGTTTCCAAAACCGATCAGAACTGACGTGACAGGCATGATCTTAGAAGAAGGTGCACCCGAAGGGACCGTGGTCACATTACTCATCAAAAGGCCAACTGGGGAACTCATGCCCCTGGCAGCCAGAATGGGAACCCATGCAACCATGAAGATTCAAGGGCGCACTGTTGGAGGTCAAATGGGCATGCTCCTAACAGGATCCAACGCTAAGAGTATGGACCTAGGCACCACACCAGGTGACTGTGGCTGTCCCTACATTTACAAGAGGGGAAATGACTACGTAGTCATTGGAGTTCACACGGCTGCCGCCCGTGGAGGAAACACTGTCATTTGTGCCACCCAGGGGAGCGAGGGAGAAGCCACACTTGAAGGCGGTGATAGCAAGGGGACCTACTGCGGTGCACCAATCTTAGGTCCAGGAAGTGCCCCAAAACTCAGCACCAAGACTAAATTCTGGAGGTCGTCCACGGCACCACTCCCACCTGGTACCTATGAGCCAGCCTACCTCGGCGGCAAGGACCCCAGAGTTAAGGGTGGCCCCTCATTACAACAAGTTATGAGGGACCAACTGAAACCATTCACTGAGCCCAGGGGCAAACCACCAAAGCCAAGTGTGTTAGAAGCTGCCAAGAAAACCATCGTCAACGTCCTTGAACAAACAATTGATCCACCTCAAAAATGGTCGTTTGCACAAGCTTGCGCTTCCCTTGACAAGACCACTTCCAGCGGTCATCCGCACCATATGCGAAAAAATGACTGTTGGAACGGGGAGTCCTTCACAGGAAAATTAGCAGACCAGGCTTCCAAGGCTAACCTGATGTTCGAGGAGGGAAAGAACATGACCCCAGTCTACACGGGTGCGCTTAAGGACGAGCTGGTCAAGACTGACAAGATTTATGGCAAGATCAAAAAGAGGCTTCTCTGGGGTTCGGACCTGGCGACCATGATCCGGTGCGCTCGGGCATTCGGAGGCCTGATGGAGGAACTCAAAGCACACTGTGTCACACTACCCGTCAGAGTGGGTATGAATATGAATGAGGATGGTCCTATCATCTTTGAGAGACACTCCAGATATAAATACCATTATGATGCTGATTACTCCCGGTGGGACTCAACACAACAAAGAGCCGTGTTGGCAGCAGCCTTAGAAATCATGGTCAAGTTCTCCCCAGAACCGCACCTGGCCCAAAAGGTTGCAGAAGACCTTCTTTCTCCCAGCGTGATGGACGTGGGTGATTTCAAGATATCAATCAATGAGGGTCTCCCCTCCGGGGTGCCCTGCACCTCCCAATGGAATTCCATCGCCCACTGGCTCCTCACTCTCTGTGCACTCTCTGAGGTCACAAACCTGTCCCCTGACATCATTCAGGCCAACTCTCTCTTTTCTTTCTACGGTGATGATGAAATTGTGAGTACAGACATAAAGTTGGACCCAGAAAAACTGACAGCAAAACTCAAGGAATACGGGTTGAAACCAACCCGCCCTGACAAGACTGAAGGACCACTTGTCATCTCTGAAGACCTGAATGGTCTAACTTTCCTGCGGAGGACCGTGACCCGCGACCCAGCGGGCTGGTTTGGAAAGTTGGAACAGAGTTCAATACTTAGACAAATGTATTGGACTAGGGGCCCCAACCATGAAGACCCATCTGAAACAATGATACCACATTCCCAGAGGCCCATACAATTGATGTCTTTGCTGGGTGAGGCTGCACTTCACGGCCCAGCATTCTATAGCAAAATCAGTAAACTGGTCATTGCAGAGTTGAAGGAAGGTGGCATGGATTTTTACGTGCCAAGACAAGAACCAATGTTCAGATGGATGAGATTCTCGGATCTGAGCACGTGGGAGGGCGATCGCAATCTGGCTCCCAGTTTTGTGAATGAAGATGGCGTCGAA

>KJ196276|II.12/II.13|2002|JP|Saitama/T80

ATGAAGATGGCGTCTAACGACGCTTCCGCTGTCGCTGCTGCCAACAGCAACAACGACATCGCAAAATCTTCAAGTGACGGAGTGCTTTCTAGCATGGCTGTCACTTTTAAACGAGCCCTCGGGGCGCGGCCTAAACAGCCTCCCCCGAGGGAAATACCACAGAGACCCCCACGACCACCCACACCAGAACTGGTCAAAAAGATCCCTCCTCCCCCGCCCAACGGGGAGGATGAACCAGTGGTTTCTTATAGCGTCAAAGATGGTGTTTCCGGCTTGCCTGAGCTTATCACTGTCAGGCAGCCGGATGAAGTCAACACGGCGTTCAGTGTTCCCCCACTCAACCAAAGGGAGAATAGGGACGCCCAGGAACCACTAACTGGAACAATTCTGGAAATGTGGGACGGGGAGATCTACCATTACGGCCTGTATGTGGAACGAGGTCTAGTACTTGGCGTGCACAAACCACCGGCTGCCATCAGCCTCGCCAAGGTTGAACTAACACCACTCTCTCTGTTTTGGAGGCCAGTGTACACACCTCAGTATCTCATCTCTCCAGACACTCTCAGGAGGCTGCACGGAGAATCGTTCCCCTATACGGCCTTTGACAACAATTGCTATGCCTTCTGTTGTTGGGTCCTGGACCTAAATGACTCGTGGTTGAGTAGGAGAATGATTCAGAGGACAACTGGTTTCTTTAGGCCCTACCAAGACTGGAATAGGAAACCCCTTCCTACCATGGATGATTCCAAGTTGAAGAAGGTAGCTAACGTGTTCCTGTGCGCGCTTTCTTCACTATTCACCAGGCCCATCAAAGACATAATAGGGAAGCTGAGACCTCTTAACATCCTTAACATCTTGGCCTCATGTGATTGGACTTTTGCAGGCATAGTGGAATCCTTGATACTCTTGGCAGAGCTCTTTGGAGTTTTCTGGACACCCCCAGATGTGTCTGCGATGATCGCCCCCTTACTGGGTGACTACGAGCTGCAGGGGCCCGAGGACCTTGCAGTGGAACTCGTCCCAATAGTGATGGGGGGGATTGGTTTGGTGCTAGGATTTACCAAAGAGAAGATTGGGAAAATGTTGTCATCTGCTGCATCCACCTTAAGAGCTTGCAAAGACCTTGGTGCATACGGGCTGGAAATCTTAAAATTAGTCATGAAGTGGTTCTTCCCAAAGAAAGAGGAAGCAAATGAGCTGGCTATGGTGAGATCCATCGAGGACGCGGTGCTGGACCTCGAGGCAATTGAGAACAACCACATGACTGCCCTTCTTAAAGACAAAGATAGCCTGGCAACCTACATGAGAACCCTCGACCTCGAGGAGGAGAAGGCCAGGAAGCTCTCAACCAAGTCTGCTTCACCTGATATCGTGGGTACAATCAACGCTCTTCTGGCGAGAATCGCCGCTGCGCGTTCCCTGGTGCATCGGGCGAAGGAGGAGCTCTCTAGCAGACCAAGACCTGTTGTTGTGATGATATCAGGCAGACCAGGGATAGGGAAAACCCACCTTGCCAGGGAATTGGCCAAGAGAATTGCAGCTTCCCTCACAGGGGACCAGCGTGTGGGTCTCATCCCTCGCAATGGCGTTGACCATTGGGACGCATACAAGGGAGAAAGAGTCGTCCTGTGGGACGATTATGGAATGAGTAATCCCATCCATGATGCCCTCAGATTGCAAGAACTTGCTGACACTTGCCCCCTCACGCTAAATTGTGACAGGATTGAGAATAAAGGAAAGGTATTTGACAGTGATGCCATAATCATCACCACTAACCTGGCCAACCCAGCACCACTGGACTATGTTAACTTTGAAGCATGCTCGAGGCGCATTGACTTCCTCGTGTATGCAGATGCCCCTGAGGTCGAAAAGGCAAAACGTGACTTCCCAGGTCAACCTGATATGTGGAAGAACGCTTTTAGTCCTGACTTCTCACACATAAAACTGACGCTGGCTCCGCAGGGAGGCTTCGATAAGAATGGAAACACCCCACATGGGAAAGGCGTCATGAAAACTCTCACCACTGGCTCTCTCATCGCCCGAGCATCAGGGCTACTCCATGAGAGGTTGGATGAGTACGAGCTGCAG>GGCCCAACCCCCACCACCTTCAACTTCGACCGCAACAAGGTGCTTGCTTTTAGACAGCTTGCTGCTGAAAACAGGTACGGGTTAATGGACACAATGAGAGTTGGCAGACAACTCAAGGATGTCAGGACCATGCCAGAGCTCAAACAGGCACTCAAGAATATCTCAATCAAGAGGTGTCAGATAGTGTACAGTGGCTGCACCTACACGCTTGAATCTGATGGCAAGGGTGACGTGAAAGTTGATAGAGTTCAGAGCGCCACTGTGCAGACCAACAATGAGCTGGCTGGTGCCCTACACCATCTAAGGTGTGCCAAAATTAGATACTATGTCAAGTGTGTTCAGGAGGCCCTGTACTCCATCATCCAAATTGCTGGTGCTGCATTTGTCACCACGCGCATCGTCAAGCGCATGAATATACAAGATCTTTGGTCCAAACCACAGGTGGAAGATACAGAGGAGACTACCAACAAGAATGGGTGCCCAGAACCCAAAGATGATGAAGAGTTTGTCGTCTCATCCGACGACATCAAAACTGAGGGCAAGAAAGGGAAGAACAAATCTGGCCGTGGCAAGAAGCATACAGCATTCTCAAGTAAAGGTCTCAGTGATGAAGAGTACGATGAGTACAAGAGAATCAGAGAAGAAAGAAACGGCAAATACTCCATAGAAGAGTACCTTCAGGACAGAGACAAGTATTATGAGGAGGTGGCCATCGCCAGGGCGACCGAAGAGGACTTCTGTGAGGAAGAAGAAGCCAAGATCCGACAAAGGATTTTCAGGCCAACAAGGAAACAACGCAAGGAGGAGAGAGCCTCTCTCGGCCTGGTCACAGGCTCTGAAATCAGAAAAAGAAACCCAGACGATTTCAAGCCTAAAGGGAAGCTGTGGGCTGATGACGACAGGAGTGTTGACTACAACGAGAGAATCAATTTTGAAGCCCCACCAAGCATCTGGTCGAGGATAGTCAACTTTGGCTCAGGTTGGGGCTTTTGGGTCTCCCCCAGTCTGTTCATAACATCAACTCATGTCATACCCCATGGCGCACAGGAGTTCTTTGGGGTTTCTATCAAACAGATTCAGATACACAAATCGGGTGAATTCTGTCGCTTGAGGTTTCCAAAACCAATCAGGACTGATGTGACAGGCATGATCTTAGAAGAAGGTGCGCCCGAAGGGACCGTGGCCACATTACTCATCAAGAGGCCAACTGGGGAACTCATGCCCTTGGCGGCCAGAATGGGAACCCATGCAACCATGAAGATCCAAGGGCGCACTGTTGGAGGTCAAATGGGCATGCTTCTAACAGGATCCAACGCCAAGAGTATGGATCTAGGCACCACACCGGGTGACTGTGGTTGTCCCTACATTTACAAGAGGGGGAATGACTACGTAGTCATTGGAGTCCACACGGCTGCCGCCCGTGGAGGAAACACTGTCATATGTGCCACCCAGGGGAGTGAGGGAGAAGCCACACTTGAAGGTGGTGACAGCAAGGGAACCTACTGTGGTGCACCAATCTTAGGTCCAGGCAGTGCCCCAAAACTCAGTACCAAGACTAAATTTTGGAGATCATCCACAGCACCACTCCCACCTGGTACCTATGAACCAGCCTACCTTGGCGGCAAGGACCCCAGAGTCAAGGGTGGTCCTTCATTGCAACAAGTTATGAGGGACCAGCTGAAACCATTCACTGAGCCCAGGGGCAAACCACCAAAACCAAGTGTGTTAGAGGCTGCCAAGAAAACCATCATCAATGTTCTTGAGCAAACAATTGATCCACCTCAAAAATGGTCATTCGCGCAAGCATGCGCATCCCTCGACAAGACCACCTCTAGTGGTCACCCGCACCACATGCGGAAAAACGACTGCTGGAACGGGGAGTCCTTCACAGGCAAATTGGCAGACCAGGCTTCCAAGGCTAACCTGATGTACGAAGAGGGAAAGAACATGACCCCAGTTTACACGGGTGCGCTTAAGGACGAGCTGGTCAAGACTGACAAAATTTATGGCAAAATCAAAAAGAGGCTTCTCTGGGGCTCGGACCTGGCGACCATGATCCGGTGCGCTCGGGCTTTTGGGGGCCTGATGGATGAATTTAAGGCACATTGTGTCACACTCCCCGTCAGAGTGGGTATGAATATGAATGAGGATGGTCCTATCATCTTTGAAAGACACTCCAGATACAAATATCACTATGATGCTGATTACTCTCGGTGGGACTCAACGCAACAGCGGGCCGTATTAGCAGCAGCCTTAGAAATCATGGTTAAGTTCTCCCCAGAACCTCATCTGGCCCAAAAGGTTGCAGAAGACCTTCTCTCTCCCAGCGTGATGGATGTAGGTGATTTCAAAATATCAATCAATGAGGGCCTCCCCTCCGGGGTACCCTGCACCTCCCAATGGAATTCCATCGCCCACTGGCTTCTCACCCTCTGTGCACTTTCTGAGGTCACAAACCTGTCCCCTGACATTATCCAGGCCAATTCCCTCTTTTCCTTCTATGGTGATGATGAAATTGTGAGCACAGACGTAAAGCTGGACCCAGAGAAGCTGACAGCAAAACTCAAGGAATACGGGCTGAAACCAACCCGCCCTGACAAGACTGAGGGGCCCCTTGTTATCTCTGAGGACCTGAATGGCTTGACCTTCCTGCGGAGGACTGTGACCCGCGATCCAGCTGGCTGGTTTGGAAAACTGGAACAGAGTTCAATACTTAGGCAAATGTACTGGACTAGGGGCCCTAATCATGAAGACCCATCTGAAACAATGATACCACACTCCCAAAGACCCATACAATTAATGTCTTTGCTGGGCGAGGCTGCCCTCCACGGCCCAGCGTTCTACAGCAAAATCAGCAAGTTAGTTATTGCAGAACTAAAGGAAGGTGGCATGGATTTCTACGTGCCCAGACAAGAGCCAATGTTCAGATGGATGAGATTCTCAGATCTGAGCACGTGGGAGGGCGATCGCAATCTGGCTCCCAGTTTTGTGAATGAAGATGGCGTCGAA

>KJ196282|II.12/II.12|2001|JP|Saitama/T15

ATGAAGATGGCGTCTAACGACGCTTCCGCTGCCGCTGCTGCTAACAGCAACAACGACACCGCAAAATCTTCAAGTGACGGAATGCTTTCTAGCATGGCTGTCACTTTTAAACGAGCCCTCGGGGCACGGCCTAAACAGCCTCCCCCGAGGGAAATACCACAAAGACCCCCACGACCACCCACCCCAGAACTGGTCAAAAAGATCCCCCCTCCTCCACCCAACGGGGAGGATGAACCAGTGGTTTCTTACAGCGTCAAAGATGGCGTTTCCGGCTTGCCTGAGCTTACCACTGTCAGGCAGCCGGGTGAAACCAACACGGCGTTCAGTGTTCCCCCACTCAACCAAAGGGAGAATAGGGACGCCAAGGAGCCACTAACTGGAACAATCCTGGAAATGTGGGACGGGGAGATCTACCATTACGGCCTGTATGTGGAACGAGGTCTTGTACTTGGTGTGCACAAACCACCGGCTGCCATCAGCCTCGCCAAGGTTGAATTAACACCACTCTCTCTGTTTTGGAGACCAGTGTATACACCACAGTATCTCATCTCTCCGGACACTCTCAGGAGACTGCACGGAGAGTCGTTTCCCTACACAGCCTTTGATAACAACTGCTATGCCTTCTGTTGTTGGGTCCTGGACCTAAACGACTCGTGGTTGTGCAGGAGAATGATCCAGAGGACAACTGGTTTCTTCAGGCCCTACCAAGACTGGAATAGGAAACCCCTCCCCACCATGGATGACTCCAAGTTGAAGAAGGTAGCTAACATATTTTTGTGCGCGCTATCTTCGCTATTCACTAGGCCCATCAAAGACATAATAGGAAAGTTGAGGCCTCTCAACATCCTTAACATCTTGGCTTCATGTGATTGGACTTTTGCAGGCATAGTGGAATCTTTGATTCTCTTGGCAGAGCTCTTTGGAGTTTTCTGGACACCCCCAGATGTGTCTGCGATGATCGCCCCTTTACTAGGTGACTACGAGCTGCAGGGGCCCGAGGACCTTGCAGTGGAACTCGTTCCAATAGTGATGGGGGGGATTGGTTTGGTGCTAGGATTCACCAAAGAGAAGATCGGGAAAATGTTGTCATCTGCTGCATCCACCTTAAGAGCTTGTAAAGACCTTGGTGCATACGGACTGGAAATTTTAAAATTGGTCATGAAGTGGTTCTTCCCAAAGAAAGAGGAAGCAAATGAGCTGGCTATGGTGAGATCCATCGAGGATGCGGTGCTGGACCTCGAGGCAATTGAAAATAACCACATGACTGCTCTCCTCAAAGACAAAGACAGCCTGGCAACCTATATGAGAACTCTTGACCTCGAGGAGGAGAAAGCCAGAAAGCTTTCAACCAAGTCTGCTTCACCTGATATCGTGGGCACAATCAACGCTCTCCTGGCGAGAATCGCCGCTGCACGCTCCCTGGTGCATCGGGCGAAAGAGGAGCTCTCCAGCAGACCAAGACCTGTTGTTGTGATGATATCAGGTAGGCCAGGGATAGGGAAAACCCACCTTGCCAGGGAATTGGCCAAGAAAATCGCAGCTTCTCTCACAGGGGACCAGCGTGTGGGTCTCATCCCGCGCAATGGTGTTGATCACTGGGACGCATATAAGGGAGAAAGAGTCGTTCTATGGGACGACTATGGAATGAGTAACCCCATCCACGACGCCCTCAGGTTACAAGAACTTGCTGACACCTGCCCCCTCACGCTAAATTGTGATAGGATTGAGAACAAAGGAAAGGTCTTTGACAGTGATGCCATAATCATCACCACTAACCTGACCAACCCAGCACCACTGGACTATGTCAATTTTGAAGCATGCTCGAGGCGTATCGACTTCCTCGTGTATGCAGATGCCCCTGAAGTCGAGAAGGCAAAACGTGATTTCCCAGGTCAACCTGACATGTGGAAGAACGCTTTCAGTCCTGACTTCTCGCACATAAAACTGATGCTGGCTCCGCAGGGTGGCTTCGACAAGAACGGAAACACCCCACATGGGAAAGGCGTCATGAAAACCCTCACCACTGGTTCCCTCATCGCACGAGCATCAGGGCTACTCCATGAGAGGTTAGATGAGTACGAGCTGCAG>GGCCCAACCCCCACTACCTTCAACTTTGACCGCAATAAGGTGCTTGCGTTCAGACAGCTTGCTGCTGAAAACAAGTACGGGTTGATGGACACAATGAGAGTCGGAAGACAGCTCAAGGATGTCAGGACCATGCCAGAGCTCAAACAAGCACTCAAGAATATCTCAATCAAGAGTTGCCAGATAGTGTATGGTGGCTGCACCTATATGCTTGAGTCTGATGGCAAGGGTGATGTGAAAGTTGACAGAGTTCAGAATGCTACTGTGCAGACCAACAATGAACTGGCCGGTGCCCTACACCATCTTAGGTGTGCCAGGATTAGATACTATGTCAAGTGCATTCAGGAGGCCCTGTATTCCATCATCCAAATTGCTGGAGCTGCATTTGTCACCACGCGCATTGTCAACCGCATGAACATACAAGACCTTTGGTCCAAGCCACAGGTGGAAGATACAGAGGAGACTGCTAGCAAGGATGGGTGCCCAAAACCCAAGGATGATGACGAGTTCGTTGTTTCATCCGACGACATCAAAACCGAGGGCAAGAAAGGAAAGAACAAGTCTGGCCGTGGTAAGAAGCACACGGCATTCTCAAGCAAAGGTCTCAGTGATGAGGAGTACGATGAGTACAAAAGAATCAGAGAAGAAAGAAACGGCAAGTACTCTATAGAGGAATACCTTCAGGACAGAGATAAGTATTATGAGGAGGTGGCCATCGCCAGGGCGACCGAAGAGGACTTCTGTGAAGAAGAAGAGGCCAAGATCCGACAAAGGATTTTTAGGCCAACAAGGAAACAACGCAAAGAGGAGAGGGCCTCTCTCGGCTTGGTCACAGGTTCTGAAATCAGGAAGAGGAACCCAGACGACTTCAAACCTAAAGGAAAGCTGTGGGCTGATGACGACAGGAGTGTTGACTACAATGAGAGACTCAATTTTGAAGCCCCACCAAGCATTTGGTCGAGGATAGTCAACTTTGGTTCAGGTTGGGGTTTTTGGGTTTCCCCCAGCCTGTTCATAACATCAACTCATGTCATACCCCAGGGCGCACAGGAGTTCTTTGGGGTTTCCATCAAGCAAATTCAGATACACAAATCGGGTGAATTCTGTCGCTTGAGGTTTCCAAAACCAATCAGAACTGATGTGACAGGCATGATCCTAGAAGAAGGTGCGCCCGAAGGGACCGTGGTCACATTACTCATCAAGAGACCAACTGGGGAACTCATGCCCTTGGCAGCCAGAATGGGAACCCATGCAACCATGAAGATACAAGGGCGCACTGTTGGGGGTCAAATGGGCATGCTCCTAACAGGATCTAACGCCAAGAGTATGGACTTGGGCACCACACCAGGTGACTGTGGCTGTCCCTACATTTACAAGAGAGGGAATGACTACATAGTCATTGGAGTCCACACGGCTGCTGCCCGTGGAGGAAACACCGTCATATGTGCCACCCAGGGGAGCGAGGGAGAAGCCACACTTGAAGGCGGTGACAACAAGGGAACCTACTGCGGCGCACCAATCTTAGGTCCAGGAAGTGCCCCAAAGCTCAGCACCAAGACTAAGTTTTGGAGATCATCCACAGCACCACTCCCACCTGGTACCTATGAACCAGCCTACCTTGGCGGCAAGGACCCCAGAGTCAAAGGTGGCCCCTCATTGCAACAAGTTATGAGAGACCAGCTGAAACCATTCACTGAGCCCAGGGGTAAACCACCAAAACCAAGTGTGTTAGAGGCTGCCAAGAAAACCATCATCAATGTTCTTGAACAAACAATTGATCCACCTCAAAAATGGTCATTCGCGCAGGCATGCGCATCCCTGGACAAGACCACTTCCAGTGGCCACCCGCACCACATGCGGAAAAACGACTGCTGGAACGGGGAGTCCTTCACAGGCAAATTGGCAGACCAGGCTTCCAAGGCTAACCTGATGTTCGAAGAGGGAAAGAACATGACCCCAGTCTACACAGGTGCGCTTAAGGACGAGCTGGTCAAGACTGACAAAATTTATGGCAAGATCAAAAAGAGGCTTCTCTGGGGCTCGGATCTGGCGACCATGATCCGGTGTGCTCGAGCGTTTGGAGGCCTGATGGAGGAACTCAAAGCACATTGTGTCACACTACCCGTCAGAGTAGGTATGAATATGAATGAGGATGGCCCTATCATCTTTGAGAGACACTCCAGATATAAGTATCATTATGATGCTGATTACTCCCGGTGGGACTCAACACAACAAAGAGCCGTGTTAGCAGCAGCCTTAGAAATCATGGTTAAGTTCTCCCCAGAACCGAATCTGGCCCAAAAGGTTGCAGAAGACCTTCTCTCCCCCAGCGTGATGGACGTAGGTGACTTCAAAATATCAATCAATGAGGGCCTCCCCTCCGGGGTGCCCTGCACCTCCCAATGGAATTCCATCGCCCACTGGCTCCTCACCCTCTGTGCGCTTTCTGAGGTTACAAACCTGTCCCCTGACATTATCCAGGCTAATTCCCTCTTTTCCTTCTACGGTGATGATGAAATTGTGAGCACAGACATAAAATTGGACCCAGAGAAGTTGACAGCAAAACTTAAGGAATACGGGTTGAAACCGACCCGCCCTGACAAGACTGAGGGACCCCTTGTTATCTCTGAGGACCTGGATGGCCTAACCTTCCTGCGGAGGACTGTAACCCGCGACCCAGCTGGCTGGTTTGGAAAGCTGGAACAGAGCTCAATACTTAGGCAAATGTATTGGACTAGGGGCCCTAACCATGAAGACCCATCTGAAACAATGATACCACACTCCCAAAGACCCATACAATTGATGTCTTTGCTGGGCGAGGCTGCACTCCACGGCCCAGCATTCTACAGCAAAATCAGCAAGCTGGTCATTGCAGAGCTGAAGGAAGGTGGCATGGATTTTTACGTGCCCAGACAAGAGCCAATGTTCAGATGGATGAGGTTTTCAGATCTGAGCACGTGGGAGGGCGATCGCAATCTGGCTCCCAGTTTTGTGAATGAAGATGGCGTCGAG

>KJ196294|II.12/II.12|2000|JP|Saitama/KU16

ATGAAGATGGCGTCTAACGACGCTTCCGCTGCCGCTGCTGCTAACAGCAACAACGACACCGCAAAATCTTCAAGTGACGGAGTGCTTTCTAGCATGGCTGTCACTTTTAAACGAGCCCTCGGGGCACGGCCTAAACAGCCTCCCCCGAGGGAAATACCACAAAGGCCCCCACGACCACCCACCCCAGAACTGGTCAAAAAGATCCCTCCTCCTCCACCTAACGGGGAGGATGAACCAGTGGTTTCTTACAGCGTCAAAGATGGCGTTTCCGGCTTGCCTGAGCTCACCACTGTCAGGCAGCCGGGTGAAACCAACACGGCGTTCAGTGTTCCCCCACTCAACCAAAGGGAGAATAGGGACGCCAAGGAGCCATTAACTGGAACAATCCTGGAAATGTGGGACGGGGAGATCTACCATTACGGCCTGTATGTGGAACGAGGTCTTGTACTTGGTGTGCACAAACCACCGGCTGCCATCAGCCTCGCCAAGGTTGAACTAACACCACTTTCTCTGTTCTGGAGACCAGTGTATACACCACAGTATCTCATCTCTCCGGACACTCTCAGGAGACTGCACGGAGAGTCGTTCCCCTACACAGCCTTTGACAACAACTGCTATGCCTTCTGTTGTTGGGTCCTGGACCTAAACGACTCGTGGTTGTGCAGGAGAATGATCCAGAGGACAACTGGTTTCTTCAGGCCTTACCAAGACTGGAATAGGAAACCCCTTCCCACCATGGATGACTCCAAGTTGAAGAAGGTAGCTAACATATTCTTGTGTGCGCTATCTTCGCTATTCACTAGGCCCATCAAAGACATAATAGGAAAGTTGAGGCCTCTTAACATCCTTAACATCTTGGCCTCATGTGATTGGACTTTTGCAGGCATAGTGGAATCTTTGATCCTCTTGGCAGAGCTCTTTGGAGTTTTCTGGACACCCCCAGATGTGTCTGCGATGATCGCCCCTTTACTAGGTGACTACGAGCTGCAGGGGCCCGAGGACCTTGCAGTGGAACTCGTTCCAATAGTGATGGGGGGGATTGGTTTGGTGCTAGGATTCACCAAAGAGAAAATCGGGAAAATGTTGTCATCTGCTGCATCCACCTTAAGAGCTTGTAAAGACCTTGGTGCATACGGACTGGAAATCTTAAAATTGGTCATGAAGTGGTTCTTCCCAAAGAAAGAGGAAGCAAATGAGCTGGCTATGGTGAGGTCCATCGAGGATGCGGTGCTGGACCTCGAGGCAATTGAGAACAATCACATGACTGCCCTCCTCAAAGACAAAGACAGCCTGGCAACCTATATGAGAACTCTTGACCTCGAGGAGGAGAAAGCTAGGAAGCTCTCAACCAAGTCTGCTTCACCTGATATCGTGGGCACAATCAACGCTCTCCTGGCGAGAATCGCCGCTGCACGTTCCCTGGTGCATCGGGCGAAAGAGGAACTCTCCAGCAGACCAAGACCTGTTGTTGTTATGATATCAGGCAGGCCAGGGATAGGGAAAACCCACCTTGCCAGGGAATTGGCCAAGAGAATCGCAGCTTCTCTCACAGGGGACCAGCGTGTGGGTCTCATCCCTCGCAATGGTGTTGATCACTGGGACGCATATAAAGGAGAAAGAGTCGTCCTATGGGACGATTATGGAATGAGTAATCCCATCCACGACGCCCTCAGGTTACAAGAACTTGCTGACACCTGCCCCCTCACGCTAAATTGTGACAGGATTGAGAACAAAGGAAAGGTCTTTGACAGTGATGCCATAATCATCACCACTAATCTGGCCAACCCAGCACCACTGGACTATGTCAATTTTGAAGCATGCTCGAGGCGCATCGACTTCCTCGTGTATGCAGATGCCCCTGAGGTCGAGAAGGCAAAGCGCGATTTCCCAGGTCAACCTGACATGTGGAAGAACGCTTTCAGTCCTGACTTCTCGCACATAAAACTGATGCTGGCTCCGCAGGGTGGCTTCGACAAGAACGGAAACACCCCACATGGGAAAGGCGTCATGAAAACCCTCACCACTGGTTCCCTCATCGCTCGAGCATCGGGGCTACTCCATGAGAGGTTAGATGAGTACGAGCTGCAG>GGCCCAACCCCCACCACCTTCAACTTTGACCGCAACAAGGTGCTTGCGTTCAGACAGCTTGCTGCCGAAAACAAGTACGGGTTGATGGACACAATGAGAGTTGGAAGACAGCTCAAGGATGTCAGGACCATGCCAGAGCTCAAACAAGCACTCAAGAATATCTCAATTAATAGTTGTCAGATAGTGTATGGTGGCTGCACCTATAAGCTTGAGTCTGATGGCAAGGGTGATGTGAAAGTTGACAGAGTTCAGAACGCCACTGTACAGACCAACAATGAACTTGCCGGTGCCCTACATCATCTTAGGTGTGCCAGAATTAGATATTATGTCAAGTGTGTTCAGGAGGCCCTATATTCCATCATCCAAATTGCTGGAGCTGCATTTGTAACCACGCGCATTGTCAAGCGCATGAACATACAAGACCTTTGGTCCAAGCCACAGGTGGAAGATACAGAGGAGACTGCTAGCAAGGATGGGTGCCCAAAACCCAAGGATGATGACGAGTTCGTTGTCTCATCCGACGACATCAAAACCGAGGGCAAGAAAGGGAAGAACAAGTCTGGCCGTGGTAAGAAGCACACAGCATTCTCAAGCAAAGGTCTCAGTGATGAAGAGTACGATGAGTACAAAAGAATCAGAGAAGAAAGAAACGGCAAATACTCCATAGAGGAATACCTCCAGGACAGAGACAAGTATTATGAGGAGGTGGCCATCGCCAGGGCGACCGAAGAGGACTTCTGTGAAGAAGAAGAGGCCAAGATCCGACAAAGGATTTTCAGGCCAACAAGGAAACAACGCAAAGAGGAGAGGGCCTCTCTCGGTTTGGTCACAGGCTCTGAAATCAGGAAGAGGAACCCAGACGACTTCAAGCCTAAAGGAAAGCTGTGGGCTGATGACGACAGGAGTGTTGACTACAATGAGAGACTCAATTTTGAAGCCCCACCAAGCATCTGGTCGAGGATAGTCAACTTTGGTTCAGGTTGGGGCTTTTGGGTTTCCCCCAGCCTGTTCATAACATCAACTCATGTCATACCCCAGGGCGCACAGGAGTTCTTTGGGGTTTCCATCAAACAAATTCAGATACACAAATCGGGTGAATTCTGTCGCTTGAGGTTTCCAAAACCAATCAGAACTGACGTGACAGGCATGATCCTAGAAGAAGGTGCGCCCGAAGGGACCGTGGTCACACTACTCATCAAGAGGCCAACTGGAGAACTCATGCCCTTGGCAGCCAGAATGGGAACCCATGCAACCATGAAGATTCAAGGGCGCACTGTTGGGGGTCAGATGGGCATGCTCCTAACAGGATCCAACGCCAAGAGTATGGATCTGGGCACCACACCAGGTGACTGTGGCTGTCCCTACATTTACAAGAGGGGGAATGACTACGTAGTCATTGGAGTCCACACGGCTGCTGCCCGTGGAGGAAACACTGTCATATGTGCCACCCAGGGGAGCGAGGGAGAAGCCACACTTGAAGGCGGTGACAACAAGGGAACCTACTGTGGTGCACCAATCTTAGGTCCAGGAAGTGCCCCAAAGCTCAGCACCAAGACTAAGTTCTGGAGATCATCCACAGCACCACTCCCACCTGGTACCTATGAGCCAGCCTACCTTGGCGGCAAGGACCCCAGAGTCAAGGGTGGCCCTTCATTGCAACAAGTTATGAGGGACCAGCTGAAACCATTCACTGAGCCCAGGGGTAAACCACCAAAACCAAGTGTGTTAGAGGCTGCCAAGAAAACCATCATCAATGTTCTTGAACAAACAATTGATCCACCTCAAAAATGGTCATTCGCGCAGGCATGCGCATCCCTCGACAAGACCACTTCCAGTGGTCACCCGCACCACATGCGGAAAAACGACTGCTGGAATGGGGAGTCCTTTACAGGCAAATTGGCAGACCAGGCTTCCAAGGCCAACCTGATGTTCGAAGAGGGAAAGAACATGACCCCAGTCTACACAGGTGCGCTTAAGGACGAGCTGGTCAAGACTGACAAAATTTATGGCAAGATCAAAAAGAGGCTTCTCTGGGGCTCGGATCTGGCGACCATGGTCCGGTGCGCTCGAGCGTTCGGGGGCCTGATGGAGGAACTCAAAGCACATTGTGTCACACTACCCGTCAGAGTAGGTATGAACATGAATGAGGATGGTCCTATCATCTTTGAGAGACACTCCAGATACAAATACCATTATGATGCTGATTACTCCCGGTGGGACTCAACACAACAAAGAGCCGTGTTAGCAGCAGCCTTAGAAATCATGGTTAAGTTCTCCCCAGAACCGCATCTGGCCCAAAAGGTTGCAGAAGACCTTCTCTCTCCCAGCGTGATGGACGTAGGTGACTTCAAAATATCAATCAATGAGGGCCTCCCCTCCGGGGTGCCCTGCACCTCCCAATGGAATTCCATCGCCCACTGGCTCCTCACTCTCTGTGCACTTTCTGAGGTTACAAACCTGTCCCCTGACATCATCCAGGCCAACTCTCTCTTTTCCTTCTACGGTGATGATGAGATTGTGAGCACAGACATAAAGTTGGATCCAGAGAAGTTGACAGCAAAACTTAAGGAATACGGGTTGAAACCGACCCGCCCTGATAAGACTGAGGGACCCCTTGTTATCTCTGAGGACCTGAATGGCCTAACCTTCCTGCGGAGGACTGTGACCCGCGACCCAGCTGGCTGGTTTGGAAAGTTGGAACAGAGTTCAATACTCAGGCAAATGTATTGGACTAGGGGCCCCAACCATGAAGACCCATCTGAAACAATGATACCACACTCCCAAAGACCCATACAATTGATGTCTTTGCTGGGCGAGGCTGCACTCCACGGTCCAGCATTCTACAGCAAAATCAGCAAGCTGGTCATTGCAGAGCTGAAGGAAGGTGGCATGGATTTTTACGTGCCCAGACAAGAGCCAATGTTCAGATGGATGAGGTTCTCAGATCTGAGCACGTGGGAGGGCGATCGCAATCTGGCTCCCAGTTTTGTGAATGAAGATGGCGTCGAG

>KJ196299|II.12/II.12|2001|JP|Saitama/T18

ATGAAGATGGCGTCTAACGACGCTTCCGCTGCCGCTGCTGCTAACAGCAACAACGACACCGCAAAATCTTCAAGTGACGGAATGCTTTCTAGCATGGCTGTCACTTTTAAACGAGCCCTCGGGGCACGGCCTAAACAGCCTCCCCCGAGGGAAATACCACAAAGACCCCCACGACCACCCACCCCAGAACTGGTCAAAAAGATCCCCCCTCCTCCACCCAACGGGGAGGATGAACCAGTGGTTTCTTACAGCGTCAAAGATGGCGTTTCCGGCTTGCCTGAGCTTACCACTGTCAGGCAGCCGGGTGAAACCAACACGGCGTTCAGTGTTCCCCCACTCAACCAAAGGGAGAATAGGGACGCCAAGGAGCCACTAACTGGAACAATCCTGGAAATGTGGGACGGGGAGATCTACCATTACGGCCTGTATGTGGAACGAGGTCTTGTACTTGGTGTGCACAAACCACCGGCTGCCATCAGCCTCGCCAAGGTTGAATTAACACCACTCTCTCTGTTTTGGAGACCAGTGTATACACCACAGTATCTCATCTCTCCGGACACTCTCAGGAGACTGCACGGAGAGTCGTTTCCCTACACAGCCTTTGATAACAACTGCTATGCCTTCTGTTGTTGGGTCCTGGACCTAAACGACTCGTGGTTGTGCAGGAGAATGATCCAGAGGACAACTGGTTTCTTCAGGCCCTACCAAGACTGGAATAGGAAACCCCTCCCCACCATGGATGACTCCAAGTTGAAGAAGGTAGCTAACATATTTTTGTGCGCGCTATCTTCGCTATTCACTAGGCCCATCAAAGACATAATAGGAAAGTTGAGGCCTCTCAACATCCTTAACATCTTGGCTTCATGTGATTGGACTTTTGCAGGCATAGTGGAATCTTTGATTCTCTTGGCAGAGCTCTTTGGAGTTTTCTGGACACCCCCAGATGTGTCTGCGATGATCGCCCCTTTACTAGGTGACTACGAGCTGCAGGGGCCCGAGGACCTTGCAGTGGAACTCGTTCCAATAGTGATGGGGGGGATTGGTTTGGTGCTAGGATTCACCAAAGAGAAGATCGGGAAAATGTTGTCATCTGCTGCATCCACCTTAAGAGCTTGTAAAGACCTTGGTGCATACGGACTGGAAATTTTAAAATTGGTCATGAAGTGGTTCTTCCCAAAGAAAGAGGAAGCAAATGAGCTGGCTATGGTGAGATCCATCGAGGATGCGGTGCTGGACCTCGAGGCAATTGAAAATAACCACATGACTGCTCTCCTCAAAGACAAAGACAGCCTGGCAACCTATATGAGAACTCTTGACCTCGAGGAGGAGAAAGCCAGAAAGCTTTCAACCAAGTCTGCTTCACCTGATATCGTGGGCACAATCAACGCTCTCCTGGCGAGAATCGCCGCTGCACGCTCCCTGGTGCATCGGGCGAAAGAGGAGCTCTCCAGCAGACCAAGACCTGTTGTTGTGATGATATCAGGTAGGCCAGGGATAGGGAAAACCCACCTTGCCAGGGAATTGGCCAAGAAAATCGCAGCTTCTCTCACAGGGGACCAGCGTGTGGGTCTCATCCCGCGCAATGGTGTTGATCACTGGGACGCATATAAGGGAGAAAGAGTCGTTCTATGGGACGACTATGGAATGAGTAACCCCATCCACGACGCCCTCAGGTTACAAGAACTTGCTGACACCTGCCCCCTCACGCTAAATTGTGATAGGATTGAGAACAAAGGAAAGGTCTTTGACAGTGATGCCATAATCATCACCACTAACCTGACCAACCCAGCACCACTGGACTATGTCAATTTTGAAGCATGCTCGAGGCGTATCGACTTCCTCGTGTATGCAGATGCCCCTGAAGTCGAGAAGGCAAAACGTGATTTCCCAGGTCAACCTGACATGTGGAAGAACGCTTTCAGTCCTGACTTCTCGCACATAAAACTGATGCTGGCTCCGCAGGGTGGCTTCGACAAGAACGGAAACACCCCACATGGGAAAGGCGTCATGAAAACCCTCACCACTGGTTCCCTCATCGCACGAGCATCAGGGCTACTCCATGAGAGGTTAGATGAGTACGAGCTGCAGGGCCCAACCCCCACTACCTTCAACTTTGACCGCAATAAGGTGCTTGCGTTCAGACAGCTTGCTGCTGAAAACAAGTACGGGTTGATGGACACAATGAGAGTCGGAAGACAGCTCAAGGATGTCAGGACCATGCCAGAGCTCAAACAAGCACTCAAGAATATCTCAATCAAGAGTTGCCAGATAGTGTATGGTGGCTGCACCTATATGCTTGAGTCTGATGGCAAGGGTGATGTGAAAGTTGACAGAGTTCAGAATGCTACTGTGCAGACCAACAATGAACTGGCCGGTGCCCTACACCATCTTAGGTGTGCCAGGATTAGATACTATGTCAAGTGCATTCAGGAGGCCCTGTATTCCATCATCCAAATTGCTGGAGCTGCATTTGTCACCACGCGCATTGTCAAGCGCATGAACATACAAGACCTTTGGTCCAAGCCACAGGTGGAAGATACAGAGGAGACTGCTAGCAAGGATGGGTGCCCAAAACCCAAGGATGATGACGAGTTCGTTGTTTCATCCGACGACATCAAAACCGAGGGCAAGAAAGGAAAGAACAAGTCTGGCCGTGGTAAGAAGCACACGGCATTCTCAAGCAAAGGTCTCAGTGATGAGGAGTACGATGAGTACAAAAGAATCAGAGAAGAAAGAAACGGCAAGTACTCTATAGAGGAATACCTTCAGGACAGAGATAAGTATTATGAGGAGGTGGCCATCGCCAGGGCGACCGAAGAGGACTTCTGTGAAGAAGAAGAGGCCAAGATCCGACAAAGGATTTTTAGGCCAACAAGGAAACAACGCAAAGAGGAGAGGGCCTCTCTCGGCTTGGTCACAGGTTCTGAAATCAGGAAGAGGAACCCAGACGACTTCAAACCTAAAGGAAAGCTGTGGGCTGATGACGACAGGAGTGTTGACTACAATGAGAGACTCAATTTTGAAGCCCCACCAAGCATTTGGTCGAGGATAGTCAACTTTGGTTCAGGTTGGGGTTTTTGGGTTTCCCCCAGCCTGTTCATAACATCAACTCATGTCATACCCCAGGGCGCACAGGAGTTCTTTGGGGTTTCCATCAAGCAAATTCAGATACACAAATCGGGTGAATTCTGTCGCTTGAGGTTTCCAAAACCAATCAGAACTGATGTGACAGGCATGATCCTAGAAGAAGGTGCGCCCGAAGGGACCGTGGTCACATTACTCATCAAGAGACCAACTGGGGAACTCATGCCCTTGGCAGCCAGAATGGGAACCCATGCAACCATGAAGATACAAGGGCGCACTGTTGGGGGTCAAATGGGCATGCTCCTAACAGGATCTAACGCCAAGAGTATGGACCTGGGCACCACACCAGGTGACTGTGGCTGTCCCTACATTTACAAGAGAGGGAATGACTACATAGTCATTGGAGTCCACACGGCTGCTGCCCGTGGAGGAAACACCGTCATATGTGCCACCCAGGGGAGCGAGGGAGAAGCCACACTTGAAcGGCGGTGACAACAAGGGAACCTACTGCGGCGCACCAATCTTAGGTCCAGGAAGTGCCCCAAAGCTCAGCACCAAGACTAAGTTTTGGAGATCATCCACAGCACCACTCCCACCTGGTACCTATGAACCAGCCTACCTTGGCGGCAAGGACCCCAGAGTCAAAGGTGGCCCCTCATTGCAACAAGTTATGAGAGACCAGCTGAAACCATTCACTGAGCCCAGGGGTAAACCACCAAAACCAAGTGTGTTAGAGGCTGCCAAGAAAACCATCATCAATGTTCTTGAACAAACAATTGATCCACCTCAAAAATGGTCATTCGCGCAGGCATGCGCATCCCTCGACAAGACCACTTCCAGTGGCCACCCGCACCACATGCGGAAAAACGACTGCTGGAACGGGGAGTCCTTCACAGGCAAATTGGCAGACCAGGCTTCCAAGGCTAACCTGATGTTCGAAGAGGGAAAGAACATGACCCCAGTCTACACAGGTGCGCTTAAGGACGAGCTGGTCAAGACTGACAAAATTTATGGCAAGATCAAAAAGAGGCTTCTCTGGGGCTCGGATCTGGCGACCATGATCCGGTGTGCTCGAGCGTTTGGAGGCCTGATGGAGGAACTCAAAGCACATTGTGTCACACTACCCGTCAGAGTAGGTATGAATATGAATGAGGATGGCCCTATCATCTTTGAGAGACACTCCAGATATAAGTATCATTATGATGCTGATTACTCCCGGTGGGACTCAACACAACAAAGAGCCGTGTTAGCAGCAGCCTTAGAAATCATGGTTAAGTTCTCCCCAGAACCGAATCTGGCCCAAAAGGTTGCAGAAGACCTTCTCTCCCCCAGCGTGATGGACGTAGGTGACTTCAAAATATCAATCAATGAGGGCCTCCCCTCCGGGGTGCCCTGCACCTCCCAATGGAATTCCATCGCCCACTGGCTCCTCACCCTCTGTGCGCTTTCTGAGGTTACAAACCTGTCCCCTGACATTATCCAGGCTAATTCCCTCTTTTCCTTCTACGGTGATGATGAAATTGTGAGCACAGACATAAAATTGGACCCAGAGAAGTTGACAGCAAAACTTAAGGAATACGGGTTGAAACCGACCCGCCCTGACAAGACTGAGGGACCCCTTGTTATCTCTGAGGACCTGGATGGCCTAACCTTCCTGCGGAGGACTGTAACCCGCGACCCAGCTGGCTGGTTTGGAAAGCTGGAACAGAGCTCAATACTTAGGCAAATGTATTGGACTAGGGGCCCTAACCATGAAGACCCATCTGAAACAATGATACCACACTCCCAAAGACCCATACAATTGATGTCTTTGCTGGGCGAGGCTGCACTCCACGGCCCAGCATTCTACAGCAAAATCAGCAAGCTGGTCATTGCAGAGCTGAAGGAAGGTGGCATGGATTTTTACGTGCCCAGACAAGAGCCAATGTTCAGATGGATGAGGTTTTCAGATCTGAGCACGTGGGAGGGCGATCGCAATCTGGCTCCCAGTTTTGTGAATGAAGATGGCGTCGAG

# 129 GII.3 ORF2 sequences (Table S5):

>AB028244|-/II.3|<1999|JP|NLV36

TGCTCCATCTAATGATGGTGCCGCCGGCCTCGTCCCAGAGATCAACAATGAGGCAATGGCGCTAGACCCAGTGGCGGGTGCAGCGATAGCAGCACCCCTCACTGGTCAGCAAAACATAATTGATCCCTGGATTATGAATAATTTTGTGCAAGCACCTGGTGGTGAGTTTACAGTGTCCCCTAGGAATTCCCCTGGTGAAGTGCTTCTTAATTTGGAATTGGGCCCAGAAATAAACCCTTATTTGGCCCATCTTGCTAGAATGTATAATGGTTATGCAGGTGGATTTGAAGTGCAGGTGGTCCTGGCTGGGAATGCGTTCACAGCAGGAAAGATAATCTTTGCAGCTATACCCCCTAATTTTCCAATTGATAATCTGAGCGCAGCACAAATCACAATGTGCCCGCATGTGATTGTGGATGTCAGACAGTTGGAACCGGTCAACCTTCCGATGCCTGTCGTTCGCAACAATTTCTTTCATTACAATCAAGGGTCTGATTCGCGATTGCGCTTAATTGCAATGCTGTATACACCTCTTAGGGCAAATAATTCTGGAGATGATGTTTTCACTGTGTCTTGTAGAGTACTGACTAGGCCTAGCCCTGATTTTTCATTCAATTTCCTTGTCCCACCCACCGTGGAATCAAAGACAAAACCCTTTACCCTCCCTATTCTGACTATCTCTGAAATGTCCAATTCTAGGTTTCCAGTGCCGATTGAGTCTTTGCACACCAGCCCAACTGAGAATATTGTTGTCCAGTGCCAAAATGGGCGCGTCACTCTCGATGGTGAGTTGATGGGCACCACCCAACTCTTACCGAGTCGAATTTGTGCTTTTAGGGGCGTGCTCACCAGATCAACAAGCAGGGCCAGTGATCAGGCCGATATAGCAACCCCTAGGCTGTTTAATTATTATTGGCATGTACAATTGGATAATCTAAATGGGACCCCTTATGATCCTGCAGAAGACATACCAGGCCCCCTAGGGACACCAGACTTCCGGGGCAAGGTCTTTGGCGTGGCCAGCCAGAGAAACCCTGACAGCACAACTAGAGCACATGAAGCAAAGGTGGACACAACAGCTGGTCGTTTCACCCCAAAGTTGGGCTCATTAGAAATATCTACTGAGTCCAGTGACTTTGACCAAAACCAACCAACAAGATTCACCCCAGTTGGCATTGGGGTTGACAATGAGGCAGATTTTCAACAATGGTCTTTACCCGACTATTCTGGTCAGTTCACCCACAACATGAACTTGGCCCCAGCTGTTGCTCCCAACTTCCCTGGTGAGCAGCTTCTTTTCTTCCGCTCACAGTTACCATCTTCTGGTGGGCGATCCAACGGGGTCCTAGACTGTCTGGTCCCCCAGGAATGGGTCCAACACTTCTACCAGGAATCGGCCACCGCCCAAACACAAGTGGCCCTGGTTAGGTATGTCAACCCTGACACTGGTAGAGTGCTATTTGAGGCCAAGCTGCATAAATTAGGTTTTATGACTATAGCTAAGAATGGTGATTCTCCAATAACTGTTCCCCCAAATGGATATTTTAGGTTTGAATCTTGGGTGAACCCCTTTTATACGCTTGCCCCCATGGGAACTGGGAAT

>AB039781|II.3/II.3|1997|JP|U18

ATGAAGATGGCGTCGAATGACGCTGCTCCATCTAATGATGGTGCCGCCGGCCTCGTCCCAGAGATCAACAATGAGGCAATGGCGCTAGAGCCAGTGGCGGGCGCAGCGATAGCAGCGCCCCTCACTGGCCAGCAAAATATAATTGATCCCTGGATTATGAATAATTTTGTGCAAGCACCTGGTGGTGAGTTTACAGTGTCACCTAGGAATTCCCCTGGTGAAGTGCTTCTTAATTTGGAATTAGGTCCAGAAATAAACCCCTATTTGGCTCATCTTGCTAGAATGTACAATGGTTATGCAGGTGGATTTGAAGTGCAAGTGGTCCTAGCTGGAAATGCGTTTACGGCAGGAAAGGTTATCTTTGCAGCTATACCCCCTAATTTCCCTATTGACAATCTGAGCGCGGCACAGATCACAATGTGCCCGCACGTGATTGTGGATGTCAGGCAGTTGGAACCAATCAATCTCCCGATGCCTGATGTCCGCAACAATTTCTTTCATTATAATCAAGGTTCTGATTCAAGATTACGTTTGATTGCAATGTTGTATACACCTCTTAGGGCAAATAATTCTGGAGATGATGTTTTCACTGTGTCTTGTAGGGTGTTAACTAGGCCCAGCCCTGATTTCTCGTTCAATTTTCTTGTCCCACCCACTGTGGAATCAAAGACAAAGCTTTTTACCCTCCCCATTTTAACCATCTCTGAAATGTCCAATTCCAGGTTTCCAGTTCCAATTGACTCTTTACACACCAGCCCAACTGAGAATATAGTTGTCCAGTGCCAAAATGGGCGCGTCACTCTTGACGGTGAGTTAATGGGCGCCACCCAACTCTTACCGAGCCAAATATGTGCTTTCAGGGGCACACTCACTAGATCAACAAGCAGGGCCAGCGACCAAACCGACACACCAACCCCTAGGCTATTCAACCATCGTTGGCACATACAATTGGACAATCTAAATGGAACTCCCTACGACCCTGCAGAGGACATACCAGCTCCTTTGGGCACACCAGACTTCCGGGGTAAGGTCTTTGGCGTGGCCAGCCAGAGAAACCCCGACAGCACAACAAGGGCACATGAAGCAAAAGTGGACACAACATCTGGCCGCTTCACCCCAAAATTGGGCTCTTTAGAAATAACCACTGAGTCTGATGACCTTGACACAAACCAGTCAACAAAATTCACCCCAGTTGGCATCGGGGTTGACAATGAGGCAGAATTTCAGCAATGGTCCTTACCCAACTATTCTGGTCAGTTTACTCATAACATGAACTTAGCCCCAGCTGTCGCCCTCAATTTTCCTGGTGAACAGCTACTTTTCTTCCGATCACAGCTGCCATCCTCTGGTGGGCGGTCTAACGGGGTTCTAGACTGCTTGGTCCCCCAGGAATGGGTTCAACACTTTTACCAAGAATCAGCCCCCGCCCAAACGCAGGTGGCCCTGGTTAGGTATGTCAACCCTGACACTGGCAGAGTGCTATTTGAGGCTAAGCTACACAAATTGGGTTTTATGACTATAGCAAAGAATGGTGACTCCCCAATAACTGTCCCTCCAAATGGATACTTTAGATTTGAATCTTGGGTTAACCCCTTTTATACACTTGCCCCCATGGGAACTGGAAACGGGCGTAGAAGGATTCAATAA

>AB039782|II.3/II.3|1998|JP|U201

ATGAAGATGGCGTCGAATGACGCTGCTCCATCTAATGATGGTGCCGCCGGCCTCGTCCCAGAGATCAACAATGAGGCAATGGCGCTAGAGCCAGTGGCGGGCGCAGCGATAGCAGCGCCCCTCACTGGCCAGCAAAATATAATTGATCCCTGGATTATGAATAATTTTGTGCAAGCACCTGGTGGTGAGTTTACAGTGTCACCTAGGAATTCCCCTGGTGAAGTGCTTCTTAATTTGGAATTAGGTCCAGAAATAAACCCCTATTTGGCTCATCTTGCTAGAATGTACAATGGTTATGCAGGTGGATTTGAAGTGCAAGTGGTCCTAGCTGGAAATGCGTTTACAGCAGGAAAGGTTATCTTTGCAGCTATACCCCCTAATTTCCCTATTGACAATCTGAGCGCGGCACAGATCACAATGTGCCCGCACGTGATTGTGGATGTCAGGCAGTTGGAACCAATCAATCTCCCGATGCCTGATGTCCGCAACAATTTCTTTCATTATAATCAAGGTTCTGATTCAAGATTACGTTTGATTGCAATGTTGTATACACCTCTTAGGGCAAATAATTCTGGAGATGATGTTTTCACTGTGTCTTGTAGGGTGTTAACTAGGCCCAGCCCTGATTTCTCGTTCAATTTTCTTGTCCCACCCACTGTGGAATCAAAGACAAAGCTTTTTACCCTCCCCATTTTAACCATCTCTGAAATGTCCAATTCCAGGTTTCCAGTTCCAATTGACTCTTTACACACCAGCCCAACTGAGAATATAGTTGTCCAGTGCCAAAATGGGCGCGTCACTCTTGACGGTGAGTTAATGGGCACCACCCAACTCTTACCGAGCCAAATATGTGCTTTCAGGGGCACACTCACTAGATCAACAAGCAGGGCCAGCGACCAAGCCGACACACCAACCCCTAGGCTATTCAACCATCGTTGGCACATACAATTGGACAATCTAAATGGAACTCCCTACGACCCTGCAGAGGACATACCAGCTCCTTTGGGCACACCAGACTTCCGGGGCAAGGTCTTTGGCGTGGCCAGCCAGAGAAACCCCGACAGCACAACAAGGGCACATGAAGCAAAAGTGGACACAACATCTGGCCGCTTCACCCCAAAATTGGGCTCCTTAGAAATAACCACTGAGTCTGATGACTTTGACACAAACCAGTCAACAAAATTCACCCCAGTTGGCATCGGGGTTGACAATGAGGCAGAATTTCAGCAATGGTCCTTACCCAACTATTCTGGTCAGTTTACTCATAATATGAACTTAGCCCCAGCTGTCGCCCCCAATTTTCCTGGTGAACAGCTACTTTTCTTCCGATCACAGCTGCCATCCTCTGGTGGGCGGTCTAACGGGGTCCTAGACTGCTTGGTCCCCCAGGAATGGGTTCAACACTTTTACCAAGAATCAGCCCCCGCCCAAACGCAGGTGGCCCTGGTTAGGTATGTCAACCCTGACACTGGCAGAGTGCTATTTGAGGCTAAGCTACACAAATTGGGTTTTATGACTATAGCAAAGAATGGTGACTCCCCAATTACTGTCCCTCCAAATGGATACTTTAGATTTGAATCTTGGGTTAACCCCTTTTACACACTTGCCCCCATGGGAACTGGAAACGGGCGTAGAAGGATTCAATAA

>AB067541|II.3/II.3|<2001|JP|U18GII

ATGAAGATGGCGTCGAATGACGCTGCTCCATCTAATGATGGTGCCGCCGGCCTCGTCCCAGAGATCAACAATGAGGCAATGGCGCTAGAGCCAGTGGCGGGCGCAGCGATAGCAGCGCCCCTCACTGGCCAGCAAAATATAATTGATCCCTGGATTATGAATAATTTTGTGCAAGCACCTGGTGGTGAGTTTACAGTGTCACCTAGGAATTCCCCTGGTGAAGTGCTTCTTAATTTGGAATTAGGTCCAGAAATAAACCCCTATTTGGCTCATCTTGCTAGAATGTACAATGGTTATGCAGGTGGATTTGAAGTGCAAGTGGTCCTAGCTGGAAATGCGTTTACGGCAGGAAAGGTTATCTTTGCAGCTATACCCCCTAATTTCCCTATTGACAATCTGAGCGCGGCACAGATCACAATGTGCCCGCACGTGATTGTGGATGTCAGGCAGTTGGAACCAATCAATCTCCCGATGCCTGATGTCCGCAACAATTTCTTTCATTATAATCAAGGTTCTGATTCAAGATTACGTTTGATTGCAATGTTGTATACACCTCTTAGGGCAAATAATTCTGGAGATGATGTTTTCACTGTGTCTTGTAGGGTGTTAACTAGGCCCAGCCCTGATTTCTCGTTCAATTTTCTTGTCCCACCCACTGTGGAATCAAAGACAAAGCTTTTTACCCTCCCCATTTTAACCATCTCTGAAATGTCCAATTCCAGGTTTCCAGTTCCAATTGACTCTTTACACACCAGCCCAACTGAGAATATAGTTGTCCAGTGCCAAAATGGGCGCGTCACTCTTGACGGTGAGTTAATGGGCGCCACCCAACTCTTACCGAGCCAAATATGTGCTTTCAGGGGCACACTCACTAGATCAACAAGCAGGGCCAGCGACCAAACCGACACACCAACCCCTAGGCTATTCAACCATCGTTGGCACATACAATTGGACAATCTAAATGGAACTCCCTACGACCCTGCAGAGGACATACCAGCTCCTTTGGGCACACCAGACTTCCGGGGTAAGGTCTTTGGCGTGGCCAGCCAGAGAAACCCCGACAGCACAACAAGGGCACATGAAGCAAAAGTGGACACAACATCTGGCCGCTTCACCCCAAAATTGGGCTCTTTAGAAATAACCACTGAGTCTGATGACCTTGACACAAACCAGTCAACAAAATTCACCCCAGTTGGCATCGGGGTTGACAATGAGGCAGAATTTCAGCAATGGTCCTTACCCAACTATTCTGGTCAGTTTACTCATAACATGAACTTAGCCCCAGCTGTCGCCCTCAATTTTCCTGGTGAACAGCTACTTTTCTTCCGATCACAGCTGCCATCCTCTGGTGGGCGGTCTAACGGGGTTCTAGACTGCTTGGTCCCCCAGGAATGGGTTCAACACTTTTACCAAGAATCAGCCCCCGCCCAAACGCAGGTGGCCCTGGTTAGGTATGTCAACCCTGACACTGGCAGAGTGCTATTTGAGGCTAAGCTACACAAATTGGGTTTTATGACTATAGCAAAGAATGGTGACTCCCCAATAACTGTCCCTCCAAATGGATACTTTAGATTTGAATCTTGGGTTAACCCCTTTTATACACTTGCCCCCATGGGAACTGGAAACGGGCGTAGAAGGATTCAATAA

>AB067542|II.3/II.3|<2001|JP|U201GII

ATGAAGATGGCGTCGAATGACGCTGCTCCATCTAATGATGGTGCCGCCGGCCTCGTCCCAGAGATCAACAATGAGGCAATGGCGCTAGAGCCAGTGGCGGGCGCAGCGATAGCAGCGCCCCTCACTGGCCAGCAAAATATAATTGATCCCTGGATTATGAATAATTTTGTGCAAGCACCTGGTGGTGAGTTTACAGTGTCACCTAGGAATTCCCCTGGTGAAGTGCTTCTTAATTTGGAATTAGGTCCAGAAATAAACCCCTATTTGGCTCATCTTGCTAGAATGTACAATGGTTATGCAGGTGGATTTGAAGTGCAAGTGGTCCTAGCTGGAAATGCGTTTACAGCAGGAAAGGTTATCTTTGCAGCTATACCCCCTAATTTCCCTATTGACAATCTGAGCGCGGCACAGATCACAATGTGCCCGCACGTGATTGTGGATGTCAGGCAGTTGGAACCAATCAATCTCCCGATGCCTGATGTCCGCAACAATTTCTTTCATTATAATCAAGGTTCTGATTCAAGATTACGTTTGATTGCAATGTTGTATACACCTCTTAGGGCAAATAATTCTGGAGATGATGTTTTCACTGTGTCTTGTAGGGTGTTAACTAGGCCCAGCCCTGATTTCTCGTTCAATTTTCTTGTCCCACCCACTGTGGAATCAAAGACAAAGCTTTTTACCCTCCCCATTTTAACCATCTCTGAAATGTCCAATTCCAGGTTTCCAGTTCCAATTGACTCTTTACACACCAGCCCAACTGAGAATATAGTTGTCCAGTGCCAAAATGGGCGCGTCACTCTTGACGGTGAGTTAATGGGCACCACCCAACTCTTACCGAGCCAAATATGTGCTTTCAGGGGCACACTCACTAGATCAACAAGCAGGGCCAGCGACCAAGCCGACACACCAACCCCTAGGCTATTCAACCATCGTTGGCACATACAATTGGACAATCTAAATGGAACTCCCTACGACCCTGCAGAGGACATACCAGCTCCTTTGGGCACACCAGACTTCCGGGGCAAGGTCTTTGGCGTGGCCAGCCAGAGAAACCCCGACAGCACAACAAGGGCACATGAAGCAAAAGTGGACACAACATCTGGCCGCTTCACCCCAAAATTGGGCTCCTTAGAAATAACCACTGAGTCTGATGACTTTGACACAAACCAGTCAACAAAATTCACCCCAGTTGGCATCGGGGTTGACAATGAGGCAGAATTTCAGCAATGGTCCTTACCCAACTATTCTGGTCAGTTTACTCATAATATGAACTTAGCCCCAGCTGTCGCCCCCAATTTTCCTGGTGAACAGCTACTTTTCTTCCGATCACAGCTGCCATCCTCTGGTGGGCGGTCTAACGGGGTCCTAGACTGCTTGGTCCCCCAGGAATGGGTTCAACACTTTTACCAAGAATCAGCCCCCGCCCAAACGCAGGTGGCCCTGGTTAGGTATGTCAACCCTGACACTGGCAGAGTGCTATTTGAGGCTAAGCTACACAAATTGGGTTTTATGACTATAGCAAAGAATGGTGACTCCCCAATTACTGTCCCTCCAAATGGATACTTTAGATTTGAATCTTGGGTTAACCCCTTTTACACACTTGCCCCCATGGGAACTGGAAACGGGCGTAGAAGGATTCAATAA

>AB190457|II.a/II.3|<2004|JP|SN2000JA

ATGAAGATGGCGTCGAATGACGCCACTCCATCTAATGATGGTGCCGCCGGCCTCGTCCCAGAGATCAACAATGAGGCAATGGCGCTAGACCCAGTGGCGGGTGCAGCGATAGCAGCACCCCTCACTGGTCAGCAAAATATAATTGATCCCTGGATTATGAATAATTTTGTGCAAGCACCTGGTGGTGAGTTTACAGTATCCCCCAGGAATTCCCCTGGTGAAGTGCTTCTTAATTTGGAATTGGGCCCAGAAATAAACCCCTATTTGGCTCATCTTGCTAGAATGTATAATGGTTATGCAGGTGGATTTGAAGTGCAGGTAGTCCTGGCTGGAAATGCGTTTACAGCAGGAAAGATAATCTTTGCAGCTATACCCCCTAATTTTCCAATTGATAATCTGAGCGCAGCACAGATCACAATGTGCCCGCATGTGATTGTGGATGTCAGACAGTTGGAACCGGTCAACCTTCCGATGCCTGACGTTCGCAACAATTTCTTTCATTACAATCAAGGGTCTGATTCGAGATTGCGCTTAATCGCAATGCTGTATACACCTCTTAGGGCAAATAATTCTGGGGATGATGTTTTTACTGTGTCTTGTAGAGTGTTGACTAGGCCTAGCCCTGACTTTTCATTTAATTTTCTTGTGCCACCTACTGTGGAGTCAAAGACAAAGCCCTTCACCCTCCCTATTCTAACTATCTCTGAAATGTCCAATTCTAGGTTTCCAGTGCCGATTGATTCTCTGCACACCAGCCCAACTGAGAATATTGTTGTCCAGTGTCAAAATGGGCGTGTCACTCTTGATGGTGAGTTGATGGGCACCACCCAACTCTTACCGAGTCAAATCTGTGCTTTCAGGGGCGTGCTCACCAGATCAACAAGCAGGGCCAGTGATCAGGCCGACACAGCAACCCCTAGGTTGTTTAATTATTATTGGCACATACAATTGGATAATCTAAATGGGACTCCTTATGATCCTGCAGAAGACATACCAGGCCCCCTAGGGACACCAGATTTCCGGGGCAAAGTCTTTGGCGTGGCCAGCCAGAGAAACCCCGACAGCACAACTAGAGCACATGAAGCAAAGGTGGACACAACAGCTGGTCGTTTCACCCCAAAATTAGGCTCATTAGAAATATCCACTGAATCTGGTGACTTTGACCAAAACCAACCAACAAGATTCACCCCAGTTGGCATTGGGGTTGACAATGAGGCAGACTTTCAACAATGGTCTTTACCCGACTATTCTGGTCAGTTCACCCACAACATGAACTTGGCCCCAGCTGTTGCTCCCAACTTTCCTGGTGAGCAGCTCCTTTTCTTTCGCTCACAGTTACCATCTTCTGGTGGGCGATCTAACGGGATTCTAGACTGCCTGGTCCCCCAAGAATGGGTTCAGCACTTCTACCAAGAATCGGCCCCCGCCCAAACACAAGTGGCCCTGGTTAGGTATGTCAACCCTGACACTGGTAGAGTGTTATTTGAGGCCAAGCTGCACAAATTAGGTTTCATGACTATAGCTAAGAATGGTGATTCTCCAATAACTGTCCCTCCAAATGGATACTTTAGGTTTGAATCTTGGGTGAACCCCTTTTATACACTTGCCCCCATGGGAACTGGGAATGGGCGCAGAAGGATTCAATAA

>AB195226|-/II.3|1997|JP|Sinsiro/97

ATGAAGATGGCGTCGAATGACGCTGCTCCATCTAATGATGGTGCCGCCGGCCTCGTCCCAGAGATCAACAATGAGGCAATGGCGCTAGACCCAGTGGCGGGTGCAGCGATAGCAGCACCCCTCACTGGTCAGCAAAACATAATTGATCCCTGGATTATGAATAATTTTGTGCAAGCACCTGGTGGTGAGTTTACAGTGTCCCCTAGGAATTCCCCTGGTGAAGTGCTTCTTAATTTGGAACTGGGCCCAGAAATAAACCCTTATTTGGCCCATCTTGCTAGAATGTATAATGGTTATGCAGGTGGATTTGAAGTGCAGGTGGTCCTGGCTGGGAATGCGTTCACAGCAGGAAAGATAATCTTTGCAGCTATACCCCCTAATTTTCCAATTGATAATCTGAGCGCAGCACAAATCACAATGTGCCCGCATGTGATTGTGGATGTCAGACAGTTGGAACCGGTCAACCTTCCGATGCCTGACGTTCGCAACAATTTCTTTTATTACAATCAAGGGTCTGATTCGCGATTGCGCTTAATTGCAATGCTGTATACACCTCTTAGGGCAAATAATTCTGGAGATGATGTTTTCACTGTGTCTTGTAGAGTACTGACTAGGCCTAGCCCTGATTTTTCATTCAATTTCCTTGTCCCACCCACCGTGGAATCAAAGACAAAACCCTTTACCCTCCCTATTCTGACTATCTCTGAAATGTCCAATTCTAGGTTTCCAGTGCCGATTGAGTCTTTGCACACCAGCCCAACTGAGAATATTGTTGTCCAGTGCCAAAATGGGCGCGTCACTCTCGATGGTGAGTTGATGGGCACCACCCAACTCTTACCGAGTCAAATTTGTGCTTTTAGGGGCGTGCTCACCAGATCAACAAGCAGGGCCAGTGATCAGGCCGATACAGCAACCCCTAGGCTGTTTAATTATTATTGGCATGTACAATTGGATAATCTAAATGGGACCCCTTATGATCCTGCAGAAGACATACCAGGCCCCCTAGGGACACCAGACTTCCGGGGCAAGGTCTTTGGCGTGGCCAGCCAGAGAAACCCTGACAGCACAACTAGAGCACATGAAGCAAAGGTGGACACAACAGCTGGTCGTTTCACCCCAAAGTTGGGCTCATTAGAAATATCTACTGAGTCCAGTGACTTTGACCAAAACCAACCAACAAGATTCACCCCAGTTGGCATTGGGGTTGACAATGAGGCAGATTTTCAACAATGGTCTTTACCCGACTATTCTGGTCAGTTCACCCACAACATGAACTTGGCCCCAGCTGTTGCTCCCAACTTCCCTGGTGAGCAGCTCCTTTTCTTCCGCTCACAGTTACCATCTTCTGGTGGGCGATCCAACGGGGTCCTAGACTGTCTGGTCCCCCAGGAATGGGTCCAACACTTCTACCAGGAATCGGCCCCCGCCCAAACACAAGTGGCCCTGGTTAGGTATGTCAACCCTGACACTGGTAGAGTGCTATTTGGGGCCAAGCTGCATAAATTAGGTTTTATGACTATAGCTAAGAATGGTGATTCTCCAATAACTGTTCCCCCAAATGGATATTTTAGGTTTGAATCTTGGGTGAACCCCTTTTATACACTTGCCCCCATGGGAACTGGGAATGGGCGTAGAAGGATTCAATAA

>AB242256|II.21/II.3|2003|JP|Saga/5424

ATGAAGATGGCGTCGAATGACGCCACTCCATCTAATGATGGTGCCGCCGGCCTCGTCCCAGAGATCAACAATGAGGCAATGGCGCTAGACCCAGTGGCGGGTGCAGCGATAGCAGCACCCCTCACTGGTCAGCAAAATATAATTGATCCCTGGATTATGAATAATTTTGTGCAAGCACCTGGTGGTGAGTTTACAGTATCCCCTAGGAATTCCCCTGGTGAAGTGCTTCTCAATTTGGAATTGGGTCCAGAAATAAATCCCTATTTGGCCCATCTTGCTAGAATGTATAATGGTTATGCAGGTGGATTTGAAGTGCAGGTAGTCCTAGCTGGAAATGCGTTTACAGCAAGAAAGATAATCTTTGCAGCTATACCCCCTAATTTTCCAATTGATAATTTAAGCGCAGCACATATCACAATGTGCCCACATGTGATTGTGGATGTCAGACAGTTGGAACCGGTCAACCTCCCGATGCCTGACGTTCGCAACAACTTCTTTCATTACAATCAAGGGTCTGATTCGAGATTGCGCTTAATTGCAATGCTGTATACACCTCTTAGGGCAAATAATTCTGGGGATGATGTTTTCACTGTGTCTTGTAGAGTGCTGACTAGGCCTAGCCCTGACTTTTCATTTAATTTCCTTGTGCCACCTACCTTGGAGTCAAAGACAAAGCCCTTCACCCTCCCTATTCTGACTATCTCTGAAATGTCCAATTCTAGGTTTCCAGTGCCGATTGATTCTTTGCACACCAGCCCAACTGAGAATATTGTTGTCCAGTGCCAAAATGGGCGCGTCACTCTTGATGGTGAGTTGATGGGCACCACCCAACTCTTACCTAGTCAAATCTGTGCTTTTAGGGGCGTGCTCACCAGATCAACAAGCAGGGCCAGGGATCAGGCCGACACAGCAACCCCTAGGTTGTTTAATTATTATTGGCACATACAATTGGATAATCTAAATGGGACTCCTTATGATCCTGCAGAAGACATACCAGGCCCCCTAGGGACACCAGATTTCCGGGGCAAAGTCTTTGGCGTGGCCAGCCAGAGAAATCCCGACAGCACAACTAGAGCACATGAAGCAAAGGTGGACACAACAGCTGGTCGTTTCACCCCAAAACTAGGCTCATTAGAGATATCCACTGAATCTGATGACTTTGATCAAAACCAACCAACAAGATTCACCCCAGTTGGCATTGGGGTTGACAATGAGGCAGACTTTCAACAATGGTCTTTACCCGACTATTCTGGTCAGTTCACCCACAACATGAACTTAGCCCCAGCTGTTGCTCCCAACTTCCCTGGTGAGCAGCTCCTTTTCTTCCGCTCACAGTTACCATCCTCTGGTGGGCGATCCAACGGGATTCTAGACTGCCTGGTCCCCCAAGAATGGGTTCAGCACTTCTACCAAGAATCGGCTCCCTCTCAAACTCAAGTGGCCCTGGTTAGGTATGTCAACCCTGACACTGGTAGAGTATTATTTGAAGCCAAGCTGCACAAATTAGGTTTCACGACCATAGCTAAGAATGGTGACTCTCCAATAACTGTCCCTCCAAATGGATACTTTAGGTTTGAATCTTGGGTGAACCCCTTTTATACACTTGCCCCCATGGGAACTGGGAATGGGCGTAGAAGGAATCAATAA

>AB242257|-/II.3|2004|JP|Maizuru/5017

ATGAAGATGGCGTCGAATGACGCCACTCCATCTAATGATGGTGCCGCCGGCCTCGTCCCAGAGATCAACAATGAGGCAATGGCGCTAGATCCAGTGGCGGGTGCAGCGATAGCAGCACCCCTCACTGGTCAGCAAAATATAATTGATCCCTGGATTATGAATAACTTTGTGCAAGCACCTGGTGGTGAGTTTACAGTATCCCCTAGGAATTCCCCTGGTGAAGTGCTTCTTAATTTGGAATTGGGCCCAGAAATAAATCCCTATTTGGCCCATCTTGCTAGAATGTATAATGGTTATGCAGGTGGGTTTGAAGTGCAGGTAGTCCTAGCTGGAAATGCGTTTACAGCAGGAAAGATAATCTTTGCAGCTATACCCCCTTATTTTCCAATTGATAATTTAAGCGCAGCACAGATCACAATGTGCCCACATGTGATTGTGGATGTCAGACAGTTGGAACCGGTCAACCTCCCGATGCCTGACGTTCGCAATAACTTCTTTCATTATAATCAAGGGTCTGATTCGAGATTGCGCTTAGTTGCAATGCTGTATACACCTCTTAGGGCAAATAATTCTGGGGATGATGTTTTTACTGTGTCTTTGAGAGTGCTGACTAGGCCTAGCCCTGACTTTTCATTTAATTTCCTTGTGCCACCTACTGTGGAGTCAAAGACAAAACCCTTTACCCTCCCTATTCTGACTATCTCTGAAATGTCCAATTCTAGGTTTCCAGTGCCGATTGATTCTCTGCACACCAGCCCAACTGAGAATATTGTTGTCCAGTGCCAAAATGGGCGCGTCACTCTTGATGGTGAGTTGATGGGCACCACCCAACTCTTACCTAGTCAGATCTGTGCTTTCAGGGGCGTGCTCACCAGATCAACAAGCAGGGCCAGTGACCAGGCCGACACAGCAACCCCTAGGTTGTTTAATTATTATTGGCACATACAATTGGATAATCTAAACGGGACTCCTTATGATCCTGCAGAAGACATACCAGGCCCCCTAGGGACACCAGATTTCCGGGGCAAAGTCTTTGGCGTGGCCAGCCAGAGAAATCCCGACAGCACAACTAGAGCACATGAAGCAAAGGTGGACACAACAGCTGGTCGTTTTACCCCAAAACTAGGCTCATTAGAGATATCCACTGAATCTGATGACTTTGATCAAAACCAACCAACAAGATTCACCCCAGTTGGCATTGGGGTTGACCACGAGGCAGATTTCCAACAATGGTCTTTACCCGACTATTCTGGTCAGTTCACCCACAACATGAACTTAGCCCCAGCTGTTGCTCCCAACTTCCCTGGTGAGCAGCTCCTTTTCTTCCGCTCACAGTTACCATCCTCTGGTGGGCGATCCAACGGGATTCTAGACTGCCTGGTCCCCCAAGAATGGGTTCAGCACTTCTACCAAGAATCGGCCCCCGCCCAAACTCAAGTGGCCCTGGTTAGGTATGTCAACCCTGACACTGGTAGAGTATTGTTTGAGGCCAAGCTGCACAAATTAGGTTTCATGACTATAGCTAAGAATGGTGACTCTCCAATAACTGTCCCCCCAAATGGATATTTTAGGTTTGAATCTTGGGTGAACCCATTTTATACACTTGCCCCCATGGGAACTGGGAATGGGCGTAGAAGGATTCAATAA

>AB242258|II.21/II.3|2004|JP|78/04/Ru

ATGAAGATGGCGTCGAATGACGCCACTCCATCTAATGATGGTGCCGCCGGCCTCGTCCCAGAGATCAACAATGAGGCAATGGCGCTAGATCCAGTGGCGGGTGCAGCTATAGCAGCACCCCTCACTGGCCAGCAAAATATAATTGATCCCTGGATTATGAATAATTTTGTGCAAGCACCTGGTGGTGAGTTTACAGTATCCCCTAGGAATTCCCCTGGTGAAGTGCTTCTTGATTTGGAATTGGGTCCAGAAATAAATCCCTATTTGGCCCATCTTGCTAGAATGTATAATGGTTATGCAGGTGGATTTGAAGTGCAGGTAGTCCTAGCTGGGAATGCGTTCACAGCAGGAAAGATAATCTTTGCAGCTATACCCCCTAATTTTCCAATTGATAATCTAAGCGCAGCACAGATCACAATGTGCCCACATGTGATTGTGGATGTCAGACAGTTGGAACCGGTCAACCTCCCGATGCCTGACGTTCGCAACACTTTCTTTCATTACAATCAAGGGTCTGATTCGAGATTGCGCTTAATTGCAATGCTGTATACACCTCTTAGGGCAAATAATTCTGGGGATGATGTTTTTACTGTGTCTTGTAGAGTGCTGACTAGGCCTAGCCCTGACTTTTCATTTAATTTCCTTGTGCCACCCACTGTGGAGTCAAAGACAAAACCCTTCACCCTCCCTATTCTGACTATCTCTGAAATGTCCAATTCTAGGTTTCCAGTGCCGATTGATTCTCTGCACACCAGCCCAACCGAGAGTATTGTTGTCCAGTGCCAAAATGGACGCGTCACTCTTGATGGTGAGTTGATGGGCACCACCCAACTCTTACCTAGTCAAATCTGTGCTTTTAGGGGCGTGCTCACCAGATCAACAAGCAGGGCCAGTGACCAGGCCGACACAGCAACCCCCAGGTTGTTTAATTATTATTGGCACATACAATTGGATAATCTAAATGGGACTCCTTATGATCCTGCAGAAGACATACCAGGCCCCCTAGGGACACCAGACTTCCGGGGCAAAGTCTTTGGCGTGGCCAGCCAGAGAAACCCGGACAGCACAACTAGAGCACATGAGGCAAAGGTGGACACAACAACTGGTCGTTTCACCCCAAAACTAGGCTCATTAGAGATATCCACTGAATCTGATGACTTTGATCAAAACCAACCAACAAGATTCACCCCAGTTGGCATTGGGGTTGACCATGAGGCAGACTTTCAACAATGGTCTTTACCCGATTATTCTGGTCAGTTCACCCACAACATGAACTTAGCCCCAGCTGTTGCTCCTAACTTCCGTGGTGAGCAGCTCCTTTTCTTCCGCTCACAGTTACCATCCTCTGGTGGGCGATCCAACGGGACCCTAGACTGCCTGGTCCCCCAAGAATGGGTTCAGCACTTCTACCAAGAATCGGCCCCCGCCCAAACTCAAGTGGCCCTGGTCAGGTATGTCAACCCTGACACTGGCAGAGTATTATTTGAGGCCAAGCTGCACAAATTAGGTTTCATGACTATAGCTAAGAATGGTGACTCTCCAATAACTGTCCCTCCAAATGGATATTTTAGGTTTGAATCTTGGGTGAACCCCTTTTATACACTTGCCCCCATGGGAACTGGGAATGGGCGTAGAAGGATTCAATAA

>AB365435|II.21/II.3|2004|US|TCH04-577

ATGAAGATGGCGTCGAATGACGCCACTCCATCTAATGATGGTGCCGCCGGCCTCGTCCCAGAGATCAACAATGAGGCAATGGCGCTAGATCCAGTGGCGGGTGCAGCGATAGCAGCACCCCTCACTGGCCAGCAAAATATAATTGATCCCTGGATTATGAATAATTTTGTGCAAGCACCTGGTGGTGAGTTTACAGTATCCCCTAGGAATTCCCCTGGTGAAGTGCTTCTTAATTTGGAATTGGGCCCAGAAATAAATCCCTATTTGGCACATCTCGCTAGAATGTATAATGGTTATGCAGGTGGATTTGAAGTGCAGGTAGTCCTAGCTGGAAATGCGTTTACAGCAGGAAAGATAATCTTTGCAGCTATACCCCCTAATTTCCCAATTGATAATCTAAGCGCACGACAGATTACAATGTGCCCACATGTGATTGTGGATGTCAGACAGTTGGAACCAGTCAACCTCCCGATGCCTGACGTTCGCAACAATTTCTTTCATTATAACCAAGGATCTGATTCGAGATTGCGCTTAATTGCAATGCTGTACACACCTCTTAGGGCAAATAATTCTGGGGATGATGTTTTTACTGTGTCTTGTAGAGTGCTGACTAGGCCTAGCCCTGACTTCTCATTCAATTTCCTTGTGCCACCCACTGTGGAGTCAAAGACAAAACCCTTCACCCTCCCTATTCTGACTATCTCTGAAATGTCCAATTCTAGGTTTCCAGTGCCGATTGACTCTCTGCACACCAGCCCAACTGAGAATATTGTTGTCCAGTGCCAAAATGGGCGCGTCACTCTTGATGGTGAGCTGATGGGCACCACCCAACTCTTACCTAGTCAAATCTGTGCTTTCATGGGCGTGCTCACCAGGTCAACAAGCAGGGCCAGTGATCAGGCCGACACAGCAACCCCTAGGTTGTTTAATTATTATTGGCATATACAATTGGATAATCCAAATGGGACTCCTTATGATCCTGCAGAAGACATACCAGGCCCCCTAGGGACACCAGATTTCCGGGGCAAAGTCTTTGGCGTGGCCAGCCAGAGAAACCCCGACAGCACAACTAGAGCACATGAAGCAAAGGTGGACACAACAGCTGGTCGTTTCACCCCAAAACTAGGCTCATTAGAGATATCCACTGAATCTGATGACTTTCATCAAAACCAACCAACAAGATTCACCCCAGTTGGCATTGGGGTTGACAATGAAGCAGACTTTCAACAGTGGTCTTTACCCGACTATTCTGGTCAGTTCACCCACAACATGAACTTAGCCCCAGCTGTTGCTCCCAACTTCCCTGGAGAGCAGCTCCTTTTCTTCCGCTCACAGTTACCATCCTCTGGTGGGCGATCCAACGGGATTCTAGACTGCCTGGTCCCTCAAGAGTGGGTTCAGCACTTCTACCAAGAATCGGCCCCCTCTCAAACTCAAGTGGCCCTGGTTAGGTATGTCAACCCTGACACTGGCAGAGTATTATTTGAGGCCAAGCTGCACAAATTAGGTTTCATGACTATAGCTAAGAATGGTGACTCTCCAATAACTGTCCCTCCAAATGGATACTTTAGGTTTGAATCTTGGGTGAACCCATTTTACACACTTGCCCCCATGGGAACTGGGAATGGGCGTAGAAGGATTCAATAA

>AB385626|-/II.3|2006|NL|RotterdamP1D0

ATGAAGATGGCGTCGAATGACGCCGCTCCATCTAATGATGGTGCCGCCGGCCTCGTCCCAGAGATCAACAATGAGGCAATGGCGCTAGAGCCAGTGGCGGGTGCAGCGATAGCAGCACCCCTCACTGGTCAGCAAAATATAATTGATCCCTGGATTATGAATAATTTTGTGCAAGCACCTGGTGGTGAGTTTACAGTATCCCCTAGAAATTCCCCTGGTGAAGTTCTTCTTAATTTGGAACTGGGCCCAGAAATAAATCCCTATTTGGCCCATCTTGCTAGAATGTATAATGGTTATGCAGGTGGATTTGAAGTGCAGGTGGTCCTAGCTGGAAATGCGTTTACAGCAGGAAAGATAATCTTTGCAGCTATTCCCCCCAATTTTCCAATTGACAATCTAAGTGCAGCACAGATCACAATGTGTCCACATGTGATTGTGGATGTCAGACAGTTGGAACCAGTCAACCTCCCGATGCCTGACGTTCGCAACAACTTCTTTCATTACAACCAAGGGTCTGATTCGAGATTGCGCCTAATTGCAATGCTATACACACCTCTTAGGGCAAATAATTCTGGGGATGATGTTTTTACTGTGTCTTGCAGAGTGCTAACTAGACCTAGCCCTGACTTCTCATTTAATTTCCTTGTGCCACCCACTGTGGAGTCAAAGACAAAACCCTTTTCCCTCCCTATTCTGACTATCTCTGAAATGTCCAATTCTAGGTTCCCAGTACCAATTGATTCTCTGCACACCAGCCCTACTGAGAATATTGTTGTCCAGTGCCAGAATGGGCGCGTCACCCTTGATGGTGAGTTGATGGGCACCACCCAACTCTTACCTAGCCAAATTTGTGCTTTCAGGGGCGTGCTCACCAGATCAACAAGCAGGGCCAGTGACCAGGCCGATACAGCAACCCCTAGATTGTTTAATTATTATTGGCATATACAATTGGATAATCTAAATGGAACTCCTTATGATCCTGCAGAAGACATACCAGGCCCCCTAGGGACACCAGATTTCCGGGGCAAAGTCTTTGGCGTGGCCAGCCAGAGAAATCCTGATGCCACGACTAGGGCACATGAAGCAAAGATAGACACAGCATCTGGCCGTTTCACCCCAAAACTAGGCTCATTAGAGATATCCACTGAATCTGATGATTTTGACCAAAACAAACCAACAAGATTCACCCCAGTTGGCATTGGGGTTGACCATGAGGAAGACTTTCAACAATGGACTCTACCCGACTATGCTGGCCAGTTCACCCACAACATGAACTTAGCCCCAGCTGTTGCTCCCAACTTCCCTGGTGAGCAGCTCCTTTTCTTCCGCTCACAGTTGCCATCTTCTGGTGGGCGATCCAACGGGATTCTAGACTGCCTGGTCCCCCAAGAATGGGTACAGCACTTCTACCAAGAATCAGCCCCCTCCCAATCTCAAGTGGCCCTGGTTAGGTATATCAACCCTGACACTGGTAGAGTGTTATTTGAGGCCAAGCTGCACAAATTAGGTTTCATGACTATAGCCAAGAATGGTGACTCTCCAATAACTGTCCCTCCGAATGGATACTTTAGGTTTGAATCTTGGGTGAACCCCTTTTATACACTTGCCCCCATGGGAACTGGGAATGGGCGTAGAAGGATTCAATAA

>AB385627|-/II.3|2006|NL|RotterdamP1D88

ATGAAGATGGCGTCGAATGACGCCGCTCCATCTAATGATGGTGCCGCCGGCCTCGTCCCAGAGATCAACAATGAGGCAATGGCGCTAGAGCCAGTGGCGGGTGCAGCGATAGCAGCACCCCTCACTGGTCAGCAAAATATAATTGATCCCTGGATTATGAATAATTTTGTGCAAGCACCTGGTGGTGAGTTTACAGTATCCCCTAGAAATTCCCCTGGTGAAGTTCTTCTTAATTTGGAACTGGGCCCAGAAATAAATCCCTATTTGGCCCATCTTGCTAGAATGTATAATGGTTATGCAGGTGGATTTGAAGTGCAGGTGGTCCTAGCTGGAAATGCGTTTACAGCAGGAAAGATAATCTTTGCAGCTATTCCCCCCAATTTTCCAATTGACAATCTAAGTGCAGCACAGATCACAATGTGTCCACATGTGATTGTGGATGTCAGACAGTTGGAACCAGTCAACCTCCCAATGCCTGACGTTCGCAACACCTTCTTTCATTACAACCAAGGGTCTGATTCGAGATTGCGCCTAATTGCAATGCTATACACACCTCTTAGGGCAAATAATTCTGGGGATGATGTTTTTACTGTGTCTTGCAGAGTGCTAACTAGACCTAGCCCTGACTTCTCATTTAATTTCCTTGTGCCACCCACTGTGGAGTCAAAGACAAAACCCTTTTCCCTCCCTATTCTGACTATCTCTGAAATGTCCAATTCTAGGTTCCCAGTACCAATTGATTCTCTGCACACCAGCCCTACTGAGAATATTGTTGTCCAGTGCCAGAATGGGCGCGTCACCCTTGATGGTGAGTTGATGGGCACCACCCAACTCTTACCTAGCCAAATTTGTGCTTTCAGGGGCGTGCTCACCAGATCAACAAGCAGGGCCAGTGACCAGGCCGATACAGCAACCCCTAGATTGTTTGATTATTATTGGCATATACAATTGGACAATCTAAATGGAACTCCCTATGATCCTGCAGAAGACATACCAGGCCCCCTAGGGACACCAGATTTCCGGGGCAAAGTCTTTGGAGTGGCCAGCCAGAGAAATCCTGATACCACGACTAGGGCACATGAAGCAAAAATAGACACAGCATCTGGCCGTTTCACCCCAAAACTAGGCTCATTAGAGATATCCACTGAATCTGATGATTTTGACCAAAACAAACCAACAAGATTCACCCCAGTTGGCATTGGGGTTGACCATGAGGAAGACTTTCAACAATGGACTCTACCCAACTATGCTGGCCAGTTCACCCACAACATGAACTTAGCCCCAGCTGTTGCTCCCAACTTCCCTGGTGAGCAGGTCCTTTTCTTCCGCTCACAGTTGCCATCTTCTGGTGGGCGATCCAACGGGATTCTAGACTGCCTGGTCCCCCAAGAATGGGTACAGCACTTCTACCAAGAATCAGCCCCCTCCCAATCTCAAGTGGCCCTGGTTAGGTATGTCAACCCTGACACTGGTAGAGTGTTATTTGAGGCCAAGCTGCACAAATTAGGTTTCATGACTATAGCCAGGAATGGTGACTCTCCAATAACTGTCCCTCCGAATGGATACTTTAGGTTTGAATCTTGGGTGAACCCCTTTTATACACTTGCCCCCATGGGAACTGGGAATGGGCGTAGAAGGATTCAATAA

>AB385634|-/II.3|2005|NL|RotterdamP5D0

ATGAAGATGGCGTCGAGTGACGCCACTCCATCTAATGATGGTGCCGCCGGCCTCGTCCCAGAGATCAACAATGAGGCAATGGCGCTAGATCCAGTGGCGGGTGCAGCGATAGCAGCACCCCTCACTGGTCAGCAAAATATAATTGATCCCTGGATTATGAATAATTTTGTGCAAGCACCTGGTGGTGAGTTTACAGTGTCCCCTAGGAATTCCCCTGGTGAAGTGCTTCTTAATTTGGAATTGGGCCCAGAAATAAATCCCTATTTGGCTCATCTTGCTAGAATGTATAATGGTTATGCAGGTGGATTTGAAGTGCAGGTAGTCCTAGCTGGAAATGCGTTTACAGCAGGAAAGATAATCTTTGCAGCTATACCCCCTAACTTTCCAATTGATAATTTGAGTGCAGCACAGATCACAATGTGCCCACATGTAATTGTGGATGTCAGACAGTTGGAACCGGTCAACCTCCCGATGCCTGACGTTCGCAACAACTTCTTTCATTATAATCAAGGGTCTGATTCGAGATTGCGCTTAATTGCAATGCTGTATACACCTCTTAGGGCAAATAATTCTGGGGATGATGTTTTTACTGTGTCTTGTAGAGTGCTGACTAGGCCTAGCCCTGACTTTTCATTTAATTTCCTTGTGCCACCTACTGTGGAGTCAAAGACAAAACCCTTCACCCTCCCTATTCTGACTATCTCTGAAATGTCCAATTCTAGGTTTCCAGTGCCGATCGACTCTCTGCACACCAGCCCAACTGAGAATATTGTTGTCCAGTGCCAAAATGGGCGCGTCACTCTTGATGGTGAGTTGATGGGCACCACCCAACTCTTACCTAGTCAAATTTGTGCTTTTAGGGGCGTGCTCACCAGATCAACAAGCAGGGCCAGTGACCAAGCCGATACAGCAACCCCTAGATTGTTTAATTATTATTGGCACATACAATTGGATAATCTAAATGGGACTCCTTATGACCCTGCAGAAGACATACCAGGCCCCCTGGGGACACCAGATTTTCGGGGCAAGGTCTTTGGCGTGGCCAGCCAGAGAAATCCCGACAGTACAACTAGAGCACATGAGGCAAAGGTGGACACAACAGCTGGTCGTTTCACCCCAAAACTAGGCTCATTAGAGATATCCACTGAATCTGATGACTTTGATCAAAACCAACCAACAAGATTCACCCCAGTTGGCATTGGGGTTGACCATGAGGCAGACTTCCAACAATGGTCTTTACCCGACTATTCTGGTCAGTTCACCCACAACATGAACTTAGCCCCAGCTGTTGCTCCCAACTTTCCTGGTGAACAACTCCTTTTCTTCCGCTCACAGCTACCATCCTCTGGTGGGCGATCCAACGGGATTCTAGACTGCTTGGTCCCCCAAGAATGGGTTCAGCACTTCTACCAAGAATCGGCCCCCGCCCAAACTCAAGTGGCCCTGGTTAGGTATGTCAACCCTGACACTGGTAGAGTATTATTTGAGGCCAAGCTGCACAAATTAGGTTTCATGACTATAGCTAAGAATGGTGATTCTCCAATAACTGTCCCTCCAAATGGGTACTTTAGGTTTGAATCTTGGGTGAACCCCTTTTATACTCTTGCCCCCATGGGAACTGGGAATGGGCGTAGAAGGATTCAATAA

>AB385641|-/II.3|2006|NL|RotterdamP8D0

ATGAAGATGGCGTCGAATGACGCCGCTCCATCTAATGATGGTGCCGCCGGCCTCGTCCCAGAGATCAACAATGAGGCAATGGCGCTAGAGCCAGTGGCGGGTGCAGCGATAGCAGCACCCCTCACTGGTCAGCAAAATATAATTGATCCCTGGATTATGAATAATTTTGTGCAAGCACCTGGTGGTGAGTTTACAGTGTCCCCTAGAAATTCCCCTGGTGAAGTCCTTCTTAATTTGGAATTGGGCCCAGAAATAAATCCCTATTTGGCCCATCTTGCTAGAATGTATAATGGTTATGCAGGTGGATTTGAAGTGCAGGTGGTCCTAGCTGGAAATGCGTTTACAGCAGGAAAGATAATCTTTGCAGCTATCCCCCCCAATTTTCCAATTGACAATCTAAGTGCAGCACAGATCACAATGTGCCCACATGTGATTGTGGATGTCAGACAGTTGGAACCAGTCAACCTCCCGATGCCTGACGTTCGCAACAACTTCTTTCATTACAATCAAGGGTCTGATTCGAGATTGCGCCTAATTGCAATGCTATACACACCTCTTAGGGCAAATAATTCTGGGGATGATGTTTTTACTGTGTCTTGCAGAGTGCTAACTAGACCTAGTCCTGACTTCTCATTTAATTTCCTTGTGCCACCTACTGTGGAGTCAAAGACAAAACCCTTCTCCCTCCCTATTCTGACTATCTCTGAAATGTCCAATTCTAGGTTCCCAGTACCAATTGATTCTCTGCACACCAGTCCTACTGAGAATATTGTTGTCCAGTGCCAGAATGGGCGCGTCACCCTTGATGGTGAGTTGATGGGCACCACCCAACTCTTACCTAGCCAAATCTGTGCTTTCAGGGGCGTTCTCACCAGATCAACAAGCAGGGCCAGTGACCAGGCCGATACAGCAACCCCTAGATTATTTAATTATTATTGGCATATACAATTGGATAATCTAAATGGAACTCCTTATGATCCTGCAGAAGACATACCAGGCCCCCTAGGGACACCAGATTTCCGGGGCAAAGTCTTTGGCGTGGCCAGCCAGAGAAATCCTGATACCACGACTAGGGCACATGAAGCAAAGATAGACACAACATCTGGCCGTTTCACCCCAAAACTAGGCTCACTAGAGATATCCACTGAATCTGATGATTTTGATCAAAACAAACCAACAAGATTCACCCCAGTTGGCATTGGGGTTGACCGTGAGGTAGACTTTCAACAATGGGCTCTACCCGACTACGCTGGTCAGTTCACCCACAACATGAACTTAGCCCCAGCTGTTGCTCCCAACTTCCCTGGTGAACAGCTCCTTTTCTTCCGCTCACAGTTGCCATCTTCTGGTGGGCGATCCAACGGGATTTTAGACTGCCTGGTCCCCCAAGAATGGGTACAGCACTTCTACCAAGAATCAGCCCCCTCCCAATCTCAAGTGGCCCTGGTTAGGTATATCAACCCTGACACTGGTAGAGTGTTATTTGAGGCCAAGCTGCATAAATTAGGTTTCATGACTATAGCCAAGAATGGTGACTCTCCAATAACTGTCCCTCCAAATGGATACTTTAGGTTTGAATCTTGGGTGAACCCCTTTTATACACTTGCCCCCATGGGAACTGGGAATGGGCGTAGAAGGATTCAATAA

>AB385642|-/II.3|2006|NL|RotterdamP8D31

ATGAAGATGGCGTCGAATGACGCCGCTCCATCTAATGATGGTGCCGCCGGCCTCGTCCCAGAGATCAACAATGAGGCAATGGCGCTAGAGCCAGTGGCGGGTGCAGCGATAGCAGCACCCCTCACTGGTCAGCAAAATATAATTGATCCCTGGATTATGAATAATTTTGTACAAGCACCTGGTGGTGAGTTTACAGTGTCCCCTAGAAATTCCCCTGGTGAAGTCCTTCTTAATTTGGAATTGGGCCCAGAAATAAATCCCTATTTGGCCCATCTTGCTAGAATGTATAATGGTTATGCAGGTGGATTTGAAGTGCAGGTGGTCCTAGCTGGAAATGCGTTTACAGCAGGAAAGATAATCTTTGCAGCTATCCCCCCCAATTTTCCAATTGACAATCTAAGTGCAGCACAGATCACAATGTGCCCACATGTGATTGTGGATGTCAGACAGTTGGAACCAGTCAACCTCCCGATGCCTGACGTTCGCAACAACTTCTTTCATTACAATCAAGGGTCTGATTCGAGATTGCGCCTAATTGCAATGCTATACACACCTCTTAGGGCAAATAATTCTGGGGATGATGTTTTTACTGTGTCTTGCAGAGTGCTAACTAGACCTAGTCCTGACTTCTCATTTAATTTCCTTGTGCCACCTACTGTGGAGTCAAAGACAAAACCCTTCTCCCTCCCTATTCTGACTATCTCTGAAATGTCCAATTCTAGGTTCCCAGTACCAATTGATTCTCTGCACACCAGTCCTACTGAGAATATTGTTGTCCAGTGCCAGAATGGGCGCGTCACCCTTGATGGTGAGTTGATGGGCACCACCCAACTCTTACCTAGCCAAATCTGTGCTTTCAGGGGCGTTCTCACCAGATCAACAAGCAGGGCCAGTGACCAGGCCGATACAGCAACCCCTAGATTATTTAATTATTATTGGCATATACAATTGGATAATCTAAATGGAACTCCTTATGATCCTGCAGAAGACATACCAGGCCCCCTAGGGACACCAGATTTCCGGGGCAAAGTCTTTGGCGTGGCCAGCCAGAGAAATCCTGATACCACGACTAGGGCACATGAAGCAAAGATAGACACAACATCTGGCCGTTTCACCCCAAAACTAGGCTCACTAGAGATATCCACTGAATCTGATGATTTTGATCAAAACAAACCAACAAGATTCACCCCAGTTGGCATTGGGGTTGACCATGAGGTAGACTTTCAACAATGGGCTCTACCCGACTACGCTGGTCAGTTCACCCACAACATGAACTTAGCCCCAGCTGTTGCTCCCAACTTCCCTGGTGAACAGCTCCTTTTCTTCCGCTCACAGTTGCCATCTTCTGGTGGGCGATCCAACGGGATTTTAGACTGCCTGGTCCCCCAAGAATGGGTACAGCACTTCTACCAAGAATCAGCCCCCTCCCAATCTCAAGTGGCCCTGGTTAGGTATATCAACCCTGACACTGGTAGAGTGTTATTTGAGGCCAAGCTGCATAAATTAGGTTTCATGACTATAGCCAAGAATGGTGACTCTCCAATAACTGTCCCTCCAAATGGATACTTTAGGTTTGAATCTTGGGTGAACCCCTTTTATACACTTGCCCCCATGGGAACTGGGAATGGGCGTAGAAGGATTCAATAA

>AB629943|II.12/II.3|2010|JP|Tokyo/10-1105

ATGAAGATGGCGTCGAATGACGCCGCTCCATCTAATGATGGTGCCGCCGGCCTCGTCCCAGAGATCAACAATGAGGCAATGGCGCTAGAACCAGTGGCGGGTGCAGCGATAGCAGCACCCCTCACTGGCCAGCAAAATATAATTGATCCCTGGATTATGAATAATTTTGTGCAAGCACCTGGTGGTGAGTTTACAGTGTCTCCTAGGAATTCCCCTGGTGAAGTGCTTCTCAATTTGGAATTGGGCCCAGAAATAAATCCCTATTTGGCCCATCTTGCTAGAATGTATAATGGTTATGCAGGTGGGTTTGAAGTGCAGGTAGTCCTAGCTGGAAATGCGTTTACAGCAGGAAAGATAATTTTTGCAGCTATACCCCCTAACTTCCCAATTGACAATCTAAGCGCAGCACAGATCACAATGTGCCCACATGTGATTGTGGATGTCAGACAGTTGGAACCGGTCAACCTTCCGATGCCTGACGTTCGCAACAACTTCTTCCATTACAACCAAGGGTCTGATTCGAGATTGCGCTTAGTTGCAATGCTGTATACACCTCTTAGGGCAAATAATTCTGGGGATGATGTTTTTACTGTGTCTTGTAGAGTGCTAACTAGGCCTAGCCCTGACTTTTCATTTAACTTCCTTGTGCCACCCACTGTGGAGTCAAAGACAAAACCCTTCACCCTCCCTATTTTGACTATCTCTGAAATGTCTAATTCTAGGTTTCCAGTGCCGATTGATTCTCTGCACACCAGCCCAACTGAGAATATTGTTGTCCAGTGCCAAAATGGGCGCGTCACTCTTGATGGTGAGTTGATGGGCACCACCCAACTCTTACCTAGTCAAATCTGTGCTTTTAGGGGCGTGCTCACAAGATCAACAAGCAGGGCCAGTGACCAGGCCGACACAGCAACCCCTAGATTGTTTAATTATTATTGGCACATACAATTGGCTAATCTAAATGGGACTCCTTATGATCCTGCAGAAGACATACCAGGCCCCCTGGGAACACCAGACTTCCGGGGCAAAGTCTTTGGCGTGGCCTGCCAGAGAAATCCCGACTGTACAACTAGAGCACATGAAGCAAAGGTGGACACAACAGCTGGTCGCTTCACCCCAAAACTAGGCTCATTGGAGATATCTACTGAATCTGGTGACTTTGACCAAAACCAACCAACAAGATTCACCCCAGTTGGCATTGGGGTTGACCACGAGGCAGACTTCCAACAATGGTCCTTACCCGATTATTCTGGTCAGTTCACTCACAACATGAACTTAGCCCCAGCTGTTGCTCCCAATTTCCCTGGTGAGCAGCTCCTTTTCTTCCGCTCACAGTTACCATCTTCTGGTGGGCGATCCAATGGGATTCTAGACTGCCTGGTCCCCCAAGAATGGGTTCAGCACTTCTACCAAGAATCGGCCCCCGCCCAAACCCAGGTGGCCCTGGTTAGATATGTCAACCCTGACACTGGTAGAGTGTTGTTTGAGGCCAAGCTGCACAAATTAGGCTTCATGACTATAGCTAAGAATGGTGACTCTCCAATAACTGTCCCCCCAAATGGATACTTTAGGTTTGAATCTTGGGTGAACCCATTTTATACACTTGCCCCCATGGGAACTGGGAATGGGCGTAGAAGGATTCAATAA

>AB758450|-/II.3|2009|JP|GII.3/2009/Miyagi

ATGAAGATGGCGTCGAATGACGCTGCTCCATCTAATGATGGTGCCGCCGGCCTCGTCCCAGAGATCAACAATGAGGCAATGGCGCTAGACCCAGTGGCGGGTGCAGCGATAGCAGCACCCCTCACTGGTCAGCAAAATATAATTGATCCCTGGATTATGAATAATTTTGTACAAGCACCTGGTGGTGAGTTTACAGTATCCCCTAGGAATTCCCCTGGTGAAGTGCTTCTTAATTTGGAATTGGGCCCAGAAATAAACCCCTATTTGGCCCATCTTGCTAGAATGTATAATGGTTATGCAGGTGGATTTGAAGTGCAAGTAGTCCTGGCTGGAAATGCGTTTACAGCAGGAAAGATAATCTTTGCAGCTATACCCCCTAATTTTCCAATTGATAACCTGAGCGCAGCACAGATCACAATGTGCCCGCATGTGATTGTGGATGTCAGACAATTGGAACCGGTCAATCTTCCGATGCCTGACGTTCGCAACAATTTCTTTCATTATAATCAAGGGTCTGATTCGAGATTGCGCTTAATTGCAATGCTGTACACACCTCTTAGGGCAAATAATTCTGGGGATGATGTTTTTACTGTGTCATGCAGAGTGCTGACTAGGCCTAGCCCTGATTTTTCATTTAATTTCCTTGTGCCACCCACTGTGGAGTCAAAGACAAAGCCCTTCACCCTTCCTATTCTGACTATCTCTGAAATGTCCAATTCTAGGTTTCCAGTTCCGATTGATTCTCTGCACACAAGCCCAACTGAGAATATTGTTGTCCAGTGCCAAAACGGGCGCGTCACTCTTGACGGTGAGTTGATGGGAACCACCCAACTCTTACCGAGTCAGATCTGTGCTTTCAGGGGCGTACTCACCCGATCAACAAGCAGGGCCAGTGATCAGGCCGACACAGCAACCCCTAGGTTGTTTAATTATTACTGGCACATACAATTGGATAATCTAAATGGAACACCTTATGACCCTGCAGAAGACATACCAGGCCCCCTAGGGACACCAGATTTTCGGGGCAAAGTCTTTGGCGTGGCCAGCCAAAGAAACCCCGACAGCACAACTAGGGCACATGAAGCAAAGGTGGATACAACATCTGGCCGTTTTACCCCAAAATTAGGCTCATTAGAAATATCCACTGAATCTGGTGACTTTGACCAAAATCAACCAACAAGATTCACTCCAGTTGGCATTGGGGTTGATAACGAGGCAGACTTTCAACAATGGTCTTTACCCGACTATTCTGGTCAGTTCACCCACAACATGAACTTGGCACCAGCTGTTGCCCCCAACTTCCCTGGTGAGCAGCTCCTCTTCTTCCGCTCACAGTTACCATCTTCTGGTGGACGGTCCAACGGGATTCTAGACTGCCTAGTTCCCCAAGAATGGGTTCAGCACTTCTACCAAGAATCGGCCCCCGCCCAAACACAGGTGGCCTTGGTTAGGTATGTCAACCCTGACACTGGTAGAGTGTTATTTGAGGCTAAGCTGCACAAATTAGGTTTCATGACTATAGCTAAGAGTGGTGATTCCCCAATAACTGTCCCTCCAAATGGATATTTTAGATTTGAATCTTGGGTGAACCCCTTTTATACACTTGCCCCCATGGGAACTGGGAATGGGCGCAGAAGGATTCAATAA

>AF190817|II.a/II.3|<2001|US|Arg320

ATGAAGATGGCGTCGAATGACGCCACTCCATCTAATGATGGTGCCGCCGGCCTCGTCCCAGAGATCAACAATGAGGCAATGGCGCTAGACCCAGTGGCGGGTGCAGCGATAGCAGCACCCCTCACTGGTCAGCAAAACATAATTGATCCCTGGATTATGAATAATTTTGTGCAAGCACCTGGTGGTGAGTTTACAGTGTCCCCTAGGAATCCCCCTGGTGAAGTGCTTCTTAATTTGGAATTGGGCCCAGAAATAAACCCCTATTTGGCCCATCTTGCTAGAATGTATAATGGTTATGCAGGTGGATTTGAAGTGCAGGTAGTCCTGGCTGGGAATGCGTTTACAGCAGGAAAGATAATCTTTGCAGCTATACCTCCTAATTTTCCAATTGATAATCTGAGCGCAGCACAAATCACAATGTGCCCGCATGTGATTGTGGATGTCAGACAGTTGGAACCGGTCAACCTCCCGATGCCTGACGTTCGCAACAACTTCTTTCATTACAATCAAGGGTCTGATTCGAGATTGCGCTTAATTGCAATGCTGTATACACCTCTTAGGGCAAATAATTCTGGAGATGATGTTTTTACTGTGTCCTGTAGAGTACTGACTAGGCCTAGCCCTGACTTCTCATTCAATTTCCTTGTCCCACCTACTGTGGAATCAAAGACAAAACCCTTTACCCTCCCTATTCTGACTATCTCTGAAATGTCCAATTCTAGGTTTCCAGTGCCGATTGAGTCTTTGCACACCGGCCCAACTGAGAATATTGTTGTCCAGTGCCAAAATGGGCGCGTCACTCTTGATGGTGAGTTGATGGGCACCACCCAACTCTTACCGAGTCAAATTTGTGCTTTTAGGGGCGTGCTCACCAGATCAACAAGTAGGGCCAGTGATCAGGCCGATACAGCAACCCCTAGGCTGTTTAATTATTATTGGCATGTACAATTGGATAATCTAAATGGGACACCTTATGACCCTGCAGAAGACATACCAGGCCCCCTAGGGACACCAGACTTCCGGGGCAAGGTCTTTGGCGTGGCCAGCCAGAGAAACCCTGACAGCACAACTAGAGCACATGAAGCAAAGGTGGACACAACAGCTGGTCGTTTCACCCCAAAATTGGGCTCATTAGAGATATCTACTGACTCCAGTGACTTTGACCAAAACCAACCAACAAGATTCACCCCAGTTGGCATTGGGGTTGACAATGAGGCAGATTTTCAACAATGGTCTTTACCCGACTATTCTGGTCAATTCACTCACAACATGAACTTGGCCCCAGCTGTTGCTCCCAACTTCCCTGGTGAGCAGCTCCTTTTCTTCCGATCACAGTTACCATCTTCTGGTGGGCGATCCAACGGGGTTCTAGACTGTCTGGTCCCCCAGGAATGGGCTCAACACTTCTACCAGGAATCGGCCCCGGCCCAAACACAAGTGGCCCTGGTTAGGTATGTCAACCCTGACACTGGTAGAGTGCTATTTGAGGCCAAGCTGCATAAATTAGGTTTCATGACTATAGCTAAGAATGGTGACTCTCCAATAACTGTCCCCCCAAATGGATACTTTAGGTTTGAATCTTGGGTGAACCCCTTTTATACACTTGCCCCCATGGGAACTGGGAATGGGCGTAGAAGGATTCAATAA

>AF414411|II.3/II.3|1993|US|Lionville/247

ATGAAGATGGCGTCGAATGACGCTGCTCCATCTAATGATGGTGCCGCCGGCCTCGTCCCAGAGATCAACAATGAGGCAATGGCGCTAGAGCCAGTGGCGGGTGCAGCGATAGCAGCGCCCCTCACTGGCCAGCAAAATATAATTGATCCCTGGATTATGAATAATTTTGTGCAAGCACCTGGTGGTGAGTTTACAGTGTCACCTAGGAATTCCCCTGGTGAAGTGCTCCTTAATTTGGAATTAGGTCTAGAAATAAATCCCTATTTGGCTCATCTTGCTAGAATGTACAATGGTTATGCAGGTGTATTTGAAGTGCAAGTGGTCCTTGCTGGAAATGCGTTTACAGCAGGAAAGGTTATCTTCGCAGCTATACCCCCTAATTTCCCTATTGACAATCTGAGCGCGGCGCAGATCACAATGTGTCCGCACGTGATTGTGGATGTCAGGCAGTTGGAACCAATCAATCTCCCGATGCCTGATGTCCGCAACAATTTCTTTCATTATAATCAAGGTTCTGATTCAAGATTACGTTTGATTGCAATGTTGTATACACCTCTTAGGGCAAATAATTCTGGAGATGATGTTTTTACTGTGTCTTGTAGGGTGTTAACTAGGCCCAGCCCTGATTTCTCATTCAATTTTCTTGTCCCACCCACTGTGGAATCAAAGACAAAACTTTTTACCCTCCCCATTTTAACCATCTCTGAAATGTCCAATTCCAGGTTTCCAGTTCCAATTGACTCTCTGCACACCAGCCCAACTAAGAATATAGTTGTCCAGTGCCAAAATGGGCGCGTCACTCTTGACGGTGAGTTAATGGGCACCACCCAACTCTTACCGAGCCAAATATGTGCTTTCAGGGGCACACTCACTAGATCAACAAGCAGGGCCAGTGACCAAGCCGACACACCAACCCCTAGGCTATTCGGCCACCGTTGGCACATACAATTGGATAATCTAAATGGAACTCCCTACGACCCTGCAGAGGACATACCAGCTCCTTTGGGCACACCAGACTTCCGGGGCAAGGTCTTTGGTGTGGCCAGCCAGAGAAACCCCGACAGCACAACAAGGGCACATGAAGCAAAAGTGGACACGACATCTGGCCGCTTCACCCCAAAATTGGGCTCCTTAGAAATAATCACTGAATCTGATGACTTTGACACAAACCAGTCAACAAAATTCACCCCAGTTGGCATCGGAGTTGACAATGAGGCAGAATTTCAGCAATGGTCCTTGCCCAACTATTCTGGTCAGTTTACTCATAATATGAACTTAGCCCCAGCTGTAGCCCCCAATTTTCCTGGCGAACAGCTACTTTTCTTCCGATCACAGCTGCCATCCTCTGGTGGGCGGTCTAACGGGGTTCTAGACTGCCTGGTCCCCCAGGAATGGGTTCAACACTTTTACCAAGAATCAGCCCCCGCCCAAACGCAGGTGGCCCTGGTTAGGTATGTCAACCCTGACACTGGCAGAGTGCTATTTGAGGCCAAGCTACACAAATTGGGTTTTATGACTATAGCAAAGAATGGTGACTCCCCAATAACTGTCCCTCCAAATGGATATTTTAGATTTGAATCTTGGGTCAACCCCTTTTACACACTTGCCCCCATGGGAACTGGAAACGGGCGTAGAAGGATTCAATAA

>AF414412|II.3/II.3|1994|US|New Orleans/279

ATGAAGATGGCGTCGAATGACGCTGCTCCATCTAGTGATGGTGCCGCCGGCCTCGTCCCAGAGATCAACAATGAGGCAATGGCGCTAGAGCCAGTGGCGGGTGCAGCGATAGCAGCGCCCCTCACTGGCCAGCAAAATATAATTGATCCCTGGATTATGAATAATTTTGTGCAAGCACCTGGTGGTGAGTTTACAGTGTCACCTAGGAATTCCCCTGGTGAAGTGCTTCTTAATTTGGAATTAGGCCCAGAAATAAATCCCTATTTGGCTCATCTTGCTAGAATGTACAATGGTTATGCAGGTGGATTTGAAGTGCAAGTGGTCCTAGCTGGAAATGCGTTTACAGCAGGAAAGGTTATCTTTGCAGCTATACCCCCTAATTTCCCTATTGACAATCTGAGCGCGGCACAGATCACAATGTGTCCGCACGTGATTGTGGATGTCAGGCAGTTGGAACCAATCAACCTCCCGATGCCTGATGTCCGCAACAATTTCTTTCATTATAATCAAGGTTCTGATTCAAGATTACGCTTGATTGCAATGCTGTATACACCTCTTAGGGCAAACAATTCTGGAGATGATGTTTTCACTGTGTCTTGTAGGGTGTTAACTAGGCCTAGCCCTGATTTCTCATTTAATTTTCTTGTCCCACCCACTGTGGAATCAAAGACAAAACTTTTTACCCTCCCCATTTTAACCATCTCTGAAATGTCTAATTCCAGGTTTCCGGTGCCAATTGACTCTCTGCACACCAGCCCAACTGAGAATATAGTTGTCCAGTGCCAAAATGGGCGCGTCACTCTTGACGGTGAGTTAATGGGCACCACCCAACTCTTACCAAGCCAAATATGTGCTTTCAGGGGCACACTCACTAGATCAACAAGCAGGGCCAGTGACCAAGCCGACACACCAACCCCTAGGCTATTCAACTATTATTGGCACATACAATTGGATAATCTAAATGGAACTCCCTACGACCCTGCAGAGGACATACCAGCTCCTTTGGGCACACCGGACTTCCGGGGCAAGGTCTTTGGCGTAGCCAGCCAGAGAAACCCCGACAGCACAACAAGGGCACATGAAGCAAAAGTGGACACAACATCTGGCCGCTTCACCCCAAAATTGGGCTCCTTAGAAATAAGCACTGAATCTGATGACTTTGACCCAAACCAGCCAACAAAATTCACCCCAGTTGGCATTGGAGTTGACAATGAGGCAGATTTTCAACAATGGTCCTTACCTAACTATTCTGGTCAGTTTACTCATAATATGAATTTAGCCCCAGCTGTCGCCCCCAATTTTCCTGGTGAACAGCTACTTTTCTTCCGATCACAGCGGCCATCCTCTGGTGGGCGGTCTAACGGGATTCTAGACTGCCTGGTCCCCCAGGAATGGGTTCAACACTTTTACCAAGAATCGGCCCCCGCACAAACACAGGTGGCCCTGGTTAGGTATGTCAACCCTGACACTGGTAGAGTGCTATTTGAGGCCAAGCTACACAAATTGGGTTTTATGACTATAGCAAAGAATGGTGATTCCCCAATAACTGTCCCTCCAAATGGATATTTTAGATTTGAATCTTGGGTTAACCCCTTTTACACACTTGCCCCCATGGGAACTGGAAACGGGCGTAGAAGGATTCAATAA

>AF414413|II.3/II.3|1994|US|Montgomery/312

ATGAAGATGGCGTCGAATGACGCTGCTCCATCTAATGATGGTGCCGCCGGCCTCGTCCCAGAGATCAACAATGAGGCAATGGCGCTAGAGCCAGTGGCGGGTGCAGCGATAGCAGCGCCCCTCACTGGCCAGCAAAATATAATTGATCCCTGGATTATGAATAATTTTGTGCAAGCACCTGGTGGTGAGTTTACAGTGTCACCTAGGAATTCCCCTGGTGAAGTGCTTCTTAATTTGGAATTAGGTCCAGAAATAAATCCCTATTTGGCTCATCTTGCTAGAATGTACAATGGTTATGCAGGTGGATTTGAAGTGCAGGTGGTCCTAGCTGGAAATGCGTTTACAGCAGGAAAGGTTATCTTTGCAGCTATACCCCCTAATTTCCCTATTGACAATCTGAGCGCGGCACAGATCACAATGTGCCCGCACGTGATTGTGGATGTCAGGCAGTTGGAACCAATCAATCTCCCGATGCCTGATGTCCGCAACAATTTCTTTCATTATAATCAAGGTTCTGATTCAAGATTACGTTTGATTGCAATGTTGTATACACCTCTTAGGGCAAATAATTCTGGAGATGGTGTTTTTACTGTATCTTGTAGGGTGTTAACTAGGCCCAGCCCTGATTTCTCATTCAATTTTCTTGTCCCACCCACTGTGGAATCAAAGACAAGACTTTTTACCCTCCCCATTTTAACCATCTCTGAAATGTCCAATTCCAGGTTTCCAGTTCCAATTGACTCTCTGCACACCAGCCCAACTGAGAATATAGTTGTCCAGTGCCAAAATGGGCGCGTCACTCTTGACGGTGAGTTAATGGGCACCACCCAACTCTTACCGAGCCAAATATGTGCTTTCAGGGGCACACTCACTAGGTCAACAAGCAGGGCCAGTGACCAAGCCGACACACCAATCCCCAGGCTATTCAACCACCGTTGGCATATACAATTGGATAATCTGAATGGAACTCCCTACGACCCTGCAGAGGACATACCAGCTCCTTTGGGCACGCCAGACTTCCGGGGCAAGGTCTTTGGCGTGGCCAGCCAGAGAAACCCCGACAGCACAACAAGGGCACATGAAGCAAAAGTGGACGCAACACCTGGTCGCTTCACCCCAAAATTGGGCTCCTTAGAAATAATCACTGAATCTGATGACTTTGACACAAACCAGTCAACAAAATTCACCCCAGTTGGCATCGGAGTTGACAATGAGGCAGAATTTCAGCAATGGTCCTTACCTAACTATTCTGGTCAGTTTACTCACAATATGAACTTAGCCCCAGCTGTTGCCCCCAATTTTCCTGGTGAACAGCTACTTTTCTTCCGGTCACAGCTGCCATCCTCTGGTGGGCGTTCCAACGGGGTTCTAGACTGCCTGGTCCCCCAGGAATGGGTTCAACACTTTTACCAAGAATCAGCCCCCGCCCAAACGCAGGTGGCCCTGGTTAGGTATGTCAACCCTGACACTGGCAGAGTGCTATTTGAGGCCAAGCTGCACAAATTGGGTTTTATGACTATAGCAAAGAATGGTGACTCACCAATAACTGTCCCCCCAAATGGATACTTTAGATTTGAATCTTGGGTTAACCCCTTTTATACACTTGCCCCCATGGGAACTGGAAATGGGCGTAGAAGGATTCAATAA

>AF414414|II.3/II.3|1994|US|Towson/313

ATGAAGATGGCGTCGAATGACGCTGCTCCATCTAATGATGGTGCCGCCGGCCTCGTCCCAGAGATCAACAATGAGGCAATGGCGCTAGAGCCAGTGGCGGGTGCAGCGATAGCAGCGCCCCTCACTGGCCAGCAAAATATAATTGATCCCTGGATTATGAACAATTTTGTGCAAGCACCTGGTGGTGAGTTTACAGTGTCACCTAGGAATTCCCCTGGTGAAGTGCTTCTTAATTTGGAATTAGGTCCAGAAATAAATCCCTATTTAGCTCATCTTGCTAGAATGTACAATGGTTATGCAGGTGGATTTGAAGTGCAAGTGGTCCTAGCTGGAAATGCGTTTACAGCAGGAAAGGTTATCTTTGCAGCTATACCCCCTAATTTCCCTATTGATAATCTGAGCGCGGCACAGATCACAATGTGCCCGCACGTGATTGTGGATGTCAGGCAGTTGGAACCAATCAATCTCCCGATGCCTGATGTCCGCAACAATTTCTTTCATTATAATCAAGGTTCTGATTCAAGATTACGTTTGATTGCAATGCTGTATACACCTCTTAGGGCAAACAATTCTGGAGATGATGTTTTTACTGTGTCTTGTAGGGTGTTAACTAGGCCCAGCCCTGATTTCTCATTCAATTTTCTTGTCCCACCCACTGTGGAATCAAAGACAAAACTTTTTACCCTCCCCATTTTAACCATCTCTGAAATGTCCAATTCCAGGTTTCCAGTTCCAATTGACTCTCTGCACACTAGCCCAACTGAGAATATAGTTGTCCAGTGCCAAAATGGGCGCGTCACTCTTGACGGTGAGTTGATGGGCACCACCCAACTCTTACCGAGCCAAATATGTGCTTTCAGGGGCACACTCACTAGGTCAACAAGCAGGGCCAGTGACCAAGCCGACACACCAACTCCCAGGCTATTCAACCATCGCTGGCACATACAATTGGATAATCTAAATGGAACTCCCTACGACCCTGCAGAGGACATACCAGCTCCTTTGGGCACACCAGACTTCCGGGGCAAGGTCTTTGGCGTGGCCAGCCAGAGAAACCCCGACAGCACAACAAGGGCACATGAAGCAAAAGTGGACACAACATCTGGCCGCTTCACCCCAAAATTAGGCTCCTTAGAAATAATCACGGAATCTGACGACTTTGACACAAACCAGTCAACAAAATTCACCCCAGTTGGCATCGGAGTTGACAATGAGTCAGAATTTCAGCAATGGTCCTTACCCAACTATTCTGGTCAATTCACTCATAATATGAACTTAGCCCCAGCTGTTGCCCCCAATTTTCTTGGTGAACAGCTACTTTTCTTCCGGTCACAGCTGCCATCCTCTGGTGGGCGGTCTAACGGGGTTCTAGACTGCCTGGTCCCCCAGGAATGGGTTCAACACTTTTACCAAGAATCAGCCCCCGCCCAAACGCAGGTGGCCCTGGTTAGGTATGTCAACCCTGACACTGGTAGAGTGCTATTTGAGGCCAAGCTACACAAATTGGGTTTTATGACTATAGCAAAGAATGGTGACTCCCCAATAACTGTCCCTCCAAATGGATACTTTAGATTTGAATCTTGGGTTAACCCCTTTTATACACTTGCCCCCATGGGAACTGGAAACGGGCGTAGAAGGATTCAATAA

>AF414415|II.3/II.3|1995|US|Brattleboro/321

ATGAAGATGGCGTCGAATGACGCTGCTCCATCTAATGATGGTGCTGCCGGCCTCGTCCCAGAGATCAACAATGAGGCAATGGCGCTAGAGCCAGTGGCGGGTGCAGCGATAGCGGCACCCCTCACTGGCCAGCAAAATATAATTGATCCCTGGATTATGAATAATTTTGTGCAAGCACCTGGTGGTGAGTTTACAGTGTCACCTAGGAATTCCCCTGGTGAAGTGCTTCTTAATTTGGAATTAGGTCCAGAAATAAATCCCTATTTGGCTCATCTTGCTAGAATGTACAATGGTTATGCAGGTGGATTTGAAGTGCAAGCGGTCCTAGCTGGAAATGCGTTTACAGCAGGAAAGGTTATCTTTGCAGCTATACCCCCTAATTTCCCTATTGACAATCTGAGTGCGGCACAGATCACAATGTGCCCGCACGTGATTGTGGATGTCAGGCAGTTGGAACCAATCAATCTCCCGATGCCTGATGTCCGCAACAATTTCTTTCATTATAATCAAGGTTCTGACTCAAGATTACGTTTGATTGCAATGTTGTATACACCTCTTAGGGCAAACAATTCTGGAGATGATGTTTTTACTGTGTCTTGTAGGGTGTTAACTAGGCCCAGCCCTGATTTCTCATTCAATTTTCTTGTCCCACCCACTGTGGAATCAAAGACAAAACTTTTTACCCTCCCCATTTTAACCATCTCTGAAATGTCCAATTCCAGGTTTCCAGTTCCAATTGACTCTCTGCACACCAGCCCAACTGAGAATATAGTTGTCCAGTGCCAAAATGGGCGCGTCACTCTTGACGGTGAGTTAATGGGCACCACCCAACTCTTACCGAGCCAAATATGTGCTTTCAGGGGCACACTCACTAGGTCAACAAGCAGGGCCAGTGACCAAGCCGACACACCAACCCCCAGGCTATTCAACCATCGTTGGCACATACAATTGGATAATCTAAATGGAACTCCCTACGACCCTGCAGAGGACATACCAGCTCCTTTGGGCACACCAGACTTCCGGGGCAAGGTCTTTGGCGTGGCCAGCCAGAGAAACCCCGACAGCACAACAAGGGCACATGAAGCAAAAGTGGACACAACATCTGACCGCTTCACCCCAAAATTGGGCTCCTTAGAAATAATCACTGAATCTGGTGACTTTGACACAAACCAGTCAACAAAATTCACCCCAGTTGGCATCGGAGTTGACAATGAGGCAGAATTTCAGCAATGGTCCTTACCCAACTATTCTGGTCAATTTACTCATAATATGAACTTAGCCCCAGCTGTTGCCCCCAATTTTCCTGGAGAACAGCTACTTTTCTTCCGGTCACAGCTGCCATCCTCTGGTGGGCGGTCTAACGGGGTTCTAGACTGCCTGGTCCCCCAGGAATGGGTTCAACACTTTTACCAAGAATCAGCCCCCGCCCAAACGCAGGTGGCCCTGGTTAGGTATGTCAACCCTGACACTGGCAGAGTGCTATTTGAGGCCAAGCTACACAAATTGGGTTTTATGACTATAGCAAAGAATGGTGACTCCCCAATAACTGTCCCTCCAAATGGATACTTTAGATTTGAATCTTGGGTTAACCCCTTTTATACACTTGCCCCCATGGGAACTGGAAACGGGCGTAGAAGGATTCAATAA

>AF425768|-/II.3|2001|DE|Oberhausen 455

ATGAAGATGGCGTCGAATGACGCCACTCCATCTAATGATGGTGCCGCCGGCCTCGTCCCAGAGATCAACAATGAGGCAATGGCGCTAGATCCAGTGGCGGGTGCAGCGATAGCAGCACCCCTCACTGGTCAGCAGAATATAATTGATCCCTGGATTATGAATAATTTTGTGCAAGCACCTGGTGGTGAGTTTACAGTATCCCCTAGGAATTCCCCTGGTGAAGTGCTTCTTAATTTGGAATTGGGCCCAGAAATAAATCCCTATTTGGCCCATCTTGCTAGAATGTATAATGGTTATGCAGGTGGATTTGAAGTGCAGGTAGTCCTAGCTGGAAATGCGTTTACAGCAGGAAAGATAATCTTTGCAGCTATACCCCCTAATTTTCCAATTGATAATCTAAGCGCAGCACAGATCACAATGTGCCCACATGTGATTGTGGATGTCAGACAGTTGGAACCGGTCAACCTCCCGATGCCTGACGTTCGCAACAACTTCTTTCATTACAATCAAGGGTCTGATTCGAGATTGCGCTTAATTGCAATGCTGTATACACCTCTTAGGGCAAATAATTCTGGGGATGATGTTTTTACTGTGTCTTGTAGAGTGCTGACTAGGCCTAGCCCTGACTTTTCATTTAATTTCCTTGTGCCACCTACTGTGGAGTCAAAGACAAAACCCTTCACCCTCCCTATTCTGACTATCTCTGAAATGTCCAATTCTAGGTTTCCAGTGCCGATTGATTCTCTGCACACCAGCCCAACTGAGAATATTGTTGTCCAGTGCCAAAATGGGCGCGTCACTCTTGATGGTGAGTTGATGGGCACCACCCAACTCTTACCTAGTCAAATCTGTGCTTTCAGGGGCGTGCTCACCAGATCAACAAGCAGGGCCAGTGATCAGGCCGACACAGCAACCCCTAGGTTGTTTAATTATTATTGGCACATACAATTGGATAATCTAAGTGGGACTCCTTATGATCCTGCAGAAGACATACCAGGCCCCCTAGGGACACCAGATTTCCGGGGCAAGGTCTTTGGCGTGGCCAGCCAGAGAAATCCCGACAGCACAACTAGAGCACATGAAGCAAAGGTGGACACAACAGCTGGTCGTTTCACCCCAAAACTAGGCTCATTAGAGATATCCACTGAATCAGATGACTTTGATCAAAACCAACCAACAAGATTCACCCCAGTTGGCGTTGGGGTTGACCATGAGGCAGACTTTCAACAATGGTCTTTACCCGACTATTCTGGTCAGTTCACCCACAACATGAACTTAGCCCCAGCTGTTGCTCCCAACTTCCCTGGTGAGCAGCTCCTTTTCTTTCGCTCACAGTTACCATCCTCTGGTGGGCGATCCAACGGGATTCTAGACTGCCTGGTCCCCCAAGAATGGGTTCAGCACTTCTACCAAGAATCGGCCCCCGCCCAAACTCAAGTGGCCCTGGTTAGGTATGTCAACCCTGACACTGGTAGAGTATTATTTGAGGCCAAGCTGCACAAATTAGGTTTCATGACTATAGCTAAGAATGGTGACTCTCCAATAACTGTCCCTCCAAATGGATACTTTAGGTTTGAATCTTGGGTGAACCCATTTTATACACTTGCCCCCATGGGAACTGGGAATGGGCGTAGAAGGATTCAATAA

>AF427111|-/II.3|2001|DE|Berlin/226

ATGAAGATGGCGTCGAATGACGCCACTCCATCTAATGATGGTGCCGCCGGCCTCGTCCCAGAGATCAACAATGAGGCAATGGCGCTAGATCCAGTGGCGGGTGCAGCGATAGCAGCACCCCTCACTGGTCAGCAAAATATAATTGATCCCTGGATTATGAATAATTTTGTGCAAGCACCTGGTGGTGAGTTTACAGTATCCCCTAGGAATTCCCCTGGTGAAGTGCTTCTTAATTTGGGATTGGGCCCAGAAATAAATCCCTATTTGGCCCATCTTGCTAGAATGTATAATGGTTATGCAGGTGGATTTGAAGTGCAGGTAGTCCTAGCTGGAAATGCGTTTACAGCAGGAAAGATAATCTTTGCAGCTAWACCCCCTAATTTTCCAATTGATAATCTAAGCGCAGCACAGATCACAATGTGCCCACATGTGATTGTGGATGTCAGACAGTTGGAACCGGTCAACCTCCCGATGCCTGACGTTCGCAATAATTTCTTTCATTACAATCAAGGGTCTGATTCGAGATTGCGTTTAATTGCAATGCTGTATACACCTCTTAGGGCAAATAATTCTGGGGATGATGTTTTTACTGTGTCTTGTAGAGTGCTGACTAGGCCTAGCCCTGACTTTTCATTTAATTTCCTTGTGCCACCTACTGTGGAGTCAAAGACAAAACCCTTCACCCTCCCTATTCTGACTATCTCTGAAATGTCCAATTCTAGGTTTCCAGTGCCGATTGATTCTCTGCACACCAGCCCAACTGAGAATATTGTTGTCCAGTGCCAAAATGGGCGCGTCACTCTTGATGGTGAGTTGATGGGCACCACCCAACTCTTACCTAGTCAAATCTGTGCCTTTAGGGGCATGCTCACCAGATCAACAAGCAGGACCAGCGATCAGGCTGACACAGCAACCCCTAGGTTGTTTAATTATTATTGGCACATACAATTGGATAATCTAAATGGGACTCCTTATGATCCTGCAGAAGACATACCAGGCCCCCTAGGGACACCAGATTTCCGGGGCAAAGTCTTTGGCGTGGCCAGCCAGAGAAATCCCGACAGCACAACTAGAGCACATGAGGCAAAGGTGGACACGACAGCTGGTCGTTTCACCCCAAAACTAGGCTCATTAGAGATATCCACTGAATCTGGTGACTTTGATCAAAACCAACCAACAAGATTCACCCCAGTTGGCATTGGGGTTGACCAAGAGGCAGACTTTCAACAATGGTCTTTACCCGACTATTCTGGTCAGTTCACCCACAACATGAACTTAGCCCCAGCTGTTGCTCCCAGCTTCCCTGGTGAGCAGCTCCTTATCTTCCGCTCACAGTTACCATCCTCTGGTGGGCGATCCAACGGGGTTCTAGACTGCCTGGTCCCCCAAGAATGGGTTCAGCACTTCTACCAAGAATCGGCCCCCGCCCAAACTCAAGTGGCCCTGGTCAGGTATGTCAACCCTGACACTGGTAGAGTATTATTCGAGGCCAAGCTGCACAAATTAGGTTTCATGACTATAGCTAAGAATGGTGACTCTCCAATAACTGTCCCTCCAAATGGATACTTTAGGTTTGAATCTTGGGTGAACCCCTTTTATACACTTGCCCCCATGGGAACGGGGAATGGGCGTAGAAGGATTCAATAA

>AF427112|-/II.3|<2001|DE|Bitburg/289

ATGAAGATGGCGTCGAATGACGCCACTCCATCTAATGATGGTGCCGCCGGCCTCGTCCCAGAGATCAACAATGAGGCAATGGCGCTAGATCCAGTGGCGGGTGCAGCGATAGCAGCACCCCTCACTGGTCAGCAAAATATAATTGATCCCTGGATTATGAATAATTTTGTGCAAGCACCTGGTGGTGAGTTTACAGTATCCCCTAGGAATTCTCCTGGTGAAGTGCTTCTTAATTTGGAATTGGGCCCAGAAATAAATCCCTATTTGGCCCATCTTGCTAGAATGTATAATGGTTATGCAGGTGGATTTGAAGTGCAGGTAGTCCTAGCTGGAAATGCGTTTACAGCAGGAAAGATAATCTTTGCAGCTATACCCCCTAATTTTCCAATTGATAATCTAAGCGCAGCACAGATCACAATGTGCCCACATGTGATTGTGGATGTCAGACAGTTGGAACCGGTCAACCTCCCGATGCCTGACGTTCGCAACAACTTCTTTCATTACAATCAAGGGTCTGATTCGAGATTGCGCTTAATTGCAATGCTGTATACACCTCTTAGGGCAAATAATTCTGGGGATGATGTTTTTACTGTGTCTTGTAGAGTGCTGACTAGGCCTAGCCCTGACTTTTCATTTAATTTCCTTGTGCCACCTACTGTGGAGTCAAAGACAAAACCCTTCACCCTCCCCATTCTGACTATCTCTGAAATGTCCAATTCTAGGTTTCCAGTGCCGATTGATTCTCTGCACACCAGACCAACTGAGAATATTGTTGTCCAGTGCCAAAATGGGCGCGTCACTCTTGATGGTGAGTTGATGGGCACCACCCAACTCTTACCTAGTCAAATCTGTGCTTTTAGGGGCGTGCTCACCAGATCAACAAGCAGGGCCAGTGATCAGGCCGACACAGCAACCCCTAGGTTGTTTAATTATTATTGGCACATACAATTGGATAATCTAAATGGGACTCCTTGTGATCCTGCAGAAGACATACCAGGCCCCCTAGGGACACCAGATTTCCGGGGCAAAGTCTTTGGCGTGGCCAGCCAGAGAAATCCCGACAGCACAACTGGAGCACATGAAGCAAAGGTGGACACAACAGCTGGTCGCTTCACCCCAAAACTAGGCTCATTAGAGATATCCACTGAATCTGGTGACTTTGATCAAAACCAACCAACAAAATTCACCCCAGTCGGCATTGGGGTTGACCATGAGGCAGACTTTCAACAATGGTCTTTACCCGACTATTCTGGTCAGTTCACCCACAACATGAACTTAGCCCCAGCTGTTGCTCCCAACTTCCCTGGTGAGCAGCTCCTTTTCTTCCGCTCACAGTTACCATCCTCTGGTGGGCGATCCAACGGGATTCTAGACTGCCTGGTCCCCCAAGAATGGGTTCAGCACTTCTACCAAGAATCGGCCCCCGCCCAAACTCAAGTAGCCCTGGTTAGGTATGTCAACCCTGACACTGGTAGAGTATTATTTGAGGCCAAGCTGCACAAATTAGGTTTCATGACTATAGCTAAGAATGGTGACTCTCCAATAACTGTCCCTCCAAATGGATACTTTAGGTTTGAATCTTGGGTGAACCCCTTTTATACACTTGCCCCCATGGGAACTGGGAATGGGCGTAGAAGGATTCAATAA

>AF539439|II.21/II.3|2001|DE|Herzberg 38

ATGAAGATGGCGTCGAGTGACGCCACCCCATCTAACGATGGTGCCGCCGGCCTCGTCCCAGAGATCAACAATGAGGCAATGGCGCTAGATCCGGTGGCGGGTGCAGCGATAGCAGCACCCCTCACTGGTCAGCAAAATATAATTGATCCCTGGATTATGAATAATTTTGTGCAAGCACCTGGTGGTGAGTTTACAGTGTCCCCTAGGAATTCCCCTGGTGAATTGCTTCTTAACTTGGAATTGGGTCCAGAAATAAACCCCTATTTAGCCCATCTTGCTAGAATGTATAATGGTTATGCAGGTGGATTTGAAGTGCAGGTAGTCCTGGCTGGAAATGCGTTTACAGCAGGGAAGATAATCTTTGCAGCTATACCCCCTAATTTTCCAATTGACAATCTGAGCGCAGCACAGATCACGATGTGTCCGCATGTGATTGTGGATGTCAGACAGTTGGAACCGGTCAACCTTCCGATGCCTGACGTTCGCAACAACTTCTTTCATTACAATCAAGGGTCTGATTCGAGATTGCGCTTAATTGCAATGCTGTATACACCTCTTAGGGCAAATAATTCTGGGGATGATGTTTTCACTGTGTCTTGTAGAGTGTTGACTAGGCCTAGCCCTGACTTTTCATTTAATTTCCTTGTGCCACCTACTGTGGAGTCAAAGACAAAACCCTTCACCCTCCCTATTCTGACTATCTCTGAAATGTCCAATTCTAGGTTTCCAGTGCCGATTGATTCTCTGCACACCAGCCCAACTGAGAACATTGTTGTCCAGTGCCAAAATGGGCGCGTCACTCTTGACGGTGAGTTGATGGGCACCACCCAACTCTTACCGAGTCAAATCTGTGCTTTTAGGGGCGTGCTCACCAGATCAACAAGCAGGGCCAGTGATCAGGCCGACACACCAACCCCTAGGTTGTTTAATTATTATTGGCACATACAATTGGACAACCTAAATGGGACTCCTTATGACCCTGCAGAAGACATACCAGGCCCCCTAGGGACACCAGATTTTCGGGGCAAAGTCTTTGGCGTGGCCAGCCAGAGAAATCCCGACAGCACAACTAGAGCACATGAAGCAAAGGTGGACACAACAGCTGGTCGTTTCACCCCAAAATTAGGCTCATTAGAGATATCCACTGAATCTGGTGACTTTGACCAAAACCAACCAACAAGATTCACCCCAGTTGGCATTGGGGTTGACCATGAGGCAGACTTTCAACAATGGTCTTTACCCGACTATTCTGGTCAGTTCACTCACAACATGAACTTGGCCCCAGCTGTTGCTCCCAACTTCCCTGGTGAGCAGCTCCTTTTCTTCCGCTCACAATTACCATCCTCTGGTGGGCGATCCAACGGGATTCTAGACTGCCTGGTCCCCCAAGAATGGGTTCAGCACTTCTACCAAGAATCTGCCCCCGCCCAAACACAAGTGGCCCTGGTTAGGTATGTCAACCCTGACACTGGTAGAGTGTTATTTGAGGCCAAGCTGCACAAGTTAGGTTTCATGACTATAGCTAAGAATGGTGACTCTCCAATAACTGTCCCTCCAAATGGATACTTTAGGTTTGAATCTTGGGTAAACCCCTTTTATACACTTGCCCCCATGGGAACTGGGAATGGGCGTAGAAGGATTCAATAA

>AF539440|II.21/II.3|2001|DE|Oberhausen 455

ATGAAGATGGCGTCGAATGACGCCACTCCATCTAATGATGGTGCCGCCGGCCTCGTCCCAGAGATCAACAATGAGGCAATGGCGCTAGATCCAGTGGCGGGTGCAGCGATAGCAGCACCCCTCACTGGTCAGCAGAATATAATTGATCCCTGGATTATGAATAATTTTGTGCAAGCACCTGGTGGTGAGTTTACAGTATCCCCTAGGAATTCCCCTGGTGAAGTGCTTCTTAATTTGGAATTGGGCCCAGAAATAAATCCCTATTTGGCCCATCTTGCTAGAATGTATAATGGTTATGCAGGTGGATTTGAAGTGCAGGTAGTCCTAGCTGGAAATGCGTTTACAGCAGGAAAGATAATCTTTGCAGCTATACCCCCTAATTTTCCAATTGATAATCTAAGCGCAGCACAGATCACAATGTGCCCACATGTGATTGTGGATGTCAGACAGTTGGAACCGGTCAACCTCCCGATGCCTGACGTTCGCAACAACTTCTTTCATTACAATCAAGGGTCTGATTCGAGATTGCGCTTAATTGCAATGCTGTATACACCTCTTAGGGCAAATAATTCTGGGGATGATGTTTTTACTGTGTCTTGTAGAGTGCTGACTAGGCCTAGCCCTGACTTTTCATTTAATTTCCTTGTGCCACCTACTGTGGAGTCAAAGACAAAACCCTTCACCCTCCCTATTCTGACTATCTCTGAAATGTCCAATTCTAGGTTTCCAGTGCCGATTGATTCTCTGCACACCAGCCCAACTGAGAATATTGTTGTCCAGTGCCAAAATGGGCGCGTCACTCTTGATGGTGAGTTGATGGGCACCACCCAACTCTTACCTAGTCAAATCTGTGCTTTCAGGGGCGTGCTCACCAGATCAACAAGCAGGGCCAGTGATCAGGCCGACACAGCAACCCCTAGGTTGTTTAATTATTATTGGCACATACAATTGGATAATCTAAATGGGACTCCTTATGATCCTGCAGAAGACATACCAGGCCCCCTAGGGACACCAGATTTCCGGGGCAAGGTCTTTGGCGTGGCCAGCCAGAGAAATCCCGACAGCACAACTAGAGCACATGAAGCAAAGGTGGACACAACAGCTGGTCGTTTCACCCCAAAACTAGGCTCATTAGAGATATCCACTGAATCTGATGACTTTGATCAAAACCAACCAACAAGATTCACCCCAGTTGGCGTTGGGGTTGACCATGAGGCAGACTTTCAACAATGGTCTTTACCCGACTATTCTGGTCAGTTCACCCACAACATGAACTTAGCCCCAGCTGTTGCTCCCAACTTCCCTGGTGAGCAGCTCCTTTTCTTTCGCTCACAGTTACCATCCTCTGGTGGGCGATCCAACGGGATTCTAGACTGCCTGGTCCCCCAAGAATGGGTTCAGCACTTCTACCAAGAATCGGCCCCCGCCCAAACTCAAGTGGCCCTGGTTAGGTATGTCAACCCTGACACTGGTAGAGTATTATTTGAGGCCAAGCTGCACAAATTAGGTTTCATGACTATAGCTAAGAATGGTGACTCTCCAATAACTGTCCCTCCAAATGGATACTTTAGGTTTGAATCTTGGGTGAACCCATTTTATACACTTGCCCCCATGGGAACTGGGAATGGGCGTAGAAGGATTCAATAA

>AJ277611|-/II.3|1995|UK|Bham132/95

ATGAAGATGGCGTCGAATGACGCTGCTCCATCTAATGATGGTGCCGCCGGCCTCGCTCCAGAGATCAACAATGAGGCAATGGCGCTAGAGCCAGTGGCGGGTGCAGCGATAGCAGCGCCCCTCACTGGCCAGCAAAATATAATTGATCCCTGGATTATGAATAATTTTGTGCAAGCACCTGGTGGTGAGTTTACAGTGTCACCTAGGAATTCTCCTGGTGAAGTGCTTCTTAATTTGGAATTAGGTCCAGAAATAAATCCCTATTTGGCTCATCTTGCTAGAATGTACAATGGTTATGCAGGTGGATTTGAAGTGCAAGTGGTCCTAGCTGGAAATGCGCTTACAGCAGGAAAGGGTATCTTTGCAGCTATACCCCCTAATTTCCCTATTGACAATCTGAGCGCGGCACAGATCACAATGTGCCCGCACGTGATTGTGGATGTCAGGCAGTTGGAACCAATCAATCTCCCGATGCCTGATGTCCGCAACAATTTCTTTCATTATAATCAAGGTTCTGATTCAAGATTACGTTTGATTGCAATGTTGTATACACCTCTTAGGGCAAATAATTCTGGAGATGATGTTTTCACTGTGTCTTGTAGGGTGTTAACTAGGCCCAGCCCTGATTTCTCATTTAATTTTCTTGTCCCACCCACTGTGGAATCAAAAACAAAACTTTTTACCCTCCCCATTTTAACCATCTCTGAAATGTCCAATTCCAGGTTTCCAGTTCCAATTGACTCTCTGCACACCAGCCCAACTGAGAATATAGTTGTCCAGTGCCAAAATGGGCGCGTCACTCTTGACGGTGAGTTAATGGGCACCACCCAACTCTTACCGAGCCAAATATGTGCTTTCAGGGGCACACTCACTAGATCAACGAGCAGGGCCAGTGACCAAGTCGACACACCAACCCCCAGGCTATTCAACCATCGTTGGCACATACAATTGGATAATCTAAATGGAACTCCCTACGACCCTGCAGAGGACATACCAGCTCCTTTGGGCACACCAGACTTCCGGGGCAAGGTCTTTGGCGTGGCCAGCCAGAGAAACCCCGACAGTACAACAAGGGCACATGAAGCAAAAGTGGACACAACATCTGGTCGCTTCACCCCAAAATTGGGCTCCTTAGAAATAATCACTGAATCTGATGATTTTGATGCAAACCAGTCAACAAAATTCACCCCAGTTGGCATCGGAGTTGACAATGAGGCAGAATTTCAGCAATGGTCCTTACCCAACTATTCTGGTCAGTTTACTCATAATATGAACTTAGCCCCAGCTGTCGCCCCCAATTTTCCTGGTGAACAGCTACTTTTCTTCCGATCACAGCTACCATCCTCTGGTGGGCGGTCTAACGGGGTTCTAGACTGCCTGGTCCCCCAGGAATGGGTTCAACACTTTTACCAAGAATCAGCCCTCGCCCAAASGCARGTGGCCCTGGTTAGGTATGTCAACCCTGACACTGGCAGAGTGCTATTTGAGGCCAAGCTACACAAATTGGGTTTTATGACTATAGCAAAGAATGGTGACTCCCCAATAACTGTCCCTCCAAMTGGATATTTTAGATTTGAATTTTGGGTTAACCCTTTTTACACATTTGCCCCCATGGGAAGTGGAAACGGGCGTAGAAGGATTCAATAA

>AJ277617|-/II.3|1993|UK|Rbh/93

ATGAAGATGGCGTCGAATGACGCTGCTCCATCTAATGATGGTGCCGCCGGCCTCGTCCCAGAGATCAACAATGAGGCAATGGCGCTAGAGCCAGTGGCGGGTGCAGCGATAGCAGCGCCCCTCACTGGCCAGCAAAATATAATTGATCCCTGGATTATGAATAATTTTGCGCAAGCACCTGGTGGTGAGTTTACAGTGTCACCTAGGAACTCCCCTGGTGAAGTGCTTCTTAATTTGGAATTAGGTCCAGAAATAAATCCCTATTTGGCTCATCTTGCTAGAATGTACAATGGTTATGCAGGTGGATTTGAAGTGCAAGTGGTCCTAGCTGGAAATGCGTTTACAGCAGGAAAGGTTATCTTTGCAGCTATACCCCCTAATTTCCCTATTGACAATCTGAGCGCGGCACAGATCACTATGTGCCCGCACGTGATTGTGGACGTCAGGCAGTTGGAACCAATCAATCTCCCGATGCCTGATGTCCGCAACAATTTCTTTCATTATAATCAAGGTTCTGATTCAAGATTACGTTTGATTGCAATGTTGTATACACCTCTTAGGGCAAATAATTCTGGAGATGAAGTTTTCACTGTGTCTTGTAGGGTGTTAACTAGGCCCAGCCCTGATTTCTCATTCAATTTTCTTGTCCCACCCACTGTGGAATCAAAGACAAAACTTTTTACCCTCCCCATTTTAACCATCTCTGAAATGTCCAATTCCAGGTTTCCAGTTCCAATTGACTCTCTGCACACCAGCCCAACTGAGAATATAGTTGTCCAGTGCCAAAATGGGCGCGTCACTCTTGATGGTGAGTTAATGGGCACCACCCAACTCTTACCGAGCCAAATATGTGCTTTCAGGGGCACACTCACTAGATCAACAAGCAGGGCCAGTGACCAAGCCGACACACCAACCCCCAGGCTATTCAACCATCGTTGGCACATACAATTGGATAATCTAAATGGAACTCCCTACGACCCTGCAGAGGACATACCAGCTCCTTTGGGCACACCAGACTTCCGGGGTAAGGTCTTTGGCGTGGCCAGCCAGAGAAACCCCGACAGCACAACAAGGGCACATGAAGCAAAAGCGGACACAACATCTGGCCGCTTCACCCCAAAATTGGGCTCCTTAGAAATAATCACTGAATCTGATGACTTTGACACAAACCAGTCAACAAAATTCACCCCAGTTGGCATCGGAGTTGACAATGAGGCAGAATTTCAGCAATGGTCCTTACCCAACTATTCTGGTCAGTTTACTCATAATATGAACTTAGCCCCAGCTGTCGCTCCCAATTTTCCTGGTGAACAGCTACTTTTCTTCCGATCACAGCTGCCATCTTCTGGTGGGCGGTCTAACGGGGTTCTAGACTGCCTGATCCCCCAGGAATGGGTTCAACACTTTTACCAAGAATCAGCCCCCGCCCAAACGCAGGTGGCCCTGGTTAGGTATGTCAACCCTGACACTGGCAGAGTGCTATTTGAGGCCAAGCTACACAAATTGGGTTTTATGACTATAGCAAAGAATGGTGACTCCCCAATAACTGTCCCTCCAAATGGATACCTTAGATTTGAATCTTGGGTTAACCCCTTTTACACACTTGCCCCCATGGGAACTGGAAACGGGCGTAGAAGGATTCAATAA

>AY030312|-/II.3|1987|US|MD101-2

ATGAAGATGGCGTCGAATGACGCTGCTCCATCTAATGATGGTGCCGCCGGCCTCGTCCCAGAGATCAACAATGAGGCAATGGCGCTAGATCCAGTGGCGGGTGCAGCGATAGCAGCACCCCTCACTGGCCAGCAAAATATAATTGATCCCTGGATTATGAATAACTTTGTGCAAGCACCTGGTGGTGAGTTTACAGTGTCACCTAGGAATTCCCCTGGTGAAGTGCTTCTTAACTTGGAATTAGGTCCAGAAATAAATCCCTATTTGGCTCATCTTGCTAGAATGTACAATGGTTATGCAGGTGGGTTTGAAGTGCAAGTGGTCCTGGCTGGAAATGCGTTTACAGCAGGAAAGGTGATCTTTGCAGCTATACCCCCCAACTTCCCTATTGATAATCTGAGCGCAGCACAGATCACAATGTGCCCGCACGTGATTGTGGATGTCAGGCAGTTGGAACCAATCAATCTTCCGATGCCTGATGTCCGCAACAATTTCTTTCATTATAATCAAGGGTCTGATTCAAGATTACGTTTAATTGCAATGCTGTATACACCTCTTAGGGCAAATAATTCTGGAGATGATGTTTTCACTGTGTCTTGTAGGGTGTTAACTAGGCCTAGCCCTGATTTCTCATTCAATTTTCTTGTCCCACCCACTGTGGAATCAAAGACAAAACCTTTTACCCTCCCCATTTTAACCATCTCTGAAATGTCTAATTCCAGGTTTCCGGTGCCAATTGACTCTCTGCACACCAGCCCAACTGAGAGTATCGTTGTCCAGTGCCAAAATGGGCGCGTCACTCTTGACGGTGAGTTAATGGGCACCACCCAACTCTTACCGAACCAAATATGTGCTTTCAGGGGAACACTTACTAGATCAACAAACAGGGCCAGTGACCAAGCCGACACAGCAACCCCCAGGCTATTTAACCATCATTGGCACATACAATTGGATAATCTAAATGGAACCCCCTACGACCCTGCAGAGGACATACCAGCTCCTTTGGGTACACCAGACTTCCGGGGCAAAGTCTTTGGCGTAGCCAGCCAGAGAAACCCCGACAGCACAACAAGGGCACATGAAGCAAAAGTGGACACAACATCTGGCCGCTTCACCCCAAAATTGGGCTCCCTAGAAATAACCACTGAATCTGATGACTTTGACCCAAACCAGTCAACAAAATTCACCCCAGTTGGCATTGGAGTTGACAATGAGGCAGATTTTCAGCAGTGGTCCTTACCTGACTATTCCGGTCAGTTTACTCATAACATGAACTTAGCCCCAGCTGTCGCCCCCAATTTTCCTGGTGAACAGCTTCTTTTCTTCCGATCACAGCTGCCATCTTCTGGTGGGCGGTCTAACGGGATTCTAGACTGCCTGGTCCCCCAGGAATGGGTTCAACACTTCTACCAAGAATCAGCCCCCGCCCAAACACAGGTGGCCCTGGTTAGGTATGTCAACCCTGACACTGGTAGAGTGCTATTTGAGGCCAAGCTACACAAATTGGGTTTTATGACTATAGCAAAGAATGGTGACTCCCCAATAACTGTCCCTCCAAATGGGTATTTTAGATTTGAATCTTGGGTTAACCCCTTTTACACACTTGCCCCCATGGGAACTGGAAACGGGCGTAGAAGGATTCAATAA

>AY030313|-/II.3|1987|US|MD134-10

ATGAAGATGGCGTCGAATGACGCTGCTCCATCTAATGATGGTGCCGCCGGCCTCGTCCCAGAGATCAACAATGAGGCAATGGCGCTAGATCCAGTGGCGGGTGCAGCGATAGCAGCACCCCTCACTGGCCAGCAAAATATAATTGATCCCTGGATTATGAATAACTTTGTGCAAGCACCTGGTGGTGAGTTTACAGTGTCACCTAGGAATTCCCCTGGTGAAGTGCTTCTTAACTTGGAATTAGGTCCAGAAATAAATCCCTATTTGGCTCATCTTGCTAGAATGTACAATGGTTATGCAGGTGGGTTTGAAGTGCAAGTGGTCCTGGCTGGAAATGCGTTTACAGCAGGAAAGGTGATCTTTGCAGCTATACCCCCCAACTTCCCTATTGATAATCTGAGCGCAGCACAGATCACAATGTGCCCGCACGTGATTGTGGATGTCAGGCAGTTGGAACCAATCAATCTTCCGATGCCTGATGTCCGCAACAATTTCTTTCATTATAATCAAGGGTCTGATTCAAGATTACGTTTAATTGCAATGCTGTATACACCTCTTAGGGCAAATAATTCTGGAGATGATGTTTTCACTGTGTCTTGTAGGGTGTTAACTAGGCCTAGCCCTGATTTCTCATTCAATTTTCTTGTCCCACCCACTGTGGAATCAAAGACAAAACCTTTTACCCTCCCCATTTTAACCATCTCTGAAATGTCTAATTCCAGGTTTCCGGTGCCAATTGACTCTCTGCACACCAGCCCAACTGAGAGTATCGTTGTCCAGTGCCAAAATGGGCGCGTCACTCTTGACGGTGAGTTAATGGGCACCACCCAACTCTTACCGAACCAAATATGTGCTTTCAGGGGAACACTTACTAGATCAACAAACAGGGCCAGTGACCAAGCCGACACAGCAACCCCCAGGCTATTTGACCATCATTGGCACATACAATTGGATAATCTAAATGGAACCCCCTACGACCCTGCAGAGGACATACCAGCTCCTTTGGGTACACCAGACTTCCGGGGCAAAGTCTTTGGCGTAGCCAGCCAGAGAAACCCCGACAGCACAACAAGGGCACATGAAGCAAAAGTGGACACAACATCTGGCCGCTTCACCCCAAAATTGGGCTCCCTAGAAATAACCACTGAATCTGATGACTTTGACCCAAACCAGTCAACAAAATTCACCCCAGTTGGCATTGGAGTTGACAATGAGGCAGATTTTCAGCAATGGTCCTTACCTGACTATTCCGGTCAGTTTACTCATAACATGAACTTAGCCCCAGCTGTCGCCCCCAATTTTCCTGGTGAACAGCTTCTTTTCTTCCGATCACAGCTGCCATCTTCTGGTGGGCGGTCTAACGGGATTCTAGACTGCCTGGTCCCCCAGGAATGGGTTCAACACTTCTACCAAGAATCAGCCCCCGCCCAGACACAGGTGGCCCTGGTTAGGTATGTCAACCCTGACACTGGTAGAGTGCTATTTGAGGCCAAGCTACACAAATTGGGTTTTATGACTATAGCAAAGAATGGTGACTCCCCAATAACTGTCCCTCCAAATGGGTATTTTAGATTTGAATCTTGGGTTAACCCCTTTTACACACTTGCCCCCATGGGAACTGGAAACGGGCGTAGAAGGATTCAATAA

>AY247431|-/II.3|2001|SE|1937-00

ATGAAGATGGCGTCGAATGACGCCACTCCATCTAATGATGGTGCCGCCGGCCTCGTCCCAGAGATCAACAATGAGGCAATGGCGCTAGACCCAGTAGCGGGTGCAGCGATRGCAGCACCCCTCACTGGTCAGCAAAACATAATTGATCCCTGGATTATGAATAATTTTGTGCAAGCACCTGGTGGTGAGTTTACAGTATCCCCTAGGAATTCCCCTGGTGAAGTGCTTCTTAATTTGGAATTGGGCCCAGAAATAAATCCCTATTTGGCCCATCTTGCTAGAATGTATAATGGTTATGCAGGTGGATTTGAAGTGCAGGTAGTCCTGGCTGGAAATGCGTTTACAGCAGGAAAGATAATCTTTGCAGCTATACCCCCTAATTTTCCAATTGATAATCTGAGCGCGGCACAGATCACAATGTGCCCGCATGTGATTGTGGATGTCAGACAGTTGGAACCAGTCAACCTCCCGATGCCTGACGTTCGCAACAACTTCTTCCATTATAATCAAGGGTCTGATTCAAGATTGCGCCTAATTGCAATGCTGTATACACCTCTTAGGGCAAATAATTCTGGGGATGATGTTTTTACTGTGTCTTGTAGAGTGTTGACTAGGCCTAGCCCTGACTTTTCATTTAATTTCCTTGTGCCACCTACTGTGGAGTCAAAGACRAAACCCTTCACTCTCCCTATTCTAACTATCTCTGAAATGTCCAATTCTAGGTTTCCAGTGCCGATTGATTCTCTGCACACCAGCCCAAATRGTAATTTTGTTGTCCAGTGCCAGAATGGGCGCGTCACTCTTGATGGCGAGTTGATGGGTACCACCCAACTCTTGCCGAGTCAAATCTGTGCTTTTAGGGGCGTGCTCACCAGACAAGTAAGCAGGGCCGGTGATCAGATCGACACAGCAACCCCTAGGTYGTTTGGCAATCATTGGCATATACAATTGGATAATTTAAATGGGACTCCTTATGATCCTGCAGAAGACRTACCAGGCCCCCTAGGGACACCAGACTTCCAGGGCATAGTCTTTGGCGTGGCCAGCCAGAGAAACTCCGATGGCACAACTAGAGCACATGAAGCAAAGGTGGACACAAGAACTGGCCGCTTCACCCCAAAATTAGGCTCATTAGTRATATCCACTGATTCTGAAGACTTTCAACAAAACAAACCAACAAGATTCACCCCAGTTGGCGTTGGGGTTGACCGTGAGGAAGAATTTGATCAATGGTCTCTACCCGACTATTCTGGTCCTATCACCCACAACATGAACCTGGCCCCAGCTGTTGCYCCCAACTTCCCTGGTGAGCAGCTCCTTTTCTTCCGCTCGCAGTTACCATYTTCTGGTGGGATATCCAAGGGGATCCTGGACTGCCTGGTCCCCCAAGAATGGGTCCAGCACTTCTAYCAAGAATCAGCCCCCGCCCAAACACAAGTGGCCCTGGTTAGGTATGTCAACCYTGATACTGGTAGAGTACTATTTGAGGCCAAGTTGCACAAATTAGGTTTCATGRCTATAGCTAAGGATGGTGATTCTCCAATAACTGTCCCTCCAAATGGATAYTTTAGGTTTGAATCTTGGGTGAACCCCTTTTATACACTTGCCCCCATGGGAACTGGGAATGGGCGTAGAAGGATTCAATAA

>AY247432|-/II.3|2004|SE|2004-00

ATGAAGATGGCGTCGAATGACGCCACTCCATCTAATGATGGTGCCGCCGGCCTCGTCCCAGAGATCAACAATGAGGCAATGGCGCTAGACCCAGTAGCGGGTGCAGCGATAGCAGCACCCCTCACTGGTCAGCAAAACATAATTGATCCCTGGATTATGAATAATTTTGTGCAAGCACCTGGTGGTGAGTTTACAGTATCCCCTAGGAATTCCCCTGGTGAAGTGCTTCTTAATTTGGAATTGGGCCCAGAAATAAATCCCTATTTGGCCCATCTTGCTAGAATGTATAATGGTTATGCAGGTGGATTTGAAGTGCAGGTAGTCCTGGCTGGAAATGCGTTTACAGCAGGAAAGATAATCTTTGCAGCTATACCCCCTAATTTTCCAATTGATAATCTGAGCGCGGCACAGATCACAATGTGCCCGCATGTGATTGTGGATGTCAGACAGTTGGAACCAGTCAACCTCCCGATGCCTGACGTTCGCAACAACTTCTTCCATTATAATCAAGGGTCTGATTCAAGATTGCGCCTAATTGCAATGCTGTATACACCTCTTAGGGCAAATAATTCTGGGGATGATGTTTTTACTGTGTCTTGTAGAGTGTTGACTAGGCCTAGCCCTGACTTTTCATTTAATTTCCTTGTGCCACCTACTGTGGAGTCAAAGACAAAACCCTTTACTCTCCCTATTCTAACTATCTCTGAAATGTCCAATTCTAGGTTTCCAGTGCCGATTGATTCTCTGCACACCAGCCCAAATGGTAATTTTGTTGTCCAGTGCCAGAATGGGCGCGTCACTCTTGATGGCGAGTTGATGGGTACCACCCAACTCTTGCCGAGTCAAATCTGTGCTTTTAGGGGCGTGCTCACCAGACAAGTAAGCAGGGCCGGTGATCAGATCGACACAGCAACCCCTAGGTCGTTTGGCAATCATTGGCATATACAATTGGATAATTTAAATGGGACTCCTTATGATCCTGCAGAAGACATACCAGGCCCCCTAGGGACACCAGACTTCCAGGGCATAGTCTTTGGCGTGGCCAGCCAGAGAAACTCCGATGGCACAACTAGAGCACATGAAGCAAAGGTGGACACAAGAACTGGCCGCTTCACCCCAAAATTAGGCTCATTAGTAATATCCACTGATTCTGAAGACTTTCAACAAAACAAACCAACAAGATTCACCCCAGTTGGCGTTGGGGTTGACAGTGAGGAAGAATTTGATCAATGGTCCCTACCCGACTATTCTGGTCCTATCACCCACAACATGAACCTGGCCCCAGCTGTTGCTCCCAACTTCCCTGGTGAGCAGCTCCTTTTCTTCCGCTCGCAGTTACCATCTTCTGGTGGGATATCCAAGGGGATCCTGGACTGCCTGGTCCCCCAAGAATGGGTCCAGCACTTCTACCAAGAATCAGCCCCCGCCCAAACACAAGTGGCCCTGGTTAGGTATGTCAACCCTGATACTGGTAGAGTACTATTTGAGGCCAAGTTGCACAAATTAGGTTTCATGACTATAGCTAAGGATGGTGATTCTCCAATAACTGTCCCTCCAAATGGATACTTTAGGTTTGAATCTTGGGTGAACCCCTTTTATACACTTGCCCCCATGGGAACTGGGAATGGGCGTAGAAGGATTCAATAA

>AY247433|-/II.3|2000|SE|2102-00

ATGAAGATGGCGTCGAATGACGCCRCTCCATCTAATGATGGTGCCGCCGGCCTCGTCCCAGAGATCAACAATGAGGCAATGGCGCTAGACCCAGTAGCGGGTGCAGCGATAGCAGCACCCCTCACTGGTCAGCAAAACATAATTGATCCCTGGATTATGAATAATTTTGTGCAAGCACCTGGTGGTGAGTTTACAGTATCCCCTAGGAATTCCCCTGGTGAAGTGCTTCTTAATTTGGAATTGGGYCCAGAAATAAATCCCTATTTGGCCCATCTTGCTAGAATGTATAATGGTTATGCAGGTGGATTTGAAGTGCAGGTAGTCCTGGCTGGAAATGCGTTTACAGCAGGAAAGATAATCTTTGCAGCTATACCCCCTAATTTTCCAATTGATAATCTGAGYGCGGCACAGATCACAATGTGCCCGCATGTGATTGTGGATGTCAGACAGTTGGAACCAGTCAACCTCCCGATGCCTGACGTTCGCAACAACTTCTTCCATTATAATCAAGGGTCTGATTCAAGATTGCGCCTAATTGCAATGCTGTATACACCTCTTAGGGCAAATAATTCTGGGGATGATGTTTTTACTGYGTCTTGTAGAGTGTTGACTAGGCCTAGCCCTGACTTTTCATTTAATTTYCTTGTGCCACCTACTGTGGAGTCAAAGACAAAACCCTTCACTCTCCCTATTCTAACTATCTCTGAAATGTCCAATTCTAGGTTTCCAGTGCCGATTGATTCTCTGCACACCAGCCCAAATARTAATTTTGTTGTCCAGTGCCAGAATGGGCGCGTCACTCTTGATGGCGAGTTGATGGGTACCACCCAACTCTTGCCGAGTCAAATCTGTGCTTTTAGGGGCGTGYTCACCAGACAAGTAAGCAGRGYCGGTGATCAGATCGACACAGCAACCCCTAGGTCGTTTGGCAATCATTGGCATATACAATTGGATAATYTAAATGGGACTCCTTATGAYCCTGCAGAAGACATACCAGGCCCCCTAGGGACACCAGACTTCMAGGGCATAGTCTTTGGCGTGGCCAGCCAGAGAAACTCCGATGGCACAACTAGAGCACATGAAGCAAAGGTGGACACAAGARCTGGCCGCTTCACCCCAAAATTAGGCTCATTAGTAATATCCACTGATTCTGARGACTTTCAACAAAACAAACCAACAAGATTCACCCCAGTTGGCGTTGGGGTTGACMRTGRGGAAGAATTTGATCAATGGTCTCTACCCGACTATTCTGGTCCTATCACCCACAACATGAACCTGGCCCCAGCTGTTGCTCCCAACTTCCCTGGTGAGCAGCTCCTTTTCTTCCGCTCGCAGTTACCATCTTCTGGTGGGATATCCAAGGGGATCCTGGACTGCCTGGTCCCCCAAGAATGGGTCCAGCACTTCTACCAAGAATCAGCCCCCGCCCAARCACAAGTGGCCCTGGTTAGGTATGTCAACCCTGAYACTGGTAGAGTACTATTTGAGGCCAAGTTGCACAAATTAGGTTTCATGACTATAGCTAAGGATGGTGATTCTCCARTAACTGTCCCTCCAAATGGATACTTTAGGTTTGAATCTTGGGTGAACCCCTTTTATACACTTGCCCCCATGGGAACTGGGAATGGGCGTRGAAGGATTCAATAA

>AY247434|-/II.3|2000|SE|2115-00

ATGAAGATGGCGTCGAATGACGCCGCTCCATCTAATGATGGTGCCGCCGGCCTCGTCCCAGAGATCAACAATGAGGCAATGGCGCTAGACCCAGTRGCGGGTGCAGCGATAGCAGCACCCCTCACTGGTCAGCAAAACATAATTGATCCCTGGATTATGAAYAATTTTGTGCAAGCACCTGGTGGTGAGTTTACAGTATCCCCTAGGAATTCCCCTGGTGAAGTGCTTCTTAATTTGGAATTGGGCCCAGAAATAAATCCCTATTTGGCCCATCTTGCTAGAATGTATAATGGTTATGCAGGTGGATTTGAAGTGCAGGTAGTCCTGGCTGGAAATGCGTTTACAGCAGGAAAGATAATCTTTGCAGCTATACCCCCTAATTTTCCAATTGATAATCTGAGYGCGGCACAGATCACAATGTGCCCGCATGTGATTGTGGATGTCAGACAGTTGGAACCAGTCAACCTCCCGATGCCTGACGTTCGCAACAACTTCTTCCATTATAATCAAGGGTCTGATTCAAGATTGCGCCTAATTGCAATGCTGTATACACCTCTTAGGGCAAATAATTCTGGGGATGATGTTTTTACTGTGTCTTGTAGAGTGTTGACTAGGCCTAGCCCTGACTTTTCATTTAATTTCCTTGTGCCACCTACTGTGGAGTCAAAGACAAAACCYTTCACTCTCCCTATTCTAACTATCTCTGAAATGTCCAATTCTAGGTTTCCAGTGCCGATYGATTCTCTGCACACCAGCCCAAATARTAATTTTGTTGTCCAGTGCCAGAATGGGCGCGTCACTCTTGATGGCGAGTTGATGGGTACCACCCAACTCTTGCCGAGTCAAATCTGTGCTTTTAGGGGCGTGCTCACCAGACAARTAAGCAGGGCCGGTGATCAGATCGACACAGCAACCCCTAGGTCGTTTGGCAATCATTGGCATATACAATTGGATAATYTAAATGGGACTCCTTATGATCCTGCAGARGACATACCAGGCCCCCTAGGGACACCAGACTTCAAGGGCATAGTCTTTGGCGTGGCCAGCCAGAGAAACTCCGATGGCACAACTAGAGCACATGAAGCAAAGGTGGACACAAGAACTGGCCGCTTCACCCCAAAATTAGGCTCATTAGTAATATCCACTGATTCTGAAGACTTTCAACAAAACAAACCAACAAGATTCACCCCAGTTGGCGTTGGGGTTGACAATGAGGAAGAATTTGATCAATGGTCTCTACCCGACTATTCTGGTCCTATCACCCACAACATGAACCTGGCCCCAGCTGTTGCTCCCAACTTCCCTGGTGAGCAGCTCCTTTTCTTCCGCTCGCAGTTACCATCTTCTGGTGGGMTATCCAAGGGGATCCTGGACTGCCTGGTCCCCCAAGAATGGGTCCAGCACTTCTACCAAGAATCAGCCCCCGCCCAAACACAAGTKGCCCTGGTTAGGTATGTCAACCCTGATACTGGTAGAGTACTATTTGAGGCCAAGTTGCACAAATTAGGTTTCATGACTATAGCTAAGGATGGTGATTCTCCAGTAACTGTCCCTCCAAATGGATAYTTTAGGTTTGAATCTTGGGTGAACCCCTTTTATACACTTGCCCCCATGGGAACTGGGAATGGGCGTAGAAGGATTCAATAA

>AY247435|-/II.3|2000|SE|2197-00

ATGAAGATGGCGTCGAATGACGCCGCTCCATCTAATGATGGTGCCGCCGGCCTCGTCCCAGAGATCAACAATGAGGCAATGGCGCTAGACCCAGTAGCGGGTGCAGCGATAGCAGCACCCCTCACTGGTCAGCAAAACATAATTGATCCCTGGATTATGAACAATTTTGTGCAAGCACCTGGTGGTGAGTTTACAGTATCCCCTAGGAATTCCCCTGGTGAAGTGCTTCTTAATTTGGAATTGGGCCCAGAAATAAATCCCTATTTGGCCCATCTTGCTAGAATGTATAATGGTTATGCAGGTGGATTTGAAGTGCAGGTAGTCCTGGCTGGAAATGCGTTTACAGCAGGAAAGATAATCTTTGCAGCTATACCCCCTAATTTTCCAATTGATAATCTGAGTGCGGCACAGATCACAATGTGCCCGCATGTGATTGTGGATGTCAGACAGTTGGAACCAGTCAACCTCCCGATGCCTGACGTTCGCAACAACTTCTTCCATTATAATCAAGGGTCTGATTCAAGATTGCGCCTAATTGCAATGCTGTATACACCTCTTAGGGCAAATAATTCTGGGGATGATGTTTTTACTGTGTCTTGTAGAGTGTTGACTAGGCCTAGCCCTGACTTTTCATTTAATTTTCTTGTGCCACCTACTGTGGAGTCAAAGACAAAACCCTTCACTCTCCCTATTCTAACTATCTCTGAAATGTCCAATTCTAGGTTTCCAGTGCCGATTGATTCTCTGCACACCAGCCCAAATAGTAATTTTGTTGTCCAGTGCCAGAATGGGCGCGTCACTCTTGATGGCGAGTTGATGGGTACCACCCAACTCTTGCCGAGTCAAATCTGTGCTTTTAGGGGCGTGCTCACCAGACAAGTARGCAGGGCCGGTGATCAGATCGACACAGCAACCCCTAGGTCGTTYGGCAATCATTGGCATATACAATTGGATAATYTAAATGGGACTCCTTATGATCCTGCAGAAGACATACCAGGCCCCCTAGGGACACCAGACTTCAAGGGCATAGTCTTTGGYGTGGCCAGCCAGAGAAACTCCGATGGCACAACTAGAGCACATGAAGCAAAGGTGGACACAAGAACTGGCCGCTTCACCCCAAAATTAGGCTCATTAGTAATATCCACTGATTCTGAAGACTTTCAACAAAACAAACCAACAAGATTCACCCCAGTTGGCGTTGGGGTTGACAATGAGGAAGAATTTGATCAATGGTCTCTACCCGACTATTCTGGTCCTATCACCCACAACATGAACCTGGCCCCAGCTGTTGCTCCCAACTTCCCTGGTGAGCAGCTCCTTTTCTTCCGCTCGCAGTTACCATCTTCTGGTGGGMTATCCAAGGGGATCCTGGACTGCCTGGTCCCCCAAGAATGGGTCCAGCACTTCTACCAAGAATCAGCCCCCGCCCAAACACAAGTGGCCCTGGTTAGGTATGTCAACCCTGATACTGGTAGAGTACTATTTGAGGCCAAGTTGCACAAATTAGGTTTCATGACTATAGCTAAGGATGGTGATTCTCCARTAACTGTCCCTCCAAATGGATACTTTAGGTTTGARTCTTGGGTGAACCCCTTTTATACACTTGCCCCCATGGGAACTGGGAATGGGCGTAGAAGGATTCAATAA

>AY247436|-/II.3|2000|SE|2366-00

ATGAAGATGGCGTCGAATGACGCCGCTCCATCTAATGATGGTGCCGCCGGCCTCGTCCCAGAGATCAACAATGAGGCAATGGCGCTAGACCCAGTRGCGGGTGCAGCGATAGCAGCACCCCTCACTGGTCAGCAAAACATAAYTGATCCCTGGATTATGAAYAATTTTGTGCAAGCACCTGGYGGWGAGTTTACAGTATCCCCTAGGAATTCCCCTGGTGAAGTGCTTCTTAATTTGGAATTGGGCCCAGAAATAAATCCCTATTTGGCCCATCTTGCTAGAATGTATAATGGTTATGCAGGTGGATTTGAAGTGCAGGTAGTCCTGGCTGGAAATGCGTTTACAGCAGGAAAGATAATCTTTGCAGCTATACCCCCTAATTTTCCAATTGATAATCTGAGTGCGGCACAGATCACAATGTGCCCGCATGTGATTGTGGATGTCAGACAGTTGGAACCAGTCAACCTCCCGATGCCTGACGTTCGCAACAACTTCTTCCATTATAATCAAGGGTCTGATTCAAGATTGCGCCTAATTGCAATGCTGTATACACCTCTTAGGGCAAATAATTCTGGGGATGATGTTTTTACTGTGTCTTGTAGAGTGTTGACTAGGCCTAGCCCTGACTTYTCATTTAATTTYCTTGTGCCACCTACTGTGGAGTCAAAGACAAAACCCTTCACTCTCCCTATTCTAACTATCTCTGAAATGTCCAATTCTAGGTTTCCAGTGCCGATTGATTCTCTGCACACCAGCCCAAATARTAATTTTGTTGTCCAGTGCCAGAATGGGCGCGTCACTCTTGATGGCGAGTTGATGGGTACCACCCAACTCTTGCCGAGTCAAATCTGTGCTTTTAGGGGCGTGCTCRCCAGACAARTAAGCAGGGCCGGTGATCAGATCGACACAGCAACCCCTAGGTCGTTTGGCAATCATTGGCATATACAATTGGATAATYTAAATGGGACTCCTTATGATCCTGCAGAAGACATACCAGGCCCCCTAGGGACACCAGACTTCAAGGGCATAGTCTTTGGWGTGGCCAGCCAGAGAAACTCCGATGGCACAACTAGAGCACATGAAGCAAAGGTGGACACAAGARCTGGCCGCTTCACCCCAAAATTAGGCTCATTAGTAATATCCACTGATTCTGAAGACTTTCAACAAAACAAACCAACAAGATTCACCCCAGTTGGCGTTGGGGTTGACAATGAGGAAGAATTTGATCAATGGTCTCTACCCGACTATTCTGGTCCTATCACCCACAACATGAACCTGGCCCCAGCTGTTGCTCCCAACTTCCCTGGTGAGCAGCTCCTTTTCTTCCGCTCGCAGTTACCATCTTCTGGTGGGATATCCAAGGGGATCCTGGACTGCCTGGTCCCCCAAGAATGGGTCCAGCACTTYTACCAAGAATCAGCCCCCGCCCAAACACAAGTGGCCCTGGTTAGGTATGTCAACCCTGATACTGGTAGAGTACTATTTGAGGCCAAGTTGCACAAATTAGGTTTCATGACTATAGCTAAGGATGGTGATTCTCCARTAACTGTCCCTCCAAATGGATACTYTAGGTTTGAATCTTGGGTGAACCCCTTTTATACACTTGCCCCCATGGGAACTGGGAATGGGCGTAGAAGGATTCAATAA

>AY247437|-/II.3|2001|SE|186-01

ATGAAGATGGCGTCGAATGACGCCGCTCCATCTAATGATGGTGCCGCCGGCCTCGTCCCAGAGATCAACAATGAGGCAATGGCGCTAGACCCAGTGGCGGGTGCAGCGATAGCAGCACCCCTCACTGGTCAGCAAAACATAATTGATCCCTGGATTATGAATAATTTTGTGCAAGCACCTGGTGGTGAGTTTACAGTATCCCCTAGGAATTCCCCTGGTGAAGTGCTTCTTAADTTGGAATTGGGCCCAGAAATAAATCCCTATTTGGCCCATCTTGCTAGAATGTATAATGGTTATGCAGGTGGATTTGAAGTGCAGGTAGTCCTGGCTGGAAATGCGTTTACAGCAGGAAAGATAATCTTTGCAGCTATACCCCCTAATTTTCCAATTGATAATCTGAGTGCGGCACAGATCACAATGTGCCCGCATGTGATTGTGGATGTCAGACAGTTGGAACCAGTCAACCTCCCGATGCCTGACGTTCGCAACAACTTCTTCCATTATAATCAAGGGTCTGATTCAAGATTGCGCCTAATTGCAATGCTGTATACACCTCTTAGGGCAAATAATTCTGGGGATGATGTTTTTACTGTGTCTTGTAGAGTGTTGACTAGGCCTAGCCCTGACTTTTCATTTAATTTTCTTGTGCCACCTACTGTGGAGTCAAAGACAAAACCCTTCACTCTCCCTATTCTAACTATCTCTGAAATGTCCAATTCTAGGTTTCCAGTGCCGATTGATTCTCTGCACACCAGCCCAAATAGTAATTTTGTTGTCCAGTGCCAGAATGGGCGCGTCACTCTTGATGGCGAGTTGATGGGTACCACCCAACTCTTGCCGAGTCAAATCTGTGCTTTTAGGGGCGTGCTCACCAGACAAGTAAGCAGGGCCGGTGATCAGATCGACACAGCAACCCCTAGGTCGTTTGACTATCATTGGCATATACAATTGGATAATYTAAATGGGACTCCTTATGATCCTGCAGAAGACATACCAGGCCCCCTAGGGACACCAGACTTCAAGGGCATAGTCTTTGGCGTGGCCAGCCAGAGARACTCCGATGGCACAACTAGAGCACATGAAGCAAAGGTGGACACAAGAACTGGCCGCTTCACCCCAAAATTAGGCTCATTAGTAATATCCACTGATTCTGAAGACTTTCAACAAAACAAACCAACAAGATTCACCCCAGTTGGCGTTGGGGTTGACAATGAGGAAGAATTTGATCAATGGTCTCTACCCGACTATTCTGGTCCTATCACCCACAACATGAACCTGGCCCCAGCTGTTGCTCCCAACTTCCCTGGTGAGCAGCTCCTTTTCTTCCGCTCGCAGTTACCATCTTCTGGTGGGMTATCCAAGGGGATCCTGGACTGCCTGGTCCCCCAAGAATGGGTCCAGCACTTTTACCAAGAATCAGCCCCCGCCCAAACACAAGTGGCCCTGGTTAGGTATGTCAACCCTGAYACTGGTAGAGTACTATTTGAGGCCAAGTTGCACAAATTAGGTTTCATGACTATAGCTAARGATGGTGATTCTCCARTAACTGTCCCTCCAAATGGATACTTTAGGTTTGAATCTTGGGTGAACCCCTTTTATACACTTGCCCCCAYGGGAACTGGGAATGGGCGTAGAAGGATTCAATAA

>AY247438|-/II.3|2001|SE|500-01

ATGAAGATGGCGTCGAATGACGCCGCTCCATCTAATGATGGTGCCGCCGGCCTCGTCCCAGAGATCAACAATGAGGCAATGGCGCTAGACCCAGTGGCGGGTGCAGCGATAGCAGCACCCCTCACTGGTCAGCAAAACATAATTGATCCCTGGATTATGAATAATTTTGTGCAAGCACCTGGTGGTGAGTTTACTGTATCCCCTAGGAATTCCCCTGGTGAAGTGCTTCTTAATTTGGAATTGGGCCCAGAAATAAATCCCTATTTGGCCCATCTTGCTAGAATGTATAATGGTTATGCAGGTGGATTTGAAGTGCAGGTAGTCCTGGCTGGAAATGCGTTTACAGCAGGAAAGATAATCTTTGCAGCTATACCCCCTAATTTTCCAATTGATAATCTGAGCGCGGCACAGATCACAATGTGCCCGCATGTGATTGTGRATGTCAGACAGTTGGAACCAGTCAACCTCCCGATGCCTGACGTTCGCAACAACTTCTTCCATTATAATCAAGGGTCTGATTCAAGATTGCGCCTAATTGCAATGCTGTATACACCTCTTAGGGCAAATAATTCTGGGGATGATGTTTTTACTGTGTCTTGTAGAGTGTTGACTAGGCCTAGCCCTGACTTCTCATTTAATTTCCTTGTGCCACCTACTGTGGAGTCAAAGACAAAACCCTTCACTCTCCCTATTCTAACTATCTCTGAAATGTCCAATTCTAGGTTTCCAGTGCCGATTGATTCTCTGCACACCAGCCCAAATAATAATTTTGTTGTCCAGTGCCAGAATGGGCGYGTCACTCTTGATGGCGAGTTGATGGGTACCACCCAACTCTTGCCGAGTCAAATCTGTGCTTTTAGGGGCGTGCTCGCCAGACAAATAAGCAGGGCCGGTGATCAGATCGACACAGCARCCCCTAGGTCATTTGGCTATCATTGGCATATACAATTGGATAATCTAAATGGGACTCCTTATGATCCTGCAGAAGACATACCAGGCCCCCTAGGGACACCAGACTTCAAGGGCATAGTCTTTGGTGTGGCCAGCCAGAGAAACTCCGATGGCACAACTAGAGCACATGAAGCAAAGGTGGACACAAGAACYGGCCGCTTCACCCCAAAATTAGGCTCATTAGTAATATYCACTGATTCTGAAGACTTTCAACAAAACAAACCAACAAGATTCACCCCAGTTGGCGTTGGGGTTGACAATGAGGAAGAATTTGATCAATGGTCTCTACCCGACTATTCTGGTCCTATCACCCACAACATGAACCTGGCCCCAGCTGTTGCTCCCAACTTCCCTGGYGAGCAGCTCCTTTTCTTCCGCTCGCAGTTACCATCTTCTGGTGGRAYATCCAAGGGGATCCTGGACTGCCTGGTCCCCCAAGAATGGGTCCAGCACTTCTACCAAGAATCAGCCCCCGCCCAAACACAAGTGGCCCTGGTTAGGTATGTCAACCCTGATACTGGTAGAGTACTATTTGAGGCCAAGTTGCACAAATTAGGTTTCATGACTATAGCTAAGGATGGTGATTCTCCAGTAACTGTCCCTCCAAATGGATACTTTAGGTTTGAATCTTGGGTGAACCCCTTTTATACACTTGCCCCCATGGGAACTGGGAATGGGCGTAGAAGGATTCAATAA

>AY247439|-/II.3|<2003|SE|1157-01

ATGAAGATGGCGTCGAATGACGCCGCTCCATCTAATGATGGTGCCGCCGGCCTCGTCCCAGAGATCAACAATGAGGCAATGGCGCTAGACCCAGTGGCGGGTGCAGCGATAGCAGCACCCCTCACTGGTCAGCAAAACATAATTGATCCCTGGATTATGAATAATTTTGTGCAAGCACCTGGTGGTGAGTTTACTGTATCCCCTAGGAATTCCCCTGGTGAAGTGCTTCTTAATTTGGAATTGGGCCCAGAAATAAATCCCTATTTGGCCCATCTTGCTAGAATGTATAATGGTTATGCAGGTGGATTTGAAGTGCAGGTAGTCCTGGCTGGGAATGCGTTTACAGCAGGAAAGATAATCTTTGCAGCTATACCCCCTAATTTTCCAATTGATAATCTGAGCGCGGCACAGATCACAATGTGCCCGCATGTGATTGTGGATGTCAGACAGTTGGAACCAGTCAACCTCCCGATGCCTGACGTTCGCAACAACTTCTTCCATTATAATCAAGGGTCTGATTCAAGATTGCGCCTAATCGCAATGCTGTATACACCTCTTAGGGCAAATAATTCTGGGGATGATGTTTTTACTGTGTCTTGTAGAGTGTTGACTAGGCCTAGCCCTGACTTCTCATTTAATTTCCTAGTGCCACCTACTGTGGAGTCAAAGACAAAACCCTTCACTCTCCCTATCCTAACTATCTCTGAAATGTCCAATTCTAGGTTTCCAGTGCCGATTGATTCTCTGCACACCAGCCCAAATAATAATTTTGTTGTCCAGTGCCAAAATGGGCGCGTCACTCTTGATGGCGAGTTGATGGGTACCACCCAACTCTTGCCGAGTCAAATCTGTGCTTTTAGGGGCGTGCTCGCCAGACAAATAAACAGGGCCGGTGATCAGATCGACACAACAACCCCTAGGTCATTTGACCATCATTGGCATATACAATTGGATAATCTAAATGGGACTCCTTATGATCCTGCAGAAGACATACCAGGCCCCCTAGGGACACCAGGCTTCAAGGGCATAGTCTTTGGTGTGGCCAGCCAGAGAAACTCCGATGGCACAACTAGAGCACATGAAGCAAAGGTGGACACAAGAACTGGCCGCTTCACCCCAAAATTAGGCTCATTAGTAATATCCACTGATTCTGAAGACTCTCAACAAAACAAACCAACAAGGTTCACCCCAGTTGGCGTTGGGGTTGACAATGAGGAAGAATTTGATCAATGGTCTCTACCCAACTATTCTGGTCCTATCACCCACAACATGAACCTGGCCCCAGCTGTTGCTCCCAACTTCCCTGGTGAGCAGCTCCTTTTCTTCCGCTCGCAGTTACCATCTTCTGGTGGGACATCCAAGGGGATCCTGGACTGCCTGGTCCCCCAAGAATGGGTCCAGCACTTCTACCAAGAATCAGCCCCCGCCCAAACACAAGTGGCCCTGGTTAGGTATGTCAACCCTGATACTGGTAGAGTACTATTTGAGGCCAAGTTGCACAAATTAGGTTTCATGACTATAGCTAAAGATGGTGATTCTCCAATAACTGTCCCTCCAAATGGATACTTTAGGTTTGAATCTTGGGTGAACCCCTTTTATACACTTGCCCCCATGGGAACTGGGAATGGGCGTAGAAGGATTCAATAA

>AY247440|-/II.3|2001|SE|1312-01

ATGAAGATGGCGTCGAATGACGCCGCTCCATCTAATGATGGTGCCGCCGGCCTCRTCCCAGAGATCAACAATGAGGCAATGGCGCTAGACCCAGTAGCGGGTGCAGCGATAGCAGCACCCCTCACTGGTCAGCAAAATATAATTGATCCCTGGATTATGAACAATTTTGTGCAAGCACCTGGTGGTGAGTTTACAGTATCCCCTAGGAATTCCCCTGGTGAAGTGCTTCTTAATTTGGAATTGGGCCCAGAAATAAATCCCTATTTGGCCCATCTTGCTAGAATGTATAATGGTTATGCAGGTGGATTTGAAGTGCAGGTAGTCCTGGCTGGAAATGCGTTTACAGCAGGAAAGATAATCTTTGCAGCTATACCCCCTAATTTTCCAATTGATAATCTGAGTGCGGCACAGATCACAATGTGCCCGCATGTGATTGTGGATGTCAGACAGTTGGAACCAGTCAACCTCCCGATGCCTGACGTTCGCAACAACTTCTTCCATTATAATCAAGGGTCTGATTCAAGATTGCGCCTAATTGCAATGCTGTATACACCTCTTAGGGCAAATAATTCTGGGGATGATGTTTTTACTGTGTCTTGTAGAGTGTTGACTAGGCCTAGCCCTGACTTYTCATTTAATTTTCTTGTGCCACCTACTGTGGAGTCAAAGACAAAACCCTTCACTCTTCCTATTCTAACTATCTCTGAAATGTCCAATTCTAGGTTTCCAGTGCCGATTGATTCTCTGCACACCAGCCCAAATAGTAATTTTGTTGTCCAGTGCCAGAATGGGCGCGTCACTCTTGATGGCGAGTTGATGGGTACCACCCAACTCTTGCCGAGTCAAATCTGTGCTTTTAGGGGCGTGCTCACCAGACAAGTAAGCAGGGCCGGTGATCAGATCGACACAGTAGACCCTAGGTCGTTTGACTATCAYTGGCATRTACAATTGGATAATTTAAATGGGACTCCTTATGATCCTGCAGAAGACATACCAGGCCCCCTAGGGACACCAGACTTCAAGGGCATAGTCTTTGGCGTGGCCAGCCAGAGAAACTCCGATGGCACAACTAGAGCACATGAAGCAAAGGTGRAYACAAGAACTGGCCGCTTCACCCCAAAATTAGGCTCATTAGTAATATTCACTGATTCTGAAGACTTCCAACAAAACAAACCAACAAGATTCACCCCAGTTGGCGTTGGGGTTGACAATGAGGAAGAATTTGATCAATGGTCTCTACCCRACTATTCTGGTCCTATCACCCACAACATGAACCTGGCCCCAGCTGTTGCTCCCAACTTCCCTGGTGAGCAGCTCCTTTTCTTCCGCTCGCAGTTACCATCTTCTGGTGGGACATCCAAGGGGATCCTGGACTGYCTGGTCCCYCAAGAATGGGTCCAGCACTTCTACCAAGAATCAGCCCCCGCCCAAACACAAGTGGCCCTGGTTAGGTATGTCAACCCTGATACTGGTAGAGTACTATTTGAGGCCAAGTTGCACAAATTAGGTTTCATGACTATAGCTAAAGATGGTGATTCTCCAATAACTGTCCCTCCAAATGGATACTTTAGGTTTGAATCTTGGGTGAACCCCTTTTATACACTTGCCCCCATGGGAACTGGGRATGGGCGTAGAAGGATTCAATAA

>AY247441|-/II.3|2001|SE|1464-01

ATGAAGATGGCGTCGAATGACGCCGCTCCATCTAATGATGGTGCCGCCGGCCTCGTCCCAGAGATCAACAATRAGGCAATGGCGCTAGACCCAGTAGCGGGTGCAGCGATAGCAGCACCCCTCACTGGTCAGCAAAACATAATTGATCCCTGGATTATGAACAATTTTGTGCAAGCACCTGGTGGTGAGTTTACAGTATCCCCTRGGAATTCCCCTGGTGAAGTGCTTCTTAATTTGGAATTGGGCCCAGAAATAAATCCCTATTTGGCCCATCTTGCTAGAATGTATAATGGTTATGCAGGTGGATTTGAAGTGCAGGTAGTCCTGGCTGGAAATGCGTTTACAGCAGGRAAGATAATCTTTGCAGCTATACCCCCTAATTTTCCAATTGATAATCTGAGTGCGGCACAGATCACAATGTGYCCGCATGTGATTGTGGATGTCAGACAGTTGGAACCAGTCAACCTCCCGATGCCTGACGTTCGCAACAACTTCTTCCATTATAATCAAGGGTCTGATTCAAGATTGYGCCTAATTGCAATGCTGTATACACCTCTTAGGGCAAATAATTCTGGGGATGATGTYTTYAYTGTKTCTTGTAGAGTGTTGACTAGGCCTAGCCCTGACTTYTCATTTAATTTTCTTGTGCCACCTACTGTGGAGTCAAAGACAAAACCCTTCACTCTTCCTATTCTAACTATCTCTGAAATGTCCAATTCTAGGTTTCCAGTGCCGATTGAYTCTCTGCACACCAGCCCAAATAGTAATTTTGTTGTYCAGTGCCAGAATGGGCGCGTCACTCTTGATGGCGAGTTGATGGGTACCACCCAACTCTTGCCGAGTCAAATCTGTGCTTTTAGGGGCGTRCTCACCAGACAAGTAAGCAGGGCCGGTGATCAGATCGACACAGTAGACCCTAGGTCGTTTGACTATCATTGGCATATACAATTGGATAATTTAAATGGGACTCCTTATGATCCTGCAGAAGACATACCAGGCCCCCTAGGGACACCAGACTTCAAGGGCATAGTCTTTGGCGTGGCCAGCCAGAGAAACTCCGATGGCACAACTAGAGCACATGAAGCAAAGGTGGACACAAGAGCTGGCCGCTTCACCCCAAAATTAGGCTCATTAGTAATATTCACTGATTCTGAAGACTTCCAAGAAAAYAAACCAACAAGATTCACCCCAGTTGGCGTTGGGGTTGACAATGAGGAAGAATTTGATCAATGGTCTCTACCCAACTATTCTGGTCCTATCACCCACAACATGAACCTGGCCCCAGCTGTTGCTCCCAACTTCCCTGGTGAGCAGCTCCTTTTCTTCCGCTCGCAGTTACCATCTTCTGGTGGGACATCCAAGGGGATCCTGGACTGCCTGGTCCCCCAAGAATGGGTCCAGCACTTCTACCAAGAATCAGCCCCCGCCCAAACACAAGTGGCCCTGGTTAGGTATGTCAACCCTGACACTGGTAGAGTACTATTTGAGGCCAAGTTGCACAAATTAGGTTTCATGACTATAGCTAAGGATGGTGATTCTCCAGTAACTGTCCCTCCAAATGGATACTTTAGGTTTGARTCTTGGGTGAACCCCTTTTATACACTTGCCCCCATGGGAACTGGGAATGGGCGTAGAAGGATTCAATAA

>AY247442|-/II.3|2001|SE|1581-01

ATGAAGATGGCGTCGAATGACGCCGCTCCATCTAATGATGGTGCCGCCGGCCTCGTCCCAGAGATCAACAATGAGGCAATGGCGCTAGACCCAGTAGCGGGTGCAGCGATAGCAGCACCCCTCACTGGTCAGCAAAACATAATTGATCCCTGGATTATGAACAATTTTGTGCAAGCACCTGGTGGTGAGTTTACAGTATCCCCTAGGAATTCCCCTGGTGAAGTGCTTCTTAATTTGGAATTGGGCCCAGAAATAAATCCCTATTTGGCCCATCTTGCTAGAATGTATAATGGTTATGCAGGTGGATTTGAAGTGCAGGTAATCCTGGCTGGAAATGCGTTTACAGCAGGAAAGATAATCTTTGCAGCTATACCCCCTAATTTTCCAATTGATAATCTGAGTGCGGCACAGATCACAATGTGCCCGCATGTGATTGTGGATGTCAGACAGTTGGAACCAGTCAACCTCCCGATGCCTGACGTTCGCAACAACTTCTTCCATTATAATCAAGGGTCTGAWTCAAGATTGCGCCTAATTGCAATGCTGTATACACCTCTTAGGGCAAATAATTCTGGGGATGATGTTTTTACTGTGTCTTGTAGAGTGTTGACTAGGCCTAGCCCTGACTTTTCATTTAATTTTCTTGTGCCACCTACTGTGGAGTCAAAGACAAAACCCTTCACTCTTCCTATTCTAACTATCTCTGAAATGTCCAATTCTAGGTTTCCAGTGCCGATTGATTCTCTGCACACCAGCCCAAATAGTAATTTTGTTGTCCAGTGCCAGAATGGGCGCGTCACTCTTGATGGCGAGTTGATGGGTACCACCCAACTCTTGCCGAGTCAAATCTGTGCTTTTAGGGGCGTGCTCATCAGACAAGTAAGCAGGGCCGGTGATCAGATCGACACAGTAGACCCTAGGTCGTTTGACTATCATTGGCATATACAATTGGATAATCTAAATGGGACTCCTTATGATCCTGCAGAAGACATACCAGGCCCCCTAGGGACACCAGACTTCAAGGGCATAGTCTTTGGTGTGGCCAGCCAGAGAAACTCCGATGGCACAACTAGACCACATGAAGCAAAGGTGGACACAAGAACTGGCCGCTTCACCCCAAAATTAGGCTCATTAGTAATATTCACTGATTCTGAAGACTTCCAAGAAAACAAACCAACAAGATTCACCCCAGTTGGCGTTGGGGTTGACAATGAGGAAGAATTTGATCAATGGTCTCTACCCAACTATTCTGGTCCTATCACCCACAAYATGAACCTGGCCCCAGCTGTTGCTCCCAACTTCCCTGGTGAGCAGCTCCTTTTCTTCCGCTCGCAGTTACCATCTTCTGGTGGGACATCCAAGGGGATCCTGGACTGCCTGGTCCCCCAAGAATGGGTCCAGCACTTCTACCAAGAATCAGCCCCCGCCCAAACACAAGTGGCCCTGGTTAGGTATGTCAACCCTGATACTGGTAGAGTACTATTTGAGGCCAAGTTGCACAAATTAGGTTACATGACTATRGCTAAAGATGGTGATTCTCCAATAACTGTCCCTCCAAATGGATAYTTTAGGTTTGAATCTTGGGTGAACCCCTTTTATACACTTGCCCCCATGGGAACTGGGAATGGGCGTAGAAGGATTCAATAA

>AY588132|II.a/II.3|<2004|AU|Sydney 2212

ATGAAGATGGCGTCGAATGACGCCACTCCATCCAATGATGGTGCCGCCGGCCTCGTCCCAGAGATCAACAATGAGGCAATGGCGCTAGATCCAGTGGCGGGTGCAGCGATAGCAGCACCCCTCACTGGTCAGCAAAACATAATTGATCCCTGGATTATGAATAATTTTGTGCAAGCACCTGGTGGTGAGTTTACAGTATCCCCTAGGAATTCCCCTGGTGAAGTGCTTCTTAATTTGGAATTGGGCCCAGAAATAAACCCCTATTTGGCCCATCTTGCTAGAATGTATAATGGTTATGCAGGTGGATTTGAAGTGCAGGTAGTCCTGGCTGGAAATGCGTTTACAGCAGGAAAGATAATCTTTGCAGCTATACCCCCTAATTTTCCAATTGATAATCTGAGCGCAGCACAGATCACAATGTGCCCGCATGTGATTGTGGATGTCAGACAGTTGGAACCGGTCAACCTTCCGATGCCTGACGTTCGCAACAACTTCTTTCATTACAATCAAGGGTCTGATTCGAGATTGCGCTTAATTGCAATGCTGTATACACCTCTTAGGGCAAATAATTCTGGGGATGATGTTTTTACTGTGTCTTGTAGAGTGTTGACTAGGCCTAGCCCTGACTTTTCATTTAATTTCCTTGTGCCACCTACTGTGGAGTCAAAGACAAAACCCTTCACCCTCCCTATTCTGACTATCTCTGAAATGTCCAATTCTAGGTTTCCAGTGCCGATTGATTCTCTGCACACCAGCCCAACTGAGAATATTGTTGTCCAGTGCCAAAATGGGCGCGTCACTCTTGATGGTGAGTTGATGGGCACCACCCAACTCTTGCCGAGTCAAATCTGTGCTTTTAGGGGCGTGCTCACCAGATCAACAAGCAGGGCCAGTGATCAGGCCGACACAGTAACCCCTAGGTTGTTTAATTATTATTGGCATATACAATTGGATAATCTAAATGGGACTCCTTATGATCCTGCAGAAGACATACCAGGCCCCCTAGGGACACCAGATTTCCGGGGCAAAGTCTTTGGCGTGGCCAGCCAGAGAAACCCCGACAGCACAACTAGAGCACATGAAGCAAAGGTGGACACAACAGCTGGTCGTTTCACCCCAAAATTAGGCTCATTAGAAATATCCACTGAATCTGATGACTTTGACCAAAACCAACCAACAAGATTCGCCCCAGTTGGCATTGGGGTTGACCGTGAGGCAGGCTTTCAACAATGGTCTTTGCCCGACTATTCTGGTCAGTTCTCCCGCATCATGAGCTTGGCCCCAGCTGTTGCTCCCATCTTCCCTGGTGAGCAGCTCCTTTTCTTCCGCTCACAGTTACCATCTTCTGGTGGGCGATCCAACGGGATTTTAGACTGCCTGGTCCCCCAAGAATGGGTTCAGCACTTCTACCAAGAATCGGCCCCCGCCCAAACACAAGTGGCCCTGGTTAGGTATGTCAATCCTGACACTGGTAGAGTGCTATTTGAGGCCAAGCTGCACAAATTAGGTTTCATGACTATAGCTAAGAATGGTGACTCTCCAATAACCGTCCCTCCAAATGGATACTTTAGGTTTGAATCTTGGGTGAACCCCTTTTATACACTTGCCCCCATGGGAACTGGGAATGGGCGTAGAAGGATTCAATAA

>AY652979|II.21/II.3|2003|US|Paris Island/2003

ATGAAGATGGCGTCTAAGGACGCCACTCCATCTAATGATGGTGCCGCCGGCCTCGTCCCAGAGATCAACAATGAGGCAATGGCGCTAGATCCAGTGGCGGGTGCAGCGATAGCAGCACCCCTCACTGGTCAGCAAAATATAATTGATCCCTGGATTATGAATAATTTTGTGCAAGCACCTGGTGGTGAGTTTACAGTATCCCCTAGGAATTCCCCTGGTGAAGTGCTTCTTAATTTGGAATTGGGCCCAGAAATAAATCCCTATTTGGCCCATCTTGCCAGAATGTATAATGGTTATGCAGGTGGATTTGAAGTGCAGGTAGTCCTAGCTGGAAATGCGTTTACAGCAGGAAAGATAATCTTTGCAGCTATACCCCCCAATTTTCCAATTGATAATCTAAGCGCAGCACAGATCACAATGTGCCCACATGTGATTGTGGACGTCAGACAGTTGGAACCAGTCAACCTCCCGATGCCTGACGTTCGCAACAACTTCTTCCATTACAATCAAGGGTCTGATTCGAGATTGCGCTTAATTGCAATGCTGTATACACCTCTTAGGGCAAATAATTCTGGTGATGATGTTTTTACTGTGTCTTGTAGAGTGCTGACTAGGCCTAGCCCTGACTTCTCATTTAATTTCCTTGTGCCACCTACTGTGGAGTCAAAGACAAAACCCTTCACCCTCCCTATTCTGACTATCTCCGAAATGTCCAATTCTAGGTTTCCAGTGCCGATTGACTCTCTGCACACCAGCCCAACTGAGAATATTGTTGTCCAGTGCCAAAATGGGCGCGTCACTCTTGATGGTGAGTTGATGGGCACCACCCAACTCTTACCTAGTCAAATCTGTGCTTTCAGGGGCGTGCTCACCAGATCAACAAGCAGGGCCAGTGATCAGGCCGACACAGCAACCCCTAGGTTGTTTAATTATTATTGGCACATACAATTGGATAATCTAAATGGGACTCCCTATGATCCTGCAGAAGATATACCAGGCCCCCTAGGGACGCCAGATTTCCGGGGCAAAGTCTTTGGCGTGGCCAGCCAGAGAAACCCCGACAGCACAACTAGAGCACATGAAGCAAAGGTGGACACAACAGCTGGTCGTTTCACCCCAAAACTAGGCTCATTAGAGATATCCACTGAATCTGGTGACTTTGACCAAAACCAACCAACAAGATTCACCCCAGTTGGCATTGGGGTTGACAATGAAGCAGACTTTCAACAATGGTCTTTACCTGACTATTCTGGTCAGTTCACCCACAACATGAACTTAGCCCCAGCTGTTGCTCCCAACTTCCCTGGTGAGCAGCTCCTTTTCTTCCGCTCACAGTTACCATCCTCTGGTGGGCGATCCAACGGGATTCTAGACTGCCTGGTCCCCCAAGAGTGGGTTCAGCACTTCTACCAAGAATCGGCCCCCTCTCAAACTCAAGTGGCCCTGGTTAGGTATGTCAACCCTGACACTGGTAGAGTATTATTTGAGGCCAAGCTGCACAAACTAGGTTTCATGACTATAGCTAAGAATGGTGACTCTCCAATAACTGTCCCTCCAAATGGATACTTTAGGTTTGAATCTTGGGTGAACCCATTTTATACACTTGCCCCCATGGGAACTGGGAATGGGCGTAGAAGGATTCAATAA

>AY845056|II.21/II.3|2002|AU|C14

ATGAAGATGGCGTCGAATGACGCCACTCCATCTAATGATGGTGCCGCCGGCCTCGTCCCAGAGATCAACAATGAGGCAATGGCGCTAGATCCAGTGGCGGGTGCAGCGATAGCAGCACCCCTCACTGGTCAGCAAAATATAATTGATCCCTGGATTATGAATAATTTTGTGCAAGCACCTGGTGGTGAGCTTACAGTATCCCCTAGGAATTCCCCTGGTGAAGTGCTTCTTAATTTGGAATTGGGCCCAGAAATAAACCCCTATTTGGCCCATCTTGCTAGAATGTATAATGGTTATGCAGGTGGATTTGAAGTGCAGGTAGTCCTAGCTGGAAATGCGTTTACAGCAGGAAAGATAATCTTTGCAGCTATACCCCCTAATTTTCCAATTGATAATCTAAGTGCAGCACAGATCACAATGTGCCCACATGTGATTGTGGATGTCAGACAGTTGGAACCGGTCAACCTCCCGATGCCTGACGTTCGCAACAACTTCTTTCATTACAATCAAGGGTCTGATACGAGATTGCGCTTAATTGCAATGCTGTATACACCTCTTAGGGCAAATAGTTCTGGGGATGATGTTTTTACTGTGTCTTGTAGGGTGCTGACTAGGCCTAGCCCTGATTTTTCATTTAATTTCCTTGTGCCACCTACTGTGGAGTCAAAGACAAAGCCCTTCACCCTCCCTATCCTGACTATCTCTGAAATGTCCAATTCTAGGTTTCCAGTGCCGATTGATTCTCTGCACACCAGCCCAACTGAGAATATTGTTGTCCAGTGCCAAAATGGGCGCGTCACTCTTGATGGTGAGTTGATGGGCACCACCCAACTTTTACCTAGTCAAATCTGTGCTTTCAGGGGCATGCTCACCAGGTCAACAAGCAGGGCCAGTGATCAGGCCGACACAGCAGCCCCTAGGTTGTTTAATTATCATTGGCATATACAGTTGGATAATCTAAATGGGACTCCTTATGATCCTGCAGAGGACATACCAGGCCCCCTAGGGACACCAGATTTCCGGGGCAAAGTCTTTGGTGTGGCCAGCCAGAGAAATCCCGACAGCACAACTAGAGCGCATGAAGCAAAGGTGGACACAACAGCTGGTCGTTTCACCCCAAAATTAGGCTCATTAGAGATATCCACCGAATCTGGTGACCTTGATCAAAACCAACCAACAAAATTCACCCCAGTTGGCATTGGGGTTGACCATGAGGAAGACTTTCAACAATGGTCCTTACCCGACTATTCTGGTCAGTTCACCCACAACATGAACTTAGCCCCAGCTGTTGCTCCCAACTTCCCTGGTGAGCAGCTCCTTTTCTTCCGCTCACAGTTACCATCCTCTGGTGGGCGATCCAACGGGATTCTAGACTGCCTGGTCCCCCAGGAATGGGTTCAGCACTTCTACCAAGAATCGGCCCCCGCCCAAACTCAAGTGGCCCTGGTTAGGTATGTCAACCCTGACACTGGTAGAGTATTATTTGAGGCCAAGCTGCACAAATTAGGTTTCATGACTATAGCTAAGAATGGTGACTCCCCAATAACTGTCCCTCCAAATGGATACTTTGGGTTTGAATCTTGGGTGAACCCATTTTATACACTTGCCCCCATGGGAACTGGGAATGGGCGTAGAAGGATTCAATAA

>DQ078841|-/II.3|<2005|AU|Sydney715D

ATGAAGATGGCGTCGAGTGACGCCACTCCATCTAATGATGGTGCCGCCGGCCTCGTCCCAGAGATCGACAATGAGGCAATGGCGCTAGATCCAGTGGCGGGTGCAGCGATAGCAGCACCCCTCACTGGTCAGCAAAATATAATTGATCTCTGGATTATGAATAATTTTGTGCAAGCACCTGGTGGTGAGTTTACAGTATCCCCTAGAAATTCCCCTGGTGAAGTGCTTCTTAATTTGGAATTGGGCCCAGAAATAAATCCCTATTTGGCTCATCTTGCTAGAATGCATAATGGTTATGCAGGTGGATTTGAAGTGCAGGTAGTCCTAGCTGGAAATGCGTTTACAGCAGGAAAGATAATCTTTGCAGCTATACCCCCTAATTTTCCAATTGATAATTTGAGTGCAGCACAGATCACAATGTGCCCACATGTGATTGTGGATGTCAGACAGTTGGAACCGGTCAACCTCCCGATGCCTGACGTTCGCAACAACTTCTTTCATTATAATCAAGGGTCTGATTCGAGATTGCGCTTAATTGCAATGCTGTATACACCTCTTAGGGCAAATAATTCTGGGGATGATGTTTTCACTGTGTCTTGTAGAGTGCTGACTAGGCCTAGCCCTGACTTTTCATTTAATTTCCTTGTGCCACCTACTGTGGAGTCAAAGACAAAACCCTTCACCCTCCCTATTCTGACTATCTCCGAAATGTCCAATTCTAGGTTTCCAGTGCCGATTGATTCTTTGCTCACCAGCCCAACTGAGAATATTGTTGTCCAGTGCCAAAATGGGCGCGTCACTCTTGATGGTGAGTTGATGGGCACCACCCAACTCTTACCTAGTCAAATTTGTGCTTTTAGGGGCGTGCTCACCAGATCAACAAGCAGGGCCAGTGACCAAGCCGACACAGCAACCCCTAGATTGTTTAATTACTATTGGCACATACAATTGGATAATCTAAATGGGACCCCTTATGACCCTGCAGAAGACATACCAGGCCCCCTAGGGACACCAGATTTTCGGGGCAAGGTCTTTGGCGTGGCCAGCCAGAGAAATCCCGACAGCACAACTAGAGCACATGAGGCAAAGGTGGACACAACAGCTGGTCGTTTCACCCCAAAACTAGGCTCATTAGAGATATCCACTGAATCTGATGACTTTGATCAAAACCAACCAACAAGATTCACCCCAGTTGGCATTGGGGTTGACCGTGAGGCAGACTTTCAACAATGGTCTTTACCCGACTATTCTGGTCAGTTCACCCACAACATGAACTTAGCCCCAGCTGTTGCTCCCAACCTTCCTGGTGAACAGCTCCTTTTCTTCCGCTCGCAGCTACCATCCTCTGGTGGGCGATCCACCGGGATTCTAGACTGCCTGGTCCCCCAAGAATGGGTTCAGCACTTCTACCAAGAATCGGCCCCCGCCCAAACTCAAGTGGCCCTGGTTAGGTATATCAACCCTGACACTGGTAGAGTATTATTTGAGGCCAAGCTGCACAAATTAGGTTTCATGACTATAGCTAAGAATGGTGACTCTCCAATAACTGTCCCTCCAAATGGGTACTTTAGGTTTGAATCTTGGGTGAACCCCTTTTATACACTTGCCCCCATGGGAACTGGGAGTGGGCGTAGAAGGATTCAATAA

>DQ093062|-/II.3|<2005|JP|42447

ATGAAGATGGCGTCGAATGACGCTGCTCCATCTAATGATGGTGCCGCCGGCCTCGTCCCAGAGATCAACAATGAGGCAATGGCGCTAGAGCCAGTGGCGGGTGCAGCGATAGCAGCGCCCCTCACTGGCCAGCAAAATATAATTGATCCCTGGATTATGAATAATTTTGTGCAAGCACCTGGTGGTGAGTTTACAGTGTCACCTAGGAATTCCCCTGGTGAAGTGCTTCTCAATTTGGAATTAGGTCCAGAAATAAATCCCTATTTGGCTCATCTTGCTAGAATGTATAATGGTTATGCAGGTGGGTTTGAAGTGCAAGTGGTCCTAGCTGGAAATGCGTTTACAGCAGGAAAGGTTATCTTTGCAGCTATACCCCCTAATTTCCCTATTGACAATCTGAGCGCGGCACAGATCACAATGTGCCCGCACGTGATTGTGGATGTCAGGCAGTTAGAACCAATCAATCTCCCGATGCCTGATGTCCGCAACAATTTCTTTCATTATAATCAAGGTTCTGATTCAAGATTGCGTTTGATTGCAATGTTGTATACACCTCTTAGGGCAAATAATTCTGGAGATGATGTTTTCACTGTGTCTTGTAGGGTGTTAACTAGGCCCAGTCCTGATTTCTCATTCAATTTTCTTGTCCCACCCACTGTGGAATCAAAGACAAAGCTTTTTACCCTCCCCATTTTAACCATCTCTGAAATGTCCAATTCCAGGTTTCCGGTTCCAATTGATTCTTTACACACCAGCCCAACTGAGAATATAGTTGTCCAGTGCCAAAATGGGCGCGTCACTCTTGACGGTGAGTTAATGGGCACCACCCAACTCTTACCGAGCCAAATATGTGCTTTCAGGGGCACACTCACTAGACCAACAAGCAGGGCCAGTGACCAAGCCGACACACCAACCCCCAGGCTATTCAACCATCGTTGGCACATACAATTGGACAATCTAAATGGAACTCCCTATGACCCCGCGGAGGACATACCAGCTCCTTTGGGCACACCAGACTTCCGGGGCAAGGTCTTTGGCGTGGCCAGCCAGAGAAACCCCGACAGCACAACAAGGGCACATGAAGCAAAAGTGGACACAACATCTGGCCGCTTCACCCCAAAATTGGGTTCCTTAGAAATAACCACTGAGTCTGATGACTTTGACACAAACCAGTCAACAAAATTCACCCCAGTTGGCATCGGGGTTGACAATGAGGCAGAATTTCAGCAATGGTCCTTACCCAACTATTCTGGTCAGTTCACTCATAATATGAACTTAGCCCCAGCTGTCGCCCCCAATTTTCCTGGTGAGCAGCTACTTTTCTTCCGATCACAGCTGCCATCCTCTGGTGGGCGGTCTAACGGGGTTCTAGACTGCCTGGTCCCCCAGGAATGGGTACAACACTTTTACCAAGAATCAGCCCCCGCCCAAACACAGGTGGCCCTGGTTAAGTATGTCAACCCTGACACTGGCAGAGTGCTATTTGAGGCTAAGCTACACAAGTTGGGTTTTATGACTATAGCAAAGAATGGTGACTCCCCAATAACTGTCCCTCCAAATGGATACTTTAGATTCGAATCTTGGGTTAACCCCTTTTACACACTTGCCCCCATGGGAACTGGAAACGGGCGTAGAAGGATTCAATAA

>DQ093063|-/II.3|<2005|JP|336

ATGAAGATGGCGTCGAATGACGCTGCTCCATCTAATGATGGTGCCGCCGGCCTCGTCCCAGAGATCAACAATGAGGCAATGGCGCTAGACCCAGTGGCGGGTGCAGCAATAGCAGCACCCCTTACTGGCCAGCAAAATATAATTGATCCCTGGATTATGAATAACTTTGTGCAAGCACCTGGTGGTGAGTTCACAGTGTCACCTAGAAATTCTCCTGGTGAAGTGTTACTTAATTTGGAATTGGGTCCAGAAATAAATCCCTACTTGGCCCATCTTGCTAGAATGTACAATGGCTATGCAGGTGGGTTTGAAGTACAGGTAGTCCTGGCTGGAAATGCATTTACAGCAGGAAAGGTGATCTTTGCAGCCATACCCCCCAACTTCCCTATTGATAATCTGAGCGCAGCACAGATCACAATGTGCCCGCATGTGATTGTGGATGTCAGGCAACTGGAACCAATCAATCTCCCAATGCCTGATGTCCGCAACAATTTCTTTCATTATAATCAAGGGTCTGATTCAAGATTACGCTTAATTGCAATGCTGTATACACCCCTTAGAGCAAATAATTCTGGGGATGATGTTTTCACTGTGTCTTGTAGAGTTTTAACCAGGCCTAGCCCTGATTTCTCATTTAATTTTCTTGTCCCACCCACTGTGGAATCAAAGACAAAACCTTTCACCCTCCCCATTTTGACCATCTCTGAAATGTCCAATTCCAGGTTTCCAGTGCCAATCGACTCCCTACACACCAGCCCGACCGAGAGTGTCGTTGTCCAATGCCAAAATGGGCGCGTCACCCTCGATGGTGAGCTAATGGGTACCACCCAACTCTTGCCAAGTCAAATATGTGCTTTCAGGGGCACACTTACTAGACCAACAAATAGGGCCAGTGACCAAGCTGACACAGCAACCCCTAGGCTGTTCAACCATCAGTGGCACATACAATTGGATAATCTAAATGGAACCCCCTATGATCCCGCAGAGGACATACCAGCTCCCTTGGGTACACCAGACTTCCGGGGCAAGGTCTTTGGTGTGGCCAGTCAAAGAGACCCCGACGGCACAACAAGAGCACATGAAGCAAAGGTGGACACAACATCTGGTCGCTTCACTCCAAAATTGGGTTCCCTAGAAATAACCACTGAATCTGATGATTTTAACCAAAATAAGCCAACGAGATTCACCCCAGTTGGCATTGGAGTTGACAATGAGGCAGATTTCCAACAATGGATCTTACCTGATTACTCCGGCCAGTTTACTCATAATATGAATTTGGCCCCAGCTGTCGCCCCCAATTTCCCTGGTGAGCAACTTCTTTTCTTCCGCTCACAGTTGCCATCTTCTGGTGGGCGGTCGAACGGGATTCTAGACTGCCTGGTCCCCCAAGAATGGGTTCAACACTTCTACCAGGAATCAGCCCCCGCCCAGACACAGGTGGCCCTGGTTAGATATGTCAACCCTGACACTGGTAGGGTGCTATTTGAGGCCAAGCTTCACAAAATGGGCTTCATGACTATAGCAAAGAATGGTGATTCTCCAATAACTGTCCCTCCAAATGGATACTTTAGATTTGAATCTTGGGTGAACCCCTTTTATACACTCGCCCCCATGGGAACTGGCAAGGGGCGTAGAAGGATTCAATAA

>DQ093066|-/II.3|<2005|JP|1152

ATGAAGATGGCGTCGAATGACGCTGCTCCATCTAACGATGGTGCCGCCGGCCTCGTCCCAGAGATCAACAATGAGGCAATGGCGCTAGACCCAGTGGCGGGTGCAGCGATAGCAGCGCCCCTCACTGGACAGCAAAACATAATTGATCCCTGGATTATGAATAATTTTGTGCAAGCACCTGGTGGTGAGTTTACAGTGTCACCTAGGAATTCCCCTGGTGAAGTGCTTCTAAACTTAGAATTAGGCCCAGAAATAAACCCCTATTTGGCTCACCTTGCTAGGATGTATAATGGTTATGCAGGTGGGTTTGAAGTGCAGGTAGTCCTGGCTGGAAATGCGTTTACAGCAGGAAAGGTGATCTTTGCAGCTATACCCCCTAATTTTCCAATTGATAATCTGAGCGCCGCACAGATTACAATGTGCCCTCATGTGATTGTGGACGTTAGGCAGTTGGAACCAATCAACCTTCCGATGCCTGATGTTCGCAACAATTTCTTTCATTATAATCAAGGGTCTGATTCAAGGTTGCGCTTAATTGCAATGTTGTATACACCTCTTAGGGCAAACAATTCTGGAGATGATGTTTTTACTGTGTCTTGTAGAGTATTAACTAGGCCTAGTCCTGATTTCTCATTCAATTTCCTTGTCCCACCCACTGTGGAATCAAAGACAAAGCCTTTCACCCTCCCCATTCTGACTATCTCTGAAATGTCTAATTCCAGGTTTCCGGTGCCAATTGACTCTCTGCACACCAGCCCGACTGAGAACATTGTTGTCCAATGTCAAAATGGGCGCGTCACTCTTGACGGTGAGTTGATGGGTACCACCCAACTCTTACCGAGTCAGATATGCACTTTCAGGGGCACGCTCACCAGGTCAACAAGCAGGGCCAGTGACCAAGCCGACACAGCAACCCCTAGGCTATTCAACTATTATTGGCATATACAATTGGACAACCTAAATGGAACCCCCTACGACCCTGCAGAGGATATACCAGCCCCTCTGGGAACACCTGATTTCCGGGGCAAGGTCTTTGGCGTGGCCAGCCAGAGAAACCCCGACAGCACAACAAGAGCACATGAAGCAAAAGTGGACACAACATCTGGTCGCTTCATCCCGAAATTGGGCTCTCTAGAAATCTCCACTGAGTCCGATGATTTTGACCAAAACCAACCAACAAGGTTCACCCCAGTTGGCATTGGGGTTGACAATGAGGCAGGTTTTCAGCAATGGTCCTTACCTGACTATTCTGGTCAGTTTACTCACAACATGAACTTAGCCCCAGCTGTCGCCCCCAATTTTCCTGGTGAGCAACTTCTTTTCTTCCGCTCACAGCTGCCATCTTCTGGTGGGAGGTCTAACGGGATTCTAGACTGCCTGGTCCCCCAGGAATGGGTTCAACACTTCTACCAGGAATCTGCCCCTGCCCAAACACAAGTGGCCCTGGTTAGGTATGTCAACCCTGACACTGGTAGAGTGTTGTTTGAGGCCAAGTTACATAAATTAGGTTTCATGACTATATCTAAGAATGGTGATTCCCCAATAACTGTTCCCCCAAATGGGTACTTTAGATTTGAATCTTGGGTGAACCCCTTTTATACACTTGCCCCCATGGGAACTGGAAATGGGCGTAGAAGGATTCAATAA

>DQ379713|I.1/II.3|1983|AU|Goulburn Valley G5175 A

ATGAAGATGGCGTCGAATGACGCTGCTCCATCTAACGATGGTGCCGCCGGCCTCGTCCCAGAGATCAACAATGAGGCAATGGCGCTAGAGCCAGTGGCAGGTGCAGCAATAGCAGCACCTCTCACTGGCCAGCAAAATATAATTGATCCCTGGATTATGAATAATTTTGTGCAAGCACCTGGTGGTGAGTTTACAGTGTCGCCTAGGAACTCCCCTGGTGAAGTACTTCTCAATTTAGAATTAGGTCCAGAAATAAACCCTTATTTGGCCCACCTTGCTAGGATGTACAATGGTTATGCAGGTGGGTTTGAGGTGCAGGTAGTCCTGGCTGGAAATGCGTTTACAGCAGGAAAGGTGATCTTTGCAGCTATACCCCCTAATTTTCCAATTGATAATCTGAGCGCAGCACAGATTACAATGTGCCCGCATGTGATTGTGGATGTCAGACAGTTGGAACCAATCAACCTTCCGATGCCTGATGTCCGTAATAACTTCTTTCATTATAATCAAGGGTCTGATTCTAGGTTGCGCTTAATTGCAATGCTGTATACACCTCTTAGGGCAAATAATTCAGGGGATGATGTTTTCACTGTGTCTTGTAGGGTGTTAACTAGGCCTAGCCCTGATTTCTCATTTAATTTTCTCGTCCCGCCTACTGTAGAATCAAAGACAAAGCCCTTCACCCTCCCCATCTTGACTATTTCTGAAATGTCTAATTCTAGGTTTCCAGTACCAATTGACTCTCTGCACACCAGCCCGACTGAGAACATTGTTGTCCAGTGCCAAAATGGGCGAGTCACCCTTGACGGTGAGTTGATGGGCACCACTCAACTCTTGCCGAGTCAAATATGTGCCTTCAGGGGCACGCTCACCAGATCAACAAGCAGGGCCAGTGACCAAGCTGACACGGCAACCCCTAGGTTGTTCAACTACTATTGGCACATACAATTGGATAATCTAAATGGAACCCCATATGACCCTGCAGAGGACATACCAGCCCCCTTGGGAACACCAGACTTCCGGGGCAAGGTCTTCGGCGTAGCTAGCCAGAGAAACCCTGATAGTACAACAAGGGCACATGAAGCAAAAGTGGACACAACATCTGGTCGCTTCGCCCCGAAATTGGGTTCCCTAGAAATATCCACTGAATCCAGTGATTTTGACTCAAACCAACCAACAAGGTTCACCCCAGTTGGCATTGGGGTTGACAATGAGGCAGATTTTCAACAATGGTCCCTACCTGACTACTCCGGTCAGTTCACTCACAACATGAACTTAGCCCCAGCTGTCGCCCCCAATTTCCCTGGTGAGCAGCTTCTCTTCTTCCGTTCACAGCTGCCATCTTCTGGTGGGCGGTCTAATGGGATTCTGGACTGCCTGGTCCCCCAGGAATGGGTTCAACATTTCTACCAGGAATCAGCCCCTGCCCAAACACAGGTAGCCCTGGTTAGGTATGTTAACCCTGACACTGGCAGAGTGCTATTTGAGGCCAAGTTACACAAACTGGGCTTCATGACTATAGCTAAGAATGGTGATTCTCCAATAACTGTCCCTCCAAATGGGTACTTCAGATTTGAATCTTGGGTGAACCCCTTCTACACACTTGCCCCCATGGGAACTGGAAATGGGCGTAGAAGGATTCAATAA

>DQ419909|-/II.3|2004|CN|CR2987

ATGAAGATGGCGTCGAATGACGCCACTCCATCTAATGATGGTGCCGCCGGCCTCGTCCCAGAGATCAACAATGAGGCAATGGCGCTAGATCCAGTGGCGGGTGCAGCGATAGCAGCACCCCTCACTGGTCAGCAAAATATAATTGACCCCTGGATTATGAATAATTTTGTGCAAGCACCTGGTGGTGAGTTTACAGTATCCCCTAGGAATTCCCCTGGTGAAGTGCTTCTCAATTTGGAATTGGGCCCAGAAATAAATCCCTATTTGGCCCATCTTGCTAGAATGTATAATGGTTATGCAGGTGGGTTTGAAGTGCAGGTAGTCCTAGCTGGAAATGCGTTTACAGCAGGAAAGATAATCTTTGCAGCTATACCCCCTAATTTCCCAATTGATAATCTAAGCGCAGCACAGATCACAATGTGCCCACATGTGATTGTGGATGTCAGACAGTTGGAACCGGTCAACCTTCCGATGCCTGACGTTCGCAATAACTTCTTCCATTACAACCAAGGGTCTGATTCGAAATTGCGCTTAGTTGCAATGCTGTATACACCTCTTAGGGCAAATAATTCTGGGGATGATGTTTTTACTGTGTCTTGTAGAGTGCTGACCAGGCCTAGCCCTGACTTTTCATTTAATTTCCTTGTGCCACCTACTGTGGAGTCAAAGACAAAACCCTTCACCCTACCTATTCTGACTATCTCTGAAATGTCCAATTCTAGGTTTCCAGTGCCGATTGATTCTCTGCACACCAGCCCAACTGAGAATATTAGTGTCCAGTGCCAAAATGGACGCGTCACTCTTGATGGTGAGTTGATGGGCACCACCCAACTCTTACCTAGTCAAATCTGTGCTTTTAGGGGCGTGCTCACCAGATCAACAAGCAGGGCCAGTGACCAGGCCGACACAGCAACCCCTAGGTTGTTTAATTATTATTGGCACATACAATTGGATAATCTAAATGGGACTCCTTATGACCCTGCAGAAGACATACCAGGCCCCCTAGGGACACCAGATTTCCGGGGCAAAGTCTTTGGCGTGGCCAGCCAGAGAAATCCCGACAGCACAACTAGAGCACATGAAGCAAAGGTGGACACAACAGCTGGTCGTTTTACCCCAAAACTAGGCTCATTAGAGATATCCACTGAATCTGGTGACTTTGATCAAAACCAACCAACAAGATTCACCCCAGTTGGTATTGGGGTTGACCACGAGGAAAATTTCCAACAATGGTCTTTACCCGACTATTCTGGTCAGTTCACCCACAACATGAATTTAGCCCCAGCTGTTGCTCCCAACTTCCCTGGTGAGCAGCTTCTTTTCTTCCGCTCACAGTTACCATCTTCTGGTGGGCGATCCAACGGGATTCTAGACTGCCTGGTCCCCCAAGAATGGGTTCAGCACTTCTACCAAGAATCGGCCCCCGCCCAAACTCAAGTGGCCCTGGTTAGGTATGTCAACCCTGACACTGGTAGAGTGTTGTTTGAGGCCAAGCTGCACAAATTAGGTTTCATGACTATAGCTAAGAATGGTGACTCTCCAATAACTGTCCCCCCAAATGGATACTTTAGGTTTGAATCTTGGGTGAACCCATTTTATACACTTGCCCCCATGGGAACTGGGAATGGGCGTAGAAGGATTCAATAA

>EF547399|-/II.3|2001|JP|Maizuru/010524

ATGAAGATGGCGTCGAATGACGCTGCTCCATCTAATGATGGTGCCGCCGGCCTCGTCCCAGAGATCAACAATGAGGCAATGGCGCTAGACCCAGTGGCGGGTGCAGCGATAGCAGCACCCCTCACTGGTCAGCAGAATATAATTGATCCCTGGATTATGAATAATTTTGTGCAAGCACCTGGTGGTGAGTTTACAGTGTCCCCTAGGAATTCCCCTGGTGAAGTGCTTCTTAATTTGGAATTGGGCCCAGAAATAAACCCCTATTTAGCCCATCTTGCTAGAATGTATAATGGTTATGCAGGTGGATTTGAAGTGCAGGTAGTCCTGGCTGGAAATGCGTTTACAGCAGGAAAGATAATCTTTGCAGCTATACCCCCTAATTTTCCAATTGACAACCTGAGCGCAGCACAGATCACAATGTGCCCGCATGTGATTGTGGATGTCAGACAGTTGGAACCGGTCAACCTTCCGATGCCTGACGTTCGCAACAATTTCTTTCATTACAATCAAGGGTCTGATTCGAGATTGCGCTTAATTGCAATGCTGTATACACCTCTTAGGGCAAATAATTCTGGGGATGACGTTTTTACTGTGTCATGTAGAGTGTTGACTAGGCCTAGCCCTGACTTTTCATTTAATTTCCTTGTGCCACCTACTGTGGAGTCAAAGACAAAGCCCTTCACCCTTCCTATTCTGACTATTTCTGAAATGTCCAATTCTAGGTTTCCAGTGCCGATTGATTCTCTGCACACCAGCCCAACTGAGAATATTGTTGTCCAGTGCCAAAACGGGCGCGTCACTCTTGATGGTGAGTTAATGGGCACCACCCAACTCTTACCGAGTCAAATCTGTGCTTTCAGGGGCGTGCTCACCCGATCAACAAGCAGGGCCAGTGATCAGGCCGACACAGCAACCCCTAGGTTGTTTAATTATTATTGGCACATACAATTGGATAATCTGAATGGAACACCTTATGATCCTGCAGAAGACATACCAGGCCCCCTAGGAACACCAGATTTCCGGGGCAAAGTCTTTGGCGTGGCCAGCCAAAGAAACCCCGATAGCACAACTAGAGCACATGAAGCAAAGGTGGACACAACAGCTGGTCGTTTCACCCCAAAATTAGGCTCATTAGAAATATCCACTGAATCTGATGACTTTGACCAAAACCAACCAACAAGATTCACTCCAGTTGGCATTGGGGTTGACCATGAGGCAGACTTTCAACAATGGTCTTTACCCGACTATTCTGGCCAGTTCACCCACAACATGAACTTGGCCCCAGCTGTTGCTCCCAACTTCCCTGGTGAGCAGCTCCTCTTCTTCCGCTCACAGTTACCATCTTCTGGTGGGCGATCCAACGGGATTCTAGACTGCCTGGTTCCCCAAGAATGGGTTCAGCACTTCTACCAAGAATCGGCCCCCGCCCAAACACAAGTAGCCCTGGTTAGGTATGTCAACCCTGACACTGGTAGAGTATTATTTGAGGCCAAGCTGCATAAATTAGGTTTCATGACTATAGCTAAGAATGGTGATTCTCCAATAACTGTTCCTCCAAATGGATACTTTAGGTTCGAATCTTGGGTGAACCCCTTTTATACACTCGCCCCCATGGGAACTGGGAATGGGCGTAGAAGGATTCAATAA

>EF670649|II.12/II.3|2006|CN|Hebei/48580

ATGAAGATGGCGTCGAATGACGCCACTCCATCTAATGATGGTGCCGCCGGCCTCGTCCCAGAGATCAACAATGAGGCAATGGCGCTAGATCCAGTGGCGGGTGCAGCGATAGCAGCACCCCTCACTGGCCAGCAAAATATAATTGATCCCTGGATTATGAATAATTTTGTGCAAGCACCTGGTGGTGAGTTTACAGTGTCCCCTAGGAATTCCCCTGGTGAAGTGCTTCTCAATTTGGAATTGGGCCCAGAAATAAATCCCTATTTGGCCCATCTTGCTAGAATGTATAATGGTTATGCAGGTGGGTTTGAAGTGCAGGTAGTCCTAGCTGGAAATGCGTTTACAGCAGGAAAGATAATCTTTGCAGCTATACCCCCTAATTTCCCAATTGACAATCTAAGCGCAGCACAGATCACAATGTGCCCACATGTGATTGTGGATGTCAGACAGTTGGAACCGGTCAACCTTCCGATGCCTGACGTTCGCAATAACTTCTTCCATTACAACCAAGGGTCTGATTCGAGATTGCGCTTAATTGCAATGCTGTATACACCTCTTAGGGCAAATAATTCTGGGGATGATGTTTTTACTGTGTCTTGTAGAGTGCTGACTAGGCCTAGCCCTGACTTTTCATTTAATTTCCTTGTGCCACCTACTGTGGAGTCAAAGACAAAACCCTTCACCCTCCCTATTCTGACTATCTCTGAAATGTCTAATTCTAGGTTTCCAGTGCCGATTGATTCTCTGCACACCAGCCCAACTGAGAATATTGTTGTCCAGTGCCAAAATGGACGCGTCACTCTTGATGGTGAGTTGATGGGCACCACCCAACTCTTACCTAGTCAAATCTGTGCTTTTAGGGGCGTGCTCACCAGATCAACAAGCAGGCCCAGTGATCAGGCCGACACAGCAACCCCTAGATTGTTTAATTATTATTGGCACATACAATTGGATAATCTAAATGGGACTCCTTATGATCCTGCAGAAGACATACCAGGCCCCCTGGGGACACCAGATTTCCGGGGCAAAGTCTTTGGCGTGGCCAGCCAGAGAAATCCCGACAGTACAACTAGAGCACATGAAGCAAAGGTGGACACAACAGCTGGTCGTTTTACCCCAAAACTAGGCTCATTAGAGATATCCACTGAATCTGGTGACTTTGATCAAAACCAACCAACAAGATTCACCCCAGTTGGCATTGGGGTTGACCACGAGGCAGATTTCCAACAATGGTCTTTACCCGACTATTCTGGTCAGTTCACCCACAACATGAACTTAGCCCCAGCTGTTGCTCCCAACTTCCCTGGTGAGCAGCTCCTTTTCTTCCGCTCGCAGTTACCATCTTCTGGTGGGCGATCCAACGGGATTCTAGACTGCCTGGTCCCCCAAGAATGGGTCCAGCACTTCTACCAAGAATCGGCCCCCGCCCAAACCCAAGTGGCCCTGGTTAGATATGTCAACCCTGACACTGGTAGAGTGTTGTTTGAGGCCAAGCTGCACAAATTAGGTTTCATGACTATAGCTAAGAATGGTGACTCTCCAATAACTGTCCCCCCAAATGGATACTTTAGGTTTGAATCTTGGGTGAACCCATTTTATACACTTGCCCCCATGGGAACTGGGAATGGGCGTAGAAGGATTCAATAA

>EU072241|II.21/II.3|2004|CN|CHN39186/CC04

ATGAAGATGGCGTCGAATGACGCCACTCCATCTAATGATGGTGCCGCCGGCCTCGTCCCAGAGATCAACAATGAGGCAATGGCGCTAGACCCAGTGGCGGGTGCAGCGATAGCAGCACCCCTCACTGGTCAGCAGAATATAATTGATCCCTGGATTATGGATAATTTTGTGCAAGCACCTGGTGGTGAGTTTACAGTATCCCCTAGGAATTCCCCTGGTGAAGTGCTTCTTAATTTGGAATTGGGCCCAGAAATAAATCCCTATTTGGCTCATCTTGCTAGAATGTATAATGGTTATGCAGGTGGATTTGAAGTGCAGGTAGTCCTAGCTGGAAATGCGTTTACAGCGGGAAAGATAATCTTTGCAGCTATACCCCCTAATTTCCCAATTGATAATCTAAGCGCAGCACAGATCACAATGTGCCCACACGTGATCGTGGATGTCAGACAGTTGGAACCGGTCAACCTCCCGATGCCTGACGTTCGCAACAACTTCTTTCATTACAATCAAGGGTCTGATTCGAGATTGCGCTTAATTGCAATGCTGTACACACCTCTTAGGGCAAACAATTCTGGGGATGATGTTTTTACTGTGTCTTGTAGAGTGCTGACTAGACCTAGCCCTGACTTTTCATTTAATTTCCTTGTGCCACCTACTGTGGAGTCAAAGACAAAACCCTTCACCCTCCCTATTCTGACTATCTCTGAAATGTCCAATTCTAGGTTTCCAGTGCCGATTGATTCTCTGCACACCAGCCCAACTGATAATATTGTTGTCCAGTGCCAAAATGGGCGCGTCACTCTTGATGGTGAGTTGATGGGCACCACCCAACTCTTACCTAGTCAAATCTGTGCTTTCAGGGGCGTGCTCACCAGATCGACAAGCAGGGCCAGTGATCAGGCCGACACAGCAACCCCTAGGTTGTTCAATTATTATTGGCACATACAATTGGATAATCTAAATGGGACTCCTTATGATCCTGCAGAAGACATACCAGGCCCCCTAGGGACACCAGATTTCCGGGGCAAAGTCTTTGGCGTGGCCAGCCAGAGAAACCCCGACAGCACAACTAGAGCACATGAAGCAAAGGTGGACACAACAGCTGGTCGTTTTACCCCAAAACTAGGCTCATTAGAGATATCCACTGAATCTAATGACTTTGACCAAAACCAACCAACAAGATTCACCCCAGTTGGTATTGGGGTTGACCACGAGGCAGACTTTCAACAATGGTCTTTACCCGACTACTCTGGTCAGTTCACCCACAACATGAACTTAGTCCCAGCTGTTGCTCCCAACTTCCCTGGTGAGCAGCTCCTTTTCTTCCGCTCACAGTTACCATCCTCTGGTGGGCGATCCAACGGGATTCTAGACTGCCTGGTCCCCCAAGAATGGGTTCAGCACTTCTACCAAGAATCGGCCCCCGCCCAAACTCAAGTGGCCCTGGTTAGGTATGTCAACCCTGACACTGGTAGAGTATTATTTGAGGCCAAGCTGCACAAATTAGGTTTCATGACTATAGCTAAGAATGGTGACTCTCCAATAACTGTCCCTCCAAATGGATACTTTAGGTTTGAATCTTGGGTGAACCCCTTTTATACACTTGCCCCCATGGGAACTGGGAATGGGCGTAGAAGAAATCAATAA

>EU072243|II.21/II.3|2004|CN|CHN39246/CC04

ATGAAGATGGCGTCGAATGACGCCACTCCATCTAATGATGGTGCCGCCGGCCTCGTCCCAGAGATCAACAATGAGGCAATGGCGCTAGACCCAGTGGCGGGTGCAGCGATAGCAGCACCCCTCACTGGTCAGCAAAATATAATTGATCCCTGGATTATGAATAATTTTGTGCAAGCACCTGGTGGTGAGTTTACAGTTTCCCCTAGGAATTCCCCTGGTGAAGTGCTTCTTAATTTGGAATTGGGCCCAGAAATAAATCCCTATTTGTCCCATCTTGCTAGAATGTATAATGGTTATGCAGGTGGATTTGAAGTGCAGGTTGTCCTAGCTGGAAATGCGTTTACAGCAGGAAAGATAATCTTTGCAGCTATACCCCCTAATTTCCCAATTGACAATTTAAGCGCAGCACAGATCACAATGTGCCCACATGTGATTGTGGATGTCAGACAGTTGGAACCGGTCAACCTCCCGATGCCTGACGTTCGCAACAACTTCTTTCATTACAATCAAGGGTCTGATTCGAGGTTGCGCTTAATTGCAATGCTGTATACACCTCTTAGGGCAAATAATTCTGGGGATGATGTTTTTACTGTGTCTTGTAGAGTGCTGACTAGGCCTAGCCCTGACTTTTCATTTAATTTCCTTGTGCCACCTACTGTGGAATCAAAGACAAAACCCTTCACCCTCCCTATTCTGACTATCTCTGAGATGTCCAATTCTAGGTTTCCAGTGCCGATTGATTCTCTGCACACCAGCCCAACTGAGAATATTGTTGTCCAGTGTCAAAATGGGCGCGTCACTCTTGATGGTGAGTTGATGGGCACCACCCAACTCTTACCTAGTCAAATCTGTGCTTTTAGGGGCGTGCTCACCAGATCAACAAGCAGGGCCAGTGACCAGGCCGACACAGCAACCCCTAGGTTGTTTAATTACTATTGGCACATACAATTGGATAATCTAAATGGGACTCCTTATGACCCTGCAGAAGACATACCAGGCCCCCTAGGGACACCAGATTTCCGGGGCAAAGTCTTTGGCGTGGCCAGCCAAAGAAACCCCGACAGCACAACTAGAGCACATGAAGCAAAGGTGGACACAACAGCTGGTCGTTTCACCCCAAAACTAGGCTCATTGGAGATATCCACTGAATCTGATGACTTTGACCAAAACCAACCGACAAGATTCACCCCAGTTGGCATTGGGGTTGACCATGAGGCAGACTTTCAACAATGGTCTTTACCCGACTACTCTGGACAGTTCACCCACAACATGAACTTAGCCCCAGCTGTTGCTCCCAACTTCCCTGGTGAGCAGCTCCTTTTCTTCCGCTCACAGTTACCATCCTCTGGTGGGCGATCCAACGGGATTCTAGACTGCCTGGTCCCCCAAGAATGGGTTCAGCACTTCTACCAAGAATCGGCCCCCGCCCAAACCCAAGTGGCCCTGGTTAGGTATGTCAACCCTGACACTGGTAGAGTATTATTTGAGGCCAAGCTGCACAAATTAGGTTTCATGACTATAGCTAAGAATGGTGACTCTCCAATAACTGTCCCTCCAAATGGATACTTTAGGTTTGAGTCTTGGGTGAACCCCTTTTACACACTTGCCCCCATGGGAACTGGGAATGGGCGTAGAAGAAATCAATAA

>EU085479|II.21/II.3|2004|SE|P1.Confectionary2004

ATGAAGATGGCGTCGAGTGACGCCACTCCATCTAATGATGGTGCCGCCGGCCTCGTCCCAGAGATCAACAATGAGGCAATGGCGCTAGATCCAGTGGCGGGTGCAGCGATAGCAGCACCCCTCACTGGTCAGCAAAATATAATTGATCCCTGGATTATGAATAATTTTGTGCAAGCACCTGGTGGTGAGTTTACTGTGTCCCCTAGGAATTCCCCTGGTGAAGTGCTTCTTAATTTGGAATTGGGCCCAGAAATAAATCCCTATTTGGCCCATCTTGCTAGAATGTATAATGGTTATGCAGGTGGATTTGAAGTGCAGGTAGTCCTAGCTGGAAATGCGTTTACAGCAGGAAAGATAATCTTTGCAGCTATACCCCCTAATTTTCCAATTGATAATTTAAGCGCAGCACAGATCACAATGTGCCCACATGTGATCGTGGATGTCAGACAATTGGAACCGGTCAACCTTCCGATGCCTGACGTTCGCAATAACTTCTTTCACTACAATCAAGGGTCTGATTCGAGATTGCGCTTAATTGCAATGCTGTACACACCTCTCAGGGCAAATAATTCTGGGGATGATGTTTTTACTGTGTCTTGTAGAGTGCTGACCAGGCCTAGCCCTGACTTTTCATTTAATTTTCTTGTGCCACCTACTGTGGAGTCAAAGACAAAACCCTTCACCCTCCCTATTCTGACTATCTCTGAAATGTCTAATTCTAGGTTTCCAGTGCCGATTGATTCTCTGCACACCAGCCCAACTGAGAATATTGTTGTCCAGTGCCAAAATGGGCGCGTCACTCTTGATGGTGAGTTGATGGGCACCACCCAACTCTTACCTAGTCAAATCTGTGCTTTCAGGGGCGTGCTCACCAGATCAACAAGCAGGACCAGTGATCAGGCCGATACAGCAACCCCTAGATTGTTTAATTATTATTGGCATATACAGTTGGATAATCTAAATGGGACTCCTTATGATCCTGCAGAAGACATACCAGGCCCCCTAGGGACACCAGATTTCCGGGGCAAAGTCTTTGGCGTGGCCAGCCAGAGAAATCCCGACAGCACAACTAGAGCACATGAGGCAAAGGTGGACACAACAGCTGGTCGTTTCACCCCAAAACTAGGCTCATTAGAGATATCCACTGAATCTAGTGACTTTGATCAAAATCAACCAACAAGATTCACCCCAGTTGGCATTGGGGTTGACCATGAGACAGACTTTCAACAATGGTCTTTACCCGACTACTCTGGTCAGTTCACCCACAACATGAACTTAGCCCCAGCTGTTGCTCCCAACTTTCCTGGTGAGCAGCTCCTTTTCTTCCGCTCACAGCTACCATCCTCTGGTGGGCGATCCAACGGGATTCTAGATTGCCTGGTCCCCCAAGAATGGGTTCAGCACTTCTACCAAGAATCGGCCCCCGCCCAAACGCAAGTGGCCCTGGTTAGGTATGTCAACCCTGACACTGGTAGAGTATTATTTGAAGCCAAGCTGCACAAATTAGGTTTTATGACCATAGCCAAAAATGGTGACTCTCCAATAACTGTCCCTCCAAATGGGTACTTTAGGTTTGAATCTTGGGTGAACCCCTTTTATACACTTGCCCCCATGGGATCTGGAAAGG

>EU187437|II.21/II.3|2003|JP|5017.34

ATGAAGATGGCGTCGAATGACGCCACTCCATCTAATGATGGTGCCGCCGGCCTCGTCCCAGAGATCAACAATGAGGCAATGGCGCTAGATCCAGTGGCGGGTGCAGCGATAGCAGCACCCCTCACTGGTCAGCAAAATATAATTGATCCCTGGATTATGAATAACTTTGTGCAAGCACCTGGTGGTGAGTTTACAGTATCCCCTAGGAATTCCCCTGGTGAAGTGCTTCTTAATTTGGAATTGGGCCCAGAAATAAATCCCTATTTGGCCCATCTTGCTAGAATGTATAATGGTTATGCAGGTGGGTTTGAAGTGCAGGTAGTCCTAGCTGGAAATGCGTTTACAGCAGGAAAGATAATCTTTGCAGCTATACCCCCTTATTTTCCAATTGATAATTTAAGCGCAGCACAGATCACAATGTGCCCACATGTGATTGTGGATGTCAGACAGTTGGAACCGGTCAACCTCCCGATGCCTGACGTTCGCAATAACTTCTTTCATTATAATCAAGGGTCTGATTCGAGATTGCGCTTAGTTGCAATGCTGTATACACCTCTTAGGGCAAATAATTCTGGGGATGATGTTTTTACTGTGTCTTTGAGAGTGCTGACTAGGCCTAGCCCTGACTTTTCATTTAATTTCCTTGTGCCACCTACTGTGGAGTCAAAGACAAAACCCTTTACCCTCCCTATTCTGACTATCTCTGAAATGTCCAATTCTAGGTTTCCAGTGCCGATTGATTCTCTGCACACCAGCCCAACTGAGAATATTGTTGTCCAGTGCCAAAATGGGCGCGTCACTCTTGATGGTGAGTTGATGGGCACCACCCAACTCTTACCTAGTCAGATCTGTGCTTTCAGGGGCGTGCTCACCAGATCAACAAGCAGGGCCAGTGACCAGGCCGACACAGCAACCCCTAGGTTGTTTAATTATTATTGGCACATACAATTGGATAATCTAAACGGGACTCCTTATGATCCTGCAGAAGACATACCAGGCCCCCTAGGGACACCAGATTTCCGGGGCAAAGTCTTTGGCGTGGCCAGCCAGAGAAATCCCGACAGCACAACTAGAGCACATGAAGCAAAGGTGGACACAACAGCTGGTCGTTTTACCCCAAAACTAGGCTCATTAGAGATATCCACTGAATCTGATGACTTTGATCAAAACCAACCAACAAGATTCACCCCAGTTGGCATTGGGGTTGACCACGAGGCAGATTTCCAACAATGGTCTTTACCCGACTATTCTGGTCAGTTCACCCACAACATGAACTTAGCCCCAGCTGTTGCTCCCAACTTCCCTGGTGAGCAGCTCCTTTTCTTCCGCTCACAGTTACCATCCTCTGGTGGGCGATCCAACGGGATTCTAGACTGCCTGGTCCCCCAAGAATGGGTTCAGCACTTCTACCAAGAATCGGCCCCCGCCCAAACTCAAGTGGCCCTGGTTAGGTATGTCAACCCTGACACTGGTAGAGTATTGTTTGAGGCCAAGCTGCACAAATTAGGTTTCATGACTATAGCTAAGAATGGTGACTCTCCAATAACTGTCCCCCCAAATGGATATTTTAGGTTTGAATCTTGGGTGAACCCATTTTATACACTTGCCCCCATGGGAACTGGGAATGGGCGTAGAAGGATTCAATAA

>EU850823|-/II.3|2005|CN|Beijing/06

ATGAAGATGGCGTCGAATGACGCCACTCCATCTAATGATGGTGCCGCCGGCCTCGTCCCAGAGATCAACAATGAGGCAATGGCGCTAGATCCAGTGGCGGGTGCAGCGATAGCAGCACCCCTCACTGGTCAGCAAAATATAATTGATCCCTGGATTATGAATAATTTTGTGCAAGCACCTGGTGGTGAGTTTACAGTATCCCCTAGGAATTCCCCTGGTGAAGTGCTTCTCAATTTGGAATTGGGCCCAGAAATAAATCCCTATTTGGCTCATCTTGCTAGAATGTATAATGGTTATGCAGGTGGATTTGAAGTGCAGGTAGTCCTAGCTGGAAATGCGTTTACAGCAGGAAAGATAATCTTTGCAGCTATACCCCCTAATTTCCCAATTGAAAATCTAAGCGCAGCACAGATCACAATGTGCCCACATGTGATTGTGGATGTCAGACAGTTGGAACCGGTCAACCTTCCGATGCCTGACGTTCGCAATAACTTCTTCCATTACAACCAAGGGTCTGATTCGAAATTGCGCTTAGTTGCAATGTTGTATACACCTCTTAGGGCAAATAATTCTGGGGATGATGTTTTTACTGTGTCTTGTAGAGTGCTGACTAGGCCTAGCCCTGACTTTTCATTTAATTTCCTTGTGCCACCTACTGTGGAGTCAAAGACAAAACCCTTCACCCTCCCTATTCTGACTATCTCTGAAATGTCCAATTCGAGGTTTCCAGTGCCGATTGATTCTCTGCACACCAGCCCAACTGAGAATATTGTTGTCCAGTGCCAAAATGGACGCGTCACTCTTGATGGTGAGTTGATGGGCACCACCCAACTCTTACCTAGTCAAATCTGTGCTTTTAGGGGCGTGCTCACCAGATCAACAAGCAGGGCCAGTGACCAGGCCGACACGGCAACCCCTAGGTTGTTTAATTATTACTGGCACATACAATTGGATAATCTAAACGGGACTCCTTATGATCCTGCAGAAGACATACCAGGCCCCCTAGGGACACCAGATTTCCGGGGCAAAGTCTTTGGCGTGGCCAGCCAGAGAAATCCCGACAGCACAACTAGAGCACATGAAGCAAAGGTGGACACAACAGCTGGTCGTTTCACCCCAAAACTAGGCTCATTAGAGATATCCACTGAATCTGGTGACTTTGATCAAAACCAACCAACAAGATTCACCCCAGTTGGCATTGGGGTTGACCACGAGGCAGATTTCCAACAATGGTCTTTACCCGACTATTCTGGTCAGTTCACCCACAACATGAACTTAGCCCCAGCTGTTGCCCCCAACTTCCCTGGTGAGCAGCTCCTTTTCTTCCGCTCACAGTTACCATCTTCTGGTGGGCGATCCAACGGGATTCTAGACTGCCGGGTCCCCCAAGAATGGGTTCAGCACTTCTACCAAGAATCGGCCCCCGCCCAAACTCAAGTGGCCCTGGTTAGGTATGTCAACCCTGACACTGGTAGAGTGTTGTTTGAGGCCAAGCTGCACAAATTAGGTTTCATGACTATAGCTAAGAATGGTGACTCTCCAATAACCGTCCCCCCAAATGGATACTTTAGGTTTGAATCTTGGGTGAACCCATTTTATACACTTGCCCCCATGGGAACTGGGAATGGGCGTAGAAGGATTCAATAA

>EU850824|-/II.3|2005|CN|Beijing/48

ATGAAGATGGCGTCGAATGACGCCACTCCATCTAATGATGGTGCCGCCGGCCTCGTCCCAGAGATCAACAATGAGGCAATGGCGCTAGATCCAGTGGCGGGTGCAGCGATAGCAGCACCCCTCACTGGTCAGCAAAATATAATTGATCCCTGGATTATGAATAATTTTGTGCAAGCACCTGGTGGTGAGTTTACAGTATCCCCTAGGAATTCCCCTGGTGAAGTGCTTCTCAATTTGGAATTGGGCCCAGAAATAAATCCCTATTTGGCCCATCTTGCTAGAATGTATAATGGTTATGCAGGTGGGTTTGAAGTGCAGGTAGTCCTAGCTGGAAATGCGTTTACAGCAGGAAAGATAATCTTTGCAGCTATACCCCCTAATTTCCCAATTGATAATCTAAGTGCAGCACAGATCACAATGTGCCCACATGTGATTGTGGATGTCAGACAGTTGGAACCGGTCAACCTTCCGATGCCTGACGTTCGCAATAACTTCTTCCATTACAACCAAGGGTCTGATTCGAGATTGCGCTTAGTTGCAATGCTGTATACACCTCTTAGGGCAAATAATTCTGGGGATGATGTTTTCACTGTGTCTTGTAGAGTGCTGACTAGGCCTAGCCCTGACTTTTCATTTAATTTCCTTGTGCCACCTACTGTGGAGTCAAAGACAAAACCCTTCACCCTCCCTATTCTGACTATCTCTGAAATGTCCAATTCTAGGTTTCCAGTGCCGATTGACTCTTTGCACACCAGCCCAACTGAGAATATTGTTGTCCAGTGCCAAAATGGACGCGTCACTCTTGATGGTGAGTTGATGGGCACCACCCAACTCTTACCTAGTCAAATCTGTGCTTTTAGGGGCGTGCTCACCAGATCAACAAGCAGGGCCAGTGACCAGGCCGACACAGCAACCCCTAGGTTGTTTAATTATTATTGGCACATACAATTGGATAATCTAAATGGGACTCCTTATGATCCTGCAGAAGACATACCAGGCCCCCTAGGGACACCAGATTTCCGGGGCAAAGTCTTTGGCGTGGCCAGCCAGAGAAATCCCGACAGCACAACTAGAGCACATGAAGCAAAGGTGGACACAACAGCTGGTCGTTTTACCCCAAAACTAGGCTCATTAGAGATATCCACTGAATCTGGTGACTTTGATCAAAACCAACCAACAAGATTCACCCCAGTTGGCATTGGGGTTGACCACGAGGCAGATTTCCAACAATGGTCTTTACCCGACTATTCTGGTCAGTTCACCCACAACATGAACTTAGCCCCAGCTGTTGCTCCCAACTTCCCTGGTGAGCAGCTCCTTTTCTTCCGCTCACAGTTACCATCTTCTGGTGGGCGATCCAACGGGATTCTAGACTGCCTGGTCCCCCAAGAATGGGTTCAGCACTTTTACCAAGAATCGGCCCCCGCCCAAACTCAAGTGGCCCTGGTTAGGTATGTCAACCCTGACACTGGTAGAGTGTTGTTTGAGGCCAAGCTGCACAAATTAGGTTTCATGACTATAGCTAAGAATGGTGACTCTCCAATAACTGTCCCCCCAAATGGATACTTTAGGTTTGAATCTTGGGTGAACCCATTTTATACACTTGCCCCCATGGGAACTGGGAATGGGCGTAGAAGGATTCAATAA

>EU850825|-/II.3|2005|CN|Beijing/148

ATGAAGATGGCGTCGAATGACGCCACTCCATCTAATGATGGTGCCGCCGGCCTCGTCCCAGAGATCAACAATGAGGCAATGGCGCTAGATCCAGTGGCGGGTGCAGCGATAGCAGCACCCCTCACTGGTCAGCAAAATATAATTGATCCCTGGATTATGAATAATTTTGTGCAAGCACCTGGTGGTGAGTTTACAGTATCCCCTAGGAATTCCCCTGGTGAAGTGCCTCTCAATTTGGAATTGGGCCCAGAAATAAATCCCTATTTGGCCCATCTTGCTAGAATGTATAATGGTTATGCAGGTGGGTTTGAAGTGCAGGTAGTCCTAGCTGGAAATGCGTTTACAGCAGGAAAGATAATCTTTGCAGCTATACCCCCTAACTTCCCAACTGATAATCTAAGTGCAGCACAGATCACAATGTGCCCACATGTGATTGTGGATGTCAGACAGTTGGAACCGGTCAACCTTCCGATGCCTGACGTTCGCAATAATTTCTTCCATTACAACCAAGGGTCTGATTCGAGATTGCGCTTAGTTGCAATGCTGTATACACCTCTTAGGGCAAATAATTCTGGGGATGATGTTTTCACTGTGTCTTGTAGAGTGCTGACTAGGCCTAGCCCTGACTTTTCATTTAATTTCCTTGTGCCACCTACTGTGGAGTCAAAGACAAAACCCTTCACCCTCCCTATTCTGACTATCTCTGAAATGTCCAATTCTAGGTTTCCAGTGCCGATTGATTCTCTGCACACCAGCCCAACTGAGAATATTGTTGTCCAGTGCCAAAATGGACGCGTCACTCTTGATGGTGAGTTGATGGGCACCACCCAACTCTTACCTAGTCAAATCTGTGCTTTTAGGGGTGTGCTCACCAGATCAACAAGCAGGGCCAGTGACCAGGCCGACACAGCAACCCCTAGGTTGTTTAATTATTATTGGCACATACAATTGGATAATCTAAATGGGACTCCTTATGATCCTGCAGAAGACATACCAGGCCCCCTAGGGACACCAGATTTCCGGGGCAAAGTCTTTGGCGTGGCCAGCCAGAGAAATCCCGACAGCACAACTAGAGCACATGAAGCAAAGGTGGACACAACAGCTGGTCGTTTTACCCCAAAACTAGGCTCATTAGAGATATCCACTGAATCTGATGACTTTGATCAAAACCAACCAACAAGATTCACCCCAGTTGGCATTGGGGTTGACCACGAGGCAGATTTCCAACAATGGTCTTTACCCGACTATTCTGGTCAGTTCACCCACAACATGAACTTAGCCCCAGCTGTTGCTCCCAACTTCCCTGGTGAGCAGCTCCTTTTCTTCCGCTCACAGTTACCATCTTCTGGTGGGCGATCCAACGGGATTCTAGACTGCCTGGTCCCCCAAGAATGGGTTCAGCACTTCTACCAAGAATCGGCCCCCGCCCAAACTCAAGTGGCCCTGGTTAGGTATGTCAACCCTGACACTGGTAGAGTGTTGTTTGAGGCCAAGCTGCACAAATTAGGTTTCATGACTATAGCTAAGAATGGTGATTCTCCAATAACTGTCCCCCCAAATGGATACTTTAGGTTTGAATCTTGGGTGAACCCATTTTATACACTTGCCCCCATGGGAACTGGGAATGGGCGTAGAAGGATTCAATAA

>EU850826|-/II.3|2005|CN|Beijing/169

ATGAAGATGGCGTCGAATGACGCCACTCCATCTAATGATGGTGCCGCCGGCCTCGTCCCAGAGATCAACAATGAGGCAATGGCGCTAGATCCAGTGGCGGGTGCAGCGATAGCAGCACCCCTCACTGGCCAGCAAAATATAATTGATCCCTGGATTATGAATAATTTTGTGCAAGCACCTGGTGGTGAGTTTACAGTATCCCCTAGGAATTCCCCTGGTGAAGTGCTTCTCAATTTGGAATTGGGCCCAGAAATAAATCCCTATTTGGCCCATCTTGCTAGAATGTATAATGGTTATGCAGGTGGGTTTGAAGTGCAGGTAGTCCTAGCTGGAAATGCGTTTACAGCAGGAAAGATAATCTTTGCAGCTATACCCCCTAATTTTCCAATTGATAATCTAAGCGCAGCACAGATCACAATGTGCCCACATGTGATTGTGGATGTCAGACAGTTGGAACCGGTCAACCTTCCGATGCCTGACGTTCGCAATAACTTCTTCCATTACAACCAGGGGTCTGATTCGAGATTGCGCTTAGTTGCAATGCTGTATACACCTCTTAGGGCAAATAATTCTGGGGATGATGTTTTCACTGTGTCTTGTAGAGTGCTGACTAGACCTAGTCCTGACTTTTCATTTAATTTCCTCGTTCCACCTACTGTGGAGTCAAAGACAAAACCCTTCACCCTCCCTATTCTGACTATCTCTGAAATGTCCAATTCTAGGTTTCCAGTGCCGATTGATTCTCTGCACACCAGCCCAACTGAGAATATTGTTGTCCAGTGCCAAAATGGACGCGTCACTCTTGATGGTGAGTTGATGGGCACCACCCAACTCTTACCTAGTCAAATTTGTGCTTTTAGGGGCGTGCTCACCAGATCAACAAGCAGGGCCAGTGACCAGGCCGACACAGCAACCCCTAGGTTGTTTAATTACTATTGGCACATACAATTGGATAATCTAAATGGGACTCCTTATGATCCTGCAGAAGACATACCAGGCCCCCTAGGGACACCAGATTTCCGGGGCAAAGTCTTTGGCGTGGCCAGCCAGAGAAATCCCGACAGCACAACTAGAGCACATGAAGCAAAGGTGGACACAACAGCTGGTCGTTTTACCCCAAAACTAGGCTCATTAGAGATATCCACTGAATCTGATGACTTTGACCAAAACCAACCAACAAGATTCACCCCAGTTGGCATTGGGGTTGACCATGAGGCAGATTTCCAACAATGGTCTTTACCCGACTATTCTGGCCAGTTCACCCACAACATGAACTTAGCCCCAGCTGTTGCTCCCAACTTCCCTGGTGAGCAGCTCCTTTTCTTCCGCTCACAGTTACCATCTTCTGGTGGGCGATCCAACGGGATTCTAGACTGCCTGGTCCCCCAAGAATGGGTTCAGCACTTCTACCAAGAATCGGCCCCCGCCCAAACTCAAGTGGCCCTGGTCAGGTATGTCAACCCTGACACTGGTAGAGTGTTGTTTGAGGCCAAGCTGCACAAATTAGGTTTCATGACTATAGCTAAGAATGGTGACTCTCCAATAACTGTCCCCCCAAATGGATACTTTAGGTTTGAATCTTGGGTGAACCCATTTTATACACTTGCCCCCATGGGAACTGGGAATGGGCGTAGAAGGATTCAATAA

>EU850827|-/II.3|2005|CN|Beijing/375

ATGAAGATGGCGTCGAATGACGCCACTCCATCTAATGATGGTGCCGCCGGCCTCGTCCCAGAGATCAACAATGAGGCAATGGCGCTAGATCCAGTGGCGGGTGCAGCGATAGCAGCACCCCTCACTGGTCAGCAAAATATAATTGATCCCTGGATTATGAATAATTTTGTGCAAGCACCTGGTGGTGAGTTTACAGTATCCCCTAGGAATTCCCCTGGTGAAGTGCTTCTCAATTTGGAATTGGGCCCAGAAATAAATCCCTATTTGGCCCATCTTGCTAGAATGTATAATGGTTATGCAGGTGGGTTTGAAGTGCAGGTAGTCCTAGCTGGAAATGCGTTTACAGCAGGAAAGATAATCTTTGCAGCTATACCCCCTAACTTCCCAATTGATAATCTAAGTGCAGCACAGATCACAATGTGCCCACATGTGATTGTGGATGTCAGACAGTTGGAACCGGTCAACCTTCCGATGCCTGACGTTCGCAATAATTTCTTCCATTACAACCAAGGGTCTGATTCGAGATTGCGCTTGGTTGCAATGCTGTATACACCTCTTAGGGCAAATAATTCTGGGGATGATGTTTTCACTGTATCTTGTAGAGTGCTGACTAGGCCTAGCCCTGACTTTTCATTTAATTTCCTTGTGCCACCTACTGTGGAGTCAAAGACAAAACCCTTCACCCTCCCTATTCTGACTATCTCTGAAATGTCCAATTCTAGGTTTCCAGTGCCGATTGATTCTCTGTACACCAGCCCAACTGAGAATATTGTTGTCCAGTGCCAAAATGGACGCGTCACTCTTGATGGTGAGTTGGTGGGCACCACCCAACTCTTACCTAGTCAAATCTGTGCTTTTAGGGGCGTGCTCACCAGATCAACAAGCAGGGCCAGTGACCAGGCCGACACAGCAACCCCTAGGTTGTTTAATTATTATTGGCACATACAATTGGATAATCTAAATGGGACTCCTTATGATCCTGCAGAAGACATACCAGGCCCCCTAGGGACACCAGATTTCCGGGGCAAAGTCTTTGGCGTGGCCAGCCAGAGAAATCCCGACAGCACAACTAGAGCACATGAAGCAAAGGTGGACACAACAGCTGGTCGTTTTACCCCAAAACTAGGCTCATTAGAGATATCCACTGAATCTGGTGACTTTGATCAAAACCAACCAACAAGATTCACCCCAGTTGGCATTGGGGTTGACCACGAGGCAGATTTCCAACAATGGTCTTTACCCGACTATTCTGGTCAGTTCACCCACAACATGAACTTAGCCCCAGCTGTTGCTCCCAACTTCCCTGGTGAGCAGCTCCTTTTCTTCCGCTCACAGTTACCATCTTCTGGTGGGCGATCCAACGGGATTCTGGACTGCCTGGTCCCCCAAGAATGGGTTCAGCACTTCTACCAAGAATCGGCCCCCGCCCAAACTCAAGTGGCCCTGGTTAGGTATGTCAACCCTGACACTGGTAGAGTGTTGTTTGAGGCCAAGCTGCACAAATTAGGTTTCATGACTATAGCTAAGAATGGTGACTCTCCAATAACTGTCCCCCCAAATGGATACTTTAGGTTTGAATCTTGGGTGAACCCATTTTATACACTTGCCCCCATGGGAACTGGGAATGGGCGTAGAAGGATTCAATAA

>EU921389|II.21/II.3|2007|IN|PC52

ATGAAGATGGCGTCGAATGACGCCGCTCCATCTAATGATGGTGCCGCCGGCCTCGTCCCAGAGATCAACAATGAGGCAATGGCGCTAGAGCCAGTGGCGGGTGCAGCGATAGCAGCACCCCTCACTGGTCAGCAAAATATAATTGATCCCTGGATTATGAATAATTTTGTGCAAGCACCTGGTGGTGAGTTTACAGTATCCCCTAGAAATTCCCCTGGTGAAGTTCTTCTTAATTTGGAATTGGGCCCAGAAATAAATCCCTATTTGGCCCATCTTGCTAGAATGTATAATGGTTATGCAGGTGGATTTGAAGTGCAGGTGGTCCTAGCTGGAAATGCGTTTACAGCAGGAAAGATAATCTTTGCAGCTATTCCCCCTAATTTTCCAATTGATAATCTAAGTGCAGCACAGATCACAATGTGTCCACATGTGATTGTGGATGTCAGACAGCTGGAACCAGTCAACCTCCCAATGCCTGACGTTCGTAACAACTTCTTTCATTACAATCAAGGGTCTGATTCGAGATTGCGCCTAATTGCAATGCTGTATACACCTCTTAGGGCAAATAATTCTGGGGATGATGTTTTTACTGTGTCTTGCAGAGTGCTAACTAGACCTAGTCCTGACTTCTCATTTAATTTCCTTGTGCCACCTACTGTGGAGTCAAAGACAAAACCCTTTTCCCTCCCTATTCTGACTATCTCTGAAATGTCTAATTCTAGGTTCCCAGTACCAATTGATTCTCTGCACACCAGCCCTACTGAGAACATTGTTGTCCAGTGTCAGAATGGACGCGTCACCCTTGATGGTGAGTTGATGGGCACCACCCAACTCTTACCTAGCCAAATCTGTGCTTTCAGGGGCGTGCTCACCAGATCAACAAGCAGGGCCAGTGACCAGGCCGATACAGCAACCCCTAGATTGTTTAATTATTATTGGCATATACAGTTGGATAATCTAAATGGAACTCCTTATGACCCTGCAGAAGATATACCAGGCCCCCTAGGGACACCAGATTTTCGGGGCAAAGTCTTTGGCGTGGCCAGCCAGAGAAATCCTGATAGCACGACTAGGGCACATGAAGCAAAGATAGACACAACATCTGGCCGTTTCACCCCAAAACTAGGCTCATTAGAGATTTCCACTGAGTCTGATGATTTTGATCAAAACAAACCAACAAGATTCACCCCAGTTGGCATTGGGGTTGACCATGAGGCAGACTTTCAACAATGGGCTCTTCCCGACTATGCTGGCCAGTTCACCCACAACATGAACTTAGCCCCAGCTGTTGCTCCCAACTTTCCTGGTGAGCAGCTCCTTTTCTTCCGCTCACAGTTGCCATCTTCTGGTGGGCGATCCAACGGGATTCTAGACTGCCTGGTCCCCCAAGAATGGGTACAGCACTTCTACCAAGAATCAGCCCCCTCCCAATCTCAAGTGGCCCTGGTTAGGTATATCAACCCTGACACTGGTAGAGTGTTATTTGAGGCCAAGCTGCACAAATTAGGTTTCATAACTATAGCCAAGAATGGTGACTCTCCAATAACTGTCCCTCCAAATGGATACTTTAGGTTTGAATCTTGGGTGAACCCCTTTTATACACTTGCCCCCATGGGAACTGGGAATGGGCGTAGAAGGATTCAATAA

>GQ849127|-/II.3|2007|AU|Sydney740C

ATGAAGATGGCGTCGAATGACGCCGCTCCATCTAATGATGGTGCCGCCGGCCTCGTCCCAGAGATCAACAGTGAGGCAATGGCGCTAGAGCCAGTGGCGGGTGCAGCGATAGCAGCACCTCTCACTGGTCAGCAAAATATAATTGATCCCTGGATTATGAATAATTTTGTGCAAGCACCTGGTGGTGAGTTTACAGTATCCCCTAGAAATTCCCCTGGTGAAGTTCTTCTTAATTTGGAACTGGGCCCAGAAATAAATCCCTATTTGGCCCATCTTGCTAGAATGTACAACGGTTATGCAGGTGGATTTGAAGTGCAGGTGGTCCTAGCTGGAAATGCGCTTACAGCAGGAAAGATAATCTTTGCAGCTATTCCCCCCAATTTTCCAATTGACAATCTAAGTGCAGCACAGATCACAATGTGCCCACATGTGATTGTGGATGTCAGACAGTTGGAACCAGTCAACCTCCCGATGCCTGACGTTCGCAATAATTTCTTTCATTACAATCAAGGGTCTGATTCGAGATTGCGCCTAATTGCAATGCTATACACACCTCTTAGGGCAAATAATTCTGGGGATGATGTTTTTACTGTGTCTTGCAGAGTGCTAACTAGACCTAGTCCTGACTTCTCATTTAATTTCCTTGTGCCACCTACTGTGGAGTCAAAAACAAAACCCTTTTCCCTCCCTATTCTGACTATCTCTGAAATGTCCAATTCTAGGTTCCCAGTACCAATTGATTCTCTGCACACTAGTCCTACTGAGAATATTGTTGTCCAGTGCCAGAATGGGCGTGTCACCCTTGATGGTGAGTTGATGGGCACCACCCAACTCTTACCTAGCCAAATCTGTGCTTTCAGGGGCGTTCTCACCAGATCAACAAGCAGGGCCAGTGACCAGGCCGATACAGCAACCCCTAGATTGTTTAATTATTATTGGCACATACAATTGGATAATCTAAATGGAACTCCTTATGATCCTGCAGAAGACATACCAGGCCCCCTAGGGACACCAGATTTCCGGGGCAAAGTCTTTGGCGTGGCCAGCCAGAGAAATCCTGATGCCACGACTAGGGCACATGAAGCAAAGATAGACACAACATCTGGCCGTTTCACCCCAAAATTAGGCTCATTAGAGATATCCACTGAATCTGATGATTTTGATCAAAACAAACCAACAAGATTCACCCCAGTTGGCATTGGGGTTGACCATGAGGAAGACTTTCAACAATGGACTCTACCCGACTACGCTGGCCAGTTCACCCACAACATGAACTTAGCCCCAGCTGTTGCTCCCAATTTCCCTGGTGAGCAGCTCCTTTTCTTCCGCTCACAGTTGCCATCTTCTGGTGGGCGATCCAACGGGATTCTAGACTGCCTGGTCCCCCAAGAATGGGTGCAGCACTTCTACCAGGAATCAGCCCCCTCCCAAACTCAAGTGGCCCTGGTTAGGTATATTAACCCTGACACTGGTAGAGTGTTATTTGAGGCCAAGCTGCATAAATTAGGTTTCATGACTATAGCCAAGAATGGTGACTCTCCAATAACTGTCCCTCCAAATGGATACTTTAGGTTTGAATCTTGGGTGAACCCCTTCTACACACTTGCCCCCATGGGAACTGGGAACGGGCGTAGAAGAATTCAATAA

>GQ856466|II.12/II.3|2007|CN|Beijing/55037

ATGAAGATGGCGTCGAATGATGCCACTCCATCTAATGATGGTGCCGCCGGCCTCGTCCCAGAGATCAACAATGAGGCAATGGCGCTAGATCCAGTGGCGGGTGCAGCGATAGCAGCGCCCCTCACTGGCCAGCAAAATATAATTGATCCCTGGATTATGAATAATTTTGTGCAAGCACCTGGTGGTGAGTTTACAGTGTCCCCTAGGAATTCCCCTGGTGAAGTGCTCCTCAATTTGGAATTGGGCCCAGAAATAAATCCCTATTTGGCCCATCTTGCTAGAATGTATAATGGTTATGCAGGTGGATTTGAAGTGCAGGTAGTCCTAGCTGGAAATGCGTTTACAGCAGGAAAGATAATCTTTGCAGCTATACCCCCTAATTTTCCAATTGACAATCTAAGTGCAGCACAGATCACAATGTGCCCACATGTGATTGTGGATGTCAGACAGTTGGAACCGGTCAACCTTCCGATGCCTGACGTTCGCAATAACTTCTTCCATTACAACCAAGGGTCTGATTCCAGATTGCGCCTAGTTGCAATGCTGTATACACCTCTTAGGGCAAATAATTCTGGGGATGATGTTTTTACTGTGTCTTGTAGAGTGCTGACTAGGCCTAGCCCTGACTTTTCATTTAATTTCCTTGTGCCACCTACTGTGGAGTCAAAGACAAAACCCTTCACCCTCCCTATTCTGACTATCTCTGAAATGTCTAATTCTAGGTTCCCAGTGCCAATTGATTCTCTGCACACCAGCCCAACTGAGAATATTGTTGTCCAGTGCCAAAATGGACGCGTCACTCTTGATGGTGAGTTGATGGGCACCACCCAACTCTTACCTAGTCAAATCTGTGCTTTTAGGGGCGTGCTCACCAGATCAACAAGCAGGGCCAGTGACCAGGCCGACACAGCAACCCCTAGATTGTTTAATTATTATTGGCACATACAATTGGATAATCTAAATGGGACTCCTTATGATCCTGCAGAAGACATACCAGGCCCCCTGGGGACACCAGATTTCCGGGGCAAAGTCTTTGGCGTGGCCAGCCAGAGAAATCCCGACAGCACAACTAGAGCACATGAAGCAAAAGTGGACACAACAGCTGGTCGTTTTACCCCAAAACTAGGCTCATTAGAGATATCCACTGAATCTGGTGACTTTGATCAAAACCAACCAACAAGATTCACCCCAGTTGGCATTGGGGTTGACCACGAGGCAGATTTCCAACAATGGTCTCTACCCGACTATTCTGGTCAGTTCACCCACAACATGAACTTAGCCCCAGCTGTTGCTCCCAACTTCCCTGGTGAGCAGCTCCTTTTCTTCCGCTCACAGTTACCATCTTCTGGTGGGCGGTCCAACGGGATTCTAGACTGCCTGGTCCCCCAAGAATGGGTTCAGCACTTCTACCAAGAATCGGCCCCCGCCCAAACCCAGGTGGCCCTGGTTAGATATGTCAACCCTGACACTGGTAGAGTGTTGTTTGAGGCCAAGCTGCACAAATTAGGTTTCATGACTATAGCTAAGAATGGTGACTCTCCAATAACTGTCCCCCCAAATGGATACTTTAGGTTTGAATCTTGGGTGAACCCATTTTATACACTTGCCCCCATGGGAACTGGGAATGGGCGTAGAAGGATTCAATAA

>GQ856467|II.12/II.3|2007|CN|Beijing/55040

ATGAAGATGGCGTCGATTGACGCCGCTCCATCTAATGATGGTGCCGCCGGCCTCGTCCCAGAGATCAACAATGAGGCAATGGCGCTAGATCCAGTGGCGGGTGCAGCGATAGCAGCACCCCTCACTGGCCAGCAAAATATAATTGATCCCTGGATTATGAATAATTTTGTGCAAGCACCTGGTGGTGAGTTTACAGTGTCCCCTAGAAATTCCCCTGGTGAAGTGCTCCTCAATTTGGAATTGGGCCCAGAAATAAATCCCTATTTGGCCCATCTTGCTAGAATGTATAATGGTTTTGCAGGTGGGTTTGAAGTGCAGGTAGTCCTAGCTGGAAATGCGTTTACAGCAGGAAAGATAATCTTTGCAGCTATACCCCCTAATTTCCCAATTGACAATCTAAGCGCAGCACAGATCACAATGTGTCCACATGTGATTGTGGACGTCAGACAGTTGGAACCGGTCAACCTCCCGATGCCTGATGTTCGCAATAACTTCTTCCATTACAACCAAGGGTCTGATTCGAAATTGCGCTTAGTTGCAATGCTGTACACACCTCTCAGGGCAAATAATTCTGGGGATGATGTCTTTACTGTGTCTTGTAGAGTGCTGACTAGGCCTAGCCCTGACTTTTCATTTAATTTCCTTGTGCCACCCACTGTGGAGTCAAAGACAAAACCCTTCACCCTCCCTATTCTGACTATCTCTGAGATGTCTAATTCTAGGTTTCCAGTGCCGATTGATTCACTGCACACTAGCCCAACTGAGAATATTGTTGTCCAATGCCAAAATGGACGCGTCACTCTTGATGGTGAGTTGATGGGCACCACCCAACTCTTACCTAGTCAAATCTGTGCTTTTAGGGGCGTGCTCACCAGATCAACAAGCAGGGCCAGTGACCAGGCCGACACAGCAACCCCTAGATTGTTTAATTATTATTGGCACATACAATTGGATAATCTAAATGGGACTCCTTATGATCCTGCAGAAGACATACCAGGCCCCCTGGGGACACCAGATTTCCGGGGCAAAGTCTTTGGCGTGGCCAGCCAGAGAAATCCCGACAGCACAACTAGAGCACATGAAGCAAAGGTGGACACAACAGCTGGTCGTTTCACCCCAAAACTAGGCTCATTAGAGATATCCACTGAATCTGATGACTTTGATCAAAACCAACCAACAAGGTTCACCCCAGTTGGCATTGGGGTTGACCACGAGGCAGATTTTCAACAATGGTCTTTGCCCGACTATTCTGGTCAGTTCACCCACAACATGAACTTAGCCCCAGCTGTTGCTCCCAACTTCCCTGGTGAGCAGCTCCTTTTCTTCCGCTCACAGTTACCATCTTCTGGTGGGCGATCCAATGGGATTCTAGACTGCCTGGTCCCCCAAGAATGGGTTCAGCACTTCTACCAAGAATCGGCCCCCGCCCAAACCCAAGTGGCCCTGGTCAGATATGTCAACCCAGACACTGGTAGAGTGTTGTTTGAGGCCAAGCTGCACAAATTAGGTTTCATGACTATAGCTAAGAATGGTGACTCTCCAATAACTGTCCCCCCAAATGGATACTTTAGGTTTGAATCTTGGGTGAACCCATTTTATACACTTGTCCCCATGGGAACTGGGAATGGGCGTAGAAGGATTCAATAA

>GU980585|II.12/II.3|2006|KR|CBNU1

ATGAAGATGGCGTCGAATGACGCCACTCCATCTAATGATGGTGCCGCCGGCCTCGTCCCAGAGATCAGTAATGAGGCAATGGCGCTAGATCCAGTGGCGGGTGCAGCGATAGCAGCGCCCCTCACTGGTCAGCAAAATATAATTGATCCCTGGATTATGAATAATTTTGTGCAAGCACCTGGTGGTGAGTTTACAGTATCCCCTAGGAATTCCCCTGGTGAAGTGCTTCTCAATTTGGAATTGGGCCCAGAAATAAATCCCTATTTGGCCCATCTTGCTAGAATGTATAATGGTTATGCAGGTGGGTTTGAAGTGCAGGTAGTCCTAGCTGGAAATGCGTTTACAGCAGGAAAGATAATCTTTGCAGCTATACCCCCTAATTTCCCAATTGATAATCTAAGCGCAGCACAGATCACAATGTGCCCACATGTGATTGTGGATGTCAGACAGTTGGAACCGGTCAACCTTCCGATGCCTGACGTTCGCAATAACTTCTTCCACTACAACCAAGGGTCTGATTCGAGATTGCGCTTAGTTGCAATGCTGTATACACCTCTTAGGGCAAATAATTCTGGGGATGATGTTTTTACTGTGTCTTGTAGGGTGCTGACTAGGCCTAGCCCTGACTTTTCATTTAATTTCCTTGTGCCACCTACTGTGGAGTCAAAGACAAAACCCTTCACCCTCCCTATTCTGACTATCTCTGAAATGTCCAATTCTAGGTTTCCAGTGCCGATTGATTCTCTGCACACCAGCCCAACTGAAAATGTTGTTGTCCAGTGCCAAAATGGACGCGTCACTCTTGATGGTGAGTTGATGGGCACCACCCAACTCTTACCTAGTCAAATCTGTGCTTTTAGGGGCGTGCTCACCAGATCAACAAGCAGGGCCAGTGACCAGGCCGACACAGCAACCCCTAGGTTGTTTAATTATTATTGGCACATACAATTGGATAATCTAAATGGGACTCCTTATGATCCTGCAGAAGACATACCAGGCCCCCTAGGGACACCAGATTTCCGGGGCAAAGTCTTTGGCGTGGCCAGCCAGAGAAATCCCGACAGCACAACTAGAGCACATGAAGCAAAGATAGACACAACAGCTGGTCGTTTTACCCCAAAACTAGGCTCATTAGAGATATCCACTGAATCTGGTGACTTTGATCAAAACCAACCAACAAGATTCACCCCAGTTGGCATTGGGGTTGACCACGAGGCAGATTTCCAACAATGGTCTTTACCCGACTATTCTGGTCAGTTCACCCACAACATGAACTTAGCCCCAGCTGTTGCTCCCAACTTCCCTGGTGAGCAGCTCCTTTTCTTCCGCTCACAGTTACCATCTTCTGGTGGGCGATCCAACGGGATTCTAGACTGCCTGGTCCCCCAAGAATGGGTTCAGCACTTCTACCAAGAATCGGCCCCCGCCCAAACTCAAGTGGCCCTGGTTAGGTATGTCAACCCTGACACTGGTAGAGTGTTGTTTGAGGCCAAGCTGCACAAATTGGGTTTCATGACTATAGCTAAGAATGGTGACTCTCCAATAACTGTCCCCCCAAATGGATACTTTAGGTTTGAATCTTGGGTGAACCCATTTTATACACTTGCCCCCATGGGAACTGGGAATGGGCGTAGAAGGATTCAATAA

>GU991355|II.12/II.3|2009|CN|SH312

ATGAAGATGGCGTCGAATGACGCCGCTCCATCTAACGATGGTGCCGCCGGCCTCGTCCCAGAGATCAACAATGAGGCAATGGCGCTAGAACCAGTGGCGGGTGCAGCGATAGCAGCACCCCTCACTGGCCAGCAAAATATAATTGATCCCTGGATTATGAATAATTTTGTGCAAGCACCTGGTGGTGAGTTTACAGTGTCTCCTAGGAATTCCCCTGGTGAAGTGCTTCTCAATTTGGAATTGGGCCCAGAAATAAATCCCTATTTGGCCCATCTTGCTAGAATGTATAATGGTTATGCAGGTGGGTTTGAAGTGCAGGTAGTCCTAGCTGGAAATGCGTTTACAGCAGGAAAGATAATTTTTGCAGCTATACCCCCTAACTTCCCAATTGACAATCTAAGCGCAGCACAGATCACAATGTGCCCACATGTGATTGTGGATGTCAGACAGTTGGAACCGGTCAACCTTCCGATGCCTGACGTTCGCAATAACTTCTTCCATTACAACCAAGGGTCTGATTCGAGATTGCGCTTAGTTGCAATGCTGTATACACCTCTTAGGGCAAATAATTCTGGGGATGATGTTTTTACTGTGTCTTGTAGAGTGCTGACTAGGCCTAGCCCTGACTTTTCATTTAACTTCCTTGTGCCACCCACTGTGGAGTCAAAGACAAAACCCTTCACCCTCCCTATTTTGACTATCTCTGAAATGTCTAATTCTAGGTTTCCAGTGCCGATTGATTCTCTGCACACCAGCCCAACTGAGAATATTGTTGTCCAGTGCCAAAATGGGCGCGTCACTCTTGATGGTGAGTTGATGGGCACCACCCAACTCTTACCTAGTCAAATCTGTGCTTTTAGGGGCGTGCTCACCAGATCAACAAGCAGGGCCAGTGACCAGGCCGACACAGCAACCCCTAGATTGTTTAATTATTATTGGCACATACAATTGGATAATCTAAATGGGACTCCTTATGATCCTGCAGAAGACATACCAGGCCCCCTGGGGACACCAGACTTCCGGGGCAAAGTCTTTGGCGTGGCCAGCCAGAGAAATCCCGACAGTACAACTAGAGCACATGAAGCGAAGGTGGACACAACAGCTGGTCGCTTTACCCCAAAACTAGGCTCATTGGAGATATCCACTGAATCTGGTGACTTTAATCAAAACCAACCAACAAGATTCACCCCAGTTGGCATTGGGGTTGACCACGAGGAAGACTTCCAACAATGGTCCTTACCCGACTATTCTGGTCAGTTCACTCACAACATGAACTTAGCCCCAGCTGTTGCTCCCAACTTCCCTGGTGAGCAGCTCCTTTTCTTCCGCTCACAGTTACCATCTTCTGGTGGGCGATCCAATGGGATTCTAGACTGCCTGGTCCCCCAAGAATGGGTTCAGCACTTCTACCAAGAATCGGCCCCCACCCAAACCCAGGTGGCCCTGGTTAGATATGTCAACCCTGACACTGGTAGAGTGTTGTTTGAGGCCAAGCTGCACAAATTAGGTTTCATGACTATAGCTAAGAATGGTGACTCTCCAATAACTGTCCCCCCAAATGGATACTTTAGGTTTGAATCTTGGGTGAACCCATTTTATACACTTGCCCCCACGGGAACTGAGAATGGGCGTAGAAGGGTTCAATAA

>U22498|II.3/II.3|<1995|US|MX

ATGAAGATGGCGTCGAATCGCGCTGCTCCATCTAATGATGGTGCCGCCTGCCTCGTCCCAGAGATCAACAATGAGGCAATGGCGCTAGAGCCAGTGGCGGGTGCAGCGATAGCAGCGCCCCTCACTGGCCAGCAAAATATAATTGATCCCTGGATTATGAATAATTTTGTGCAAGCACCTGGTGGTGAGTTTACAGTGTCACCCAGGAATTCCCCTGGTGAAGTGCTTCTTAATTTGGAATTAGGTCCAGAAATAAATCCTTATTTGGCTCATCTTGCTAGAATGTACAATGGTTATGCAGGTGGATTTGAAGTGCAAGTGGTCCTGGCTGGAAATGCGTTTACAGCAGCAAAAATTATCTTTGCAGCTATACCCCCTAACTTCCCTATTGACAATCTGAGCGCGGCACAGATCACAATGTGCCCGCATGTGATTGTGGATGTCAGGCAGTTGGAACCAATCAATCTTCCGATGCCTGATGTCCGCAACAATTTCTTTCATTATAATCAAGGTTCTGATTCAAGATTACGCTTAATTGCAATGCTGTATACACCTCTTAGGGCAAATAATTCTGGAGATGATGTTTTCACTGTGTCTTGTAGGGTGTTAACTAGGCCTAGCCCTGATTTCTTATTCAATTTTCTTGTCCCACCCACTGTGGAATCAAAGACAAAACCTTTTACCCTCCCCATTTTAACCATCTCTGAAATGTCTAATTCCAGGTTTCCGGTGCCAATTGACTCTCTGCACACCAGCCCAACTGAGAATATAGTTGTCCAGTGCCAAAATGGGCGCGTCACTCTTGACGGTGAGTTGATGGGCACCACCCAACTCTTACCGAGCCAAATATGTGCTTTCAGGGGCACACTCACTGGATCAACAAGCAGGGCCAGTGACCAAGCCGACACACCAACCCCTAGGCTATTCAACCATCATTGGCACATACAATTGGATAATCTAAATGGAACTCCCTACGACCCTGCAGAGGACATACCAGCTCCTTTGGGCACACCAGACTTCCGGGGCAAGGTCTTTGGCGTAGCCGGCCAGAGAAACCCCGACAGCACAACAAGGGCACATGAAGCAAAAGTGGACACAACATCTGGCCGCTTCACCCCAAAATTGGGCTCCTTAGAAATAACCACTGAATCTGATGACCTTGACCTAAGCCAGCCAACAAAATTCACCCCAGTTGGCATTGGAGTTGACAATAGGGCAGAATTTCAGCAATGGTCCTTACCTGACTATTCCGGTCAGTTTACTCACAACATGAACTTGGCCCCAGCTGTCGCCCCCAATTTTCCTGGTGAACAGCTACTTTTCTTCCGATCACAGCTGCCATCCTCTGGTGGGCGGTCTAACGGGGTTCTAGACTGCCTGGTCCCCCAGGAATGGGTTCAACACTTTTACCAAGAATCAGCCCCCGCCCAAACACAGGTGGCCCTGGTTAGGTATGTCAACCCTGACACTGGTAGAGTGCTATTTGAGGCCAAGCTACACAAATTGGGTTTTATGACTGTAGCAAAGAATGGTGACTCCCCAATAACTGTCCCTCCAAATGGTTATTTTAGATTTGAATCTTGGGTTAACCCCTTTTACACACTTGCCCCCATGGGAACTGGAAACGGGCGTAGAAGGATTCAATAA

>HM072040|-/II.3|1991|US|CHDC5365

ATGAAGATGGCGTCGAATGACGCTGCTCCATCTAATGATGGTGCCGCCGGCCTCGTCCCAGAGATCAACAATGAGGCAATGGCGCTAGAGCCAGTGGCGGGTGCAGCGATAGCAGCGCCCCTCACTGGCCAGCAAAATATAATTGATCCCTGGATTATGAATAATTTTGTGCAAGCACCTGGTGGTGAGTTTACAGTGTCACCTAGGAATTCCCCTGGTGAAGTGCTTCTTAATTTGGAATTAGGTCCAGAAATAAATCCCTATTTGGCTCATCTTGCTAGAATGTACAATGGTTATGCAGGTGGATTTGAAGTGCAAGTGGTCCTAGCTGGAAATGCGTTTACAGCAGGAAAGGTTATCTTTGCAGCTATACCCCCTAATTTCCCTATTGACAATCTGAGCGCGGCACAGATCACAATGTGCCCGCACGTGATTGTGGATGTCAGGCAGTTGGAACCAATCAATCTCCCGATGCCTGATGTCCGCAACAATTTCTTTCATTATAATCAAGGTTCTGATTCAAGATTACGTTTGATTGCAATGCTGTATACACCTCTTAGGGCAAATAATTCTGGAGATGATGTTTTCACTGTGTCTTGTAGGGTGTTAACTAGGCCTAGCCCTGATTTCTCATTCAATTTTCTTGTCCCACCCACTGTGGAATCAAAGACAAAACCTTTTACCCTCCCCATTTTAACCATCTCTGAAATGTCTAATTCCAGGTTTCCGGTGCCAATTGACTCTCTGCACACCAGCCCAACTGAGAATATAGTTGTCCAGTGCCAAAATGGGCGCGTCACTCTTGACGGTGAGTTAATGGGCACCACCCAACTCTTACCGAGCCAAATATGTGCTTTCAGGGGCACACTCATTAGATCAACAAGCAGGGCCAGTGACCAAGCCGACACATCAACCCCTAGGCTATTCAACTATTATTGGCACATACAATTGGATAATCTAAATGGAACTCCCTACGACCCTGCAGAGGACATACCAGCTCCTTTGGGCACACCAGACTTCCGGGGCAAGGTCTTTGGCGTAGCCAGCCAGAGAAACCCCGACAGCACAACAAGGGCACATGAAGCAAAAGTGGACACAACATCTGGCCGCTTCACCCCAAAATTGGGTTCCTTAGAAATAATCACTGAATCTGATGACTTTGACTTAAACCAGTCAACAAAATTCACCCCAGTTGGCATTGGAGTTGACAATGAGGCAGAATTTCAGCAGTGGTCCTTACCCAACTATTCTGGTCAGTTTACTCATAATATGAACTTAGCCCCAGCTGTCGCCCCCAACTTTCCTGGTGAACAGCTACTTTTCTTCCGATCACAGCTGCCATCCTCTGGTGGGCGATCTAACGGGGTGCTAGATTGCCTGGTCCCCCAGGAATGGGTTCAACACTTTTACCAAGAATCAGCCCCCGCCCAAACACAGGTGGCCCTGGTTAGGTATGTCAACCCTGACACTGGCAGAGTGCTATTTGAGGCCAAGCTACACAAATTGGGTTTTATGACTATAGCAAAGAATGGTGACTCCCCAATAACTGTCCCTCCAAATGGATATTTTAGATTTGAATCTTGGGTTAACCCCTTTTACACACTTGCCCCCATGGGAACTGGAAACGGGCGTAGAAGGATTCAATAA

>HM072041|-/II.3|1990|US|CHDC5261

ATGAAGATGGCGTCGAATGACGCTGCTCCATCTAACGATGGTGCCGCCGGCCTCGTACCAGAGATCAATAATGAGGCAATGGCGCTAGAGCCAGTGGCGGGTGCAGCGATAGCAGCACCCCTCACTGGTCAGCAAAATATAATAGATCCCTGGATTATGAATAATTTTGTGCAAGCACCTGGTGGTGAGTTTACAGTGTCACCTAGGAACTCCCCTGGTGAAGTGCTTCTTAACTTAGAGTTAGGCCCAGAAATAAACCCCTATTTGGCACACCTTGCTAGGATGTACAATGGTTATGCAGGTGGATTTGAAGTGCAGGTTGTCCTGGCTGGAAATGCGTTTACAGCAGGGAAAGTGATCTTTGCAGCTATACCCCCTAATTTTCCAATTGATAATCTGAGTGCAGCACAGATTACAATGTGTCCACATGTGATTGTGGACGTCAGGCAATTGGAACCAATCAATCTCCCGATGCCTGATGTCCGCAACAATTTCTTCCATTACAATCAAGGGTCTGATTCGAGGTTACGCTTAATTGCAATGCTGTACACACCTCTTAGGGCAAATAATTCTGGAGATGATGTTTTCACTGTGTCCTGCAGGGTGTTAACTAGGCCTAGCCCTGATTTCTCATTCAATTTCCTTGTCCCGCCCACTGTGGAGTCAAAGACAAAACCCTTCACCCTTCCCATTCTAACTATTTCTGAAATGTCTAATTCCAGGTTTCCAGTGCCAATTGACTCTCTGCACACCAGCCTGACTGAAAATATTGTTGTCCAGTGCCAGAATGGGCGCGTCACTCTTGACGGCGAGCTAATGGGCACCACCCAACTCTTGCCGAGTCAAATATGCGCTTTCAGGGGCACTCTCACCAGATCAACAAGCAGGGCCAGTGACCAAGCCGACACAGCAACCCCTAGGCTTTTCAACTATTATTGGCATATACAATTGAATAACCTAAATGGAACCCCCTACGACCCTGCAGAGGACATACCAGCCCCTTTGGGGACGCCAGACTTTCGGGGCAAGGTCTTTGGCGTAGCCAGCCAGAGAAACCCTGATAGCACAACAAGAGCACATGAAGCAAAAGTGGACACAACATCTGGCCGTTTCGCCCCAAAATTGGGCTCCCTGGAAATATCCACTGAAACCGACGACTTTAACCAAAACCAACCAACAAGATTTACCCCAGTTGGCATTGGGGTTGATAATGAGGCAGATTTTCAACAATGGTCCTTGCCTGACTACTCCGGTCAGTTCACCCACAACATGAACTTGGCCCCAGCTGTCGCACCCAACTTCCCCGGTGAACAGCTTCTCTTCTTCCGCTCACAGCTGCCATCCTCTGGTGGGCGGTCCAGTGGGATTCTAGACTGCCTGGTCCCCCAAGAATGGGTTCAACACTTCTACCAAGAATCAGCCCCTGCGCAAACACAGGTGGCTCTGGTCAGGTATGTCAACCCTGATACTGGTAGAGTGTTATTTGAGGCCAAGCTACATAAATTAGGTTTCATGACCATAGCTAAGAATGGTGACTCTCCAATAACTGTCCCTCCAAATGGATACTTTAGGTTTGAATCTTGGGTGAACCCCTTTTATACACTAGCCCCCATGGGAACTGGAAATGGGCGTAGAAGGATTCAATAA

>HM072042|-/II.3|1979|US|CHDC4671

ATGAAGATGGCGTCGAATGACGCTGCTCCATCTAACGATGGTGCCGCCGGCCTCGTCCCAGAGATCAACAATGAGGCAATGGCGCTAGAGCCAGTGGCAGGTGCGGCAATAGCAGCACCTCTCACTGGTCAGCAAAATATAATTGATCCCTGGATTATGAATAATTTTGTGCAAGCACCTGGTGGTGAGTTTACAGTGTCACCTAGGAACTCTCCTGGTGAAGTACTTCTTAATTTAGAATTAGGTCCAGAAATAAACCCCTATTTGGCTCACCTTGCCAGGATGTACAATGGTTATGCAGGTGGGTTTGAGGTGCAGGTAGTCCTGGCTGGAAATGCGTTTACAGCAGGAAAGGTGATCTTTGCAGCTATACCCCCCAATTTCCCAATTGACAATTTGAGCGCAGCACAGATTACAATGTGCCCGCATGTGATTGTGGATGTTAGACAATTGGAACCAATCAACCTTCCGATGCCTGACGTCCGTAACAATTTCTTTCATTATAATCAAGGGTCTGATTCGAGGTTGCGTTTAATTGCAATGTTATATACACCCCTTAGGGCAAATAATTCAGGAGATGATGTTTTCACTGTGTCTTGTAGGGTGTTAACTAGGCCTAGCCCTGATTTCTCATTCAACTTTCTTGTTCCACCTACTGTAGAGTCAAAGACAAAGCCTTTCACCCTCCCCATTTTGACCATTTCCGAAATGTCTAATTCTAGATTTCCAGTGCCAATTGATTCTCTGCACACCAGCCCGACTGAGAACATTGTTGTCCAGTGCCAAAATGGTCGAGTCACCCTTGATGGTGAGTTAATGGGCACCACTCAACTCCTGCCGAGTCAAATATGTGCTTTCAGGGGCACGCTCACCAGATCAACAAGCAGGACCGGTGACCAAGCTGACACGGCATCCCCTAGATTGTTCAACTATTATTGGCACATACAATTGGATAATCTAAATGGAACCCCCTATGACCCTGCAGAAGACATACCAGCCCCCTTGGGAACACCAGATTTCCGGGGCAAGGTCTTTGGCGTAGCTAGCCAGAGAAACCCTGACAGCACAACAAGAGCACATGAAGCAAAAGTGGACACAACATCTGGTCGCTTTGCCCCAAAATTGGGTTCCCTAGAAATATCCACTGAATCTGATGACTTTGACTCAAACCAACCAACAAGGTTTACCCCAGTTGGCATTGGGGTTGACCGAGAGACAGATTTTCAGCAATGGACCTTACCTGAATACTCCGGTCAGTTCACACATAACATGAATCTAGCCCCAGCTGTCGCCCCCAACTTCCCTGGTGAGCAGCTTCTTTTCTTCCGCTCGCAGTTGCCATCTTCTGGCGGGCGGTCTGGCGGGATTCTGGACTGCCTGGTTCCCCAGGAATGGGTTCAACACTTCTACCAGGAATCAGCCCCTGCCCAAACACAGGTAGCCCTAGTTAGGTATGTTAACCCTGACACTGGCAGAGTGCTATTTGAGGCCAAACTACATAAATTGGGCTTCATGACTATAGCTAAAAATGGTGACTCTCCAATAACTGTCCCTCCAAATGGATACTTTAGGTTTGAATCTTGGGTGAACCCCTTTTATACACTTGCCCCCATGGGAACTGGAAATGGGCGTAGAAGGATTCAATAA

>HM072043|-/II.3|1988|US|CHDC4090

ATGAAGATGGCGTCGAATGACGCTGCTCCATCTAATGATGGTGCCGCCGGCCTCGTCCCAGAGATCAACAATGAGGCAATGGCGCTAGATCCAGTGGCGGGTGCAGCTATAGCAGCACCCCTCACTGGCCAGCAAAATATAATTGATCCCTGGATTATGAATAACTTTGTGCAAGCACCTGGTGGTGAGTTTACAGTGTCACCTAGGAATTCCCCTGGTGAAGTGCTTCTTAATTTGGAATTAGGTCCAGAAATAAATCCCTATTTGGCTCATCTTGCTAGAATGTACAATGGTTATGCAGGTGGGTTTGAAGTGCAAGTGGTCCTGGCTGGAAATGCGTTTACAGCAGGAAAGGTTATCTTTGCAGCTATACCCCCCAACTTCCCTATTGATAATCTGAGCGCAGCACAGATCACAATGTGCCCGCATGTGATTGTGGATGTCAGGCAGTTGGAACCAATCAATCTTCCGATGCCTGATGTTCGCAACAATTTCTTTCATTATAATCAAGGGTCTGATTCAAGATTACGTTTAATTGCAATGCTGTATACACCTCTTAGGGCAAATAATTCTGGAGATGATGTTTTCACTGTGTCTTGTAGGGTGTTAACTAGGCCTAGCCCTGATTTCTCATTCAATTTTCTTGTCCCACCCACTGTGGAATCAAAGACAAAACCTTTTACCCTCCCCATTTTAACCATCTCTGAAATGTCTAATTCCAGGTTTCCGGTGCCAATTGACTCTCTACACACCAGCCCAACTGAGAATATCGTTGTCCAGTGCCAAAATGGGCGCGTCACTCTTGACGGTGAGTTAATGGGCACCACTCAACTCTTACCGAGCCAAATATGTGCTTTCAAGGGAACACTTACTAGATCAACAAACAGGGTCAGTGACCAAGCCGACACAGCAACCCCCAGGCTATTTAACTATCATTGGCACATACAATTGGATAATCTAAATGGAACCCCCTACGACCCTGCAGAGGACATACCAGCTCCTTTGGGTACACCAGACTTCCGGGGCAAAGTCTTTGGCGTAGCCAGCCAGAGAAACCCCRACAGCACAACAAGGGCACATGAAGCAAAAGTGGACACAACATCTGGCCGCTTCACCCCAAAATTGGGCTCCCTAGAAATAACCACTGAATCTAATGACTTTGACTCAAACCAGCCAACAAAATTCACCCCAGTTGGCATTGGAGTTGACAATGAGGTAGATTTTCAGCAATGGTCCTTACCTGACTATTCCGGTCAGCTTACTCATAACATGAACTTAGCCCCAGCTGTCGCCCCCAATTTTCCTGGTGAACAGCTTCTTTTCTTCCGATCACAGCTGCCATCTTCTGGTGGGCGGTCTAACGGGACTCTAGACTGCCTGGTCCCCCAGGAATGGGTTCAACATTTCTATCAAGAATCAGCCCCCGCCCAAACACAGGTGGCCCTGGTTAGGTATGTCAACCCTGACACTGGTAGAGTGCTATTTGAGGCCAAGCTACACAAATTGGGTTTTATGACTATAGCAAAGAATGGTGACTCCCCAATAACTGTCCCTCCAAATGGGTATTTTAGATTTGAATCTTGGGTTAACCCCTTTTACACACTTGCCCCCATGGGAACTGGAAACGGGCGTAGAAGGATTCAATAA

>HM072044|-/II.3|1988|US|CHDC4031

ATGAAGATGGCGTCGAATGACGCTGCTCCATCTAATGATGGTGCCGCCGGCCTCGTCCCAGAGATCAACAATGAGGCAATGGCGCTAGATCCAGTGGCGGGTGCAGCGATAGCAGCACCCCTCACTGGCCAGCAAAATATAATTGATCCCTGGATTATGAACAATTTTGTGCAAGCACCTGGTGGTGAGTTTACAGTGTCACCTAGGAATTCCCCTGGTGAAGTGCTTCTTAATTTGGAATTAGGTCCAGAAATAAATCCCTATTTGGCTCATCTTGCTAGAATGTATAATGGTTATGCAGGTGGGTTTGAAGTGCAAGTGGTCCTGGCTGGAAATGCGTTTACAGCAGGAAAGGTGATCTTTGCAGCTATACCCCCCAACTTCCCTATTGACAATCTGAGCGCAGCACAGATCACAATGTGCCCGCACGTGATTGTGGATGTCAGGCAGTTGGAACCAATCAATCTTCCGATGCCTGATGTCCGCAACAATTTCTTTCATTATAATCAAGGGTCTGATTCAAGATTACGTTTAATTGCAATGCTGTATACACCTCTTAGGGCAAATAATTCTGGAGATGATGTTTTCACTGTGTCTTGTAGGGTGTTAACTAGGCCTAGCCCTGATTTCTCATTCAATTTTCTTGTCCCACCCACTGTGGAATCAAAGACAAAACCTTTTACCCTCCCCATTTTAACCATCTCTGAAATGTCTAATTCCAGGTTTCCGGTGCCAATTGACTCTCTGCACACCAGCCCAACTGAGAGTATCGTTGTCCAGTGTCAAAATGGGCGCGTCACTCTTGACGGTGAGTTAATGGGCACCACCCAACTCTTACCGAACCAAATATGTGCTTTCAGGGGCACACTTACTAGGTCAACAAACAGGGCCAGTGATCAAGCCGACACAGCAACCCCCAGGCTATTCAACCATCATTGGCACATACAATTGGATAATCTAAATGGAACCCCCTACGACCCTGCAGAGGACATACCAGCTCCTTTGGGCACACCAGACTTCCGGGGCAAAGTCTTTGGCGTAGCCAGCCAGAGAAACCCCGACAGCACAACAAGGGCACATGAAGCAAAAGTGGACACAACATCTGGCCGCTTCACCCCAAAATTGGGCTCCCTAGAAATAACCACTGAATCTGATGACTTTGACCCAAACCAGTCAACAAAATTCACCCCAGTTGGCATTGGAGTTGACAATGAGGCAGATTTTCAGCAATGGTCCTTACCTGACTATTCCGGTCAGTTTACTCATAACATGAACTTAGCCCCAGCTGTCGCCCCCAATTTTCCTGGTGAACAGCTTCTTTTCTTCCGATCACAGCTGCCATCTTCTGGTGGGCGGTCTAACGGGATTCTAGACTGCCTGGTCCCCCAGGAATGGGTTCAACACTTCTACCAGGAATCAGCCCCCGCCCAAACACAGGTGGCCCTGGTTAGGTATGTCAACCCTGACACTGGTAGAGTGCTATTTGAGGCCAAGCTACACAAATTGGGTTTTATGACTATAGCAAAGAATGGTGACTCCCCAATAACTGTCCCTCCAAATGGGTATTTTAGATTTGAATCTTGGGTTAACCCCTTTTACACACTTGCCCCCATGGGAACTGGAAACGGGCGTAGAAGGATTCAATAA

>HM072045|-/II.3|1975|US|CHDC2005

ATGAAGATGGCGTCGAATGACGCTGCTCCATCTAACGATGGTGCCGCCGGCCTCGTCCCAGAGATCAACAATGAGGCAATGGCGCTAGAGCCAGTGGCAGGTGCAGCAATAGCAGCACCTCTCACTGGCCAGCAAAACATAATTGATCCCTGGATTATGAATAATTTTGTGCAAGCACCTGGTGGTGAGTTTACAGTGTCACCTAGGAACTCCCCTGGTGAAGTACTTCTTAATTTAGAATTAGGTCCAGAAATAAACCCCTATTTGGCTCACCTTGCTAGGATGTACAATGGTTATGCAGGTGGGTTTGAGGTGCAGGTAGTCCTGGCTGGAAATGCGTTTACAGCAGGAAAGGTGATCTTTGCAGCTATACCCCCCAATTTCCCAATTGACAATCTGAGCGCAGCACAGATTACAATGTGCCCGCATGTGATTGTGGATGTCAGACAATTGGAACCAATCAACCTTCCGATGCCTGATGTCCGTAATAATTTCTTTCATTATAATCAAGGGTCTGATTCGAGGTTGCGTTTAATTGCAATGTTATACACACCTCTTAGGGCAAATAATTCAGGAGATGATGTTTTCACTGTGTCTTGTAGGGTATTAACTAGGCCTAGCCCTGATTTCTCATTCAATTTTCTCGTTCCACCTACTGTGGAATCAAAGACAAAGCCTTTCACCCTCCCCATTTTGACTATTTCTGAAATGTCTAATTCTAGATTTCCAGTGCCAATTGACTCTCTGCACACTAGCCCGACTGAGAACATTGTTGTCCAGTGCCAAAATGGGCGCGTCACCCTTGACGGTGAGTTAATGGGCACCACTCAACTCTTGCCGAGTCAAATATGTGCTTTCAGGGGCACGCTCACCAGATCAACAAGCAGGGCCGGTGACCAAGCTGACACGGCAACCCCTAGATTGTTCAATTATTATTGGCACATACAATTGGATAACCTAAATGGAACCCCCTATGACCCTGCAGAAGACATACCAGCCCCTTTGGGAACACCAGACTTCCGGGGCAAGGTCTTTGGCGTAGCTAGCCAGAGAAACCCTGACAGCACAACAAGAGCACATGAAGCAAAAGTGGACACAACATCTGGTCGCTTCGCCCCGAAATTGGGTTCCCTAGAAATATCCACTGAATCCAGTGACTTTGACTCAAACCAACCAACAAGGTTCACCCCAGTTGGCATTGGGGTTGACAATGAGGCAGATTTTCAGCAATGGTCCTTACCTGACTACTCCGGTCAGTTCACTCATAACATGAACTTAGCCCCAGCTGTCGCCCCCAATTTCCCTGGTGAGCAGCTTCTTTTCTTCCGCTCACAGTTGCCATCTTCTGGTGGGCGGTCTAACGGGATTCTGGACTGCCTGGTTCCCCAGGAATGGGTTCAACACTTCTACCAGGAATCAGCCCCTGCCCAAACACAGGTAGCCCTGGTTAGATATGTTAACCCTGACACTGGTAGAGTGCTATTTGAGGCCAAGCTACACAAATTGGGCTTCATGACTATAGCTAAGAATGGTGACTCTCCAATAACTGTCCCTCCAAATGGGTACTTTAGGTTTGAATCTTGGGTGAACCCCTTTTATACACTTGCCCCCATGGGAACTGGAAATGGGCGTAGAAGGATTCAATAA

>HM072046|-/II.3|1976|US|CHDC32

ATGAAGATGGCGTCGAATGACGCTGCTCCATCTAACGATGGTGCCGCCGGCCTCGTCCCAGAGATCAACAATGAGGCAATGGCGCTAGAGCCAGTGGCAGGTGCAGCAATAGCAGCACCTCTCACTGGCCAGCAAAATATAATTGATCCCTGGATTATGAATAATTTTGTGCAAGCACCTGGTGGTGAGTTTACAGTGTCACCTAGGAACTCCCCTGGTGAAGTACTCCTTAATTTAGAATTAGGTCCAGAAATAAACCCCTATTTGGCTCACCTTGCTAGGATGTACAATGGTTATGCAGGTGGGTTTGAGGTGCAGGTAGTCCTGGCTGGAAATGCGTTTACAGCAGGAAAGGTGATCTTTGCAGCTATACCCCCCAATTTCCCAATTGATAATCTGAGCGCAGCACAGATTACAATGTGCCCGCATGTGATTGTGGATGTCAGACAATTGGAACCAATCAACCTTCCGATGCCTGATGTCCGTAATAACTTCTTTCATTATAATCAAGGGTCTGACTCGAGGTTGCGTCTAATTGCAATGTTGTATACACCTCTTAGGGCAAATAATTCAGGAGATGATGTTTTCACTGTGTCTTGTAGGGTATTAACTAGGCCTAGCCCTGATTTCTCATTCAATTTTCTCGTTCCGCCTACTGTAGAATCAAAAACAAAGCCTTTCACCCTCCCCATTTTGACTATTTCTGAAATGTCTAATTCTAGATTTCCAGTGCCAATTGACTCTCTGCACACCAGCCCGACTGAGAACATTGTTGTCCAGTGCCAAAATGGGCGCGTCACCCTTGACGGTGAGTTAATGGGCACCACTCAACTCTTGCCGAGTCAAATATGTGCTTTCAGGGGCACGCTCACCAGATCAACAAGCAGGGCCGGTGACCAAGCTGACACGGCAACCCCTAGATTGTTCAATTATTATTGGCACATACAATTGGATAATCTAAATGGAACCCCCTATGACCCTGCAGAAGACATACCAGCCCCTTTGGGAACACCAGACTTCCGGGGCAAGGTCTTTGGCGTAGCCAGCCAGAGAAACCCTGACAGCACAACAAGAGCACATGAAGCAAAAGTGGACACAACATCTGGTCGCTTCGCCCCGAAATTGGGTTCCCTAGAAATATCCACTGAATCCAGTGACTTTGACTCAAACCAACCAACAAGGTTCACCCCAGTTGGCATTGGGGTTGACAATGAGGCAGATTTTCAACAATGGTCCTTACCTGACTACTCCGGTCAGTTCACTCATAACATGAACTTAGCCCCAGCTGTCGCCCCCAATTTCCCTGGTGAGCAGCTTCTTTTCTTCCGCTCACAGTTGCCATCTTCTGGTGGGCGGTCTCACGGGATTCTGGACTGCCTGGTTCCCCAGGAATGGGTTCAACACTTCTACCAGGAATCAGCCCCTGCCCAAACACAGGTAGCCCTGGTTAGGTATGTTAACCCTGACACTGGTAGAGTGCTATTTGAGGCCAAGCTACACAAATTGGGCTTCATGACTATAGCTAAGAATGGTGACTCTCCAATAACTGTCCCTCCAAATGGGTACTTTAGGTTTGAATCTTGGGTGAACCCCTTTTATACACTTGCCCCCATGGGAACTGGAAACAAGCGTAGAAGGATTCAATAA

>JN565063|-/II.3|2010|US|Milwaukee009

ATGAAGATGGCGTCGAATGACGCTGCTCCATCTAATGATGGTGCCGCCGGCCTCGTACCAGAGATCAATAATGAGGCAATGGCGCTAGAACCAGTGGCGGGTGCAGCGATAGCAGCACCCCTTACTGGCCAGCAAAATATAATAGATCCCTGGATTATGAATAATTTTGTGCAAGCACCTGGTGGTGAGTTTACAGTGTCACCTAGGAACTCCCCTGGTGAAGTACTTTTAAATTTAGAATTAGGCCCAGAAATAAATCCCTATCTGGCACATCTTGCTAGGATGTACAATGGTTATGCAGGTGGGTTTGAGGTGCAGGTGGTCCTGGCTGGAAATGCGTTTACAGCAGGGAAAGTGATCTTTGCAGCTATACCCCCCAATTTCCCAACTGACAATTTGAGTGCAGCGCAGATTACAATGTGTCCTCATGTGATTGTGGACGTCAGGCAGTTGGAACCAATCAACCTCCCAATGCCTGATGTCCGCAATAATTTCTTTCATTACAATCAAGGTTCTGATTCGAGATTACGCTTAGTTGCAATGTTGTATACACCCCTTAGGGCAAATAATTCTGGAGATGATGTTTTCACTGTGTCATGTAGGGTGTTGACTAGGCCCAGTCCTGATTTCTCATTCAACTTCCTCGTCCCACCCACTGTGGAATCAAAGACAAAACCTTTTACCCTCCCTATTTTGACTATCTCTGAGATGTCTAATTCTAGATTTCCAGTGCCAATTGACTCTCTGCACACCAGTCCGACTGATAACATTGTTGTCCAGTGCCAAAATGGGCGCGTCACCCTTGATGGTGAGCTAATGGGCACCACTCAACTCTTGCCGAGTCAGATATGTGCTTTTAGGGGTGTGCTCACCAGATCAACAAGCAGGGCCAGTGACCAAGCTGACACAGCAACCCCCAGACTCTTCAACTACCACTGGCACATACAACTGGACAACCTAAATGGGACCCCATATGATCCTGCAGAGGACATACCAGCTCCTCTGGGGACGCCAGACTTTCGGGGCAAAGTCTTTGGCGTGGCCAGCCAGAGAAACCCTGACAGCACAACAAGGGCACATGAGGCAAAAGTGGACACAACATCTGGCCGCTTCACCCCAAAATTGGGCTCCCTAGAAATATCCACTGAATCTGGTGACTTTGACCAAAATCAACCAACAAGATTCACCCCAGTTGGCATCGGAGTTGACAATGAGGCAGACTTTCAACAATGGACCTTACCTGACTATTCCGGTCAGTTCACCCACAATATGAATCTGGCCCCAGCTGTCGCTCCCAACTTTCCCGGCGAACAGCTTCTTTTCTTCCGCTCACATTTGCCATCCTCTGGCGGGCGGTCCAATGGTATTCTAGACTGCCTGGTCCCCCAGGAATGGGTCCAACACTTCTACCAGGAATCCGCCCCTGCCCAAACGCAGGTGGCTCTGGTCAGGTATGTCAACCCTGATACTGGTAGGGTGTTATTTGAGGCTAAGCTGCATAAGCTAGGCTTCATGACCATAGCTAAGAGTGGTGATTCTCCAATAACTGTACCTCCAAATGGATACTTTAGGTTTGAATCTTGGGTGAACCCCTTTTATACATTAGCCCCCATGGGAACTGGGAATGGGCGTAGAAGGATTCAATAA

>JN699039|-/II.3|1978|HK|HK71

ATGAAGATGGCGTCGAATGACGCTGCTCCATCTAACGATGGTGCCGCCGGCCTCGTCCCAGAGATCAACAATGAGGCAATGGCGCTAGAGCCAGTGGCGGGTGCAGCGATAGCAGCACCCCTCACTGGCCAGCAAAACATAATTGATCCCTGGATTATGAATAATTTTGTGCAAGCACCTGGTGGTGAGTTTACAGTGTCACCTAGGAATTCCCCTGGTGAAGTGCTTCTTAATTTAGAATTAGGTCCAGAAATAAACCCCTATTTGGCTCACCTTGCTAGGATGTACAATGGTTATGCAGGTGGGTTTGAAGTGCAGGTAGTCCTGGCTGGAAACGCGTTTACAGCAGGAAAGGTGATCTTTGCAGCTATACCCCCCAATTTTCCAATTGATAATCTGAGCGCAGCACAAATTACAATGTGCCCGCATGTGATTGTGGATGTCAGGCAGCTGGAACCAATTAATCTTCCGATGCCTGATGTCCGCAACAATTTCTTTCATTATAATCAAGGGTCTGATTCGAGGTTACGCTTAATTGCAATGCTGTATACACCTCTTAGGGCAAACAATTCCGGAGATGATGTTTTTACTGTGTCCTGTAGAGTATTAACTAGGCCTAGCCCTGATTTCTCATTCAATTTTCTTGTCCCACCCACTGTGGAATCAAAGACAAAACCCTTCACCCTCCCCATTCTGACTATCTCTGAAATGTCTAATTCCAGGTTTCCAGTGCCAATTGACTCTCTACACACCAGCCCGACTGAGAACATTGTTGTCCAGTGCCAAAATGGGCGCGTCACTCTTGACGGTGAGTTAATGGGTACCACCCAACTCTTGCCGAGTCAGATATGTGCTTTCAGGGGCACGCTCACCAGATCAACAAGCAGGGCCAGTGATCAAGCCGACACAGCAACCCCTAGGTTATTCAATTATTATTGGCACATACAATTGGACAATCTAAATGGAACCCCCTACGACCCTGCAGAGGACATACCAGCCCCTCTGGGAACACCAGACTTCCGGGGCAAGGTCTTTGGCGTAGCCAGCCAGAGAAACCCTGACAGCACAACAAGAGCACATGAAGCAAAAGTGGACACAACATCTGGTCGCTTCACCCCGAAATTGGGTTCCCTAGAAATATCCACTGAATCCGATGACTTTGACCCAAACCAACCAACAAGATTCACCCCAGTTGGCATTGGGGTTGACAATGAGGCAGATTTTCAGCAATGGTCCTTACCTGACTATTCCGGTCAGTTCACTCACAACATGAACTTAGCCCCAGCTGTCGCCCCCAATTTCCCTGGTGAGCAGCTTCTTTTCTTCCGCTCACAGTTGCCATCTTCTGGTGGGCGGTCTAACGGGATTCTAGACTGCCTGGTCCCCCAGGAATGGGTTCAACACTTCTACCAGGAATCAGCCCCTGCCCAAACACAGGTGGCCCTGGTTAGGTATGTCAACCCTGACACTGGTAGAGTGCTATTTGAGGCCAAGCTACATAAATTAGGTTTCATGACTATAGCTAAGAATGGTGACTCTCCAATAACCGTCCCTCCAAATGGGTACTTTAGGTTTGAATCTTGGGTGAACCCCTTTTATACACTTGCCCCCATGGGAACTGGAAATGGGCGCAGAAGGATTCAATAA

>JN699040|-/II.3|1977|HK|HK54

ATGAAGATGGCGTCGAATGACGCTGCTCCATCTAACGATGGTGCCGCCGGCCTCGTCCCAGAGATCAACAATGAGGCAATGGCGCTAGAGCCAGTGGTGGGTGCAGCGATAGCAGCACCCCTCACTGGCCAGCAAAACATAATTGATCCCTGGATTATGAATAATTTTGTGCAAGCACCTGGTGGTGAGTTTACAGTGTCACCTAGGAATTCCCCTGGTGAAGTGCTTCTTAATTTAGAATTAGGTCCAGAAATAAACCCCTATTTGGCTCACCTTGCTAGGATGTACAATGGTTATGCAGGTGGGTTTGAAGTGCAGGTAGTCCTGGCTGGAAATGCGTTTACAGCAGGAAAGGTGATCTTTGCAGCTATACCCCCCAATTTTCCAATTGATAATCTGAGCGCAGCACAAATTACAATGTGCCCGCATGTGATTGTGGATGTCAGGCAGCTGGAACCAATTAATCTTCCGATGCCTGATGTCCGCAACAATTTCTTTCATTATAATCAAGGGTCTGATTCGAGGTTACGCTTAATTGCAATGCTGTATACACCTCTTAGGGCAAACAATTCCGGAGATGATGTTTTTACTGTGTCCTGTAGAGTATTAACTAGGCCTAGCCCTGATTTCTCATTCAATTTTCTTGTCCCACCCACTGTGGAATCAAAGATAAAACCCTTCACCCTCCCCATTCTGACTATCTCTGAAATGTCTAATTCCAGGTTTCCAGTGCCAATTGACTCTCTGCACACCAGCCCGACTGAGAACATTGTTGTCCAGTGCCAAAATGGGCGCGTCACTCTTGACGGTGAGTTAATGGGTACCACCCAACTCTTGCCGAGTCAGATATGTGCTTTCAGGGGCACGCTCACCAGATCAACAAGCAGGGCCAGTGACCAAGCCGACACAGCAACCCCTAGGTTATTCAATTATTATTGGCACATACAATTGGACAATCTAAATGGAACCCCCTACGACCCTGCAGAGGACATACCAGCCCCTCTGGGAACACCAGACTTCCGGGGCAAGGTCTTTGGCGTAGCCAGCCAGAGAAACCCTGACAGCACAACAAGAGCACATGAAGCAAAAGTGGACACAACATCTGGTCGCTTCACCCCGAAATTGGGCTCCCTAGAAATATCCACTGAATCCGGTGACTTTGACCCAAACCAACCAACAAGATTCACCCCAGTTGGCATTGGGGTTGACAATGAGGCAGATTTTCAGCAATGGTCCTTACCTGACTATTCCGGTCAGTTCACTCACAACATGCACTTAGCCCCAGCTGTCGCCCCCAATTTTCCTGGTGAGCAGCTTCTTTTCTTCCGCTCACAGTTGCCATCTTCTGGTGGGCGGTCAAACGGGATTCTAGACTGCCTGGTCCCCCAGGAATGGGTTCAACACTTCTACCAGGAATCAGCCCCTGCCCAAACACAGGTGGCCCTGGTTAGGTATGTTAACCCTGACACTGGTAGAGTGCTATTTGAGGCCAAGCTACATAAATTAGGTTTCATGACTATAGCTAAGAATGGTGACTCTCCAATAACCGTCCCTCCAAATGGGTACTTTAGGTTTGAATCTTGGGTGAACCCCTTTTACACACTTGCCCCCATGGGAACTGGAAATGGGCGTAGAAGGATTCAATAA

>JN899244|-/II.3|2004|US|Glastonbury1164

ATGAAGATGGCGTCGAATGACGCCACTCCATCTAATGATGGTGCCGCCGGCCTCGTCCCAGAGATCAACAATGAGGCAATGGCGCTAGATCCAGTGGCGGGTGCAGCGATAGCAGCACCTCTCACTGGTCAGCAAAATATAATTGATCCCTGGATTATGAATAATTTTGTGCAAGCACCTGGTGGTGAGTTTACAGTATCCCCTAGGAATTCCCCTGGTGAAGTGCTTCTTAATTTGGAATTGGGCCCAGAAATAAATCCCTATTTGGCCCATCTTGCTAGAATGTATAATGGTTATGCAGGTGGATTTGAAGTGCAGGTAGTCCTAGCTGGAAATGCGTTTACAGCAGGAAAGATAATCTTTGCAGCTATACCCCCCAATTTTCCAACTGATAATCTAAGCGCAGCACAGATCACAATGTGCCCACATGTGATTGTGGATGTCAGACAGTTGGAACCAGTCAACCTCCCGATGCCTGACGTTCGCAACAACTTCTTCCATTACAATCAAGGGTCTGATTCGAGATTGCGCTTAATTGCAATGCTGTATACACCTCTTAGGGCAAATAATTCTGGTGATGATGTTTTTACTGTGTCTTGTAGAGTGCTGACTAGGCCTAGCCCTGACTTCTCATTTAATTTCCTTGTGCCACCTACTGTGGAGTCAAAGACAAAACCCTTCACTCTCCCTATTCTGACTATCTCTGAAATGTCCAATTCTAGGTTTCCAGTGCCGATTGATTCTCTGCACACCAGCCCAACTGAGAATATTGTTGTCCAGTGCCAAAATGGGCGCGTCACTCTTGATGGTGAGTTGATGGGCACCACCCAACTCTTACCCAGTCAAATCTGTGCTTTCAGGGGCGTGCTCACCAGGTCAACAAGCAGGGCCAGTGACCAGGCCGACACAGCAACCCCTAGGTTGTTTAATTATTATTGGCACATACAATTGGATAATCTAAATGGAACTCCCTATGATCCTGCAGAAGACATACCAGGCCCCCTAGGGACGCCAGATTTTCGGGGCAAAATCTTTGGCGTGGCCAGCCAGAGAAACCCTGACAGCACAACTAGAGCACATGAAGCAAAGGTGGACACAACAGCTGGTCGTTTCACCCCAAAACTAGGCTCATTAGAGATATCCACTGAATCTGGTGACTTTGACCAAAACCAACCAACAAGATTCACCCCAGTTGGCATTGGGGTTGACAATGAAGCGGACTTTCAACAATGGTCTTTACCCGACTATTCTGGTCAGTTCACCCACAACATGAACTTAGCCCCAGCTGTTGCTCCCAACTTCCCTGGTGAGCAGCTCCTCTTCTTCCGCTCACAGTTACCATCCTCTGGTGGGCGATCCAACGGGATTCTAGACTGCCTGGTCCCCCAAGAGTGGGTTCAGCACTTCTACCAAGAATCGGCCCCCGCTCAAACTCAAGTGGCCCTGGTTAGGTATGTCAACCCTGACACTGGTAGAGTATTATTTGAGGCCAAGCTGCACAAATTAGGTTTCATGACTATAGCTAAGAATGGTGATTCTCCAATAACTGTCCCTCCAAATGGATACTTTAGGTTTGAATCTTGGGTGAACCCATTTTATACACTTGCCCCCATGGGAACTGGGAATGGGCGTAGAAGGATTCAATAA

>JQ743333|-/II.3|1999|US|GII.3/1999

ATGAAGATGGCGTCGAATGACGCTGCTCCATCTAATGATGGTGCCGCCGGCCTCGTCCCAGAGATCAACAATGAGGCAATGGCGCTAGATCCAGTGGCGGGTGCAGCGATAGCAGCGCCCCTCACTGGCCAGCAAAATATAATTGATCCCTGGATTATGAATAATTTTGTGCAAGCACCTGGTGGTGAGTTTACAGTGTCACCTAGGAATTCCCCTGGTGAAGTGCTTCTTAATTTGGAATTAGGTCCAGAAATAAACCCCTATTTGGCTCATCTTGCTAGAATGTACAATGGTTATGCAGGTGGATTTGAAGTGCAAGTGGTCCTAGCTGGAAATGCGTTTACAGCAGGAAAGGTTATCTTTGCAGCTATACCCCCTAATTTCCCTATTGACAATCTGAGCGCGGCACAGATCACAATGTGCCCGCACGTGATTGTGGATGTCAGGCAGTTGGAACCAATCAATCTCCCAATGCCTGATGTCCGCAACACTTTCTTTCATTATAATCAAGATTCTGATTCAAGATTGCGTTTGATTGCAATGTTGTATACACCTCTTAGGGCAAATAATTCTGGAGATGATGTTTTCACTGTGTCTTGTAGGGTGTTAACTAGGCCCAGCCCTGATTTCTCATTCAATTTTCTTGTCCCACCTACTGTGGAATCAAAGACAAAGCTTTTTACCCTCCCCATTTTAACCATCTCTGAAATGTCCAATTCCAGGTTTCCAGTTCCAATTGACTCTTTGCACACCAGCCCAACTGAGAATATAGTAGTCCAGTGCCAAAATGGGCGCGTCACTCTTGACGGTGAGTTAATGGGCACCACCCAACTCCTACCAAGCCAAATATGTGCTTTCAGGGGCACACTCACTAGATCAACAAGCAGGGCCAGCGACCAAGCCGACACACCAACCCCCAGGCTATTCAACCATCGTTGGCACATACAATTGGACAATCTAAATGGAACTCCCTACGACCCTGCAGAGGACATACCAGCTCCTTTGGGCACACCAGACTTCCGGGGCAAGGTCTTTGGCGTGGCCAGCCAGAGAAACCCCGACAGCACAACAAGGGCACATGAAGCAAAAGTGGACACAACATCTGGCCGCTTCACCCCAAAATTAGGCTCCTTAGAAATAACCACTGAGTCTGATGACTTTGACACAAACCAGTCAACAAAATTCACCCCAGTTGGCATCGGGGTTGACAATGAGGCAGAATTTCAACAATGGTCCTTACCCAACTATTCTGGTCAGTTTACTCATAATATGAACTTAGCCCCAGCTGTCGCCCCCAATTTTCCTGGTGAACAGCTACTTTTCTTCCGATCACAGCTGCCATCCTCTGGTGGGTGGTCTAACGGGGTTCTAGACTGCCTGGTCCCCCAGGAATGGGTTCAACACTTTTACCAAGAATCAGCCCCCGCCCAAACGCAGGTGGCCCTGGTTAGGTATGTCAACCCTGACACTGGTAGAGTGTTATTTGAGGCTAAGCTACACAAATTGGGTTTTATGACTATAGCAAAGAATGGTGACTCCCCAATAACTGTCCCTCCAAATGGATACTTTAGATTTGAATCTTGGGTTAACCCCTTCTACACACTTGCCCCCATGGGAACTGGAAACGGGCGTAGAAGGATTCAATAA

>JX846924|II.g/II.3|1978|CN|HK71

ATGAAGATGGCGTCGAATGACGCTGCTCCATCTAACGATGGTGCCGCCGGCCTCGTCCCAGAGATCAACAATGAGGCAATGGCGCTAGAGCCAGTGGCGGGTGCAGCGATAGCAGCACCCCTCACTGGCCAGCAAAACATAATTGATCCCTGGATTATGAATAATTTTGTGCAAGCACCTGGTGGTGAGTTTACAGTGTCACCTAGGAATTCCCCTGGTGAAGTGCTTCTTAATTTAGAATTAGGTCCAGAAATAAACCCCTATTTGGCTCACCTTGCTAGGATGTACAATGGTTATGCAGGTGGGTTTGAAGTGCAGGTAGTCCTGGCTGGAAACGCGTTTACAGCAGGAAAGGTGATCTTTGCAGCTATACCCCCCAATTTTCCAATTGATAATCTGAGCGCAGCACAAATTACAATGTGCCCGCATGTGATTGTGGATGTCAGGCAGCTGGAACCAATTAATCTTCCGATGCCTGATGTCCGCAACAATTTCTTTCATTATAATCAAGGGTCTGATTCGAGGTTACGCTTAATTGCAATGCTGTATACACCTCTTAGGGCAAACAATTCCGGAGATGATGTTTTTACTGTGTCCTGTAGAGTATTAACTAGGCCTAGCCCTGATTTCTCATTCAATTTTCTTGTCCCACCCACTGTGGAATCAAAGACAAAACCCTTCACCCTCCCCATTCTGACTATCTCTGAAATGTCTAATTCCAGGTTTCCAGTGCCAATTGACTCTCTACACACCAGCCCGACTGAGAACATTGTTGTCCAGTGCCAAAATGGGCGCGTCACTCTTGACGGTGAGTTAATGGGTACCACCCAACTCTTGCCGAGTCAGATATGTGCTTTCAGGGGCACGCTCACCAGATCAACAAGCAGGGCCAGTGATCAAGCCGACACAGCAACCCCTAGGTTATTCAATTATTATTGGCACATACAATTGGACAATCTAAATGGAACCCCCTACGACCCTGCAGAGGACATACCAGCCCCTCTGGGAACACCAGACTTCCGGGGCAAGGTCTTTGGCGTAGCCAGCCAGAGAAACCCTGACAGCACAACAAGAGCACATGAAGCAAAAGTGGACACAACATCTGGTCGCTTCACCCCGAAATTGGGTTCCCTAGAAATATCCACTGAATCCGATGACTTTGACCCAAACCAACCAACAAGATTCACCCCAGTTGGCATTGGGGTTGACAATGAGGCAGATTTTCAGCAATGGTCCTTACCTGACTATTCCGGTCAGTTCACTCACAACATGAACTTAGCCCCAGCTGTCGCCCCCAATTTCCCTGGTGAGCAGCTTCTTTTCTTCCGCTCACAGTTGCCATCTTCTGGTGGGCGGTCTAACGGGATTCTAGACTGCCTGGTCCCCCAGGAATGGGTTCAACACTTCTACCAGGAATCAGCCCCTGCCCAAACACAGGTGGCCCTGGTTAGGTATGTCAACCCTGACACTGGTAGAGTGCTATTTGAGGCCAAGCTACATAAATTAGGTTTCATGACTATAGCTAAGAATGGTGACTCTCCAATAACCGTCCCTCCAAATGGGTACTTTAGGTTTGAATCTTGGGTGAACCCCTTTTATACACTTGCCCCCATGGGAACTGGAAATGGGCGCAGAAGGATTCAATAA

>JX984948|-/II.3|2010|CN|GZ2010-L63

ATGAAGATGGCGTCGAATGACGCCACTCCATCTAATGATGGTGCCGCCGGCCTCGTCCCAGAGATCAACAGTGAGGCAATGGCGCTAGAGCCAGTGGCGGGCGCAGCGATAGCAGCACCCCTCACTGGTCAGCAAAATATAATTGATCCCTGGATTATGAATAATTTTGTGCAAGCACCTGGTGGTGAGTTTACAGTATCCCCTAGAAATTCCCCTGGTGAAGTTCTTCTCAATTTGGAATTGGGTCCAGAAATAAATCCCTATTTGGCCCATCTTGCTAGAATGTATAATGGTTATGCAGGTGGATTTGAAGTGCAGGTGGTCCTAGCTGGAAATGCGTTTACAGCAGGAAAGATAATCTTTGCAGCTATCCCCCCTAATTTTCCAATTGATAATCTAAGTGCAGCACAGATCACAATGTGTCCACATGTGATTGTGGATGTCAGACAGTTGGAACCAGTCAACCTTCCGATGCCTGACGTTCGCAACAACTTCTTCCATTACAATCAAGGGTCTGATTCGAGATTGCGCCTAATTGCAATGCTATATACACCTCTTAGGGCAAATAATTCTGGGGATGATGTTTTCACTGTGTCTTGCAGAGTGCTAACTAGACCTAGTCCTGACTTCTCATTTAATTTCCTTGTGCCACCCACTGTGGAGTCAAAGACAAAACCCTTTTCCCTCCCTATTCTGACTATCTCTGAAATGTCCAATTCTAGGTTCCCAGTACCAATTGATTCTTTGCACACCAGCCCTACTGAGAACATTGTTGTCCAGTGTCAGAATGGACGCGTCACCCTTGATGGTGAGTTGATGGGCACCACCCAACTCTTACCTAGCCAAATCTGTGCTTTCAGGGGCATGCTCACCAGATCAACAAGCAGGGCCAGTGACCAGGCCGATACAGCAACTCCTAGATTGTTTAATTATTATTGGCATATACAATTGGATAATCTAAATGGAACTCCTTATGACCCTGCAGAAGACATACCAGGCCCCCTAGGGACACCAGATTTCCGGGGCAAAGTCTTCGGCGTGGCCAGCCAGAGAAATCCTGATGCCACGACTAGGGCACATGAAGCAAAGATAGACACAACATCTGGCCGCTTCACCCCAAAATTAGGCTCACTTGAGATTTCCACTGAGTCTGGAGATTTTGATCAAAACCAACCAACAAGATTCACCCCAGTTGGCATTGGGGTTGATCGTGAGGCAGACTTTCAACAATGGACTCTTCCCGACTATGCTGGTCAGTTCACACACAACATGAACTTAGCCCCAGCTGTTGCTCCAAACTTCCCTGGCGAGCAGCTCCTTTTCTTCCGCTCACAGTTGCCATCTTCTGGTGGGCGGTCCAACGGGATTCTAGACTGCCTGGTCCCCCAAGAATGGGTACAGCACTTCTACCAAGAATCAGCCCCCTCCCAAACTCAAGTGGCCCTGGTTAGGTATATCAACCCTGACACTGGTAGAGTGTTATTTGAGGCCAAACTGCACAAATTAGGTTTCATGACTATAGCCAAGAGTGGTGATTCTCCAATAACTGTCCCTCCAAATGGATATTTTAGGTTTGAATCTTGGGTGAACCCCTTTTATACACTTGCCCCCATGGGAACTGGGAACGGGCGTAGAAGGATTCAATAA

>KC464324|-/II.3|2001|AU|Ohio/477

ATGAAGATGGCGTCGAATGACGCCACTCCATCTAATGATGGTGCCGCCGGCCTCGTCCCAGAGATCAACAATGAGGCAATGGCGCTAGATCCAGTGGCGGGTGCAGCGATAGCAGCACCCCTCACTGGTCAGCAAAATATAATTGATCCCTGGATTATGAATAATTTTGTGCAAGCACCTGGTGGTGAGTTTACAGTATCCCCTAGGAATTCCCCTGGTGAAGTGCTTCTTAATTTGGAATTGGGCCCAGAAATAAATCCCTATTTGGCCCATCTTGCTAGAATGTATAATGGTTATGCAGGTGGATTTGAAGTGCAGGTAGTCCTAGCTGGAAATGCGTTTACAGCAGGAAAGATAATCTTTGCAGCTATACCCCCTAATTTTCCAATTGATAATCTAAGCGCAGCACAGATCACAATGTGCCCACATGTGATTGTGGATGTCAGACAGTTGGAACCGGTCAACCTCCCGATGCCTGACGTTCGCAACAACTTCTTTCATTACAATCAAGGGTCTGATTCGAGATTGCGCTTAATTGCAATGCTGTATACACCTCTTAGGGCAAATAATTCTGGGGATGATGTTTTTACTGTGTCTTGTAGAGTGCTGACTAGGCCTAGCCCTGACTTTTCATTTAATTTCCTTGTGCCACCTACTGTGGAGTCAAAGACAAAACCCTTTACCCTCCCTATTCTGACTATCTCTGAAATGTCCAATTCTAGGTTTCCAGTGCCGATTGATTCTCTGCACACCAGCCCAACTGAGAATATCGTTGTCCAGTGCCAAAATGGGCGCGTCACTCTTGATGGTGAGTTGATGGGCACCACCCAACTCTTACCTAGTCAAATCTGTGCTTTTAGGGGCGTGCTCACCAGATCAACAAGCAGGGCCAGTGATCAGGCCGACACAGCAACCCCTAGGTTGTTTAATTATTATTGGCACATACAATTGGATAATCTAAATGGGACTCCTTATGATCCTGCAGAAGACATACCAGGCCCCCTAGGGACACCAGATTTCCGGGGCAAAGTCTTTGGCGTGGCCAGCCAGAGAAATCCCGACAGCACAACTAGAGCACATGAAGCAAAGGTGGACACAACAGCTGGTCGTTTCACCCCAAAACTAGGCTCATTAGAGATATCCACTGAATCTGATGACTTTGATCAAAACCAACCAACAAGATTCACCCCAGTTGGCATTGGGGTTGACCGTGAGGCAGACTTTCAACAATGGTCTTTACCCGACTATTCTGGTCAGTTCACCCACAACATGAACTTAGCCCCAGCTGTTGCTCCCAACTTCCCTGGTGAGCAGCTCCTTTTCTTTCGCTCACAGTTACCATCCTCTGGTGGGCGATCCAACGGGGTTCTAGACTGCCTGGTCCCCCAAGAATGGGTTCAGCACTTCTACCAAGAATCGGCCCCCGCCCAAACTCAAGTGGCCCTGGTTAGGTATGTCAACCCTGACACTGGTAGAGTATTATTTGAGGCCAAGCTGCACAAACTAGGTTTCATGACTATAGCTAAGAATGGTGACTCTCCAATAACTGTCCCTCCAAATGGATATTTTAGGTTTGAATCTTGGGTGAACCCCTTTTATACACTTGCCCCCATGGGAACTGGGAATGGGCGTAGAAGGGTTCAATAA

>KC464325|-/II.3|2002|AU|02-13/424cons

ATGAAGATGGCGTCGAATGACGCCACTCCATCTAATGATGGTGCCGCCGGCCTCGTCCCAGAGATCAACAATGAGGCAATGGCGCTAGACCCAGTGGCGGGTGCAGCGATAGCAGCACCCCTCACTGGTCAGCAAAATATAATTGATCCCTGGATTATGAATAATTTTGTGCAAGCACCTGGTGGTGAGTTTACAGTATCCCCTAGGAATTCCCCTGGTGAAGTGCTTCTCAATTTGGAATTGGGCCCAGAAATAAATCCCTATTTGGCCCATCTTGCTAGAATGTATAATGGTTATGCAGGTGGATTTGAAGTGCAGGTAGTCCTAGCTGGAAATGCGTTTACAGCAGGAAAGATAATCTTTGCAGCTATACCCCCTAATTTTCCAATTGATAATCTAAGCGCAGCACAGATCACAATGTGCCCACATGTAATTGTGGATGTCAGACAGTTGGAACCGGTCATCCTCCCGATGCCTGACGTTCGCAACAACTTCTTTCATTACAATCAAGGGTCTGATTCGAGATTGCGCTTAATTGCAATGCTGTATACACCTCTTAGGGCAAATAATTCTGGGGATGATGTTTTTACTGTGTCTTGTAGAGTGCTGACTAGGCCTAGCCCTGATTTTTCATTTAATTTCCTTGTGCCACCTACTGTGGAGTCAAAGACAAAACCCTTCACTCTCCCTATTCTGACTATCTCTGAAATGTCCAATTCTAGGTTTCCAGTGCCGATTGATTCTCTGCACACCAGCCCAACTGAGAATATTGTTGTCCAGTGCCAAAATGGGCGCGTCACTCTTGATGGTGAGTTGATGGGCACCACCCAACTCTTACCTAGTCAAATCTGTGCTTTCAGGGGCGTGCTCACCAGATCAACGAGCAGGGCCAGTGATCAGGCCGACACAGTAACCCCTAGGTTGTTTAATTATTATTGGCACATACAATTGGATAATCTAAATGGGACTCCTTATGATCCTGCAGAAGACATACCAGGCCCCCTAGGGACACCAGATTTCCGGGGCAAAGTCTTTGGCGTGGCCAGCCAGAGAAATCCCGACAGCACAACTAGAGCACATGAAGCAAAAGTGGACACAACAGCTGGTCGTTTCACCCCAAAACTAGGCTCATTAGAGATATCCACCGAATCTGATGACTTTGATCAAAACCAACCAACAAGATTCACCCCAGTTGGCATTGGGGTTGACCGTGAGGCAGACTTTCAACAATGGTCTTTACCCGATTATTCTGGTCAGTTCACCCACAACATGAACTTAGCCCCAGCTGTTGCTCCCAACTTCCCTGGTGAGCAGCTCCTTTTCTTCCGCTCACAGTTACCATCCTCTGGTGGGCGATCCAACGGGATTCTAGACTGCCTGGTCCCCCAAGAATGGGTTCAGCACTTCTACCAAGAATCGGCACCCGCCCAAACTCAAGTGGCTCTGGTTAGGTATGTCAACCCTGACACTGGTAGAGTATTATTTGAGGCCAAGCTGCACAAATTAGGTTTCATGACTATAGCTAAGAATGGTGACTCTCCAATAACTGTCCCTCCAAATGGATACTTTAGGTTTGAATCTTGGGTGAACCCATTTTATACACTTGCCCCCATGGGAACTGGGAATGGGCGTAGAAGGATTCAATAA

>KC464326|-/II.3|2006|AU|41/89

ATGAAGATGGCGTCGAATGACGCCGCTCCATCTAATGATGGTGCCGCCGGCCTCGTCCCAGAGATCAACAATGAGGCAATGGCGCTAGAGCCAGTGGCGGGTGCAGCGATAGCAGCACCCCTCACTGGTCAGCAAAACATAATTGATCCCTGGATTATGAATAATTTTGTGCAAGCACCTGGTGGTGAGTTTACAGTGTCCCCTAGAAATTCCCCTGGTGAAGTTCTTCTTAATTTGGAACTGGGCCCAGAAATAAATCCCTATTTGGCCCATCTTGCTAGAATGTATAATGGTTATGCAGGTGGATTTGAAGTGCAGGTGGTCCTAGCTGGAAATGCGTTTACAGCAGGAAAGATAATCTTTGCAGCTATTCCCCCCAATTTTCCAATTGACAATCTAAGTGCAGCACAGATCACAATGTGTCCACATGTGATTGTGGATGTCAGACAGTTGGAACCAGTCAACCTCCCGATGCCTGACGTTCGCAACAACTTCTTCCATTACAATCAAGGGTCTGATTCGAGATTGCGCCTAATTGCAATGCTATACACACCTCTTAGGGCAAATAATTCTGGGGATGATGTTTTTACTGTGTCTTGCAGAGTGCTAACTAGACCTAGTCCTGACTTCTCATTTAATTTCCTTGTGCCACCTACTGTGGAGTCAAAGACAAAACCCTTTTCCCTCCCTATTCTGACTATCTCTGAAATGTCCAATTCTAGGTTCCCAGTACCAATTGATTCTCTGCACACCAGCCCCACTGAGAATATTGTTGTCCAGTGCCAGAATGGGCGAGTCACCCTTGATGGTGAGTTGATGGGCACCACCCAGCTCTTACCTAGCCAAATCTGTGCTTTCAGGGGCGTGCTCACCAGGTCAACAAGCAGGGCCAGTGACCAGGCCGATACAGCAACCCCTAGATTGTTTAATTATTATTGGCATATACAATTGGATAATCTAAATGGAACTCCTTATGATCCTGCAGAAGACATACCAGGCCCCCTAGGGACACCAGATTTCCGGGGCAAAGTCTTTGGCGTGGCCAGCCAGAGAAATCCTGATACCACGACTAGGGCACATGAAGCAAAGGTAGACACAACATCCGGCCGTTTCACCCCAAAACTAGGCTCATTAGAGATATCCACTGAATCTGATGATTTTGATCAAAACAAACCAACAAGATTCACCCCAGTTGGCATTGGGGTTGACCATGAGGCAGACTTTCAACAATGGACTCTACCCGACTATGCTGGCCAGTTCACCCACAACATGAACTTAGCCCCAGCTGTTGTTCCCAACTTTCCTGGTGAGCAGCTCCTTTTCTTCCGCTCACAGTTGCCATCCTCTGGTGGGCGATCCAACGGGATTCTAGACTGCCTGGTCCCCCAAGAATGGGTACAGCACTTCTACCAAGAATCAGCCCCCTCCCAATCTCAAGTGGCCCTGGTTAGGTATATCAACCCTGACACTGGTAGAGTGTTATTTGAGGCCAAGCTGCACAAATTAGGTTTCATGACTATAGCCAAGAATGGTGATTCTCCAATAACTGTCCCTCCAAATGGATACTTTAGGTTTGAATCTTGGGTGAACCCCTTTTATACACTTGCCCCCATGGGAACTGGAAATGGGCGTAGAAGGATTCAATAA

>KC464327|-/II.3|2007|AU|84/46

ATGAAGATGGCGTCGAATGACGCCGCTCCATCTAATGATGGTGCCGCCGGCCTCGTCCCAGAGATCAACAATGAGGCAATGGCGCTAGAGCCAGTGGCGGGTGCAGCGATAGCAGCACCCCTCACTGGTCAGCAAAATATAATTGATCCCTGGATTATGAATAATTTTGTGCAAGCACCTGGTGGTGAGTTTACAGTATCCCCTAGAAATTCCCCTGGTGAAGTTCTTCTTAATTTGGAACTGGGCCCAGAAATAAATCCCTATTTGGCCCATCTTGCTAGAATGTACAACGGTTATGCAGGTGGATTTGAAGTGCAGGTGGTCCTAGCTGGAAATGCGTTTACAGCAGGAAAGATAATCTTTGCAGCTATTCCCCCCAATTTTCCAATTGACAATCTAAGTGCAGCACAGATCACAATGTGCCCACATGTGATTGTGGATGTCAGACAGTTGGAACCAGTCAACCTCCCGATGCCTGACGTTCGCAACAACTTCTTTCATTACAATCAAGGGTCTGATTCGAGATTGCGCCTAATTGCAATGTTATACACACCTCTTAGGGCAAATAATTCTGGGGATGATGTTTTTACTGTGTCTTGCAGAGTGCTAACTAGACCTAGTCCTGACTTCTCATTTAATTTCCTTGTGCCACCTACTGTGGAGTCAAAGACAAAACCCTTTTCCCTCCCTATTTTGACTATCTCTGAAATGTCCAATTCTAGGTTCCCAGTACCAATTGATTCTCTGCACACCAGTCCTACTGAGAATATTGTTGTCCAGTGCCAGAATGGGCGTGTCACCCTTGATGGTGAGTTGATGGGCACCACCCAACTCTTACCTAGCCAAATCTGTGCTTTCAGGGGCGTTCTCACCAGATCAACAAGCAGGGCCAGTGACCAGGCCGATACAGCAACCCCTAGATTGTTTAATTATTATTGGCACATACAATTGGATAATCTAAATGGAACTCCTTATGATCCTGCAGAAGACATACCAGGTCCCCTAGGGACACCAGATTTTCGGGGCAAAGTCTTTGGTGTGGCCAGCCAGAGAAATCCTGATACCTCGACTAGGGCACATGAAGCAAAGATAGACACAACATCTGGCCGTTTCACCCCAAAACTAGGCTCATTAGAGATATCCACTGAATCTGATGATTTTGATCAAAACAAACCAACAAGATTCACCCCAGTTGGCATTGGGGTTGACCATGAGACAGACTTTCAACAATGGACTCTACCCGACTACGCTGGCCAGCTCACCCACAACATGAACTTAGCCCCAGCTGTTGCTCCCAACTTCCCTGGTGAGCAGCTCCTTTTCTTCCGCTCACAGTTGCCATCTTCTGGTGGGCGATCCAACGGGATTCTAGACTGCCTGGTCCCCCAAGAATGGGTGCAGCACTTCTACCAGGAATCAGCCCCCTCCCAATCTCAAGTGGCCCTGGTTAGGTATATCAACCCTGACACTGGTAGAGTGTTATTTGAGGCCAAGCTGCACAAATTAGGTTTCATGACTATAGCCAAGAATGGTGACTCTCCAATAACTGTCCCTCCAAATGGATACTTTAGGTTTGAATCTTGGGTGAACCCCTTTTACACACTTGCCCCCATGGGAACTGGGAACGGGCGTAGAAGGATTCAATAA

>KC464328|-/II.3|2008|AU|693/425

ATGAAGATGGCGTCGAATGACGCCGCTCCATCTAATGATGGTGCCGCCGGCCTCGTCCCAGAGATCAACAATGAGGCAATGGCGCTAGAGCCAGTGGCGGGTGCAGCGATAGCAGCACCCCTCACCGGTCAGCAAAATATAATTGATCCCTGGATTATGAATAATTTTGTGCAAGCACCTGGTGGTGAGTTTACAGTATCCCCTAGAAATTCCCCTGGTGAAGTCCTTCTCAATTTGGAATTGGGCCCAGAAATAAATCCCTATTTGGCCCATCTTGCTAGAATGTATAATGGTTATGCAGGTGGATTTGAAGTGCAGGTGGTCCTAGCTGGAAATGCGTTTACAGCAGGAAAGATAATCTTTGCAGCTATTCCCCCCAATTTTCCAATTGACAATCTAAGTGCAGCACAGATCACAATGTGTCCACATGTGATTGTGGATGTCAGACAGCTGGAACCAGTCAACCTCCCGATGCCTGACGTTCGCAACAACTTCTTTCATTACAATCAAGGGTCTGATTCGAGATTGCGCCTAATTGCAATGCTATATACACCTCTTAGGGCAAATAATTCTGGGGATGATGTTTTTACTGTGTCTTGCAGAGTGCTAACTAGACCTAGTCCTGACTTCTCATTTAATTTCCTTGTGCCACCCACTGTAGAGTCAAAGACAAAACCCTTTTCCCTACCTATTTTGACTATCTCTGAAATGTCCAACTCTAGGTTCCCAGTACCAATTGATTCTCTGCACACTAGCCCTACTGAGAATATTGTTGTCCAGTGTCAGAATGGGCGCGTCACCCTTGATGGTGAGTTGATGGGCACTACCCAACTCTTACCTAGCCAAATTTGTGCTTTCAGGGGCGTGCTCACCAGATCAACAAGCAGGGCCAGTGACCAGGCCGATACAGCAACCCCTAGATTGTTTAATTATTATTGGCATATACAATTGGATAATCTAAATGAAACTCCTTATGACCCTGCAGAAGGTATACCAGGCCCCCTAGGGACACCAGATTTTCGGGGCAAAGTCTTCGGCGTGGCCAGCCAGAGAAATCCTGATACTACAACTAGAGCGCATGAAGCAAAGGTAGACACAACATCTGGCCGTTTCACCCCAAAATTAGGCTCATTAGAAATTTCCACTGAATCTGATGATTTTGATCAAAATAAACCAACAAGATTCACCCCAGTTGGCATTGGGGTTGACCATGAGGCAGACTTTCAACAATGGACTCTTCCCGACTATGCTGGCCAGTTCACCCACAACATGAACTTAGCCCCAGCTGTTGCTCCCAACTTCCCTGGTGAGCAACTCCTTTTCTTCCGCTCACAGTTGCCATCCTCTGGTGGGCGATCCAACGGGATTCTAGACTGCCTGGTCCCCCAAGAATGGGTACAGCACTTCTACCAAGAATCAGCCCCCTCCCAATCTCAAGTGGCCCTGGTTAGGTATATCAACCCTGACACTGGTAGAGTGTTATTTGAGGCCAAGCTGCATAAATTAGGTTTCATAACTATAGCCAAGAATGGTGACTCTCCAATAACTGTCCCTCCAAATGGATACTTTAGGTTTGAATCTTGGGTGAACCCCTTTTATACACTTGCCCCCATGGGAACAGGGAATGGGCGTAGAAGGATTCAATAA

>KC464329|-/II.3|2010|AU|537/547

ATGAAGATGGCGTCGAATGACGCCGCCCCATCTAATGATGGTGCCGCCGGCCTCGTCCCAGAGATCAACAATGAGGCAATGGCGCTAGAGCCAGTGGCAGGTGCAGCGATAGCAGCACCCCTCACCGGTCAGCAAAATATAATTGATCCCTGGATTATGAATAATTTTGTGCAAGCACCTGGTGGTGAGTTTACAGTGTCCCCTAGAAATTCCCCTGGTGAAGTTCTTCTTAATTTGGAATTGGGCCCAGAAATAAATCCCTATTTGGCCCATCTTGCTAGAATGTATAATGGTTATGCAGGTGGATTTGAAGTGCAGGTGGTCCTAGCTGGAAATGCGTTTACAGCAGGAAAGATAATCTTTGCAGCTATTCCCCCCAATTTTCCAATTGACAATCTAAGTGCAGCACAGATCACAATGTGTCCACATGTGATTGTGGATGTCAGACAGCTGGAACCAGTCAACCTCCCGATGCCTGACGTTCGCAACAATTTCTTTCACTACAATCAAGGGTCTGATTCGAGATTGCGCCTAATTGCAATGTTATATACACCTCTTAGGGCAAATAATTCTGGGGATGATGTTTTTACTGTGTCTTGCAGAGTGCTAACTAGACCTAGTCCTGACTTCTCATTTAATTTCCTTGTGCCACCCACTGTGGAGTCAAAGACAAAACCCTTTTCCCTACCTATTTTGACTATCTCTGAAATGTCCAATTCTAGGTTCCCAGTACCAATTGATTCTCTGCACACTAGCCCTACTGAGAATATTGTTGTCCAGTGTCAGAATGGGCGCGTCACCCTTGATGGTGAGTTGATGGGCACTACCCAACTCTTACCTAGCCAAATTTGTGCTTTCAGGGGCGTGCTCACCAGATCAACAAGCAGGGCCAGTGACCAGGCCGATACAGCAACCCCTAGATTGTTTAATTATTATTGGCATATACAATTGGATAATCTAAATGGAACTCCTTATGACCCTGCAGAAGATATACCAGGCCCCCTAGGGACACCAGATTTTCGAGGCAAAGTTTTCGGCGTGGCCAGCCAGAGAAATCCTGATACTACAACTAGAGCGCATGAAGCAAAGGTAGACACAACATCTGGCCGCTTCACCCCAAAATTAGGCTCATTAGAAATTTCCACTGAATCTGATGATTTTGATCAAAATAAACCAACAAGATTCACCCCAGTTGGCATTGGGGTTGACCATGAGGCAGACTTTCAACAATGGACTCTTCCCGACTATGCTGGCCAGTTCACCCACAACATGAACTTAGCCCCAGCTGTTGCTCCCAACTTCCCTGGTGAGCAACTCCTCTTCTTCCGCTCACAGTTGCCATCTTCTGGTGGGCGAACCAACGGGATTCTAGACTGCCTGGTCCCCCAAGAATGGGTGCAGCACTTCTACCAAGAATCAGCCCCCTCCCAATCTCAAGTGGCCCTGGTTAGGTACATCAACCCTGACACAGGTAGAGTGTTATTTGAGGCCAAGCTGCATAAATTAGGTTTCATAACTATAGCCAAGAATGGTGACTCTCCAATAACTGTCCCCCCAAATGGATACTTTAGGTTTGAATCTTGGGTGAACCCCTTTTATACACTTGCCCCCATGGGAACAGGGAATGGGCGTAGAAGGATTCAATAA

>KC464495|II.12/II.3|2010|TW|CGMH36

ATGAAGATGGCGTCGAATGACGCCACTCCATCTAATGATGGTGCCGCCGGCCTCGTCCCAGAGATCAACAATGAGGCAATGGCGCTAGATCCAGTGGCGGGCGCAGCGATAGCAGCACCCCTCACTGGCCAGCAAAATATAATTGATCCCTGGATTATGAATAATTTTGTGCAAGCACCTGGTGGTGAGTTTACAGTGTCTCCTAGGAATTCCCCTGGTGAAGTGCTCCTCAATTTGGAACTGGGCCCAGAAATAAACCCCTATCTGGCCCATCTTGCTAGAATGTATAATGGTTATGCAGGTGGGTTTGAAGTGCAGGTAGTCCTAGCTGGAAATGCGTTTACAGCAGGAAAGATAATCTTTGCAGCTATACCCCCTAACTTCCCAATTGACAATCTAAGCGCAGCACAGATCACAATGTGCCCACATGTGATTGTGGATGTCAGACAGTTGGAACCGGTCAACCTTCCGATGCCTGACGTTCGCAATAACTTCTTCCACTACAACCAAGGGTCTGATTCGAGATTGCGCTTAGTTGCAATGTTGTATACACCTCTTAGGGCAAATAATTCTGGGGATGATGTTTTCACTGTGTCTTGTAGAGTGCTAACTAGGCCTAGCCCTGACTTTTCATTTAACTTCCTTGTGCCACCCACTGTGGAGTCAAAGACAAAACCCTTCACCCTCCCTATTCTGACTATCTCTGAAATGTCTAATTCTAGGTTTCCAGTGCCGATTGATTCTCTGCACACCAGCCCAACTGAGAATATTGTTGTCCAGTGCCAAAATGGGCGCGTCACTCTTGATGGTGAGTTGATGGGCACCACCCAACTCTTACCTAGTCAAATCTGTGCTTTTAGGGGCGTGCTCACCAGATCAACAAGCAGGGCTAGTGACCAGGCCGACACAGCAACCCCTAGATTGTTTAATTATTATTGGCACATACAATTGGATAATCTAAATGGGACTCCTTATGATCCTGCAGAAGACATACCAGGCCCCCTGGGGACACCAGATTTCCGGGGCAAAGTCTTTGGCGTGGCCAGCCAAAGAAACCCCGACAGTACAACTAGAGCACATGAAGCGAAGGTGGACACAACAGCTGGTCGCTTCACCCCAAAACTAGGCTCATTAGAGATATCCACTGAATCTGGTGACTTTGATCAAAACCAACCAACAAGATTCACCCCAGTTGGCATTGGGGTTGACCGCGAGGCAGACTTCCAACAATGGTCCTTACCCGACTATTCTGGTCAGTTCACTCACAACATGAACTTAGCCCCAGCTGTTGCTCCCAACTTCCCTGGTGAGCAGCTCCTTTTCTTCCGCTCACAGTTACCATCTTCTGGCGGGCGATCCAATGGGATTCTAGACTGCCTGGTCCCCCAAGAATGGGTTCAGCACTTCTACCAAGAATCGGCCCCCGCCCAAACCCAGGTGGCCCTGGTTAGATATGTCAACCCTGACACTGGTAGAGTGTTGTTTGAGGCCAAGCTGCACAAATTAGGTTTCATGACTATAGCTAAGAATGGTGACTCTCCAATAACTGTTCCCCCAAATGGATACTTTAGGTTTGAATCTTGGGTGAACCCATTTTACACACTTGCCCCCATGGGAACTGGGAATGGGCGTAGAAGGATTCAATAA

>KC597140|II.4/II.3|2011|US|NIHIC8.1

ATGAAGATGGCGTCGAATGACGCCGCTCCATCTAACGATGGTGCCGCCGGCCTCGTCCCAGAGATCAACAGTGAGGCAATGGCGCTAGAACCAGTGGCGGGTGCAGCGATAGCAGCACCCCTTACTGGTCAGCAAAATATAATTGATCCCTGGATTATGAATAATTTTGTGCAAGCACCTGGTGGTGAGTTTACAATATCCCCTAGGAATGGCCCTGGTGAAGTGCTTCTTAATTTGGAATTAGGCCCAGAAATAAACCCCTATTTGGCCCATCTTGCTAGAATGTATAATGGTTATGCAGGTGGATTTGAGGTGCAGGTAGTCCTAGCTGGGAATGCGTTCACAGCAGGAAAGATAATCTTTGCAGCCATACCCCCCAACTTTCCAATTGACAATCTGAGCGCAGCACAGGTCACAATGTGCCCGCATGTAATTGTGGATGTCAGGCAACTGGAACCGGTCAACCTCCCGATGCCTGATGTTCGCAACTCCTTCTTCCATTATAATCAAGGGACTGATTCGAGATTGCGCTTAATTGCTATGCTTTACACACCTCTTAGGGCAAATAATTCTGGTGATGATGTTTTCACTGTGTCTTGCAGGGTGCTGACCAGGCCTAGCCCTGACTTTTCATTTAATTTTCTTGTGCCACCTACTGTGGAATCAAAGACAAAACCCTTTACTCTTCCTATTCTGACTATCTCTGAAATGTCTAATTCTAGGTTTCCAGTGCCGATTGATTCTCTACATACCAGCCCAACTGATAACATTGTTGTCCAGTGCCAAAACGGGCGTGTCACTCTTGATGGTGAGCTGATGGGCACCACCCAACTCTTACCGAGTCAAATCTGCGCCTTCAGGGGCGTGCTCACTCAACGAACAAGCAGGGCCAGTGATCAGGCCGACACAGCAACCCCCAGGGCATTCGATCACCGTTGGCACATACAATTGGATAATCTAAATGGAACCCATTATGACCCTTCAGAAGACATACCAGGCCCTCTAGGGATACCAGATTTCCGGGGTAGAGTCTATGGCGTGGCCAGCCAGAGAAACTCTGATGGATCAACCAGAGCACATGAAGCAAAGATAGATACAACATCTAGTCGCTTCACCCCAAAATTAGGCTCATTAGAAATATCCACTGAATCTGATGATTTTGATGAAAACAAACCAACCAGATTCACCCCAGTTGGCATTGGGGTTGACAATGAGAGAGAATTTCAACAATGGTCCTTACCCGACTATTCTGGTCAATTCACCCACAACATGCACCTGGCCCCAGCTGTTGCTCCTAACTTCCCTGGTGAGCAGCTCCTTTTCTTCCGCTCGCATTTACCATCTTCTGGTGGGTGGGTTGACGGGACTCTAGACTGTCTGCTCCCCCAAGAATGGATACAACACTTCTATCAAGAATCGGCTCCCTCCCAAACACAGGTGGCCCTGGTCAGGTATGTCAACCCTGACACTGGTAGAGTGCTATTTGAGGCCAAACTGCACAAATCAGGTTTCATGACTATAGCTAGGAGTGGTGATTCTCCAATAACTGTCCCTCCAAATGGGTATTTCAGATTTGAATCTTGGGTGAACCCCTTTTACACACTTGCCCCCATGGGAACTGGGAATGGGCGTAGAAGAATTCAATAA

>KC597144|II.g/II.3|1977|CN|HK46

ATGAAGATGGCGTCGAATGACGCTGCTCCATCTAACGATGGTGCCGCCGGCCTCGTCCCAGAGATCAACAATGAGGCAATGGCGCTAGAGCCAGTGGTGGGTGCAGCGATAGCAGCACCCCTCACTGGCCAGCAAAACATAATTGATCCCTGGATTATGAATAATTTTGTGCAAGCACCTGGTGGTGAGTTTACAGTGTCACCTAGGAATTCCCCTGGTGAAGTGCTTCTTAATTTAGAATTAGGTCCAGAAATAAACCCCTATTTGGCTCACCTTGCTAGGATGTACAATGGTTATGCAGGTGGGTTTGAAGTGCAGGTAGTCCTGGCTGGAAATGCGTTTACAGCAGGAAAGGTGATCTTTGCAGCTATACCCCCCAATTTTCCAATTGATAATCTGAGCGCAGCACAAATTACAATGTGCCCGCATGTGATTGTGGATGTCAGGCAGCTGGAACCAATTAATCTTCCGATGCCTGATGTCCGCAACAATTTCTTTCATTATAATCAAGGGTCTGATTCGAGGTTACGCTTAATTGCAATGCTGTATACACCTCTTAGGGCAAACAATTCCGGAGATGATGTTTTTACTGTGTCCTGTAGAGTATTAACTAGGCCTAGCCCTGATTTCTCATTCAATTTTCTTGTCCCACCCACTGTGGAATCAAAGATAAAACCCTTCACCCTCCCCATTCTGACTATCTCTGAAATGTCTAATTCCAGGTTTCCAGTGCCAATTGACTCTCTGCACACCAGCCCGACTGAGAACATTGTTGTCCAGTGCCAAAATGGGCGCGTCACTCTTGACGGTGAGTTAATGGGTACCACCCAACTCTTGCCGAGTCAGATATGTGCTTTCAGGGGCACGCTCACCAGATCAACAAGCAGGGCCAGTGACCAAGCCGACACAGCAACCCCTAGGTTATTCAATTATTATTGGCACATACAATTGGACAATCTAAATGGAACCCCCTACGACCCTGCAGAGGACATACCAGCCCCTCTGGGAACACCAGACTTCCGGGGCAAGGTCTTTGGCGTAGCCAGCCAGAGAAACCCTGACAGCACAACAAGAGCACATGAAGCAAAAGTGGACACAACATCTGGTCGCTTCACCCCGAAATTGGGCTCCCTAGAAATATCCACTGAATCCGGTGACTTTGACCCAAACCAACCAACAAGATTCACCCCAGTTGGCATTGGGGTTGACAATGAGGCAGATTTTCAGCAATGGTCCTTACCTGACTATTCCGGTCAGTTCACTCACAACATGCACTTAGCCCCAGCTGTCGCCCCCAATTTTCCTGGTGAGCAGCTTCTTTTCTTCCGCTCACAGTTGCCATCTTCTGGTGGGCGGTCAAACGGGATTCTAGACTGCCTGGTCCCCCAGGAATGGGTTCAACACTTCTACCAGGAATCAGCCCCTGCCCAAACACAGGTGGCCCTGGTTAGGTATGTTAACCCTGACACTGGTAGAGTGCTATTTGAGGCCAAGCTACATAAATTAGGTTTCATGACTATAGCTAAGAATGGTGACTCTCCAATAACCGTCCCTCCAAATGGGTACTTTAGGTTTGAATCTTGGGTGAACCCCTTTTACACACTTGCCCCCATGGGAACTGGAAATGGGCGTAGAAGGATTCAATAA

>KF006265|-/II.3|2002|US|TCH-104

ATGAAGATGGCGTCGAATGACGCCACTCCATCTAATGATGGTGCCGCCGGCCTCGTCCCAGAGATCAACAATGAGGCAATGGCGCTAGATCCAGTGGCGGGTGCAGCGATAGCAGCGCCCCTCACTGGTCAGCAAAATATAATTGATCCCTGGATTATGAATAATTTTGTGCAAGCACCTGGTGGTGAGTTTACAGTATCCCCTAGGAATTCCCCTGGTGAAGTGCTTCTTAATTTAGAATTGGGCCCAGAAATAAATCCCTATTTGGCCCATCTTGCTAGAATGTATAATGGTTATGCAGGTGGATTTGAAGTGCAGGTAGTCCTAGCTGGAAATGCGTTTACAGCAGGAAAGATAATCTTTGCAGCTATACCCCCTAACTTTCCAATTGATAATCTAAGCGCAGCACAGATCACAATGTGCCCACATGTGATTGTGGATGTCAGACAGTTGGAACCGGTCAACCTCCCGATGCCTGATGTTCGCAACAACTTCTTTCATTACAATCAAGGGTCTGATCCGAAATTGCGCTTAATTGCAATGCTGTATACACCTCTTAGGGCAAACAATTCTGGGGATGATGTTTTTACTGTGTCTTGTAGAGTGCTGACTAGACCTAGCCCTGACTTTTCATTTAATTTCCTTGTGCCACCCACTGTGGAGTCAAAGACAAAACCCTTCACTCTCCCCATTCTGACTATCTCTGAAATGTCCAATTCTAGGTTTCCAGTGCCGATTGATTCTCTGCACACCAGCCCAACTGAGAATATTGTTGTCCAGTGCCAAAATGGGCGCGTCACTCTTGATGGTGAGTTGATGGGCACCACCCAACTCTTACCTAGTCAAATCTGTGCTTTCAGGGGCGTGCTCACCAGATCAACAAGCAGAACCAGTGATCAGGCCGACACAGCAACCCCTAGGTTGTTTAATTATTATTGGCACATACAATTGGATAATCTAAATGGGACTCCTTATGATCCTGCAGAAGACATACCAGGTCCCCTAGGGACACCAGATTTTCGGGGCAAAGTCTTTGGCGTGGCCAGCCAGAGAAATCCCGACAGCACAACTAGAGCACATGAAGCAAAGGTGGACACAACAGCTGGCCGTTTCACCCCAAAACTAGGCTCATTAGAGATATCCACTGAATCTGATGACTTTGATCAAAACCAACCAACAAGATTCACCCCAGTTGGCATTGGGGTTGACAATGAGGCAGACTTTCAACAATGGTCTTTACCCGACTATTCTGGTCAGTTCACCCACAACATGAACTTAGCCCCAGCTGTTGCTCCCAACTTCCCTGGTGAGCAGCTCCTTTTCTTCCGCTCACAGTTACCATCCTCTGGTGGGCGATCCAACGGGATTCTAGACTGCCTGGTCCCCCAAGAATGGGTTCAGCACTTCTACCAAGAATCGGCCCCCGCCCAAACTCAAGTGGCCCTGGTTAGGTATGTCAACCCTGACACTGGTAGAGTATTATTTGAGGCCAAGCTGCACAAATTAGGTTTCATGACTATAGCTAAGAATGGTGACTCTCCAATAACTGTCCCTCCAAATGGATATTTTAGGTTTGAATCTTGGGTGAACCCATTTTATACACTTGCCCCCATGGGAACTGGGAATGGGCGTAGGAGGATTCAATAA

>KF306213|II.12/II.3|2013|CN|Jingzhou/2013402

ATGAAGATGGCGTCGAATGACGCTGCTCCATCTAATGATGGTGCTGCCGGCCTCGTCCCAGAGATCAGCAGTGAGGCAATGGCGCTAGAACCAGTGGCGGGTGCAGCGATAGCAGCACCCCTCACTGGTCAGCAGAATATAATTGATCCCTGGATTATGAATAATTTTGTGCAAGCACCTGGTGGTGAGTTTACAGTATCTCCTAGAAATTCCCCTGGTGAAGTTCTTCTCAATTTGGAATTGGGTCCAGAAATAAATCCCTATTTGGCCCATCTTGCTAGAATGTATAATGGTTATGCAGGTGGATTTGAAGTACAGGTGGTCCTAGCTGGAAATGCGTTTACAGCAGGAAAGATAATCTTTGCAGCTATTCCCCCTAATTTTCCAATTGATAATCTAAGTGCAGCACAGATCACAATGTGTCCACATGTGATTGTGGATGTCAGGCAGCTGGAACCAGTCAACCTCCCGATGCCTGACGTCCGCAATAACTTCTTCCATTACAATCAAGGGTCTGATTCGAGATTGCGCCTAATTGCAATGCTATATACACCTCTTAGGGCAAATAATTCTGGGGACGATGTTTTTACTGTGTCTTGCAGAGTGCTAACTAGACCTAGCCCTGACTTCTCATTTAATTTTCTTGTGCCACCCACTGTGGAGTCAAAGACAAAACCCTTTTCCCTCCCTATTCTGACTATCTCCGAAATGTCCAATTCTAGGTTCCCAGTACCAATTGATTCTTTGCACACCAGCCCTACTGAGAACATTGTTGTCCAGTGTCAGAATGGGCGAGTCACCCTTGATGGTGAGTTGATGGGCACCACCCAACTCTTACCTAGCCAAATCTGTGCTTTCAGGGGCACGCTCACCAGATCAACAAGCAGGGCCAGTGACCAGGCTGATACAGCAACCCCTAGATTGTTTAATTATTATTGGCATATACAATTGGATAACCTAAATGGAACTCCTTATGACCCTGCAGAAGATATACCAGCCCCCCTAGGGACACCAGATTTCCGGGGTAAAGTCTTTGGCGTGGCCAGCCAGAGAAATCCTGACGCCACGACTAGGGCACATGAAGCAAAGATAGACACAACATCTGGCCGCTTCACCCCAAAACTAGGTTCACTAGAGATTTCCACTGAGTCTGGAGATTTTGACCAAAACCAACCAACAAGATTCACCCCAGTTGGCATTGGGGTTGACCATGAGCCAGATTTTCAACAATGGGCTCTTCCCGACTATGCAGGCCAGTTCACCCACAACATGAACTTAGCCCCAGCTGTTGCTCCCAACTTCCCTGGTGAGCAGCTCCTTTTCTTCCGCTCACAGTTGCCATCTTCTGGTGGGCGGTCCAACGGGATTCTAGACTGCCTGGTCCCCCAAGAATGGGTGCAGCATTTCTACCAAGAATCAGCCCCCTCCCAAACTCAAGTGGCCCTAGTTAGGTATGTCAACCCTGACACTGGTAGAGTGTTATTTGAGGCCAAGTTGCACAAATTACGTTTCATGACTATAGCCAAGAGTGGTGACTCCCCAATAACTGTCCCCCCAAATGGATACTTTAGGTTTGAATCTTGGGTGAACCCCTTTTATACACTTGCCCCCATGGGAACTGGGAATGGGCGTAGAAGGATTCAATAA

>KJ145323|II.21/II.3|2013|TW|13-BG-1

ATGAAGATGGCGTCGAATGACGCCGCTCCATCTAATGATGGTGCCGCCGGCCTCGTCCCAGAGATCAACAGTGAGGCAATGGCGCTAGAGCCAGTGGCGGGCGCAGCGATAGCAGCACCCCTCACTGGTCAGCAAAATATAATTGATCCCTGGATTATGAATAATTTTGTGCAAGCACCTGGTGGTGAGTTCACAGTATCCCCTAGAAATTCCCCTGGTGAAGTCCTTCTCAATTTGGAATTGGGTCCAGAAATAAATCCCTACTTGGCTCATCTTGCTAGGATGTATAATGGTTATGCAGGTGGATTTGAAGTGCAGGTGGTCCTAGCTGGAAATGCGTTTACAGCAGGAAAGATAATCTTTGCAGCTATTCCCCCTAATTTTCCAATTGATAATCTAAGTGCAGCACAGATCACAATGTGTCCACATGTGATTGTGGATGTTAGGCAGTTGGAACCAGTCAACCTCCCGATGCCTGACGTTCGCAATAACTTTTTCCATTACAATCAGGGGTCTGATTCGAGATTGCGCCTAATTGCAATGTTGTATACACCTCTTAGGGCAAATAACTCTGGGGATGATGTTTTTACTGTGTCTTGCAGAGTGCTAACTAGACCTAGTCCTGACTTCTCATTTAATTTCCTTGTGCCACCCACTGTGGAGTCAAAGACAAAACCCTTTTCCCTCCCCATTCTGACTATCTCTGAAATGTCCAATTCTAGGTTCCCAGTACCAATTGATTCTCTGCACACCAGCCCTACTGAGAACATTGTTGTTCAGTGCCAGAATGGACGCGTCACCCTTGATGGTGAGTTGATGGGCACCACTCAACTTTTACCTAGTCAAATCTGTGCTTTCAGGGGCATGCTTACCAGATCAACAAGCAGGGCCAGTGACCAGGCCGACACAGCAACCCCTAGATTGTTTAATTATTATTGGCATATACAATTGGATAACCTAAATGGAACTCCTTATGACCCTGCAGAAGATATACCAGGCCCCCTAGGGACACCAGATTTCCGGGGCAAAGTCTTTGGCGTGGCTAGCCAGAGAAATCCTGATGCCACGACTAGGGCACATGAAGCAAAGATAGACACAACATCTGGCCGCTTCACCCCAAAATTAGGCTCACTAGAGATTTCCACTGAGTCTGGAGATTTTGATCAAAACCAACCAACAAGATTCACCCCAGTTGGCATTGGGGTTGACCATGAGGCAGATTTTCAACAATGGACTCTTCCCGACTACGCTGGCCAGTTCACCCACAACATGAACTTAGCCCCAGCTGTTGCTCCCAACTTCCCTGGTGAGCAGCTCCTTTTCTTCCGCTCACAGTTGCCATCTTCCGGTGGGCGGTCCAACGGTATTCTAGACTGCCTGGTCCCCCAAGAATGGGTACAGCACTTCTATCAAGAATCAGCCCCCTCCCAAACTCAAGTGGCCCTGGTTAGGTATGTCAACCCCGACACTGGTAGAGTGTTATTTGAGGCCAAGCTGCACAAACTAGGTTTCATGACTATAGCCAAGAGTGGTGACTCTCCAATAACTGTCCCCCCAAATGGATACTTCAGGTTTGAATCTTGGGTGAACCCCTTTTATACACTTGCCCCCATGGGAACTGGGAATGGGCGTAGAAGGATTCAATAA

>KJ194500|II.3/II.3|1995|NL|Amsterdam/1/1995

ATGAAGATGGCGTCGAATGACGCTGCTCCATCTAATGATGGTGCCGCCGGCCTCGTCCCAGAGATCAACAATGAGGCAATGGCGCTAGAGCCAGTGGCGGGTGCAGCGATAGCAGCGCCCCTCACTGGCCAGCAAAATATAATTGATCCCTGGATTATGAATAATTTTGTGCAAGCACCTGGTGGTGAGTTTACAGTGTCGCCTAGGAATTCCCCTGGTGAAGTGCTTCTTAGTTTGGAATTAGGTCCAGAAATAAATCCCTATTTGGCTCATCTTGCTAGAATGTACAATGGTTATGCAGGTGGATTTGAAGTGCAAGTGGTCCTAGCTGGAAATGCGTTTACAGCAGGGAAGATTATCTTTGCAGCTATACCCCCTAATTTTCCTATTGACAATCTGAGCGCGGCACAGATCACAATGTGCCCGCATGTGATTGTGGATGTCAGGCAGTTGGAACCAGTCAATCTCCCGATGCCTGATGTCCGCAACAATTTCTTTCATTATAATCAAAGTTCTGAGTCAAGATTACGTTTGATTGCAATGTTGTATACACCTCTTAGGGCAAATAATTCTGGAGATGATGTTTTCACTGTGTCTTGTAGGGTGTTAACTAGGCCCAGCCCTGATTTCTCATTCAATTTTCTTGTCCCACCCACTGTGGAATCAAAGACAAAACCTTTTACCCTCCCCATTTTAACCATCTCTGAAATGTCCAATTCCAGGTTTCCAGTTCCAATTGACTCTCTGCACACCAGCCCAACTGAGAATATAGTTGTCCAGTGCCAAAATGGGCGCGTCACTCTTGACGGTGAGTTAATGGGCACCACCCAACTCTTACCGAGCCAAATATGTGCTTTCAGGGGCACACTCACTGGATCAACAAGCAGGGCCAGTGACCAAACTGACACACCAACCCCCAGGCAATTCGACCGTCGTTGGCACATACAATTGGATAATCTAAATGGAACTCCCTACGACCCTGCAGAGGACATACCAGCTCCTTTGGGCACACCAGACTTCCGGGGCAAGGTCTTTGGCGTGGCCAGCCAGAGAAACCCCGACAGAGCAACAAGGGCACATGAAGCAAAAGTGGACACAACATCTGACCGCTTCGCCCCAAAATTGGGCTCCTTAGAAATAACCACTGAATCTGGTGACTTTGAAGCAAACCAGCCAACAAAATTCACCCCAGTTGGCATCGGAGTTGACAATGAGGCAGAATTTCAGCAATGGTCCTTACCCAACTATTCTGGTCAGATTACTCACAATATGAACTTAGCCCCAGCTGTCGCCCCCAATTTTCCTGGTGAACAGCTACTTTTCTTCCGATCACAGCTGCCATCCTCTGGTGGGTGGTCCAACGGGGTTCTAGACTGCCTGCTCCCCCAGGAATGGGTTCAACACTTTTACCAAGAATCAGCCCCCGCCCAAACGCAGGTGGCCCTGGTTAGGTATGTCAACCCTGACACTGGCAGAGTGCTATTTGAGGCCAAGCTACACAAATTGGGTTTTATGACTATAGCAAAGAATGGTGACTCCCCAATAACTGTCCCTCCAAATGGATATTTTAGATTTGAATCTTGGGTTAACCCCTTTTACACACTTGCCCCCATGGGAACTGGAAACGGGCGTAGAAGGATTCAATAA

>KJ194504|II.3/II.3|1994|NL|Amsterdam/1994

ATGAAGATGGCGTCGAATGACGCTGCTCCATCTAATGATGGTGCCGCCGGCCTCGTCCCAGAGATCAACAATGAGGCAATGGCGCTAGAGCCAGTGGCGGGTGCAGCGATAGCAGCGCCCCTCACTGGCCAGCAAAATATAATTGATCCCTGGATTATGAATAATTTTGTGCAAGCACCTGGTGGTGAGTTTACAGTGTCACCTAGGAATTCCCCTGGTGAAGTGCTTCTCAATTTGGAATTAGGCCCAGAAATAAATCCCTATTTGGCTCATCTTGCTAGAATGTACAATGGTTATGCAGGTGGATTTGAAGTGCAAGTGGTCCTAGCTGGAAATGCGTTTACAGCAGGAAAGGTTATCTTTGCAGCTATACCCCCTAATTTCCCTATTGACAATCTGAGCGCGGCACAGATCACAATGTGTCCGCACGTGATTGTGGATGTCAGGCAGTTGGAACCAATCAATCTCCCGATGCCTGATGTCCGCAACAATTTCTTTCATTATAATCAAGGTTCTGATTCAAGATTACGTTTGGTTGCAATGCTGTATACACCTCTTAGGGCAAATAATTCTGGAGATGATGTTTTCACTGTGTCTTGTAGGGTGTTAACTAGGCCCAGCCCTGATTTCTCATTCAATTTTCTTGTCCCACCCACTGTGGAATCAAAGACAAAGCCTTTTACCCTCCCCATTTTAACCATCTCTGAAATGTCCAATTCCAGGTTTCCAGTTCCAATTGACTCTCTGCACACCAGCCCAACTGAGAATATAGTTGTCCAGTGCCAAAATGGGCGCGTCACTCTTGACGGTGAGTTAATGGGCACCACCCAACTCTTACCGAGCCAAATATGTGCTTTCAGGGGCACACTCACTAGATCAACAAGCAGGGCCAGTGACCAAGCCGACACACCAACCCCCAGGCAATTCGACCATCGTTGGCACATACAATTGGATAATCTAAATGGAACTCCCTACGACCCTGCAGAGGACATACCAGCTCCTTTGGGCACACCAGACTTCCGGGGTAAGGTCTTTGGCGTGGCCAGCCAGAGAAACCCCGACGGCACAACAAGGGCACATGAAGCAAAAGTGGACACAACAACTAACCGCTTCACCCCAAAATTGGGCTCCTTAGAAATAATCACTGAATCTGAAGACTTTGACACAAACCAGTCAACAAAATTCACCCCAGTTGGCGTCGGAGTTGACAATGAGGAAGAATTCCAACAATGGTCCTTACCCAACTATTCTGGTCAGTTTACTCATAATATGAACTTAGCCCCAGCTGTCGCCCCCAATTTTCCTGGTGAACAGCTACTTTTCTTCCGGTCACAGCTGCCATCCTCTGGTGGGTGGTCTAACGGGATTCTAGACTGCCTGGTCCCCCAGGAATGGGTTCAACACTTCTACCAGGAATCAGCCCCCGCCCAAACGCAGGTGGCCCTGGTTAGGTATGTCAACCCTGACACTGGCAGAGTGCTATTTGAGGCCAAGCTACACAAATTGGGTTTTATGACTATAGCAAAGAATGGTGACTCCCCAATAACTGTCCCTCCAAATGGATACTTTAGATTTGAATCTTGGGTTAACCCCTTTTATACACTTGCCCCCATGGGAACTGGAAACGGGCGTAGAAGGATTCAATAA

>KJ499441|-/II.3|2013|HK|CUHK-NS-193

ATGAAGATGGCGTCGAATGACGCCACTCCATCTAATGATGGTGCCGCCGGCCTCGTCCCAGAGATCAACAATGAGGCAATGGCGCTAGAGCCAGTGGCGGGTGCAGCGATAGCGGCACCCCTCACTGGCCAGCAAAATATAATTGATCCCTGGATTATGAATAATTTTGTGCAAGCACCTGGTGGTGAGTTCACAGTGTCTCCTAGGAATTCCCCTGGTGAAGTGCTCCTCAATTTGGAATTGGGCCAAGAAATAAACCCCTATCTGGCCCATCTTGCTAGAATGTATAATGGTTATGCAGGTGGGTTTGAAGTGCAGGTAGTCCTAGCTGGAAATGCGTTTACAGCAGGAAAGATAATCTTTGCAGCTATACCCCCTAACTTCCCAATTGACAATCTAAGTGCAGCACAGATCACAATGTGCCCACATGTGATTGTGGATGTTAGGCAGTTGGAACCGGTCAACCTCCCGATGCCTGACGTTCGCAATAACTTCTTCCACTACAACCAAGGGTCTGATTCGAGATTGCGCTTGGTTGCAATGCTGTACACACCTCTTAGGGCAAATAACTCTGGGGATGATGTTTTCACTGTGTCTTGTAGAGTGCTGACTAGACCTAGCCCTGAATTTTCATTTAACTTCCTTGTGCCACCCACTGTGGAGTCAAAGACAAAACCCTTCACCCTCCCAATTCTGACTATCTCTGAAATGTCTAATTCTAGGTTTCCAGTGCCGATTGATTCTCTGCACACCAGCCCAACTGAGAATATTGTTGTCCAGTGCCAAAATGGACGCGTCACTCTTGATGGTGAGTTGATGGGCACCACTCAGCTCTTACCTAGTCAAATCTGTGCTTTCAGGGGCGTGCTCACTAGATCAACAAGCAGGACTAGTGACCAGGCCGACACAGCAACCCCTAGATTGTTTAATTATTATTGGCACATACAATTGGATAATCTAAATGGGACTCCTTATGATCCTGCAGAAGACATACCAGGCCCCCTGGGGACGCCAGATTTCCGGGGCAAAGTCTTTGGCGTGGCCAGCCAAAGAAACCCCGACAGTACAACTAGAGCACATGAAGCAAAGGTGGACACAACAGCTGGTCGCTTCACCCCAAAACTAGGCTCATTAGAGATATCCACTGAATCTGGCGACTTTGACCAAAACCAACCAACAAGATTCACCCCAGTTGGCATTGGGGTTGACCGCGAGGCAGACTTCCAACAATGGTCCTTACCCGACTACTCTGGCCAGTTCACTCACAACATGAACTTAGCCCCAGCTGTTGCTCCCAACTTCCCTGGTGAGCAGCTCCTTTTCTTCCGCTCACAGTTACCATCTTCTGGTGGGCGATCCAATGGGATTCTAGACTGCCTGGTCCCCCAAGAATGGGTTCAGCACTTCTACCAAGAATCAGCCCCCGCCCAAACCCAGGTGGCTCTGGTTAGATATGTCAACCCTGACACTGGTAGAGTGTTGTTTGAGGCCAAGCTGCACAAATTAGGTTTCATGACTATAGCTAAGAATGGTGATTCTCCAATAACTGTCCCCCCAAATGGATACTTTAGGTTTGAATCTTGGGTGAACCCATTTTATACACTTGCCCCCATGGGAACTGGTAATGGGCGTAGAAGAGTTCAATAA

>KJ499442|-/II.3|2013|HK|CUHK-NS-201

ATGAAGATGGCGTCGAATGACGCCACTCCATCTAATGATGGTGCCGCCGGCCTCGTCCCAGAGATCAACAATGAGGCAATGGCGCTAGATCCAGTGGCGGGTGCAGCGATAGCAGCACCCCTCACTGGCCAGCAAAATATAATTGATCCCTGGATTATGAATAATTTTGTGCAAGCACCTGGTGGTGAGTTCACAGTGTCTCCTAGGAATTCCCCTGGTGAAGTGCTCCTCAATTTGGAATTGGGCCCAGAGATAAACCCCTATCTGGCCCATCTTGCTAGAATGTATAATGGTTATGCAGGTGGGTTTGAAGTGCAGGTAGTCCTAGCTGGAAATGCGTTTACAGCAGGAAAGATAATCTTTGCAGCTATACCCCCTAACTTCCCAATTGACAATCTAAGTGCAGCACAGATCACAATGTGCCCACATGTGATTGTGGATGTCAGGCAGTTGGAACCGGTCAACCTCCCGATGCCTGACGTTCGCAATAACTTCTTCCACTACAACCAAGGGTCTGATTCGAGATTGCGCTTGGTTGCAATGCTGTACACACCTCTTAGGGCAAATAACTCTGGGGATGATGTTTTCACTGTGTCTTGTAGAGTGCTGACTAGACCTAGCCCTGAATTTTCATTTAACTTCCTTGTGCCACCCACTGTGGAGTCAAAGACAAAACCCTTCACCCTCCCAATTCTGACTATCTCTGAAATGTCTAATTCTAGGTTTCCAGTGCCGATTGATTCTCTGCACACCAGCCCAACTGAGAATATTGTTGTCCAGTGCCAAAATGGACGCGTCACTCTTGATGGTGAGTTGATGGGCACCACTCAGCTCTTACCTAGTCAAATCTGTGCTTTCAGGGGCGTGCTCACTAGATCAACAAGCAGGGCTAGTGACCAGGCCGACACAGCAACCCCTAGATTGTTTAATTATTATTGGCACATACAATTGGATAATCTAAATGGGACTCCTTATGATCCTGCAGAAGACATACCAGGCCCCCTGGGGACACCAGATTTCCGGGGCAAAGTCTTTGGCGTGGCCAGCCAAAGAAACCCCGACAATACAACTAGAGCACATGAAGCAAAGGTGGACACAACAGCTGGTCGCTTCACCCCAAAACTAGGCTCATTAGAGATATCCACTGAATCTGGTGACTTTGACCAAAACCAACCAACAAGATTCACCCCAGTTGGCATTGGGGTTGACCACGAGGCAGACTTCCAACAATGGTCCTTACCCGACTACTCTGGCCAGTTCACTCACAACATGAACTTAGCCCCAGCTGTTGCTCCCAACTTCCCTGGTGAGCAGCTCCTTTTCTTCCGCTCACAGTTACCATCTTCTGGTGGGCGATCCAATGGGATTCTAGACTGCCTGGTCCCCCAAGAATGGGTTCAGCACTTCTACCAAGAATCAGCCCCCGCCCAAACCCAGGTGGCTCTGGTTAGATATGTCAACCCTGACACTGGCAGAGTGTTGTTTGAGGCCAAGCTGCACAAATTAGGTTTCATGACTATAGCTAAGAATGGTGACTCTCCAATAACTGTCCCCCCAAATGGATACTTTAGGTTTGAATCTTGGGTGAACCCATTTTATACACTTGCCCCCATGGGAACTGGGAATGGGCGTAGAAGAGTTCAATAA

>KJ499443|-/II.3|2013|HK|CUHK-NS-218

ATGAAGATGGCGTCGAATGACGCTGCTCCATCTAATGATGGTGCTGCCGGCCTCGTCCCAGAGATCAACAGTGAGGCAATGGCGCTAGAGCCAGTGGCGGGTGCAGCGATAGCGGCACCCCTCACTGGTCAGCAGAATATAATTGATCCCTGGATTATGAATAATTTTGTGCAAGCACCTGGTGGTGAGTTTACAGTATCTCCTAGAAATTCCCCTGGTGAAGTTCTTCTTAATTTGGAATTGGGTCCAGAAATAAATCCCTATTTGGCCCATCTTGCTAGAATGTATAATGGTTATGCAGGTGGATTTGAAGTGCAGGTGGTCCTAGCTGGAAATGCGTTTACAGCAGGAAAGATAATCTTTGCAGCTATCCCCCCTAATTTTCCAATTGACAATCTAAGTGCAGCACAGATCACAATGTGTCCACATGTGATTGTGGATGTCAGACAGCTGGAACCAGTCAACCTCCCGATGCCTGACGTTCGCAACAACTTCTTTCATTACAATCAAGGGTCTGATTCGAGATTGCGCCTAATTGCAATGCTATACACACCTCTTAGGGCAAACAATTCTGGGGATGATGTTTTTACTGTGTCTTGTAGAGTGCTAACTAGACCTAGCCCTGACTTCTCATTTAATTTCCTTGTGCCACCCACTGTGGAGTCAAAGACAAAACCTTTTTCCCTTCCTATTCTGACTATCTCCGAAATGTCCAATTCTAGGTTCCCAGTACCAATTGATTCTCTGCACACCAGCCCTACTGAGAACATTGTTGTCCAGTGTCAGAATGGACGAGTCACCCTTGATGGTGAGTTGATGGGCACCACCCAACTCTTACCTAGCCAAATCTGTGCTTTCAGGGGCACGCTCACCAGATCAACAAGCAGGGCCAGTGACCAGGCCGACACAGCAACCCCTAGATTGTTTAATTACTATTGGCATATACAATTGGACAACCTAAATGGAACTCCTTATGACCCTGCAGAAGATATACCAGGCCCCCTAGGGACACCAGATTTCCGGGGCAAAGTCTTTGGCGTGGCCAGCCAGAGAAATCCTGACGCCACGACTAGGGCACATGAAGCAAAGATAGACACAACATCTGGCCGCTTTACCCCAAAATTAGGTTCACTAGAGATTTCCACTGAGTCTGATGATTTTGACCAAAACCAACCAACAAGATTCACCCCAGTTGGCGTTGGGGTTGACCATGAGCCAGACTTTCAACAATGGGTTCTTCCCGACTATGCAGGCCAGTTCACCCACAACATGAACTTAGCCCCAGCTGTTGCTCCCAACTTCCCTGGTGAGCAGCTCCTTTTCTTCCGCTCACAGTTGCCGTCTTCTGGTGGGCGGTCCAACGGGATTCTAGACTGCCTGGTCCCCCAAGAATGGGTGCAGCATTTCTACCAAGAATCAGCCCCCTCCCAAACCCAAGTGGCCCTAGTTAGGTATGTCAACCCTGACACTGGTAGGGTGTTATTTGAGGCCAAGCTACACAAATTAGGTTTCATGACTATAGCCAAGAGTGGTGACTCCCCGATAACTGTCCCCCCAAATGGATATTTTAGGTTTGAATCTTGGGTGAACCCCTTTTATACACTTGCCCCCATGGGAACTGGAAATGGGCGTAGAAGGATTCAATAA

>KJ499444|-/II.3|2014|HK|CUHK-NS-227

ATGAAGATGGCGTCGAATGACGCCACTCCATCTAATGATGGTGCCGCCGGCCTCGTCCCAGAGATCAACAATGAGGCAATGGCGCTAGATCCAGTGGCGGGTGCAGCGATAGCAGCACCCCTCACTGGCCAGCAAAATATAATTGATCCCTGGATTATGAATAATTTTGTGCAAGCACCTGGTGGTGAGTTCACAGTGTCTCCTAGGAATTCCCCTGGTGAAGTGCTCCTCAATTTGGAATTGGGCCCAGAGATAAACCCCTATCTGGCCCATCTTGCTAGAATGTATAATGGTTATGCAGGTGGGTTTGAAGTGCAGGTAGTCCTAGCTGGAAATGCGTTTACAGCAGGAAAGATAATCTTTGCAGCTATACCCCCTAACTTCCCAATTGACAATCTAAGTGCAGCACAGATCACAATGTGCCCACATGTGATTGTGGATGTCAGGCAGTTGGAACCGGTCAACCTCCCGATGCCTGACGTTCGCAATAACTTCTTCCACTACAACCAAGGGTCTGATTCGAGATTGCGCTTGGTTGCAATGCTGTACACACCTCTTAGGGCAAATAACTCTGGGGATGATGTTTTCACTGTGTCTTGTAGAGTGCTGACTAGACCTAGCCCTGAATTTTCATTTAACTTCCTTGTGCCACCCACTGTGGAGTCAAAGACAAAACCCTTCACCCTCCCAATTCTGACTATCTCTGAAATGTCTAATTCTAGGTTTCCAGTGCCGATTGATTCTCTGCACACCAGCCCAACTGAGAATATTGTTGTCCAGTGCCAAAATGGACGCGTCACTCTTGATGGTGAGTTGATGGGCACCACTCAGCTCTTACCTAGTCAAATCTGTGCTTTTAGGGGCGTGCTCACTAGATCAACAAGCAGGGCTAGTGACCAGGCCGACACAGCAACCCCTAGATTGTTTAATTATTATTGGCACATACAATTGGATAATCTAAATGGGACTCCTTATGATCCTGCAGAAGACATACCAGGCCCCCTGGGGACACCAGATTTCCGGGGCAAAGTCTTTGGCGTGGCCAGCCAAAGAAACCCCGACAGTACAACTAGAGCACATGAAGCAAAGGTGGACACAACAGCTGGTCGCTTCACCCCAAAACTAGGCTCATTAGAGATATCCACTGAATCTGGTGACTTTGACCAAAACCAACCAACAAGATTCACCCCAGTTGGCATTGGGGTTGACCACGAGGCAGACTTCCAACAATGGTCCTTACCCGACTACTCTGGCCAGTTCACTCACAACATGAACTTAGCCCCAGCTGTTGCTCCCAACTTCCCTGGTGAGCAGCTCCTTTTCTTCCGCTCACAGTTACCATCTTCTGGTGGGCGATCCAATGGGATTCTAGACTGCCTGGTCCCCCAAGAATGGGTTCAGCACTTCTACCAAGAATCAGCCCCCGCCCAAACCCAGGTGGCTCTGGTTAGATATGTCAACCCTGACACTGGTAGAGTGTTGTTTGAGGCCAAGCTGCACAAATTAGGTTTCATGACTATAGCTAAGAATGGTGACTCTCCAATAACTGTCCCCCCAAATGGATACTTTAGGTTTGAATCTTGGGTGAACCCATTTTATACACTTGCCCCCATGGGAACTGGGAATGGGCGTAGAAGAGTTCAATAA

>KJ499445|-/II.3|2014|HK|CUHK-NS-232

ATGAAGATGGCGTCGAATGACGCCACTCCATCTAATGATGGTGCCGCCGGCCTCGTCCCAGAGATCAACAATGAGGCAATGGCGCTAGATCCAGTGGCGGGTGCAGCGATAGCAGCACCCCTCACTGGCCAGCAAAATATAATTGATCCCTGGATTATGAATAATTTTGTGCAAGCACCTGGTGGTGAGTTCACAGTGTCTCCTAGGAATTCCCCTGGTGAAGTGCTCCTCAATTTGGAATTGGGCCCAGAGATAAACCCCTATCTGGCCCATCTTGCTAGAATGTATAATGGTTATGCAGGTGGGTTTGAAGTGCAGGTAGTCCTAGCTGGAAATGCGTTTACAGCAGGAAAGATAATCTTTGCAGCTATACCCCCTAACTTCCCAATTGACAATCTAAGTGCAGCACAGATCACAATGTGCCCACATGTGATTGTGGATGTCAGGCAGTTGGAACCGGTCAACCTCCCGATGCCTGACGTTCGCAATAACTTCTTCCACTACAACCAAGGGTCTGATTCGAGGTTGCGCTTGGTTGCAATGCTGTACACACCTCTTAGGGCAAATAACTCTGGGGATGATGTTTTCACTGTGTCTTGTAGAGTGCTGACTAGACCTAGCCCTGAATTTTCATTTAACTTCCTTGTGCCACCCACTGTGGAGTCAAAGACAAAACCCTTCACCCTCCCAATTCTGACTATCTCTGAAATGTCTAATTCTAGGTTTCCAGTGCCGATTGATTCTCTGCACACCAGCCCAACTGAGAATATTGTTGTCCAGTGCCAAAATGGACGCGTCACTCTTGATGGTGAGTTGATGGGCACCACTCAGCTCTTACCTAGTCAAATCTGTGCTTTCAGGGGCGTGCTCACTAGATCAACAAGCAGGACTAGTGACCAGGCCGACACAGCAACCCCTAGATTGTTTAATTATTATTGGCACATACAATTGGATAATCTAAATGGGACTCCTTATGATCCTGCAGAAGACATACCAGGCCCCCTGGGGACACCAGATTTCCGGGGCAAAGTCTTTGGCGTGGCCAGCCAAAGAAACCCCGACAGTACAACTAGAGCACATGAAGCAAAGGTGGACACAACAGCTGGTCGCTTCACCCCAAAACTAGGCTCATTAGAGATATCCACTGAATCTGGTGACTTTGACCAAAACCAACCAACAAGATTCACCCCAGTTGGCATTGGGGTTGACCACGAGGCAGACTTCCAACAATGGTCCTTACCCGACTACTCTGGCCAGTTCACCCACAACATGAACTTAGCCCCAGCTGTTGCTCCCAACTTCCCTGGTGAGCAGCTCCTTTTCTTCCGCTCACAGTTACCATCTTCTGGTGGGCGATCCAATGGGATTCTAGACTGCCTGGTCCCCCAAGAATGGGTTCAGCACTTCTACCAAGAATCAGCCCCCGCCCAAACCCAGGTGGCTCTGGTTAGATATGTCAACCCTGACACTGGTAGAGTGTTGTTTGAGGCCAAGCTGCACAAATTAGGTTTCATGACTATAGCTAAGAATGGTGACTCTCCAATAACTGTCCCCCCAAATGGATACTTTAGGTTTGAATCTTGGGTGAACCCATTTTATACACTTGCCCCCATGGGAACTGGGAATGGGCGTAGAAGAGTTCAATAA

>KM198484|II.21/II.3|2009|VN|30212/2009

ATGAAGATGGCGTCGAATGACGCCGCTCCATCTAATGATGGTGCCGCCGGCCTCGTCCCAGAGATCAACAGTGAGGCAATGGCGCTAGAGCCAGTGGCGGGTGCAGCGATAGCAGCACCCCTCACTGGTCAGCAAAATATAATTGATCCCTGGATTATGAATAATTTTGTGCAAGCACCTGGTGGTGAGTTCACAGTATCCCCTAGAAATTCCCCTGGTGAAGTTCTTCTCAATTTGGAATTGGGTCCAGAAATAAATCCCTACTTGGCCCATCTTGCTAGAATGTATAATGGTTATGCAGGTGGATTTGAAGTGCAGGTGCTCCTAGCTGGAAATGCGTTTACAGCAGGAAAGATAATCTTTGCAGCTATTCCCCCTAATTTTCCAATTGATAATCTAAGTGCAGCACAGATCACAATGTGTCCACATGTGATTGTGGATGTTAGACAGCTGGAACCAGTCAACCTCCCGATGCCTGACGTTCGCAACAACTTTTTCCATTACAATCAGGGGTCTGATTCGAGATTGCGCCTAATTGCAATGCTATATACACCTCTTAGGGCAAATAACTCTGGGGATGATGTTTTCACTGTGTCTTGCAGAGTGCTAACTAGACCTAGTCCTGACTTCTCATTTAATTTCCTTGTGCCACCCACTGTGGAGTCAAAGACAAAACCCTTTTCCCTCCCCATTCTGACTATCTCTGAAATGTCCAATTCTAGGTTCCCAGTACCAATTGATTCTCTGCACACCAGCCCTACTGAGAACATTGTTGTCCAGTGCCAGAATGGACGCGTCACCCTTGATGGTGAGTTGATGGGCACCACCCAACTTTTACCTAGCCAAATCTGTGCTTTCAGGGGCATGCTCACCAGATCAACAAGCAGGGCCAGTGACCAGGCCGATACAGCAACCCCTAGATTGTTTAATTATTATTGGCATATACAATTGGATAACCTAAATGGAACTCCTTATGACCCTGCAGAAGATATACCAGGCCCCCTAGGGACACCAGATTTCCGGGGCAAAGTCTTTGGCGTGGCCAGCCAGAGAAATCCTGATGCCACGACTAGGGCACATGAAGCAAAGATAGACACAACATCTGGCCGCTTCACCCCAAAATTAGGCTCACTAGAGATTTCCACTGAGTCTGGAGATTTTGATCAAAACCAACCAACAAGATTCACCCCAGTTGGCATTGGGGTTGACCATGAGGCAGATTTTCAACAATGGACTCTTCCCGACTATGCTGGCCAGTTCACCCACAACATGAACTTAGCCCCAGCTGTTGCTCCCAACTTCCCTGGTGAGCAGCTCCTTTTCTTCCGCTCACAGTTGCCATCTTCTGGTGGGCGGTCCAACGGGATTCTAGACTGCCTGGTCCCCCAAGAATGGGTACAGCACTTCTACCAAGAATCAGCCCCCTCCCAAACTCAAGTGGCCCTGGTTAGGTATGTCAACCCTGACACTGGTAGAGTGTTATTTGAGGCCAAGCTGCACAAATTAGGTTTCATGACTATAGCCAAGAGTGGTGACTCTCCAATAACTGTCCCCCCAAATGGATACTTTAGGTTTGAATCTTGGGTGAACCCCTTTTATACACTTGCCCCCATGGGAACTGGGAATGGGCGTAGAAGGATTCAATAA

>KM198496|II.21/II.3|2010|VN|20419/2010

ATGAAGATGGCGTCGAATGACGCCGCTCCATCTAATGATGGTGCCGCCGGCCTCGTCCCAGAGATCAACAGTGAGGCAATGGCGCTAGAACCAGTGGCGGGTGCAGCGATAGCAGCACCCCTCACTGGTCAGCAAAATATAATTGATCCCTGGATTATGAATAATTTTGTGCAAGCACCTGGTGGTGAGTTCACAGTATCCCCTAGAAATTCCCCTGGTGAAGTTCTTCTCAATTTGGAATTGGGTCCAGAAATAAATCCCTACTTGGCCCATCTTGCTAGAATGTATAATGGTTATGCAGGTGGATTTGAAGTGCAGGTGGTCCTAGCTGGAAATGCGTTTACAGCAGGAAAGATAATCTTTGCAGCTATTCCCCCTAATTTTCCAATTGATAATCTAAGTGCAGCACAGATCACAATGTGTCCACATGTGATTGTGGATGTTAGACAGCTGGAACCAGTCAACCTCCCGATGCCTGACGTTCGCAATAACTTTTTCCATTACAATCAGGGGTCTGATTCGAGATTGCGCCTAATTGCAATGCTATATACACCTCTTAGGGCAAATAACTCTGGGGATGATGTTTTCACTGTGTCTTGCAGAGTGCTAACTAGACCTAGTCCTGACTTCTCATTTAATTTCCTTGTGCCACCCACTGTGGAGTCAAAGACAAAACCCTTTTCCCTCCCCATTCTGACTATCTCTGAAATGTCCAATTCTAGGTTCCCAGTACCAATTGATTCTCTGCACACCAGCCCTACTGAGAACATTGTTGTCCAGTGCCAGAATGGACGCGTCACCCTTGATGGTGAGTTGATGGGCACCACCCAACTTTTACCTAGCCAAATCTGTGCTTTCAGGGGCATGCTCACCAGATCAACAAGCAGGGCCAGTGACCAGGCCGATACAGCAACCCCTAGATTGTTTAATTATTATTGGCATATACAACTGGATAACCTAAATGGAACTCCTTATGACCCTGCAGAAGATATACCAGGCCCCCTAGGGACACCAGATTTCCGGGGCAAAGTCTTTGGCGTGGCCAGCCAGAGAAATCCTGATGCCACGACTAGGGCACATGAAGCAAAGATAGACACAACATCTGGCCGCTTCACCCCAAAATTAGGCTCACTAGAGATTTCCACTGAGTCTGGAGATTTTGATCAAAACCAACCAACAAGATTCACCCCAGTTGGCATTGGGGTTGACCATGAGGCAGATTTTCAACAATGGACTCTTCCCGACTATGCTGGCCAGTTCACCCACAACATGAACTTAGCCCCAGCTGTTGCTCCCAACTTCCCTGGTGAGCAGCTCCTTTTCTTCCGCTCACAGTTGCCATCTTCTGGTGGGCGGTCCAACGGGATTCTAGACTGCCTGGTCCCCCAAGAATGGGTACAGCACTTCTACCAAGAATCAGCCCCCTCCCAAACTCAAGTGGCCCTGGTTAGGTATGTCAACCCTGACACTGGTAGAGTGTTATTTGAGGCCAAGCTGCACAAATTAGGTTTCATGACTATAGCCAAGAGTGGTGACTCTCCAATAACTGTCCCCCCAAATGGATACTTCAGGTTTGAATCTTGGGTGAACCCCTTTTATACACTTGCCCCCATGGGAACTGGGAATGGGCGTAGAAGGATTCAATAA

>KM198500|II.21/II.3|2011|VN|C2H-20/2011

ATGAAGATGGCGTCGAATGACGCCGCTCCATCTAATGATGGTGCCGCCGGCCTCGTCCCAGAGATCAACAGTGAGGCAATGGCGCTAGAGCCAGTGGCGGGTGCAGCGATAGCAGCACCCCTCACTGGTCAGCAAAATATAATTGATCCCTGGATTATGAATAATTTTGTGCAAGCACCTGGTGGTGAGTTCACAGTATCCCCTAGAAATTCCCCTGGTGAAGTTCTTCTCAATTTGGAATTGGGTCCAGAAATAAATCCCTACTTGGCCCATCTTGCTAGAATGTATAATGGTTATGCAGGTGGATTTGAAGTGCAGGTGGTCCTAGCTGGAAATGCGTTTACAGCAGGAAAGATAATCTTTGCAGCTATTCCCCCTAATTTTCCAATTGATAATCTAAGTGCAGCACAGATCACAATGTGCCCACATGTGATTGTGGATGTTAGACAGCTGGAACCAGTCAACCTCCCGATGCCTGACGTTCGCAATAACTTTTTCCATTACAATCAGGGGTCTGATTCGAGATTGCGCCTAATTGCAATGCTGTATACACCTCTTAGGGCAAATAACTCTGGGGATGATGTTTTCACTGTGTCTTGCAGAGTGCTAACTAGACCTAGTCCTGACTTCTCATTTAATTTCCTTGTGCCACCCACTGTGGAGTCAAAGACAAAACCCTTTTCCCTCCCCATTCTGACTATCTCTGAAATGTCCAATTCTAGGTTCCCAGTACCAATTGATTCTCTGCACACCAGTCCTACTGAGAACATTGTTGTCCAGTGCCAGAACGGACGCGTCACCCTTGATGGTGAGTTGATGGGCACCACTCAACTTTTACCTAGCCAAATCTGTGCTTTCAGGGGCATGCTCACCAGATCAACAAGCAGGGCCAGTGACCAGGCCGATACAGCAACCCCTAGATTGTTTAATTATTATTGGCATATACAATTGGATAACCTAAATGGAACTCCTTATGACCCTGCAGAAGATATACCAGGCCCCCTAGGGACACCAGATTTCCGGGGCAAAGTCTTTGGCGTGGCCAGCCAGAGAAATCCTGATGCCACGACTAGGGCACATGAAGCAAAGATAGACACAACATCTGGCCGCTTCACTCCAAAATTAGGCTCACTAGAGATTTCCACTGAGTCTGGAGATTTTGATCAAAACCAACCAACAAGATTCACCCCAGTTGGCATTGGGGTTGACCATGAGGCAGATTTTCAACAATGGACTCTTCCCGACTACGCTGGCCAGTTCACCCACAACATGAACTTAGCCCCAGCTGTTGCCCCCAACTTCCCCGGTGAGCAGCTCCTTTTCTTTCGCTCACAGTTGCCATCTTCTGGCGGGCGGTCCAACGGGATTCTAGACTGCCTGGTCCCCCAAGAATGGGTACAGCACTTCTATCAAGAATCAGCCCCCTCCCAAACTCAAGTGGCCCTGGTTAGGTATGTCAACCCTGACACTGGTAGAGTGTTATTTGAGGCCAAGCTGCACAAATTAGGTTTCATGACTATAGCCAAGAGTGGTGACTCTCCAATAACTGTCCCCCCAAATGGATACTTCAGGTTTGAATCTTGGGTGAACCCCTTTTATACACTTGCCCCCATGGGAACTGGGAATGGGCGTAGAAGGATTCAATAA

>KM198505|II.21/II.3|2010|VN|20460/2010

ATGAAGATGGCGTCGAATGACGCCGCTCCATCTAATGATGGTGCCGCCGGCCTCGTCCCAGAGATCAACAGTGAGGCAATGGCGCTAGAGCCAGTGGCGGGTGCAGCGATAGCAGCACCCCTCACTGGTCAGCAAAATATAATTGATCCCTGGATTATGAATAATTTTGTGCAAGCACCTGGTGGTGAGTTCACAGTATCCCCTAGAAATTCCCCTGGTGAAGTTCTTCTCAATTTGGAATTGGGTCCAGAAATAAATCCCTACTTGGCCCATCTTGCTAGAATGTATAATGGTTATGCAGGTGGATTTGAAGTGCAGGTGGTCCTAGCTGGAAATGCGTTTACAGCAGGAAAGATAATCTTTGCAGCTATTCCCCCTAATTTTCCAATTGATAATCTAAGTGCAGCACAGATCACAATGTGTCCACATGTGATTGTGGATGTTAGACAGCTGGAACCAGTCAACCTCCCGATGCCTGATGTTCGCAATAACTTTTTCCATTACAATCAGGGGTCTGATTCGAGATTGCGCCTAATTGCAATGCTATATACACCTCTTAGGGCAAATAACTCTGGGGATGATGTTTTCACTGTGTCTTGCAGAGTGCTAACTAGACCTAGTCCTGACTTCTCATTTAATTTTCTTGTGCCACCCACTGTGGAGTCAAAGACAAAACCCTTTTCCCTCCCCATTCTGACTATCTCTGAAATGTCCAATTCTAGGTTCCCAGTACCAATTGATTCTCTGCACACCAGCCCTACTGAGAACATTGTCGTCCAGTGCCAGAATGGACGCGTCACCCTTGATGGTGAGTTGATGGGCACCACCCAACTTTTACCTAGCCAAATCTGTGCTTTCAGGGGCATGCTCACCAGATCAACAAGCAGGGCCAGTGACCAGGCCGATACAGCAACCCCTAGATTGTTTAATTATTATTGGCATATACAATTGGATAACCTAAATGGAACTCCTTATGACCCTGCAGAAGATATACCAGGCCCCCTAGGGACACCAGATTTCCGGGGCAAAGTCTTTGGCGTGGCCAGCCAGAGAAATCCTGATGCCACGACTAGGGCACATGAAGCAAAGATAGACACAACATCTGGCCGCTTCACCCCAAAATTAGGCTCACTAGAGATTTCCACTGAGTCTGGAGATTTTGATCAAAACCAACCAACAAGATTCACCCCAGTTGGCATTGGGGTTGACCATGAGGCAGATTTTCAACAATGGACTCTTCCCGACTATGCTGGCCAGTTCACCCACAACATGAACTTAGCCCCAGCTGTTGCTCCCAACTTCCCTGGTGAGCAGCTCCTTTTCTTCCGCTCACAGTTGCCATCTTCTGGTGGGCGGTCCAACGGGATTCTAGACTGCCTGGTCCCCCAAGAATGGGTACAGCACTTCTACCAAGAATCAGCCCCCTCCCAAACTCAAGTGGCCCTGGTTAGGTATGTCAACCCTGACACTGGTAGAGTGTTATTTGAGGCCAAGCTGCACAAATTAGGTTTCATGACTATAGCCAAGAGTGGTGACTCTCCAATAACTGTCCCCCCAAATGGATACTTCAGGTTTGAATCTTGGGTGAACCCCTTTTATACACTTGCCCCCATGGGAACTGGGAATGGGCGTAGAAGGATTCAATAA

>KM198509|II.21/II.3|2010|VN|20479/2010

ATGAAGATGGCGTCGAATGACGCCGCTCCATCTAATGATGGTGCCGCCGGCCTCGTCCCAGAGATCAACAGTGAGGCAATGGCGCTAGAGCCAGTGGCGGGTGCAGCGATAGCAGCACCCCTCACTGGTCAGCAAAATATAATTGATCCCTGGATTATGAATAATTTTGTGCAAGCACCTGGTGGTGAGTTCACAGTATCCCCTAGAAATTCCCCTGGTGAAGTTCTTCTCAATTTGGAATTGGGTCCAGAAATAAATCCCTACTTGGCCCATCTTGCTAGAATGTATAATGGTTATGCAGGTGGATTTGAAGTGCAGGTGGTCCTAGCTGGAAATGCGTTTACAGCAGGAAAGATAATCTTTGCAGCTATTCCCCCTAATTTTCCAATTGATAATCTAAGTGCAGCACAAATCACAATGTGTCCACATGTGATTGTGGATGTTAGACAGCTGGAACCAGTCAACCTCCCGATGCCTGACGTACGCAATAACTTTTTCCATTACAATCAGGGGTCTGATTCGAGATTGCGCCTAATTGCAATGCTATATACACCTCTTAGGGCAAATAACTCTGGGGATGATGTTTTCACTGTGTCTTGCAGAGTGCTAACTAGACCTAGTCCTGACTTCTCATTTAATTTCCTTGTGCCACCCACTGTGGAGTCAAAGACAAAACCCTTTTCCCTCCCCATTCTGACTATCTCTGAAATGTCCAATTCTAGGTTCCCAGTACCAATTGATTCTCTGCACACCAGCCCTACTGAGAACATTGTTGTCCAGTGCCAGAATGGACGCGTCACCCTTGATGGTGAGTTGATGGGCACCACCCAACTTTTACCTAGCCAAATCTGTGCTTTCAGGGGCATGCTCACCAGATCAACAAGCAGGGCCAGTGACCAGGCCGATACAGCAACCCCTAGATTGTTTAATTATTATTGGCATATACAATTGGATAACCTAAATGGAACTCCTTATGACCCTGCAGAAGATATACCAGGCCCCCTAGGGACACCAGATTTCCGGGGCAAAGTCTTTGGCGTGGCCAGCCAGAGAAATCCTGATGCCACGACTAGGGCACATGAAGCAAAGATAGACACAACATCTGGCCGCTTCACCCCAAAATTAGGCTCACTAGAGATTTCCACTGAGTCTGGAGATTTTGATCAAAACCAACCAACAAGATTCACCCCAGTTGGCATTGGGGTTGACCATGAGGCAGATTTTCAACAATGGACTCTTCCCGACTATGCTGGCCAGTTCACCCACAACATGAACTTAGCCCCAGCTGTTGCTCCCAACTTCCCTGGTGAGCAGCTCCTTTTCTTCCGCTCACAGTTGCCATCTTCTGGTGGGCGGTCCAACGGGATTCTAGACTGCCTGGTCCCCCAAGAATGGGTACAGCACTTCTACCAAGAATCAGCCCCCTCCCAAACTCAAGTGGCCCTGGTTAGGTATGTCAACCCTGACACTGGTAGAGTGTTATTTGAGGCCAAGCTGCACAAATTAGGTTTCATGACTATAGCCAAGAGTGGTGACTCTCCAATAACTGTCCCCCCAAATGGATACTTCAGGTTTGAATCTTGGGTGAACCCCTTTTATACACTTGCCCCCATGGGAACTGGGAATGGGCGTAGAAGGATTCAATAA

>KM198511|II.21/II.3|2011|VN|C2H-24/2011

ATGAAGATGGCGTCGAATGACGCCGCTCCATCTAATGATGGTGCCGCCGGCCTCGTCCCAGAGATCAACAGTGAGGCAATGGCGCTAGAGCCAGTGGCGGGTGCAGCGATAGCAGCACCCCTCACTGGTCAGCAAAATATAATTGATCCCTGGATTATGAATAATTTTGTGCAAGCACCTGGTGGTGAGTTCACAGTATCCCCTAGAAATTCCCCTGGTGAAGTTCTTCTCAATTTGGAATTGGGTCCAGAAATAAATCCCTACTTGGCCCATCTTGCTAGAATGTATAATGGTTATGCAGGTGGATTTGAAGTGCAGGTGGTCCTAGCTGGAAATGCGTTTACAGCAGGAAAGATAATCTTTGCAGCTATTCCCCCTAATTTTCCAATTGATAATCTAAGTGCAGCACAGATCACAATGTGCCCACATGTGATTGTGGATGTTAGACAGCTGGAACCAGTCAACCTCCCGATGCCTGACGTTCGCAATAACTTTTTCCATTACAATCAGGGGTCTGATTCGAGATTGCGCCTAATTGCAATGCTGTATACACCTCTTAGGGCAAATAACTCTGGGGATGATGTTTTCACTGTGTCTTGCAGAGTGCTAACTAGACCTAGTCCTGACTTCTCATTTAATTTCCTTGTGCCACCCACTGTGGAGTCAAAGACAAAACCCTTTTCCCTCCCCATTCTGACTATCTCTGAAATGTCCAATTCTAGGTTCCCAGTACCAATTGATTCTCTGCACACCAGTCCTACTGAGAACATTGTTGTCCAGTGCCAGAACGGACGCGTCACCCTTGATGGTGAGTTGATGGGCACCACTCAACTTTTACCTAGCCAAATCTGTGCTTTCAGGGGCATGCTCACCAGATCAACAAGCAGGGCCAGTGACCAGGCCGATACAGCAACCCCTAGATTGTTTAATTATTATTGGCATATACAATTGGATAACCTAAATGGAACTCCTTATGACCCTGCAGAAGATATACCAGGCCCCCTAGGGACACCAGATTTCCGGGGCAAAGTCTTTGGCGTGGCCAGCCAGAGAAATCCTGATGCCACGACTAGGGCACATGAAGCAAAGATAGACACAACATCTGGCCGCTTCACTCCAAAATTAGGCTCACTAGAGATTTCCACTGAGTCTGGAGATTTTGATCAAAACCAACCAACAAGATTCACCCCAGTTGGCATTGGGGTTGACCATGAGGCAGATTTTCAACAATGGACTCTTCCCGACTACGCTGGCCAGTTCACCCACAACATGAACTTAGCCCCAGCTGTTGCCCCCAACTTCCCCGGTGAGCAGCTCCTTTTCTTTCGCTCACAGTTGCCATCTTCTGGCGGGCGGTCCAACGGGATTCTAGACTGCCTGGTCCCCCAAGAATGGGTACAGCACTTCTATCAAGAATCAGCCCCCTCCCAAACTCAAGTGGCCCTGGTTAGGTATGTCAACCCTGACACTGGTAGAGTGTTATTTGAGGCCAAGCTGCACAAATTAGGTTTCATGACTATAGCCAAGAGTGGTGACTCTCCAATAACTGTCCCCCCAAATGGATACTTCAGGTTTGAATCTTGGGTGAACCCCTTTTATACGCTTGCCCCCATGGGAACTGGGAATGGGCGTAGAAGGATTCAATAA

>KM198528|II.21/II.3|2011|VN|C2H-25/2011

ATGAAGATGGCGTCGAATGACGCCGCTCCATCTAATGATGGTGCCGCCGGCCTCGTCCCAGAGATCAACAGTGAGGCAATGGCGCTAGAGCCAGTGGCGGGTGCAGCGATAGCAGCACCCCTCACTGGTCAGCAAAATATAATTGATCCCTGGATTATGAATAATTTTGTGCAAGCACCTGGTGGTGAGTTCACAGTATCCCCTAGAAATTCCCCTGGTGAAGTTCTTCTCAATTTGGAATTGGGTCCAGAAATAAATCCCTACTTGGCCCATCTTGCTAGAATGTATAATGGTTATGCAGGTGGATTTGAAGTGCAGGTGGTCCTAGCTGGAAATGCGTTTACAGCAGGAAAGATAATCTTTGCAGCTATTCCCCCTAATTTTCCAATTGATAATCTAAGTGCAGCACAGATCACAATGTGCCCACATGTGATTGTGGATGTTAGACAGCTGGAACCAGTCAACCTCCCGATGCCTGACGTTCGCAATAACTTTTTCCATTACAATCAGGGGTCTGATTCGAGATTGCGCCTAATTGCAATGCTGTATACACCTCTTAGGGCAAATAACTCTGGGGATGATGTTTTCACTGTGTCTTGCAGAGTGCTAACTAGACCTAGTCCTGACTTCTCATTTAATTTCCTTGTGCCACCCACTGTGGAGTCAAAGACAAAACCCTTTTCCCTCCCCATTCTGACCATCTCTGAAATGTCCAATTCTAGGTTCCCAGTACCAATTGATTCTCTGCACACCAGTCCTACTGAGAACATTGTTGTCCAGTGCCAGAACGGACGCGTCACCCTTGATGGTGAGTTGATGGGCACCACTCAACTTTTACCTAGCCAAATCTGTGCTTTCAGGGGCATGCTCACCAGATCAACAAGCAGGGCCAGTGACCAGGCCGATACAGCAACCCCTAGATTGTTTAATTATTATTGGCATATACAATTGGATAACCTAAATGGAACTCCTTATGACCCTGCAGAAGATATACCAGGCCCCCTAGGGACACCAGATTTCCGGGGCAAAGTCTTTGGCGTGGCCAGCCAGAGAAATCCTGATGCCACGACTAGGGCACATGAAGCAAAGATAGACACAACATCTGGCCGCTTCACTCCAAAATTAGGCTCACTAGAGATTTCCACTGAGTCTGGAGATTTTGATCAAAACCAACCAACAATATTCACCCCAGTTGGCATTGGGGTTGACCATGAGGCAGATTTTCAACAATGGACTCTTCCCGACTACGCTGGCCAGTTCACCCACAACATGAACTTAGCCCCAGCTGTTGCCCCCAACTTCCCCGGTGAGCAGCTCCTTTTCTTTCGCTCACAGTTGCCATCTTCTGGCGGGCGGTCCAACGGGATTCTAGACTGCCTGGTCCCCCAAGAATGGGTACAGCACTTCTATCAAGAATCAGCCCCCTCCCAAACTCAAGTGGCCCTGGTTAGGTATGTCAACCCTGACACTGGTAGAGTGTTATTTGAGGCCAAGCTGCACAAATTAGGTTTCATGACTATAGCCAAGAGTGGTGACTCTCCAATAACTGTCCCCCCAAATGGATACTTCAGGTTTGAATCTTGGGTGAACCCCTTTTATACACTTGCCCCCATGGGAACTGGGAATGGGCGTAGAAGGATTCAATAA

>KM198529|II.21/II.3|2011|VN|C2H-27/2011

ATGAAGATGGCGTCGAATGACGCCGCTCCATCTAATGATGGTGCCGCCGGCCTCGTCCCAGAGATCAACAGTGAGGCAATGGCGCTAGAGCCAGTGGCGGGTGCAGCGATAGCAGCACCCCTCACTGGTCAGCAAAATATAATTGATCCCTGGATTATGAATAATTTTGTGCAAGCACCTGGTGGTGAGTTCACAGTATCCCCTAGAAATTCCCCTGGTGAAGTTCTTCTCAATTTGGAATTGGGTCCAGAAATAAATCCCTACTTGGCCCATCTTGCTAGAATGTATAATGGTTATGCAGGTGGATTTGAAGTGCAGGTGGTCCTAGCTGGAAATGCGTTTACAGCAGGAAAGATAATCTTTGCAGCCATTCCCCCTAATTTTCCAATTGATAATCTAAGTGCAGCACAGATCACAATGTGCCCACATGTGATTGTGGATGTTAGACAGCTGGAACCAGTCAACCTCCCGATGCCTGACGTTCGCAATAACTTTTTCCATTACAATCAGGGGTCTGATTCGAGATTGCGCCTAATTGCAATGCTGTATACACCTCTTAGGGCAAATAACTCTGGGGATGATGTTTTCACTGTGTCTTGCAGAGTGCTAACTAGACCTAGTCCTGACTTCTCATTTAATTTCCTTGTGCCACCCACTGTGGAGTCAAAGACAAAACCCTTTTCCCTCCCCATTCTAACTATCTCTGAAATGTCCAATTCTAGGTTCCCAGTACCAATTGATTCTCTGCACACCAGTCCTACTGAGAACATTGTTGTCCAGTGCCAGAACGGACGCGTCACCCTTGATGGTGAGTTGATGGGCACCACTCAACTTTTACCTAGCCAAATCTGTGCTTTCAGGGGCATGCTCACCAGATCAACAAGCAGGGCCAGTGACCAGGCCGATACAGCAACCCCTAGATTGTTTAATTATTATTGGCATATACAATTGGATAACCTAAATGGAACTCCTTATGACCCTGCAGAAGATATACCAGGCCCCCTAGGGACACCAGATTTCCGGGGCAAAGTCTTTGGCGTGGCCAGCCAGAGAAATCCTGATGCCACGACTAGGGCACATGAAGCAAAGATAGACACAACATCTGGCCGCTTCACTCCAAAATTAGGCTCACTAGAGATTTCCACTGAATCTGGAGATTTTGATCAAAATCAACCAACAAGATTCACCCCAGTTGGCATTGGGGTTGACCACGAGGCAGATTTTCAACAATGGACTCTTCCCGACTACGCTGGCCAGTTCACCCACAACATGAACTTAGCCCCAGCTGTTGCTCCCAACTTCCCCGGTGAGCAGCTCCTTTTCTTTCGCTCACAGTTGCCATCTTCTGGCGGGCGGTCCAACGGGATTCTAGACTGCCTGGTCCCCCAAGAATGGGTGCAGCACTTCTATCAAGAATCGGCCCCCTCCCAAACTCAAGTGGCCCTGGTTAGGTATGTCAACCCTGACACTGGTAGAGTGTTATTTGAGGCCAAGCTGCACAAATTAGGTTTCATGACTATAGCCAAGAGTGGTGACTCTCCAATAACTGTCCCCCCAAATGGATACTTCAGGTTTGAATCTTGGGTGAACCCCTTTTATACGCTTGCCCCCATGGGAACTGGGAATGGGCGTAGAAGGATTCAATAA

>KM198547|II.21/II.3|2011|VN|C2H-47/2011

ATGAAGATGGCGTCGAATGACGCCGCTCCATCTAATGATGGTGCCGCCGGCCTCGTCCCAGAGATCAACAGTGAGGCAATGGCGCTAGAGCCAGTGGCGGGTGCAGCGATAGCAGCACCCCTCACTGGTCAGCAAAATATAATTGATCCCTGGATTATGAATAATTTTGTGCAAGCACCTGGTGGTGAGTTCACAGTATCCCCTAGAAATTCCCCTGGTGAAGTTCTTCTCAATTTGGAATTGGGTCCAGAAATAAATCCCTACTTGGCCCATCTTGCTAGAATGTATAATGGTTATGCAGGTGGATTTGAAGTGCAGGTGGTCCTAGCTGGAAATGCGTTTACAGCAGGAAAGATAATCTTTGCAGCTATTCCCCCTAATTTTCCAATTGATAATCTAAGTGCAGCACAGATCACAATGTGCCCACATGTGATTGTGGATGTTAGACAGCTGGAACCAGTCAACCTCCCGATGCCTGACGTTCGCAATAACTTTTTCCATTACAATCAGGGGTCTGATTCGAGATTGCGCCTAATTGCAATGCTGTATACACCTCTTAGGGCAAATAACTCTGGGGATGATGTTTTCACTGTGTCTTGCAGAGTGCTAACTAGACCTAGTCCTGACTTCTCATTTAATTTCCTTGTGCCACCCACTGTGGAGTCAAAGACAAAACCCTTTTCCCTCCCCATTCTGACTATCTCTGAAATGTCCAATTCTAGGTTCCCAGTACCAATTGATTCTCTGCACACCAGTCCTACTGAGAACATTGTTGTCCAGTGCCAGAACGGACGCGTCACCCTTGATGGTGAGTTGATGGGCACCACTCAACTTTTACCTAGCCAAATCTGTGCTTTCAGGGGCATGCTCACCAGGTCAACAAGCAGGGCCAGTGACCAGGCCGATACAGCAACCCCTAGATTGTTTAATTATTATTGGCATATACAATTGGATAACCTAAATGGAACTCCTTATGACCCTGCAGAAGATATACCAGGCCCCCTAGGGACACCAGATTTCCGGGGCAAAGTCTTTGGCGTGGCCAGCCAGAGAAATCCTGATGCCACGACTAGGGCACATGAAGCAAAGATAGACACAACATCTGGCCGCTTCACTCCAAAATTAGGCTCACTAGAGATTTCCACTGAGTCTGGAGATTTTGATCAAAACCAACCAACAAGATTCACCCCAGTTGGCATTGGGGTTGACCATGAGGCAGATTTTCAACAATGGACTCTTCCCGACTACGCTGGTCAGTTCACCCACAACATGAACTTAGCCCCAGCTGTTGCCCCCAACTTCCCCGGTGAGCAGCTCCTTTTCTTTCGCTCACAGTTGCCATCTTCTGGCGGTCGGTCCAACGGGATTCTAGACTGCCTGGTCCCCCAAGAATGGGTACAGCACTTCTATCAAGAATCAGCCCCCTCCCAAACTCAAGTGGCCCTGGTTAGGTATGTCAACCCTGACACTGGTAGAGTGTTATTTGAGGCCAAGCTGCACAAATTAGGTTTCATGACTATAGCCAAGAGTGGTGACTCTCCAATAACTGTCCCCCCAAATGGATACTTCAGGTTTGAATCTTGGGTGAACCCCTTTTATACACTTGCCCCCATGGGAACTGGGAATGGGCGTAGAAGGATTCAATAA

>KM198553|II.21/II.3|2010|VN|30381/2010

ATGAAGATGGCGTCGAATGACGCCGCTCCATCTAATGATGGTGCCGCCGGCCTCGTCCCAGAGATCAACAGTGAGGCAATGGCGCTAGAGCCAGTGGCGGGTGCAGCGATAGCAGCACCCCTCACTGGTCAGCAAAATATAATTGATCCCTGGATTATGAATAATTTTGTGCAAGCACCTGGTGGTGAGTTCACAGTATCCCCTAGAAATTCCCCTGGTGAAGTTCTTCTCAATTTGGAATTGGGTCCAGAAATAAATCCCTACTTGGCCCATCTTGCTAGAATGTATAATGGTTATGCAGGTGGATTTGAAGTGCAGGTGGTCCTAGCTGGAAATGCGTTTACAGCAGGAAAGATAATCTTTGCAGCTATTCCCCCTAATTTTCCAATTGATAATCTAAGTGCAGCACAGATCACAATGTGTCCACATGTGATTGTGGATGTTAGACAGCTGGAACCAGTCAACCTCCCGATGCCTGACGTTCGCAATAACTTTTTCCATTACAATCAGGGGTCTGATTCGAGATTGCGCCTAATTGCAATGCTATATACACCTCTTAGGGCAAATAACTCTGGGGATGATGTTTTCACTGTGTCTTGCAGAGTGCTAACTAGACCTAGTCCTGACTTCTCATTTAATTTCCTTGTGCCACCCACTGTGGAGTCAAAGACAAAACCCTTTTCCCTCCCCATTCTGACTATCTCTGAAATGTCCAATTCTAGGTTCCCAGTACCAATTGATTCTCTGCACACCAGCCCTACTGAGAACATTGTCGTCCAGTGCCAGAATGGACGCGTCACCCTTGATGGTGAGTTGATGGGCACCACCCAACTTTTACCTAGCCAAATCTGTGCTTTCAGGGGCATGCTCACCAGATCAACAAGCAGGGCCAGTGACCAGGCCGATACAGCAACCCCTAGATTGTTTAATTATTATTGGCATATACAATTGGATAACCTAAATGGAACTCCTTATGACCCTGCAGAAGATATACCAGGCCCCCTAGGGACACCAGATTTCCGGGGCAAAGTCTTTGGCGTGGCCAGCCAGAGAAATCCTGATGCCACGACTAGGGCACATGAAGCAAAGATAGACACAACATCTGGCCGCTTCACCCCAAAATTAGGCTCACTAGAGATTTCCACTGAGTCTGGAGATTTTGATCAAAACCAACCAACAAGATTCACCCCAGTTGGCATTGGGGTTGACCATGAGGCAGATTTTCAACAATGGACTCTTCCCGACTATGCTGGCCAGTTCACCCACAACATGAACTTAGCCCCAGCTGTTGCTCCCAACTTCCCTGGTGAGCAGCTCCTTTTCTTCCGCTCACAGTTGCCATCTTCTGGTGGGCGGTCCAACGGGATTCTAGACTGCCTGGTCCCCCAAGAATGGGTACAGCACTTCTACCAAGAATCAGCCCCCTCCCAAACTCAAGTGGCCCTGGTTAGGTATGTCAACCCTGACACTGGTAGAGTGTTATTTGAGGCCAAGCTGCACAAATTAGGTTTCATGACTATAGCCAAGAGTGGTGACTCTCCAATAACTGTCCCCCCAAATGGATACTTCAGGTTTGAATCTTGGGTGAACCCCTTTTATACACTTGCCCCCATGGGAACTGGGAATGGGCGTAGAAGGATTCAATAA

>KM198554|II.21/II.3|2011|VN|C2H-48/2011

ATGAAGATGGCGTCGAATGACGCCGCTCCATCTAATGATGGTGCCGCCGGCCTCGTCCCAGAGATCAACAGTGAGGCAATGGCGCTAGAGCCAGTGGCGGGTGCAGCGATAGCAGCACCCCTCACTGGTCAGCAAAATATAATTGATCCCTGGATTATGAATAATTTTGTGCAAGCACCTGGTGGTGAGTTCACAGTATCCCCTAGAAATTCCCCTGGTGAAGTTCTTCTCAATTTGGAATTGGGTCCAGAAATAAATCCCTACTTGGCCCATCTTGCTAGAATGTATAATGGTTATGCAGGTGGATTTGAAGTGCAGGTGGTCCTAGCTGGAAATGCGTTTACAGCAGGAAAGATAATCTTTGCAGCTATTCCCCCTAATTTTCCAATTGATAATCTAAGTGCAGCACAGATCACAATGTGCCCACATGTGATTGTGGATGTTAGACAGCTGGAACCAGTCAACCTCCCGATGCCTGACGTTCGCAATAACTTTTTCCATTACAATCAGGGGTCTGATTCGAGATTGCGCCTAATTGCAATGCTGTATACACCTCTTAGGGCAAATAACTCTGGGGATGATGTTTTCACTGTGTCTTGCAGAGTGCTAACTAGACCTAGTCCTGACTTCTCATTTAATTTCCTTGTGCCACCCACTGTGGAGTCAAAGACAAAACCCTTTTCCCTCCCCATTCTGACTATCTCTGAAATGTCCAATTCTAGGTTCCCAGTACCAATTGATTCTCTGCACACCAGTCCTACTGAGAACATTGTTGTCCAGTGCCAGAACGGACGCGTCACCCTTGATGGTGAGTTGATGGGCACCACTCAACTTTTACCTAGCCAAATCTGTGCTTTCAGGGGCATGCTCACCAGGTCAACAAGCAGGGCCAGTGACCAGGCCGATACAGCAACCCCTAGATTGTTTAATTATTATTGGCATATACAATTGGATAACCTAAATGGAACTCCTTATGACCCTGCAGAAGATATACCAGGCCCCCTAGGGACACCAGATTTCCGGGGCAAAGTCTTTGGCGTGGCCAGCCAGAGAAATCCTGATGCCACGACTAGGGCACATGAAGCAAAGATAGACACAACATCTGGCCGCTTCACTCCAAAATTAGGCTCACTAGAGATTTCCACTGAGTCTGGAGATTTTGATCAAAACCAACCAACAAGATTCACCCCAGTTGGCATTGGGGTTGACCATGAGGCAGATTTTCAACAATGGACTCTTCCCGACTACGCTGGTCAGTTCACCCACAACATGAACTTAGCCCCAGCTGTTGCCCCCAACTTCCCCGGTGAGCAGCTCCTTTTCTTTCGCTCACAGTTGCCATCTTCTGGCGGTCGGTCCAACGGGATTCTAGACTGCCTGGTCCCCCAAGAATGGGTACAGCACTTCTATCAAGAATCAGCCCCCTCCCAAACTCAAGTGGCCCTGGTTAGGTATGTCAACCCTGACACTGGTAGAGTGTTATTTGAGGCCAAGCTGCACAAATTAGGTTTCATGACTATAGCCAAGAGTGGTGACTCTCCAATAACTGTCCCCCCAAATGGATACTTCAGGTTTGAATCTTGGGTGAACCCCTTTTATACACTTGCCCCCATGGGAACTGGGAATGGGCGTAGAAGGATTCAATAA

>KM198561|II.21/II.3|2009|VN|30303/2009

ATGAAGATGGCGTCGAATGACGCCGCTCCATCTAATGATGGTGCCGCCGGCCTCGTCCCAGAGATCAACAGTGAGGCAATGGCGCTAGAGCCAGTGGCGGGTGCAGCGATAGCAGCACCCCTCACTGGTCAGCAAAATATAATTGATCCCTGGATTATGAATAATTTTGTGCAAGCACCTGGTGGTGAGTTCACAGTATCCCCTAGAAATTCCCCTGGTGAAGTTCTTCTCAATTTGGAATTGGGTCCAGAAATAAATCCCTACTTGGCCCATCTTGCTAGAATGTATAATGGTTATGCAGGTGGATTTGAAGTGCAGGTGGTCCTAGCTGGAAATGCGTTTACAGCAGGAAAGATAATCTTTGCAGCTATTCCCCCTAATTTTCCAATTGATAATCTAAGTGCAGCACAGATCACAATGTGTCCACATGTGATTGTGGATGTTAGACAGCTGGAACCAGTCAACCTCCCGATGCCTGACGTTCGCAATAACTTTTTCCATTACAATCAGGGGTCTGATTCGAGATTGCGCCTAATTGCAATGCTATATACACCTCTTAGGGCAAATAACTCTGGGGATGATGTTTTCACTGTGTCTTGCAGAGTGCTAACTAGACCTAGTCCTGACTTCTCATTTAATTTCCTTGTGCCACCCACTGTGGAGTCAAAGACAAAACCCTTTTCCCTCCCCATTCTGACTATCTCTGAAATGTCCAATTCTAGGTTCCCAGTACCAATTGATTCTCTGCACACCAGCCCTACTGAGAACATTGTTGTCCAGTGCCAGAATGGACGCGTCACCCTTGATGGTGAGTTGATGGGCACCACCCAACTTTTACCTAGCCAAATCTGTGCTTTCAGGGGCATGCTCACCAGATCAACAAGCAGGGCCAGTGACCAGGCCGATACAGCAACCCCTAGATTGTTTAATTATTATTGGCATATACAATTGGATAACCTAAATGGAACTCCTTATGACCCTGCAGAAGATATACCAGGCCCCCTAGGGACACCAGATTTCCGGGGCAAAGTCTTTGGCGTGGCCAGCCAGAGAAATCCTGATGCCACGACTAGGGCACATGAAGCAAAGATAGACACAACATCTGGCCGCTTCACCCCAAAATTAGGCTCACTAGAGATTTCCACTGAGTCTGGAGATTTTGATCAAAACCAACCAACAAGATTCACCCCAGTTGGCATTGGGGTTGACCATGAGGCAGATTTTCAACAATGGACTCTTCCCGACTATGCTGGCCAGTTCACCCACAACATGAACTTAGCCCCAGCTGTTGCTCCCAACTTCCCTGGTGAGCAGCTCCTTTTCTTCCGCTCACAGTTGCCATCTTCTGGTGGGCGGTCCAACGGGATTCTAGACTGCCTGGTCCCCCAAGAATGGGTACAGCACTTCTACCAAGAATCAGCCCCCTCCCAAACTCAAGTGGCCCTGGTTAGGTATGTCAACCCTGACACTGGTAGAGTGTTATTTGAGGCCAAGCTGCACAAATTAGGTTTCATGACTATAGCCAAGAGTGGTGACTCTCCAATAACTGTCCCCCCAAATGGATACTTCAGGTTTGAATCTTGGGTGAACCCCTTTTATACACTTGCCCCCATGGGAACTGGGAATGGGCGTAGAAGGATTCAATAA

>KM198563|II.21/II.3|2010|VN|C2365/2010

ATGAAGATGGCGTCGAATGACGCCGCTCCATCTAATGATGGTGCCGCCGGCCTCGTCCCAGAGATCAACAGTGAGGCAATGGCGCTAGAGCCAGTGGCGGGTGCAGCGATAGCAGCACCCCTCACCGGTCAGCAAAATATAATTGATCCCTGGATTATGAATAATTTTGTGCAAGCACCTGGTGGTGAGTTCACAGTATCCCCTAGAAATTCCCCTGGTGAAGTTCTTCTCAATTTGGAATTGGGTCCAGAAATAAATCCCTATTTGGCCCATCTTGCTAGAATGTATAATGGTTATGCAGGTGGATTTGAAGTGCAGGTGGTCCTAGCTGGAAATGCGTTTACAGCAGGAAAGATAATCTTTGCAGCTATTCCCCCTAATTTTCCAATTGATAATCTAAGTGCAGCACAGATCACAATGTGTCCACATGTGATTGTGGATGTTAGGCAGCTGGAACCAGTCAACCTCCCGATGCCTGACGTTCGCAACAACTTTTTCCATTACAATCAGGGGTCTGATTCGAGATTGCGCCTAATTGCAATGCTATATACACCTCTTAGGGCAAATAACTCTGGGGATGATGTTTTCACTGTGTCTTGTAGAGTGCTAACTAGACCTAGTCCTGACTTCTCATTTAATTTCCTTGTGCCACCCACTGTGGAGTCAAAGACAAAACCCTTTTCCCTCCCCATTTTGACTATCTCTGAAATGTCCAATTCTAGGTTCCCAGTACCAATTGATTCTCTGCACACCAGCCCTACTGAGAACATTGTTGTCCAGTGCCAGAATGGACGCGTCACCCTTGATGGTGAGTTGATGGGCACCACCCAACTTTTACCTAGCCAAATCTGTGCTTTCAGGGGCATGCTCACCAGATCAACAAGCAGGGCCAGTGACCAGGCCGATACAGTAACCCCTAGATTATTTAATTATTATTGGCATATACAATTGGATAACCTAAATGGAACTCCTTATGACCCTGCAGAAGATATACCAGGCCCCCTAGGGACACCAGATTTCCGGGGCAAAGTCTTTGGCGTGGCCAGCCAGAGAAATCCTGATGCCACGACTAGGGCACATGAAGCAAAGATAGACACAACATCTGGCCGCTTCACCCCAAAATTAGGCTCACTAGAGATTTCCACTGAGTCTGATGATTTTTATCAAAACCAACCAACAAGATTCACCCCAGTTGGCATTGGGGTTGACCATGAGGCAGACTTTCAACAATGGACTCTTCCCGACTATGCTGGCCAGTTCACCCACAACATGAACCTAGCCCCAGCTGTTGCTCCCAACTTCCCTGGTGAGCAGCTCCTTTTCTTCCGCTCACAGTTGCCATCTTCTGGTGGGCGGTCCAACGGGATTCTAGACTGCCTGGTCCCCCAAGAATGGGTACAGCACTTCTACCAAGAATCAGCCCCCTCCCAAACTCAAGTGGCCCTGGTTAGGTATGTCAACCCTGACACTGGTAGAGTGTTATTTGAGGCCAAGCTGCACAAATTAGGTTTCATGACTATAGCCAAAAATGGTGACTCTCCAATAACTGTCCCCCCAAATGGATACTTTAGGTTTGAATCTTGGGTGAACCCCTTTTATACACTTGCCCCCATGGGAACTGGGAATGGGCGTAGAAGGATTCAATAA

>KM198572|II.21/II.3|2010|VN|20370/2010

ATGAAGATGGCGTCGAATGACGCCGCTCCATCTAATGATGGTGCCGCCGGCCTCGTCCCAGAGATCAACAGTGAGGCAATGGCGCTAGAGCCAGTGGCGGGTGCAGCGATAGCAGCACCCCTCACTGGTCAGCAAAATATAATTGATCCCTGGATTATGAATAATTTTGTGCAAGCACCTGGTGGTGAGTTCACAGTATCCCCTAGAAATTCCCCTGGTGAAGTTCTTCTCAATTTGGAATTGGGTCCAGAAATAAATCCCTACTTGGCCCATCTTGCTAGAATGTATAATGGTTATGCAGGTGGATTTGAAGTGCAGGTGGTCCTAGCTGGAAATGCGTTTACAGCAGGAAAGATAATCTTTGCAGCTATTCCCCCTAATTTTCCAATTGATAATCTAAGTGCAGCACAGATCACAATGTGTCCACATGTGATTGTGGATGTTAGACAGCTGGAACCAGTCAACCTCCCGATGCCTGACGTTCGCAATAACTTTTTCCATTACAATCAGGGGTCTGATTCGAGATTGCGCCTAATTGCAATGCTATACACACCTCTTAGGGCAAATAACTCTGGGGATGATGTTTTCACTGTGTCTTGCAGAGTGCTAACTAGACCTAGTCCTGACTTCTCATTTAATTTCCTTGTGCCACCCACTGTGGAGTCAAAGACAAAACCCTTTTCCCTCCCCATTCTGACTATCTCTGAAATGTCCAATTCTAGGTTCCCAGTACCAATTGATTCTCTGCACACCAGCCCTACTGAGAACATTGTTGTCCAGTGCCAGAATGGACGCGTCACCCTTGATGGTGAGTTGATGGGCACCACCCAACTTTTACCTAGCCAAATCTGTGCTTTCAGGGGCATGCTCACCAGATCAACAAGCAGGGCCAGTGACCAGGCCGATACAGCAACCCCTAGATTGTTTAATTATTATTGGCATATACAATTGGATAACCTAAATGGAACTCCTTATGACCCTGCAGAAGATATACCAGGCCCCCTAGGGACACCAGATTTCCGGGGCAAAGTCTTTGGCGTGGCCAGCCAGAGAAATCCTGATGCCACGACTAGGGCACATGAAGCAAAGATAGACACAACATCTGGCCGCTTCACCCCAAAATTAGGCTCACTAGAGATTTCCACTGAGTCTGGAGATTTTGATCAAAACCAACCAACAAGATTCACCCCAGTTGGCATTGGGGTTGACCATGAGGCAGATTTTCAACAATGGACTCTTCCCGACTATGCTGGCCAGTTCACCCACAACATGAACTTAGCCCCAGCTGTTGCTCCCAACTTCCCTGGTGAGCAGCTCCTTTTCTTCCGCTCACAGTTGCCATCTTCTGGTGGGCGGTCCAACGGGATTCTAGACTGCCTGGTCCCCCAAGAATGGGTACAGCACTTCTACCAAGAATCAGCCCCCTCCCAAACTCAAGTGGCCCTGGTTAGGTATGTCAACCCTGACACTGGTAGAGTGTTATTTGAGGCCAAGCTGCACAAATTAGGTTTCATGACTATAGCCAAGAGTGGTGACTCTCCAATAACTGTCCCCCCAAATGGATACTTCAGGTTTGAATCTTGGGTGAACCCCTTTTATACACTTGCCCCCATGGGAACTGGGAATGGGCGTAGAAGGATCCAATAA

>KM198573|II.21/II.3|2011|VN|C2H-25/2011

ATGAAGATGGCGTCGAATGACGCCGCTCCATCTAATGATGGTGCCGCCGGCCTCGTCCCAGAGATCAACAGTGAGGCAATGGCGCTAGAGCCAGTGGCGGGTGCAGCGATAGCAGCACCCCTCACTGGTCAGCAAAATATAATTGATCCCTGGATTATGAATAATTTTGTGCAAGCACCTGGTGGTGAGTTCACAGTATCCCCTAGAAATTCCCCTGGTGAAGTTCTTCTCAATTTGGAATTGGGTCCAGAAATAAATCCCTACTTGGCCCATCTTGCTAGAATGTATAATGGTTATGCAGGTGGATTTGAAGTGCAGGTGGTCCTAGCTGGAAATGCGTTTACAGCAGGAAAGATAATCTTTGCAGCTATTCCCCCTAATTTTCCAATTGATAATCTAAGTGCAGCACAGATCACAATGTGCCCACATGTGATTGTGGATGTTAGACAGCTGGAACCAGTCAACCTCCCGATGCCTGACGTTCGCAATAACTTTTTCCATTACAATCAGGGGTCTGATTCGAGATTGCGCCTAATTGCAATGCTGTATACACCTCTTAGGGCAAATAACTCTGGGGATGATGTTTTCACTGTGTCTTGCAGAGTGCTAACTAGACCTAGTCCTGACTTCTCATTTAATTTCCTTGTGCCACCCACTGTGGAGTCAAAGACAAAACCCTTTTCCCTCCCCATTCTGACTATCTCTGAAATGTCCAATTCTAGGTTCCCAGTACCAATTGATTCTCTGCACACCAGTCCTACTGAGAACATTGTTGTCCAGTGCCAGAACGGACGCGTCACCCTTGATGGTGAGTTGATGGGCACCACTCAACTTTTACCTAGCCAAATCTGTGCTTTCAGGGGCATGCTCACCAGGTCAACAAGCAGGGCCAGTGACCAGGCCGATACAGCAACCCCTAGATTGTTTAATTATTATTGGCATATACAATTGGATAACCTAAATGGAACTCCTTATGACCCTGCAGAAGATATACCAGGCCCCCTAGGGACACCAGATTTCCGGGGCAAAGTCTTTGGCGTGGCCAGCCAGAGAAATCCTGATGCCACGACTAGGGCACATGAAGCAAAGATAGACACAACATCTGGCCGCTTCACTCCAAAATTAGGCTCACTAGAGATTTCCACTGAGTCTGGAGATTTTGATCAAAACCAACCAACAAGATTCACCCCAGTTGGCATTGGGGTTGACCATGAGGCAGATTTTCAACAATGGACTCTTCCCGACTACGCTGGTCAGTTCACCCACAACATGAACTTAGCCCCAGCTGTTGCCCCCAACTTCCCCGGTGAGCAGCTCCTTTTCTTTCGCTCACAGTTGCCATCTTCTGGCGGTCGGTCCAACGGGATTCTAGACTGCCTGGTCCCCCAAGAATGGGTACAGCACTTCTATCAAGAATCAGCCCCCTCCCAAACTCAAGTGGCCCTGGTTAGGTATGTCAACCCTGACACTGGTAGAGTGTTATTTGAGGCCAAGCTGCACAAATTAGGTTTCATGACTATAGCCAAGAGTGGTGACTCTCCAATAACTGTCCCCCCAAATGGATACTTCAGGTTTGAATCTTGGGTGAACCCCTTTTATACACTTGCCCCCATGGGAACTGGGAATGGGCGTAGAAGGATTCAATAA

>KM198583|II.21/II.3|2010|VN|20493/2010

ATGAAGATGGCGTCGAATGACGCCGCTCCATCTAATGATGGTGCCGCCGGCCTCGTCCCAGAGATCAACAGTGAGGCAATGGCGCTAGAGCCAGTGGCGGGTGCAGCGATAGCAGCACCCCTCACTGGTCAGCAAAATATAATTGATCCCTGGATTATGAACAATTTTGTGCAAGCACCTGGTGGTGAGTTCACAGTATCCCCTAGAAATTCCCCTGGTGAAGTTCTTCTCAATTTGGAATTGGGTCCAGAAATAAATCCCTACTTGGCCCATCTTGCTAGAATGTATAATGGTTATGCAGGTGGATTTGAAGTGCAGGTGGTCCTAGCTGGAAATGCGTTTACAGCAGGAAAGATAATCTTTGCAGCTATTCCCCCTAATTTTCCAATTGATAATCTAAGTGCAGCACAGATCACAATGTGTCCACATGTGATTGTGGATGTTAGACAGCTGGAACCAGTCAACCTCCCGATGCCTGACGTTCGCAATAACTTTTTCCATTACAATCAGGGGTCTGATTCGAGATTGCGCCTAATTGCAATGCTATATACACCTCTTAGGGCAAATAACTCTGGGGATGATGTTTTCACTGTGTCTTGCAGAGTGCTAACTAGACCTAGTCCTGACTTCTCATTTAATTTCCTTGTGCCACCCACTGTGGAGTCAAAGACAAAACCCTTTTCCCTCCCCATTCTGACTATCTCTGAAATGTCCAATTCTAGGTTCCCAGTACCAATTGATTCTCTGCACACCAGCCCTACTGAGAACATTGTTGTCCAGTGCCAGAATGGACGCGTCACCCTTGATGGTGAGTTGATGGGCACCACCCAACTTTTACCTAGCCAAATCTGTGCTTTCAGGGGCATGCTCACCAGATCAACAAGCAGGGCCAGTGACCAGGCCGATACAGCAACCCCTAGATTGTTTAATTATTATTGGCATATACAATTGGATAACCTAAATGGAACTCCTTATGACCCTGCAGAAGATATACCAGGCCCCCTAGGGACACCAGATTTCCGGGGCAAAGTCTTTGGCGTGGCCAGCCAGAGAAATCCTGATGCCACGACTAGGGCACATGAAGCAAAGATAGACACAACATCTGGCCGCTTCACCCCAAAATTAGGCTCACTAGAGATTTCCACTGAGTCTGGAGATTTTGATCAAAACCAACCAACAAGATTCACCCCAGTTGGCATTGGGGTTGACCATGAGGCAGATTTTCAACAATGGACTCTTCCCGACTATGCTGGCCAGTTCACCCACAACATGAACTTAGCCCCAGCTGTTGCTCCCAACTTCCCTGGTGAGCAGCTCCTTTTCTTCCGCTCACAGTTGCCATCTTCTGGTGGGCGGTCCAACGGGATTCTAGACTGCCTGGTCCCCCAAGAATGGGTACAGCACTTCTACCAAGAATCAGCCCCCTCCCAAACTCAAGTGGCCCTGGTTAGGTATGTCAACCCTGACACTGGTAGAGTGTTATTTGAGGCCAAGCTGCACAAATTAGGTTTCATGACTATAGCCAAGAGTGGTGACTCTCCAATAACTGTCCCCCCAAATGGATACTTCAGGTTTGAATCTTGGGTGAACCCCTTTTATACACTTGCCCCCATGGGAACTGGGAATGGGCGTAGAAGGATTCAATAA

>KM198586|II.21/II.3|2010|VN|30400/2010

ATGAAGATGGCGTCGAATGACGCCGCTCCATCTAATGATGGTGCCGCCGGCCTCGTCCCAGAGATCAACAGTGAGGCAATGGCGCTAGAGCCAGTGGCGGGTGCAGCGATAGCAGCACCCCTCACTGGTCAGCAAAATATAATTGATCCCTGGATTATGAATAATTTTGTGCAAGCACCTGGTGGTGAGTTCACAGTATCCCCTAGAAATTCCCCTGGTGAAGTTCTTCTCAATTTGGAATTGGGTCCAGAAATAAATCCCTACTTGGCCCATCTTGCTAGAATGTATAATGGTTATGCAGGTGGATTTGAAGTGCAGGTGGTCCTAGCTGGAAATGCGTTTACAGCAGGAAAGATAATCTTTGCAGCTATTCCCCCTAATTTTCCAATTGATAATCTAAGTGCAGCACAGATCACAATGTGTCCACATGTGATTGTGGATGTTAGACAGCTGGAACCAGTCAACCTCCCGATGCCTGACGTTCGCAATAACTTTTTCCATTACAATCAGGGGTCTGATTCGAGATTGCGCCTAATTGCAATGCTATATACACCTCTTAGGGCAAATAACTCTGGGGATGATGTTTTCACTGTGTCTTGCAGAGTGCTAACTAGACCTAGTCCTGACTTCTCATTTAATTTCCTTGTGCCACCCACTGTGGAGTCAAAGACAAAACCCTTTTCCCTCCCCATTCTGACTATCTCTGAAATGTCCAATTCTAGGTTCCCAGTACCAATTGATTCTCTGCACACCAGCCCTACTGAGAACATTGTTGTCCAGTGCCAGAATGGACGCGTCACCCTTGATGGTGAGTTGATGGGCACCACCCAACTTTTACCTAGCCAAATCTGTGCTTTCAGGGGCATGCTCACCAGATCAACAAGCAGGGCCAGTGACCAGGCCGATACAGCAACCCCTAGATTGTTTAATTATTATTGGCATATACAATTGGATAACCTAAATGGAACTCCTTATGACCCTGCAGAAGATATACCAGGCCCCCTAGGGACACCAGATTTCCGGGGCAAAGTCTTTGGCGTGGCCAGCCAGAGAAATCCTGATGCCACGACTAGGGCACATGAAGCAAAGATAGACACAACATCTGGCCGCTTCACCCCAAAATTAGGCTCACTAGAGATTTCCACTGAGTCTGGAGATTTTGATCAAAACCAACCAACAAGATTCACCCCAGTTGGCATTGGGGTTGACCATGAGGCAGATTTTCAACAATGGACTCTTCCCGACTATGCTGGCCAGTTCACCCACAACATGAACTTAGCCCCAGCTGTTGCTCCCAACTTCCCTGGTGAGCAGCTCCTTTTCTTCCGCTCACAGTTGCCATCTTCTGGTGGGCGGTCCAACGGGATTCTAGACTGCCTGGTCCCCCAAGAATGGGTACAGCACTTCTACCAAGAATCAGCCCCCTCCCAAACTCAAGTGGCCCTGGTTAGGTATGTCAACCCTGACACTGGTAGAGTGTTATTTGAGGCCAAGCTGCACAAATTAGGTTTCATGACTATAGCCAAGAGTGGTGACTCTCCAATAACTGTCCCCCCAAATGGATACTTCAGGTTTGAATCTTGGGTGAACCCCTTTTATACACTTGCCCCCATGGGAACTGGGAATGGGCGTAGAAGGATTCAATAA

>KM198590|II.21/II.3|2011|VN|C2H-39/2011

ATGAAGATGGCGTCGAATGACGCCGCTCCATCTAATGATGGTGCCGCCGGCCTCGTCCCAGAGATCAACAGTGAGGCAATGGCGCTAGAGCCAGTGGCGGGTGCAGCGATAGCAGCACCCCTCACTGGTCAGCAAAATATAATTGATCCCTGGATTATGAATAATTTTGTGCAAGCACCTGGTGGTGAATTCACAGTATCCCCTAGAAATTCCCCTGGTGAAGTTCTTCTCAATTTGGAATTGGGTCCAGAAATAAATCCCTACTTGGCCCATCTTGCTAGAATGTATAATGGTTATGCAGGTGGATTTGAAGTGCAGGTGGTCCTAGCTGGAAATGCGTTTACAGCAGGAAAGATAATCTTTGCAGCCATTCCCCCTAATTTTCCAATTGATAATCTAAGTGCAGCACAGATCACAATGTGCCCACATGTGATTGTGGATGTTAGACAGCTGGAACCAGTCAACCTCCCGATGCCTGACGTTCGCAATAACTTTTTCCATTACAATCAGGGGTCTGATTCGAGATTGCGCCTAATTGCAATGCTGTATACACCTCTTAGGGCAAATAACTCTGGGGATGATGTTTTCACTGTGTCTTGCAGAGTGCTAACTAGACCTAGTCCTGACTTCTCATTTAATTTCCTTGTGCCACCCACTGTGGAGTCAAAGACAAAACCCTTTTCCCTCCCCATTCTAACTATCTCTGAAATGTCCAATTCTAGGTTCCCAGTACCAATTGATTCTCTGCACACCAGTCCTACTGAGAACATTGTTGTCCAGTGCCAGAACGGACGCGTCACCCTTGATGGTGAGTTGATGGGCACCACTCAACTTTTACCTAGCCAAATCTGTGCTTTCAGGGGCATGCTAACCAGATCAACAAGCAGGGCCAGTGACCAGGCCGATACAGCAACCCCTAGATTGTTTAATTATTATTGGCATATACAATTGGATAACCTAAATGGAACTCCTTATGACCCTGCAGAAGATATACCAGGCCCCCTAGGGACACCAGATTTCCGGGGCAAAGTCTTTGGCGTGGCCAGCCAGAGAAATCCTGATGCCACGACTAGGGCACATGAAGCAAAGATAGACACAACATCTGGCCGCTTCACTCCAAAATTAGGCTCACTAGAGATTTCCACTGAATCTGGAGATTTTGATCAAAGCCAACCAACAAGATTCACCCCAGTTGGCATTGGGGTTGACCATGAGGCAGATTTTCAACAATGGACTCTTCCCGACTACGCTGGCCAGTTCACCCACAACATGAACTTAGCCCCAGCTGTTGCTCCCAACTTCCCCGGTGAGCAGCTCCTTTTCTTTCGCTCACAGTTGCCATCTTCTGGCGGGCGGTCCAACGGGATTCTAGACTGCCTGGTCCCCCAAGAATGGGTGCAGCACTTCTATCAAGAATCGGCCCCCTCCCAAACTCAAGTGGCCCTGGTTAGGTATGTCAACCCTGACACTGGTAGAGTGTTATTTGAGGCCAAGCTGCACAAATTAGGTTTCATGACTATAGCCAAGAGTGGTGACTCTCCAATAACTGTCCCCCCAAATGGATACTTCAGGTTTGAATCTTGGGTGAACCCCTTTTATACGCTTGCCCCCATGGGAACTGGGAATGGGCGTAGAAGGATTCAATAA

>KM289170|II.16/II.3|2014|DE|Muenster706

ATGAAGATGGCGTCGAATGACGCTGCTCCATCTAATGATGGTGCTGCCGGCCTCGTACCAGAGATCACTAATGAGGCAATGGCGCTAGATCCAGTGGCGGGTGCAGCGATAGCAGCACCTCTTACTGGTCAGCAAAATATAATAGATCCCTGGATTATGAATAATTTTGTGCAAGCACCTGGTGGTGAGTTTACAGTGTCACCTAGGAACTCCCCTGGTGAAGTACTCCTAAATTTAGAATTAGGCCCAGAAATAAATCCCTATCTGGCGCACCTTGCTAGGATGTACAACGGTTATGCAGGTGGGTTTGAGGTGCAGGTGGTCCTGGCTGGAAATGCGTTTACAGCAGGGAAAGTGATCTTTGCAGCCATACCCCCCAACTTCCCAACTGACAATTTGAGTGCAGCGCAGATTACAATGTGTCCTCATGTGATTGTGGATGTCAGGCAGTTGGAACCAATCAACCTCCCGATGCCTGATGTCCGCAACAATTTCTTCCACTACAATCAAGGTTCTGATTCGAGGTTACGCCTAATTGCAATGTTGTATACACCCCTTAGGGCAAATAATTCTGGAGATGATGTTTTCACTGTGTCATGTAGAGTATTGACTAGGCCCAGTCCTGATTTCTCATTCAATTTCCTTGTTCCGCCCACTGTAGAATCAAAGACAAAACCTTTTACCCTCCCTATTTTGACCATCTCTGAGATGTCTAATTCTAGATTTCCAGTGCCAATTGACTCTCTGCACACCAGTCCAACTGATAACATTGTTGTCCAGTGTCAAAATGGGCGCGTCACCCTTGATGGTGAGCTAATGGGCACCACCCAACTCTTGCCGAGTCAGATATGTGCTTTTAGGGGTGTGCTCACCAGATCAACAAGCAGGGCCAGTGATCAAGCTGACACAGCAACCCCCAGGCTCTTCAACTACTACTGGCACATACAACTAGACAACCTAAATGGGACCCCGTATGATCCTGCAGAGGACATACCGGCTCCTCTAGGAACGCCAGACTTTCGGGGCAAAGTCTTTGGCGTAGCCAGCCAGAGAAACCCTGACAGCACAACAAGGGCACATGAGACAAAAGTGGACACAACATCTGGCCGCTTCACCCCAAAATTGGGCTCCCTAGAAATATCCACTGAATCTGGTGACTTTGACCAAAATCAACCAACAAGATTCACCCCAGTTGGCATTGGAGTTGACAATGAGGCAGATTTTCAACAATGGATCTTACCTGACTATTCCGGTCAGTTCACCCACAACATGAATCTGGCCCCAGCTGTCGCTCCCAATTTTCCCGGTGAACAGCTTCTTTTCTTCCGCTCACAGTTGCCATCCTCTGGCGGGCGGTCCAATGGTATTCTAGACTGCCTGGTCCCCCAGGAATGGGTCCAACACTTCTACCAGGAATCCGCCCCTGCCCAAACGCAGGTGGCTCTGGTTAGATATGTCAACCCTGATACTGGTAGGGTGTTATTTGAGGCTAAGCTGCATAAGCTAGGCTACATGACCATAGCTAAGAGTGGTGATTCTCCAA

>KM289172|II.16/II.3|2013|DE|PV_Voelk0018

ATGAAGATGGCGTCGAATGACGCTGCTCCATCTAATGATGGTGCCGCCGGCCTCGTACCAGAGATCAATAATGAGGCAATGGCGCTAGATCCAGTGGCGGGTGCAGCGATAGCAGCACCCCTTACTGGCCAGCAAAACATAATAGATCCCTGGATTATGAATAATTTTGTGCAAGCACCTGGTGGTGAGTTTACAGTGTCACCTAGGAACTCCCCTGGTGAAGTACTTCTAAATTTAGAATTAGGCCCAGAAATAAATCCCTATCTGGCGCATCTTGCTAGGATGTACAATGGTTATGCAGGTGGGTTTGAGGTGCAAGTGGTCCTGGCTGGAAATGCGTTTACAGCAGGGAAAGTGATCTTTGCAGCTATCCCCCCCAACTTCCCAACTGACAATTTGAGTGCAGCGCAGATTACAATGTGTCCTCATGTAATTGTGGATGTCAGGCAGTTGGAACCAATCAACCTCCCGATGCCTGATGTCCGCAACAACTTCTTTCATTATAATCAAGGTTCTGATTCAAGGTTACGCTTAATTGCAATGTTGTATACACCCCTTAGGGCAAATAATTCTGGAGATGATGTTTTCACTGTGTCATGTAGGGTATTGACTAGGCCCAGTCCTGACTTCTCATTCAATTTCCTCGTCCCGCCCACTGTGGAATCGAAGACAAAACCTTTTACCCTCCCTATTTTGACTATCTCTGAGATGTCTAATTCTAGATTTCCAGTGCCAATTGATTCTCTGCACACCAGTCCAACTGATAACATTGTTGTCCAGTGCCAAAATGGGCGCGTCACCCTTGACGGTGAGCTAATGGGCACCACCCAACTCTTGCCGAGTCAGATATGTGCTTTTAGGGGTGTGCTCACCAGATCAACAAGCAGGGCCAGTGACCAAGCTGACACAGCAACCCCCAGACTCTTCAATTACTACTGGCACATACAACTAGACAACCTAAATGGGACCCCGTATGATCCTGCAGAGGACATACCAGCTCCTCTGGGGACGCCAGACTTTCGGGGCAAAGTCTTTGGCGTAGCCAGCCAGAGAAACCCTGACAGTACAACAAGGGCACATGAGGCAAAGGTGGACACAACATCTGGCCGCTTCACCCCAAAATTGGGCTCCCTAGAAATATCCACTGAATCTGGTGACTTTGACCAAAACCAACCAACAAGATTCACCCCAGTTGGCATTGGAGTTGACAGTGAGGCAGATTTTCAACAATGGACCCTACCCGACTATTCCGGTCAGTTCACCCACAACATGAACCTGGCCCCAGCTGTCGCTCCCAATTTTCCCGGTGAACAGCTTCTTTTCTTCCGCTCACAGTTGCCATCCTCTGGCGGGCGGTCCAATGGCATTCTAGACTGCCTGGTCCCCCAGGAGTGGGTCCAACATTTCTACCAGGAATCCGCCCCTGCCCAAACGCAGGTGGCTCTGGTCAGGTATGTCAACCCTGATACTGGTAGGGTGTTATTTGAGGCTAAGCTGCATAAGCTAGGCTACATGACCATAGCTAAGAGTGGTGATTCTCCAATAACTGTACCCCCAAATGGATATTTTAGGTTTGAATCTTGGGTGAATCCCTTTTATACACTAGCCCCCATGGGAACTGGGAATG

>KP064097|-/II.3|2008|FR|E2419

ATGAAGATGGCGTCGAATGACGCCGCTCCATCTAATGATGGTGCCGCCGGCCTCGTCCCAGAGATCAACAATGAGGCAATGGCGCTAGAGCCGGTGGCGGGTGCAGCGATAGCAGCACCCCTCACTGGTCAGCAAAATATAATTGATCCCTGGATTATGAATAATTTTGTGCAAGCACCTGGTGGTGAGTTTACAGTATCCCCTAGAAATTCCCCTGGTGAAGTTCTTCTTAATTTGGAATTGGGTCCAGAAATAAATCCCTATTTGGCCCATCTTGCTAGAATGTACAATGGTTATGCAGGTGGATTTGAAGTGCAGGTGGTCCTAGCTGGAAATGCGTTTACAGCAGGAAAGATAATCTTTGCAGCTATTCCCCCTAATTTTCCAATTGATAATCTAAGTGCAGCACAGATCACAATGTGTCCACATGTGATTGTGGATGTCAGACAGCTGGAACCAGTCAACCTCCCGATGCCTGACGTTCGCAATAACTTCTTTCATTACAATCAAGGGTCTGATTCGAGATTGCGCCTAATTGCAATGCTATATACGCCTCTTAGGGCAAATAATTCTGGGGATGATGTTTTTACTGTGTCTTGCAGAGTGCTAACTAGACCTAGTCCTGACTTCTCATTTAATTTCCTTGTACCACCTACTGTGGAGTCAAAGACAAAACCCTTTTCCCTCCCTATTCTGACTATCTCTGAAATGTCCAATTCTAGGTTCCCAGTACCAATTGATTCTCTGCACACCAGCCCTACTGAGAACATTGTTGTCCAGTGTCAGAATGGACGCGTCACCCTTGATGGTGAGTTGATGGGCACCACCCAACTCTTACCTAGCCAAATCTGTACTTTCAGGGGCGTGCTCACCAGATCAACAAGCAGGGCCAGTGACCAGGCCGATACAGCAACCCCTAGATTGTTTAATTATTATTGGCATATACAATTGGATAATCTAAATGGAACTCCTTATGACCCTGCAGAAGATATACCAGGCCCCCTAGGGACACCAGATTTCCGGGGCAAAGTCTTTGGCGTGGCCAGCCAGAGAAATCCTGATAGCACGACTAGGGCACATGAAGCAAAGATAGACACAACATCTGGCCGTTTCACCCCAAAACTAGGCTCATTAGAGATTTCCACTGAGTCTGATGATTTTGATCAAAACAAACCAACAAGATTCACCCCAGTTGGCATTGGGGTTGACCATGAGGCAGACTTTCAACAATGGGCTCTTCCCGACTATGCTGGCCAGTTCACCCACAACATGAACTTAGCCCCAGCTGTTGCTCCCAACTTCCCTGGTGAGCAGCTCCTTTTCTTCCGCTCACAGTTACCATCTTCTGGTGGGCGATCCAACGGGATTCTAGACTGCCTGGTCCCCCAAGAGTGGGTACAGCATTTCTACCAAGAATCAGCCCCCTCCCAATCTCAAGTGGCCCTGGTTAGGTATATCAACCCTGACACTGGCAGAGTGTTATTTGAGGCCAAGCTGCACAAATTAGGTTTCATAACTATAGCCAAGAATGGTGACTCTCCAATAACTGTCCCTCCAAATGGATACTTTAGGTTTGAATCTTGGGTGAACCCCTTTTATACACTTGCCCCCATGGGAACTGGGAATGGGCGTAGAAGGATTCAATAA

>LN854569|II.21/II.3|2014|NL|GII.21/Groningen

ATGAAGATGGCGTCGAATGACGCCGCTCCATCTAATGATGGTGCCGCCGGCCTCGTCCCAGAGATCAACAATGAGGCAATGGCGCTAGAGCCAGTGGCGGGTGCAGCGATAGCAGCACCCCTCACTGGTCAGCAAAATATAATTGATCCCTGGATTATGAATAATTTTGTGCAAGCACCTGGTGGTGAGTTTACAGTGTCCCCCAGAAATTCCCCTGGTGAAGTTCTTCTTAATTTGGAATTGGGCCCAGAAATAAATCCCTATTTGGCCCATCTTGCTAGAATGTATAATGGTTATGCAGGTGGATTTGAAGTGCAGGTGGTCCTAGCTGGAAATGCGTTTACAGCAGGAAAGATAATCTTTGCAGCTATTCCCCCCAACTTTCCAATTGACAATTTAAGTGCAGCACAGATCACTATGTGCCCACATGTGATTGTAGATGTCAGACAGTTGGAACCAGTCAACCTCCCGATGCCTGACGTTCGCAATAACTTCTTTCATTATAATCAAGGGTCTGATTCGAGGTTACGCCTAATTGCAATGCTATATACACCTCTTAGAGCAAACAATTCTGGGGATGATGTTTTTACTGTGTCTTGTAGAGTGCTAACTAGACCTAGTCCTGACTTCTCATTCAATTTCCTTGTGCCACCTACTGTGGAGTCAAAGACAAAACCCTTCTCCCTCCCTATCCTGACTATTTCTGAAATGTCTAATTCTAGGTTCCCAGTACCAATTGATTCTCTGCACACCAGTCCTACTGAGAATATTGTTGTTCAGTGCCAAAATGGGCGCGTCACCCTTGATGGTGAGTTGATGGGCACCACCCAACTCTTGCCTAGCCAAATCTGTGCTTTCAGGGGAGTTCTCACCAGATCAACAAGCAGGGCCAGTGACCAGGCCGATACAGCAACCCCTAGATTGTTTAATTATTATTGGCATATACAATTGGATAATCTAAATGGAACCCCTTATGATCCTGCAGAAGACATACCAGGCCCCCTAGGGACACCAGATTTCCGTGGCAAAGTCTTTGGCGTGGCCAGCCAGAGAAATCTTGATACCACGACTAGGGCACATGAAGCAAAGATAGACACCACATCTGGCCGCTTCACCCCAAAGCTAGGCTCATTAGAAATATCCACTGAATCTAGTGATTTTGATCAAAGCCAACCAACAAGATTCACCCCAGTTGGCATTGGAGTTGACCATGAGGCAGACTTTCAACAATGGACCCTACCCGACTACGCTGGTCAGTTCACACACAACATGAACTTAGCACCAGCTGTTGCTCCCAACTTCCCTGGTGAGCAGCTCCTTTTCTTCCGCTCACATTTGCCATCTTCTGGTGGGCGATCTAACGGGATTCTAGACTGCCTGGTCCCCCAAGAATGGGTACAGCACTTCTACCAAGAGTCAGCCCCCTCTCAGTCTCAAGTGGCCCTGGTTAGGTATGTTAACCCTGACACTGGTAGAGTGTTATTTGAGGCCAAGCTACACAAATTGGGTTTCATGACTATAGCCAAGAATGGTGATTCTCCAATAATTGTTCCTCCAAATGGATACTTTAGGTTTGAATCTTGGGTGAACCCCTTTTACACACTTGCCCCCATGGGAACTGGGAATGGGCGTAGAAGGATTCAATAA

>L23830|II.3/II.3|1989|US|SRSV-OTH-25/89/J

ATGAAGATGGCGTCGAATGACGCTGCTCCATCTAATGATGGTGCCGCCGGCCTCGTCCCAGAGATCAACAATGAGGCAATGGCGCTAGATCCAGTGGCGGGTGCAGCGATAGCAGCACCCCTCACTGGCCAGCAAAATATAATTGATCCCTGGATTATGAATAACTTTGTGCAAGCACCTGGTGGTGAGTTTACAGTGTCACCTAGGAATTCCCCTGGTGAAGTGCTTCTTAATTTGGAATTAGGTCCAGAAATAAATCCCTATTTGGCTCATCTTGCTAGAATGTACAATGGTTATGCAGGTGGGTTTGAAGTGCAAGTGGTCCTGGCTGGAAATGCGTTTACAGCAGCAAAGGTGATCTTTGCAGCTATACCCCCTAACTTCCCTATTGACAATCTGAGCGCGGCACAGATCACAATGTGCCCGCATGTGATTGTGGATGTCAGGCAGTTGGAACCAATCAATCTTCCGATGCCTGATGTCCGCAACAATTTCTTTCATTATAATCAAGGGTCTGATTCAAGATTACGTTTAATCGCAATGCTGTATACACCTCTTAGGGCAAATAATTCTGGAGATGATGTTTTCACTGTGTCTTGTAGGGTGTTAACTAGGCCTAGCCCTGACTTCTCATTTAATTTTCTTGTCCCATCCACTATGGAATCAAAGACAAAACCTTTTACCCTCCCCATTTTAACCATCTCTGAAATGTCTAATTCCAGGTTTCCGGTGCCAATTGACTCTCTGCACACCAGCCCAACTGAGAGTATCGTTGTCCAGTGCCAAAATGGGCGCGTCACTCTTGACGGTGAGTTAATGGGCACCACCCAACTCTTACCGAACCAAATATGTGCCTTCAGGGGCACACTTACTAGATCAACAAACAGGGCCAGTGACCAAGCCGACACAGCAACCCCCAGGCTATTCAACCATCATTGGCACATACAATTGGATAATCTAAATGGAACCCCCTACGACCCTGCAGAGGACATACCAGCTCCTTTGGGTACACCAGACTTCCGGGGCAAGGTCTTTGGCGTAGCCGGCCAGAGAAACCCCGACAGCACAACAAGGGCACATGAAGCAAAAGTGGACACAACATCTGGCCGCTTCACCCCAAAATTGGGCTCCCTAGAAATAACCACTGAATCTGATGACTTTGACCCAAACCAGTCAACAAAATTCACCCCAGTTGGCATTGGAGTTGACAATGAGGCAGATTTTCAGCAATGGTCCTTACCTGACTATTCCGGTCAGTTTACTCACAACATGAACTTGGCCCCAGCTGTCGCCCCCAATTTTCCTGGTGAACAGCTTCTTTTCTTCCGATCACAGCTGCCATCTTCTGGTGGGCGGTCTAACGGGATTCTAGACTGCCTGGTCCCCCAGGAATGGGTTCAACACTTCTACCAGGAATCAGCCCCCGCCCAAACACAGGTGGCCCTGGTTAGATATGTCAACCCTGACACTGGTAGAGTGCTATTTGAGGCCAAGCTACACAAATTGGGTTTTATGACTATAGCAAAGAATGGTGACTCCCCAATAACTGTCCCTCCAAATGGGTATTTTAGATTTGAATCTTGGGTTAACCCCTTTTACACACTTGCCCCCATGGGAACTGGAAACGGGCGTAGAAGGATTCAA

>U46039|-/II.3|<1996|NZ|Auckland

ATGAAGATGGCGTCGAATGACGCTGCTCCATCTAATGATGGTGCCGCCGGCCTCGTCCCAGAGATCAACAATGAGGCAATGGCGCTAGAGCCAGTGGCGGGTGCAGCGATAGCAGCGCCCCTCACTGGCCAGCAAAATATAATTGATCCCTGGATTATGAATAATTTTGTGCAAGCACCTGGTGGTGAGTTTACAGTGTCACCTAGGAATTCCCCTGGTGAAGTGCTTCTTAATTTGGAGTTAGGTCCAGAAATAAACCCCTATTTGGCTCACCTTGCTAGAATGTACAATGGTTATGCAGGTGGATTTGAAGTGCAAGTGGTCCTAGCTGGAAATGCGTTTACAGCAGGAAAGGTTATCTTTGCAGCTATACCCCCTAACTTCCCTATTGACAATCTGAGCGCGGCACAAATTACAATGTGCCCGCACGTGATTGTGGATGTCAGGCAGTTGGAACCAATCAACCTCCCGATGCCTGACGTCCGCAACAATTTCTTTCATTATAATCAAGGTTCTGATTCAAGATTACGCTTGATTGCAATGTTGTTTACACCTCTTAGGGCAAATAATTCTGGAGATGATGTTTTCACTGTGTCTTGTAGGGTGTTAACTAGGCCTAGCCCTGATTTCTCATTTAATTTTCTTGTCCCACCCACTGTGGAATCAAAGATAAAACCTTTTACCCTCCCCATTTTACCCATCTCTGAAATGTCCAATTCCAGGTTTCCGGTGCCAATTGACTCTCTGCATACCAGCCCAACTGAGAATATAGTTGTCCAGTGCCAAAATGGACGCGTCACTCTTGACGGTGAGTTAATGGGCACCACCCAACTATTCCCGAGCCAAATATGTGCTTTCAGGGGCACACTCACTAGATCAACAAGCAGGGCCAGTGACCAAGCCGACACATCAACCCCTAGGCTATTCAACTATTATTGGCACATACAATTGGATAATCTAAATGGAACTCCCTACGACCCTGCAGAGGACATACCAGCTCCTTTGGGCACACCAGACTTCCAGGGCAAGGTCTTTGGTGTAGCCAGCCAGAGAAACCCCGACAGCACAACAAGGGCGCATGAAGCAAAAGTGGACACAACATCTGGCCGCTTCACCCCAAAATTGGGCTCCTTAGAGATAAGTACTGAATCTGATGACTTTGACTCAAACCAACCAACAAAATTCACCCCAGTTGGCATTGGAGTTGACAATGAGGCAGATTTTCAGCAATGGTCCTTACCCAACTATTCTGGTCAGTTTACTCATAATATGAACTTAGCCCCAGCTGTCGCCCCCATTTTTCCTGGTGAACAGATACTTTTCTTCCGATCACAGGTGCCATCCTCTGGTGGTCGGTCTAACGGGGTTCTAGACTGCCTGGTCCCCCAGGAATGGGTTCAACACTTTTACCAAGAATCAGCCCCCGCCCAAACACAGGTGGCCCTGGTTAGGTATGTCAATCCTGACACTGGTAGAGTGCTATTTGAGGCCAAGCTACACAAATTGGGTTTTATGACTATAGCAAAGAATGGTGACTCTCCAATAACTGTCCCTCCAAATGGATATTTTAGATTTGAATCTTGGGTTAACCCCTTTTATACACTTGCCCCCATGGGAACTGGAAACGGGCGCAGAAGGATTCAATAA

# 34 GII.3 ORF3 sequences (Table S6)

>AB039781|II.3/II.3|JP|1997|U18

ATGGCTGGAGCTTTTATAGCAGGATTGGCTGGTGACATGTTCACAAATACTGTGGGGTCTTTGGTTAATGCAGGGGCCGATGCTATTAATCAAAAAGTCGATTTTGAAAATAATAAATATTTGCAAAATGCTTCTTTTAATCATGATAAGGAGATGTTAAGTGCACAAATTGAGGCAACAAAGAGGCTGCAGGCTGACATGATTGCAATCAAACAAGGGGTCTTGACCGCTGGCGGCTTTTCCCCCACTGATGCAGCACGTGGAGCAATTAATGCTCCAATGACAAAAGTTTTGGATCGGAGTGAAACAAGGTACTGGGCACCAAACGCCGCCTTCACAACTTCAATGTCAGGTGGCTTCACAAGCCAAGTTGTGCACAGAACCACACCAGATCTTAAAACGAGCCAGGCCCCCAAATTCACACCCAGCAGTGGGTCTTCAGTGAGATCAAGCTCAACCCAACTCACCAACTTGAGCTCACACTCATCCGGGTCGTCCCGATCCAGTGGGTCTACGGTTGTCAGCTCGCTGCCGTCCTCCAGTAGGACTAGGGACTGGGTCAACCAACAAAATCTCAATTTGGAACCACACATGCCTGGGTCTCTCAGGACAGCTTTTGTCACTCCACCATCTAGCACAGCCTCTAGTTCAGGCACAGTCTCAACCGTGCCCAAAAATGTTTTGGACTCCTGGAC-ATCTGCGTTTAACACGCGCAGACAGCCGCTGTTTGC-ACACCTTCGTAGAAGGGGGGAGTCAAATGTTTAG

>AB039782|II.3/II.3|JP|1998|U201

ATGGCTGGAGCTTTTATAGCAGGATTGGCTGGTGACATGTTCACAAATACTGTGGGGTCTTTGGTTAATGCAGGGGCCAATGCTATTAATCAAAAAGTTGATTTTGAAAATAATAAATATTTGCAAAATGCTTCTTTTAATCATGATAAGGAGATGTTAAGTGCACAAATTGAGGCAACAAAGAGGCTGCAGGCTGACATGATTGCAATCAAACAAGGGGTCTTGACCGCTGGCGGCTTTTCCCCCACTGATGCAGCACGTGGGGCAATTAATGCTCCAATGACAAAAGTTTTAGATTGGAGTGGAACAAGGTACTGGGCACCAAACGCCGCCTCCACAACTTCAATGTCAGGTGGCTTCACAAGCCAAGTTGTGCACAGAACCACACCAGATTTTAAAACGAGCCAGGCCCCCAAATTCACACCCAGCAGTGGGTCTTCAGTGAGATCAAGCTCAACCCAACTCACCAACTTGAGCTCACACTCATCCGGGTCGTCCCGATCCAGCGGGTCTACGGTTGTCAGCTCGCTGCCGTCCTCCAGTAGGACTAGGGACTGGGTCAACCAACAAAATCTCAATTTGGAACCACACATGCCTGGATCTCTCAGGACAGCTTTTGTCACTCCACCATCTAGCACAGCCTCTAGTTCAGGCACAGTCTCAACCGTGCCCAAAAATGTTTTGGACTCCTGGAC-ATCTGCGTTTAACACGCGCAGACAGCCGCTGTTTGC-ACACCTTCGTAGAAGGGGGGAGTCAAATGTTTAG

>AB067541|II.3/II.3|JP|<2001|U18GII

ATGGCTGGAGCTTTTATAGCAGGATTGGCTGGTGACATGTTCACAAATACTGTGGGGTCTTTGGTTAATGCAGGGGCCGATGCTATTAATCAAAAAGTCGATTTTGAAAATAATAAATATTTGCAAAATGCTTCTTTTAATCATGATAAGGAGATGTTAAGTGCACAAATTGAGGCAACAAAGAGGCTGCAGGCTGACATGATTGCAATCAAACAAGGGGTCTTGACCGCTGGCGGCTTTTCCCCCACTGATGCAGCACGTGGAGCAATTAATGCTCCAATGACAAAAGTTTTGGATCGGAGTGAAACAAGGTACTGGGCACCAAACGCCGCCTTCACAACTTCAATGTCAGGTGGCTTCACAAGCCAAGTTGTGCACAGAACCACACCAGATCTTAAAACGAGCCAGGCCCCCAAATTCACACCCAGCAGTGGGTCTTCAGTGAGATCAAGCTCAACCCAACTCACCAACTTGAGCTCACACTCATCCGGGTCGTCCCGATCCAGTGGGTCTACGGTTGTCAGCTCGCTGCCGTCCTCCAGTAGGACTAGGGACTGGGTCAACCAACAAAATCTCAATTTGGAACCACACATGCCTGGGTCTCTCAGGACAGCTTTTGTCACTCCACCATCTAGCACAGCCTCTAGTTCAGGCACAGTCTCAACCGTGCCCAAAAATGTTTTGGACTCCTGGAC-ATCTGCGTTTAACACGCGCAGACAGCCGCTGTTTGC-ACACCTTCGTAGAAGGGGGGAGTCAAATGTTTAG

>AB067542|II.3/II.3|JP|<2001|U201GII

ATGGCTGGAGCTTTTATAGCAGGATTGGCTGGTGACATGTTCACAAATACTGTGGGGTCTTTGGTTAATGCAGGGGCCAATGCTATTAATCAAAAAGTTGATTTTGAAAATAATAAATATTTGCAAAATGCTTCTTTTAATCATGATAAGGAGATGTTAAGTGCACAAATTGAGGCAACAAAGAGGCTGCAGGCTGACATGATTGCAATCAAACAAGGGGTCTTGACCGCTGGCGGCTTTTCCCCCACTGATGCAGCACGTGGGGCAATTAATGCTCCAATGACAAAAGTTTTAGATTGGAGTGGAACAAGGTACTGGGCACCAAACGCCGCCTCCACAACTTCAATGTCAGGTGGCTTCACAAGCCAAGTTGTGCACAGAACCACACCAGATTTTAAAACGAGCCAGGCCCCCAAATTCACACCCAGCAGTGGGTCTTCAGTGAGATCAAGCTCAACCCAACTCACCAACTTGAGCTCACACTCATCCGGGTCGTCCCGATCCAGCGGGTCTACGGTTGTCAGCTCGCTGCCGTCCTCCAGTAGGACTAGGGACTGGGTCAACCAACAAAATCTCAATTTGGAACCACACATGCCTGGATCTCTCAGGACAGCTTTTGTCACTCCACCATCTAGCACAGCCTCTAGTTCAGGCACAGTCTCAACCGTGCCCAAAAATGTTTTGGACTCCTGGAC-ATCTGCGTTTAACACGCGCAGACAGCCGCTGTTTGC-ACACCTTCGTAGAAGGGGGGAGTCAAATGTTTAG

>AB190457|II.a/II.3|JP|<2004|SN2000JA

ATGGCTGGAGCTTTTATAGCAGGATTGGCTGGTGACATACTTACAAATACTGTAGGATCTCTAGTTAATGCAGGGGCTAATGCTATTAATCAAAAAGTTGATTTTGAAAACAATAAATATTTACAAAATGCATCCTTCAATCATGATAAGGAAATGTTAAATGCACAAGTTGAGGCAACAAAGAGGTTACAGGCTGACATGATTGCTATCAAACAAGGGGTTTTGACCGCTGGCGGCTTCTCCCCTACTGATGCAGCCCGCGGGGCAATTAACGCCCCCATGACAAAAGTCCTAGACTGGAATGGAACGAGGTACTGGGCACCAAATGCCACCTCCACAACTTTAATGTCGGGTGGCTTCACAAATCAAGCTGTGCACAGAACCACGCCAAATTTTAAAATGAACCAGACTCCCAAATCCACACCCAGCAGTGGGTCTTCAGTGAGGTCAAACTCAACCCAAATCACTAGCCTGAGCTCACACTCGTCCGGGTCGTCTCGATCCAGCGGGTCTACAGTTGTTAGCTCATTACCATCCTCTAACAGGACTAGGGACTGGGTCAACCAACAGAATTTTAATCTGGAACCACACATGCCTGGATCTCTTAGGACAGCTTTTGTTACTCCACCATCTAGTACAGCCTCTAGTTCAGGCACGGTCTCAACCGTGCCCAAAAATGTTTTGGACTCCTGGAC-ATCTGCGTTCAATACGCGCAGACAGCCGCTATTCGC-ACACCTTCGCAGAAGGGGGGAGTCAAATGTTTAG

>AB365435|II.21/II.3|US|2004|TCH04-577

ATGGCTGGAGCTTTTATAGCAGGATTAGCTGGTGATATACTCACAAATACTGTAGGATCTTTAGTTAATGCAGGGGCTAATGCCATTAATCAAAAAGTTGATTTTGAAAATAATAAATATTTACAAAATGCTTCCTTTAATCATGATAAGGAAATGTTAAATGCACAAATTGAGGCAACAAAGAGGTTACAGGCTGACATGATTGCTATCAAACAAGGGGTTTTGACCGCTGGCGGCTTCTCCCCCACTGATGCAGCCCGCGGGGCAATTAACGCCCCCATGACAAAAGTCCTAGATTGGAATGGGACGAGGTACTGGGCACCAAATGCCACCTCCACAACCTCGATGTCGGGTGGCTTCACAAATCAGGCTGTGCACAGAACCACGCCAAATTTTAAAACGAACCAGGCTTCCAAAACCACACCCAGCAGTGGGTCTTCAGTGAGGTCAAATTCAACCCAAGTCACTAGCCTGAGCTCATACTCGTCCGGGTCGTCTCGATCCAGCGGGTCTACAGTTGTTAGCTCATTACCATCCTCTAACAGGACTAGGGACTGGGTCAACCAACAAAATTTTAATTTGGAACCACACATGCCTGGGTCTCTTAGGACAGCTTTTGTCACTCCACCATCTAGTACAGCCTCTAGTTCAGGCACGGTCTCAACCGTGCCCAAAAATGTTTTGGACTCCTGGAC-ATCTGCGTTCAATACGCGCAGACAGCCGCTATTCGC-ACACCTTCGAAGAAGGGGGGAGTCAAAAGTTTAG

>AB629943|II.12/II.3|JP|2010|Tokyo/10-1105

ATGGCTGGAGCTTTTATAGCGGGATTAGCTGGTGATATATTCACAAATACTGTAGGATCTCTAGTTAATGCAGGGGCTAATGCCATTAATCAAAAAGTTGATTTTGAAAATAATAAATATTTGCAAAATGCTTCCTTTAATCATGATAAAGAAATGTTAAATGCACAAATTGAGGCAACAAAGAGGTTACAGGCTGATATGATTGCTATCAAACAGGGGGTTTTGACCGCTGGCGGCTTTTCCCCCACTGATGCAGCTCGCGGGGCAATTAATGCCCCCATGACAAAAGTCCTAGATTGGAATGGAACGAGGTACTGGGCACCAAATGCCACCTCTACAACCTCGATGTCGGGTGGCTTCACAAACAAGGCTGTGCACAGAACCGCGCCAAATTTTAAAACGAACCAGGCGCCCAAATCCACACCCAGCAGTGGGTCTTCAGTGAGGTCACTCTCAACCCAAATCACTAGTCTGAGCTCACACTCGTCCGGGTCGTCTCGATCCAGCGGGTCTACAGTTGTGAGCTCATTACCATCCTCCAACAGGACTAGGGACTGGGTCAATCAACAAAATTTCAATTTGGAACCACACATGCCTGGGTCCCTTAGGACAGCTTTTGTCACTCCACCATCTAGTACAGTCTCTAGTTCAGGCACTGTCTCAACCGTGCCCAAAA-TGTTTTGGACTCCTGGACAATCTGCGTTTAACACGCGCAGACAGCCGCTATTCGC-ACACCTTCGTAGAAGGGGGGAGTCAAATGTTTAG

>AB758450||/II.3|JP|2009|2009/Miyagi

ATGGCTGGAGCATTTATAGCAGGATTGGCTGGTGACATATTCACAAATACTGTAGGATCCCTAGTTAATGCAGGGGCTAATGCCATTAATCAAAAAGTTGATTTTGAAAACAATAAATATTTACAAAATGCATCCTTCAATCATGATAAGGAAATGTTAAATGCACAAATTGAGGCAACAAAGAGGTTGCAGGCTGACATGATTGCTATCAAGCAAGGGGTTTTGACCGCTGGCGGCTTCTCCCCTACTGATGCAGCCCGCGGGGCAATTAACGCCCCCATGACAAAAGTCCTAGATTGGAACGGAACGAGGTACTGGGCACCAAATGCTACCTCCACAACTTCAATGTCAGGTGGCTTCACTAATCAAGCTGTGCACAGAACCACGCCAAATTTTAAAACGAACCAGGCTTCCAAATCCACACCTAGTAGTGGGTCTTCAGTGGGGCCAAACTCAACCCAAAGCACCAGTCTGAGCTCACACTCGTCCGGGTCGTCCCGATCCAGCGGGTCTACAGTCGTTAGCTCACTACCATCCTCTAACAGGACTAGGGACTGGGTCAACCAACAGAATTTCAATTTGGAACCACACATGCCTGGATCTCTTAGGACAGCTTTTGTCACCCCACCATCTAGTACAGCCTCTAGCTCAGGCACGGTCTCAACCGTGCCCAAAAATGTTTTGGACTCCTGGAC-ATCTGCGTTCAACACGCGCAGACAGCCGCTATTTGC-ACACCTTCGCAGAAGGGGGGAGTCAAATGCTTAG

>AF190817|II.a/II.3|US|<2001|Arg320

ATGGCTGGAGCTTTTATAGCAGGATTGGCTGGTGACATGCTCGCAAATACTGTAGGATCTCTAGTTAGTGCAGGGGCCAATGCTATTAATCAAAAAGTTGATTTTGAAAATAATAAATATTTACAAAATGCATCCTTCAATCATGATAAGGAAATGTTAAATGCACAAATTGAGGCAACAAAGAGGTTACAGGCTGACATGATTGCTATCAAACAAGGGGTTTTGAC-GCTGGCGGCTTCTCCCCTACTGATGCAGCCCGTG--GCAATTAATGCCCCCATGACAAAAGTTTTAGATTGGAGTGGAACAAGGTACTGGGCACCAAATGCCACCTCCACAACCTCAATGTCGGGTGGCTTCACAAACCAAGCTGTTCACAGAACCACGCCAAATTTTAAAACGAACCAGACCCCCAAATCCACACCCAGCAGTGGGTCTTCAGCGAGGTCAAACTCAACCCAACTCACTAGCCTGAGCTCACACTCGTCCGGGTCGTCTCGATCCAGCGGGTCTACAGTTATTAGCTCATTACCATCTTCTAACAGGACTAGGGACTGGGTCAACCAACAGAATTTTAATTTGGAACCACACATGCCTGGATCTCTTAGGACAGCTTTTGTTACTCCACCATCTAGTACGGCCTCTAGTTCAAGCACGGTCTCAACCGTGCCCAAAAATGTTTTGGACTCCTGGAC-ATCTGCGTTTAACACGCGCAGACAGCCGCTATTCGC-ACACCTTCGTAGAAGGGGGGAGTCAAATGTTTAG

>AF414411|II.3/II.3|US|1993|Lionville/247

ATGGCTGGAGCTTTTATAGCAGGATTGGCTGGTGACATGCTCACAAACACTGTGGGGTCTTTGGTTAATGCAGGGGCTAATGCTATTAATCAAAAAGTTGATTTTGAAAATAATAAATATTTGCAAAATGCTTCTTTTAATCATGATAAGGAGATGCTAAGTGCACAAATTGAGGCAACAAAGAGGCTGCAGGCTGACATGATTGCAATCAAACAAGGGGTCTTGGCCGCTGGCGGCTTTTCCCCCACTGATGCAGCACGTGGGGCAATTAATGCCCCAATGGCAAAAGTTTTAGATTGGAGTGGAACAAGGTACTGGGCACCAAACGCCGCCTCCACAACTTCAATGTCAGGTGGCTTCACAAGCCAAGTTGTGCACAGAACCACACCAAATTTCAAAACGAGCCAGGCCCCCAAATTCACACCCAGCAGTGGGTCTTCAGTGAGATCAAGCTCAACCCAACTTACCAACTTGAGCTCACACTCATCCGGGTCGTCCCGATCTAGCGGGTCTACGGTTGTCAGCTCGCTGCCGTCCTCCAGTAGGACTAGGGACTGGGTCAATCAACAAAATCTCAATTTGGAACCACACATGCCTGGATCTCTCAGGACAGCTTTTGTCACCCCACCATCTAGCACAGCCTCTAGTTCAGGCACAGTCTCAACCGTGCCCAAAAATGTTTTGGACTCCTGGAC-ATCTGCGTTTAACACGCGCAGACAGCCGCTGTTTGC-ACACCTTCGTAGAAGGGGGGAGTCAAATGTTTAG

>AF414412|II.3/II.3|US|1994|New Orleans/279

ATGGCTGGAGCTTTTATAGCAGGATTGGCTGGTGACATGCTCACAAACACTGTGGGGTCTTTGGTTAATGCAGGGGCTAATGCTATCAACCAAAAAGTTGATTTTGAAAATAATAAATATTTGCAAAATGCTTCTTTTAATCATGATAAGGAGATGTTAAATGCACAAATTGAGGCAACAAAGAGACTGCAGGCTGATATGATTGCAATCAAACAAGGGGTCTTGACCGCTGGCGGCTTTTCCCCCACTGATGCAGCACGTGGGGCAATTAATGCCCCAATGACAAAAGTTTTAGACTGGAGTGGAACAAGGTACTGGGCACCAAACGCCACCTCCACAACTTCAATGTCAGGTGGCTTCACAAGCCAAGTTGTGCACAGAACCACACCAAATTTTACAACGAGCCAGACCCCCAAATTCACACCCAGCAGTGGGTCTTCAGTGAGATCAAGCTCAACCCAACTCACCAACCTGAGCTCACATTCATCCGGGTCGTCCCGATCTAGCGGGTCTACTGTTGTCAGCTCGCTGCCGTCCTCCAGTAGGACTAGGGACTGGGTCAATCAACAAAATCTCAATTTGGAACCATACATGCCTGGATCTCTCAGGACAGCTTTTGTCACTCCACCATCTAGCACAGCCTCTAGTTCAGGCACAGTCTCAACCGTGCCCAAAAATGTTTTGGACTCCTGGAC-ATCTGCGTTCAACACGCGCAGACAGCCGCTGTTTGC-ACACCTCCGTAGAAGGGGGGAGTCAAATGTTTAG

>AF414413|II.3/II.3|US|1994|Montgomery/312

ATGGCTGGAGCTTTTATAGCAGGATTGGCTGGTGGCATGCTCACAAACACTGTGGGGTCTTTGGTTAATGCAGGGGCCAATGCTATTAATCAAAAAGTTGATTTTGAAAATAATAAATATTTGCAAAATGCTTCTTTTAATCATGATAAGGAGATGTTGAGTGCACAAATTGAGGCAACAAAGAGGCTGCAGGCTGACATGATTGCAATCAAACAAGGGGTCTTGACCGCTGGCGGCTTTTCCCCCACTGATGCAGCACGTGGGGCAATTAATGCCCCAATGACAAAAGTTTTAGATTGGAGTGGAACAAGGTACTGGGCACCAAACGCCGCCTCCACAACTTCAATGTCAGGTGGCTTCACAAGCCAAGTTGTGCACAGAACCACACCAAATTTCAAAACGAGCCAGGCCCCCAAATTCACACCCAGCAGTGGGTCTTCAGTGAGATCAAGCTCAACCCAACTTACCAACTTGAGCTCACACTCATCCGGGTCGTCCCGATCTAGCGGGTCTACGGTTGTCAGCTCGCTGCCGTCCTCCAGTAGGACTAGGGACTGGGTCAACCAACAAAATCTCAATTTGGAACCACACATGCCTGGATCTCTCAGGACAGCTTTTGTCACTCCACCATCTAGCACAGCCTCTAGTTCAGGCACAGTCTCAACCGTGCCCAAAGATGTTTTGGACTCCTGGAC-ATCTGCGTTTAACACGCGCAGACAGCCGCTGTTTGC-ACACCTTCGTAGAAGGGGGGAGTCAAATGTTTAG

>AF414414|II.3/II.3|US|1994|Towson/313

ATGGCTGGAGCTTTTATAGCAGGATTGGCTGGTGACATGCTCACAAGCACTGTGGGGTCTTTGGTTAATGCAGGGGCCAATGCTATTAATCAAAAAGTTGATTTTGAAAATAATAAATATTTGCAAAATGCTTCTTTTAATCATGATAAGGAGATGTTGAGTGCACAAATTGAGGCAACGAAGAGGCTGCAGGCTGACATGATTGCAATCAAGCAAGGGGTCTTGACCGCTGGCGGCTTTTCCCCCACTGATGCAGCACGTGGGGCAATTAATGCCCCAATGACAAAAGTTTTAGATTGGAGTGGAACAAGGTACTGGGCACCAAACGCCGCCTCCACAACTTCAATGTCAGGTGGCTTCACAAGCCAAGTTGTGCACAGAACCACACCAAATTTCAAAACGAGCCAGGCCCCCAAATTCACACCCAGCAGTGGGTCTTCAGTGAGGTCAAGCTCAACCCAACTTACCAACTTGAGCTCACACTCATCCGGGTCGTCCCGATCTAGCGGGTCTACAGTTGTCAGCTCGCTGCCGTCCTCCAGTAGGACTAGGGACTGGGTCAACCAACAAAATCTCAATTTGGAACCACACATGCCTGGATCTCTCAGGACAGCTTTTGTCACTCCACCACCTAGCACAGCCTCTAGTTCAGGCACAGTCTCAACCGTGCCCAAAGATGTTTTGGACTCCTGGAC-ATCTGCGTTTAACACGCGCAGACAGCCGCTGTTTGC-ACACCTTCGTAGAAGGGGGGAGTCAAATGTTTAG

>AF414415|II.3/II.3|US|1995|Brattleboro/321

ATGGCTGGAGCTTTTATAGCAGGATTGGCTGGTGACATGCTCACAAACACTGTGGGGTCTTTGGTTAATGCAGGGGCCAATGCTATTAATCAAAAAGTTGATTTTGAAAATAATAAATATTTGCAAAATGCTTCTTTTAATCATGATAAGGAGATGTTGAGTGCACAAATTGAGGCAACAAAGAGGCTGCAGGCTGACATGATTGCGATCAAACAAGGGGTCTTGACCGCTGGCGGCTTTTCCCCCACTGATGCAGCACGTGGGGCAATTAATGCCCCAATAGCAAAAGTTTTGAATTGGAGTGGAACAAGGTACTGGGCACCAAACGCCGCCTCCACAACTTCAATGTCAGGTGGCTTCACAAGCCAAGTTGTGCATAGAACCACACCAAATTTCAAAACGAGCCAGGCCCCCAAATTCACACCCAGCAGTGGGTCTTCAGTGAGATCAAGCTCAACCCAACTTACCAACTTGAGCTCACACTCATCCGGGTCGTCCCGGTCTAGCGGGCCTACGGTTGTCAGCTCGCTGCCGTCCTCCAGTAGGACTAGGGACTGGGTCAACCAACAAAATCTCAATTTGGAACCACACATGCCTGGATCTCTCAGGACAGCTTTTGTCACTCCACCATCTAGCACAGCCTCTAGTTCAGGCACAGTCTCAACCGTGCCCAAAGATGTTTTGGACTCCTGGAC-ATCTGCGTTTAACACGCGCAGACAGCCGCTGTTTGC-ACACCTTCGTAGAAGGGGGGAGTCAAATGTTTAG

>AY652979|II.21/II.3|US|2003|Paris Island/2003

ATGGCTGGAGCTTTTATAGCAGGATTAGCTGGTGATATACTTACAAATACTGTAGGATCTTTAGTTAATGCAGGGGCTAATGCCATTAATCAAAAAGTTGATTTTGAAAACAATAAATATTTACAAAATGCTTCCTTTAATCATGACAAGGAAATGTTAAATGCACAAATTGAGGCAACAAAGAGGTTACAGGCTGACATGATTGCTATCAAACAAGGGGTTTTGACCGCTGGCGGCTTCTCCCCTACTGATGCAGCCCGCGGGGCAATTAATGCCCCCATGACAAAAGTCCTAGATTGGAATGGGACGAGGTACTGGGCACCAAATGCCACCTCCACAACCTCGATGTCGGGTGGCTTCACAAATCAGGCTGTGCACAGAACCACGCCAAATTTTAAAACGAACCAGGCTCCCAAATCCACACCCAGCAGTGGGTCTTCAATGAGGTCAAACTCAACCCAAATCACTAGCCTGAGCTCACACTCGTCCGGGTCGTCTCGATCCAGCGGGTCTACAGTTGTTAGCTCATTACCATCCTCTAACAGGACTAGGGACTGGGTCAACCAGCAAAATTTTAATTTGGAACCACACATGCCTGGGTCTCTTAGGACAGCCTTTGTTACTCCACCATCTAGTACAGCCTCCAGTTCAGGTACGGTCTCGACCGTGCCCAAAAATGTTTTGGACTCCTGGAC-ATCTGCGTTCAATACGCGCAGACAGCCGCTATTCGC-ACACCTTCGCAGAAGGGGGGAGTCAAATGTTTAG

>DQ379713|I.1/II.3|AU|1983|Goulburn Valley G5175 A

ATGGCTGGGGCTTTTATAGCAGGATTGGCTGGTGACATGCTCACAAATACTGTAGGGTCTTTGGTTAATGCAGGGGCTAGTGCCATCAATCAAAAAGTTGATTTTGAGAATAATAAATATTTACAAAATGCATCTTTTGTTCATGATAAGGAGATGTTAAATGCACAAATTGAGGCAACAAAGAGGCTACAGACTGATATGATTGCTATCAAACAAGGGGTTTTGACCGCTGGCGGCTTCTCCCCCACTGATGCAGCCCGTGGGGCAATCAGTGCCCCCATGACAAAAGTTTTGGATTGGAGTGGAACGAGGTACTGGGCACCAAACGCCACCTCCACAACCTCAATGTCAGGTGGCTTCACAAGCCAAACTGTGCACAGAACCACACCAAATTTTAAGACGAACCAGGCCCCCAAGCCCGCACCCAGCAGTGGGTCTTCAGTGAGGTCAAACTCAACCCAACCCACTAGCTTGAGTTCACACTCCTCCGGATCGTCTCGATCCAGCGGGTCTACGGCTGTCAGCTCATTGTCATCTTCCAACAGGACTAGGGATTGGGTCAATCAACAGAATTTTGATTTGGAACCATACATGCCTGGATCTCTTAGGACAGCTTTTGTCACTCCACCATCTAGTACAGCCTCTAGTTCAGGCACGGTCTCAACCGTGCCCAAAAGTGTTTTGGACTCCTGGAC-ATCTGCGTTTAACACGCGCAGACAACCGCTATTTGC-ACACCTTCGTAGAAGGGGGGAGTCAAATGTTTAG

>EF670649|II.12/II.3|CN|2006|Hebei/48580

ATGGCTGGAGCTTTTATAGCAGGACTAGCTGGTGATATATTCACAAACACTGTAGGATCTCTAGTTAATGCAGGGGCTAATGCCATTAATCAAAAAGTTGATTTTGAAAACAATAAATATTTGCAAAACGCTTCCTTTAATCATGATAAGGAAATGTTAAATGCACAAATTGAGGCAACAAAGAGGTTACAGGCTGACATGATTGCTATCAAACAAGGGGTTTTGACCGCTGGCGGCTTTTCCCCTACTGATGCAGCCCGCGGGGCAATTAATGCCCCCATGACAAAAGTCCTAGATTGGAATGGAACGAGGTACTGGGCACCAAATGCCACCTCTACAACCTCGATGTCGGGTGGCTTCACAAACCAGGCTGTGCACAGAACCACGCCAAATTTTAAAACGAACCAGGCCCCCAAATCCACACCCAGCAGTGGGTCTTCAGTGAGGTCACACTCAACCCAAATCACTAGTCTGAGCTCACACTCGTCCGGGTCGTCTCGATCCAGCGGGTCTACAGTTGTTAGCTCATTACCATCCTCTAACAGGACTAGGGACTGGGTCAACCAACAAAATTTTAATTTGGAACCACACATGCCTGGGTCCCTTAGGACAGCTTTTGTTACTCCACCATCTAGTACAGCCTCTAGTTCAGGCACCGTCTCAACCGTGCCCAAAAATGTTTTGGACTCCTGGAC-ATCTGCGTTTAATACGCGCAGACAGCCGCTATTCGC-ACACCTTCGTAGAAGGGGGGAGTCAAATGTTTAG

>EU072241|II.21/II.3|CN|2004|CHN39186/CC04

ATGGCTGGAGCTTTTATAGCAGGATTAGCTGGTGATATATTCACAAATACTGTAGGATCTCTAGTTAATGCAGGGGCTAATGCCATTAATCAAAAAGTTGATTTTGAAAACAATAAATATTTGCAAAATGCTTCTTTTAATCATGATAAGGAAATGTTAAATGCACAAATTGAGGCAACAAAGAGGTTACAGGCTGACATGATTGCTATCAAACAAGGGGTTTTGACCGCTAGCGGCTTCTCCCCTACTGATGCAGCCCGCGGGGCAATCAATGCCCCCATGACAAAAGTCCTAGATTGGAATGGAACGAGGTACTGGGCACCAAATGCCACCTCCACAACCTCGATGTCGGGTGGCTTCACAAATCAGGCTGTGCACAGAACCACGCCAAATTTTAAAACGAACCAGGCTCCCAAACCCGCACCCAGCAGTGGGTCTTCAGTGAAGTCAAACTCAACCCAAATCACTAGCCTGAGCTCACACTCGTCCGGGTCGTCTCGATCCAGCGGGTCTACAGTTGTTAGCTCATTACCATCCTCTAACAGGACTAGGGACTGGGTCAACCAACAAAATTTTAATTTGGAACCACACATGCCTGGATCTCTCAGGACTGCTTTTGTTACTCCACCATCTAGTACAGCCTCCAGTTCAGGTACGGTCTCAACCGTGCCCAAAAATGTTTTGGACTCCTGGAC-ATCTGCGTTTAATACACGCAGACAGCCGCTATTCGC-ACACCTTCGCAGAAGGGGGGAGTCAAATGTTTAG

>EU072243|II.21/II.3|CN|2004|CHN39246/CC04

ATGGCTGGGGCTTTTATAGCAGGATTAGCTGGTGACATATTCACAAATACTGTAGGATCTCTAGTTAATGCAGGGGCTAATGCCATTAATCAAAAAGTTGATTTTGAAAATAATAAATATTTGCAAAATGTTTCCTTCAATCATGATAAGGAGATGTTAAATGCACAAATTGAGGCAACAAAGAGGTTACAGGCTGACATGATTGCTATCAAACAAGGGGTTTTGACCGCTGGCGGCTTCTCCCCTACTGATGCAGCCCGCGGGGCAATCAATGCCCCCGTGACTAAAGTCCTAGATTGGAACGGAACAAGGTACTGGGCACCAAATGCCACCTCCACAACCTCGATGTCGGGTGGCTTCACAAATCAGGCTGTGCACAGAACCACGCCAAATTTTAAAACGAACCAGGCTCCCAAATCCACACCCAGCAGTGGGTCTTCAGTGAAGTCAAACTCAACCCAAACCACTAGCCTGAGCTCACACTCGTCCGGGTCGTCTCGATCCAGCGGGTCTACAGTTGTTAGCTCATTATCATCCTCTAACAGGACTAGGGACTGGGTCAACCAACAAAATTTTAATTTGGAACCACACATGCCTGGATCCCTCAGGACAGCTTTTGTTACTCCACCATCTAGTACAGCCTCCAGTACAGGTACGGTCTCAACCGTGCCCAAAAATGTTTTGGACTCCTGGAC-ATCTGCGCTCAACACACGCAGACAGCCGCTATTCGC-ACACCTTCGCAGAAGGGGGGAGTCAAATGTTTAG

>EU921389|II.21/II.3|IN|2007|PC52

ATGGCTGGAGCTTTTATAGCAGGATTGGCTGGTGACATGCTCACAAATACTGTAGGATCTTTAGTTAATGCAGGGGCTAATGCCATTAATCAAACAATTGATTTTGAAAATAATAAATATTTGCAAAATGCCTCTTTTAATCATGATAAGGAGATGTTGAACGCACAAATTGAGGCAACAAAGAGATTACAGGCTGACATGATTGCTATCAAACAAGGGGTTTTGACCGCTGGCGGCTTCTCCCCTACTGATGCAGCCCGCGGGGCAATCAATGCCCCCATGACAAAAGTCCTAGATTGGAATGGAACGAGATACTGGGCACCAGGTGCCACCTCCACAACCTCGATGTCGGGTGGCTTCACAAATCAAACTGTGCACAGATCCACACCAAATTTTAAAACGAACCAGGCCCCCAAACCCACACCCAGCAGTGGGTCTTCAGTGAGGTCAAATTCAACCCAAATCACTAGCCTGAGTTCACACTCGTCCGGGTCGTCTCGATCCAGCGGGTCTACAGTTGTCAACTCAATACCATCCTCTAACAGGACTAGGGACTGGGTCAACCAACAAAATTTTAATTTGGAACCACACATGCCTGGATCTCTTAGGACAGCTTTTGTCACTCCACCATCTAGTACAGCCTCTAGCTCAGGCACAGTCTCAACTGTGCCCAAAAATGTTTTGGACTCCTGGAC-ATCTGCGTTTAACACGCGCAGACAACCGCTATTCGC-ACACCTTCGCAGAAGGGGGGAGTCGAATGTTTAG

>GQ856466|II.12/II.3|CN|2007|Beijing/55037

ATGGCTGGAGCTTTTATAGCAGGATTAGCTGGTGATGTATTCACAAACACTGTAGGATCTCTAGTTAATGCAGGGGCTAATGCCATTAATCAAAAAGTTGATTTTGAAAATAATAAATATTTGCAAAATGCTTCCTTTAATCATGATAAGGAAATGCTGAATGCACAAGTTGAGGCAACAAAGAGGTTACAGGCTGACATGATTGCTATCAAACAAGGGGTTTTGACCGCTGGCGGCTTTTCCCCTACTGATGCCGCCCGCGGGGCAATTAATGCCCCCATGACAAAAGTCCTAGATTGGAATGGAACGAGGTACTGGGCACCAAATGCCACCTCTACAACCTCGATGTCGGGTGGCTTCACAAACCAGGCTGTGCACAGAACCACGCCAAATTTTAAAACGAACCAGGCCCCCAAATCCACACCCAGCAGTGGGTCTTCATTGAGGTCACACTCAACCCAAATCACTAGTCTGAGCTCACACTCGTCCGGGTCGTCTCGATCCAGCGGGTCTACAGTTGTTAGCTCATTACCATCCTCTAACAGGACTAGGGACTGGGTCAACCAACAAAATTTTAATTTGGAACCACACATGCCTGGGTCCCTTAGGACAGCTTTTGTTACTCCACCATCTAGTACAGCCTCTAGTTCAGGCACTGTCTCAACCGTGCCCAAAAATGTTTTGGACTCCTGGAC-ATCTGCGTTTAATACGCGCAGACAGCCGCTATTCGC-ACACCTTCGTAGAAGGGGGGAGTCAAATGTTTAG

>GQ856467|II.12/II.3|CN|2007|Beijing/55040

ATGGCTGGAGCTTTTATAGCAGGGTTAGCTGGTGATATATTCACAAACACTGTAGGATCTCTGGTTAATGCAGGGGCTAATGCCATTAATCAAAAAGTTGATTTTGAAAATAATAAATATTTGCAAAATGCTTCCTTTAATCATGATAAGGAGATGTTAAATGCACAAATTGAGGCAACAAAAAGGTTACAGGCTGACATGATTGCTATCAAACAAGGGGTTTTGACCGCTGGCGGCTTTTCCCCTACTGATGCAGCCCGCGGGGCAATTAACGCCCCCATGACAAAAGTCCTAGATTGGAATGGAACGAGGTACTGGGCACCAAATGCCACCTCTACGACCTCGATGTCGGGTGGCTTCACAAACCAGGCTGTGCACAGAACCACGCCAAATCTTAAAACGAACCAGGCCCCCAAATCCACACCCAGCAGTGGGTCCTCAGTGAGGTCACACTCAACCCAAACCACTAGTCTGAGCTCACACTCGTCCGGGTCGTCTCGATCCAGCGGGTCTACAGTTGTTAGCTCATTACCATCCTCCAACAGGACTAGGGACTGGGTCAACCAGCAAAATTTTAATTTGGAACCACACATGCCTGGGTCCCTTAGGACAGCTTTTGTTGCTCCACCATCTAGTACAGCCTCTAGTTCAGGCACTGTCTCAACCGTGCCCAAAAATGTTTTGGACTCCTGGAT-ATCTGCGTTTAATACGCGCAGACAGCCGCTATTCGC-ACACCTTCGTAGAAGGGGGGAGTCAAATGTTTAG

>GU980585|II.12/II.3|KR|2006|CBNU1

ATGGCTGGAGCTTTTATAGCAGGATTAGCTGGTGATATGTTCACAAACACTGTAGGATCTCTAGTTAATGCAGGGGCTAATGCCATTAATCAAAAAGTTGATTTTGAAAACAATAAATATTTGCAAAATGCTTCCTTTAATCATGATAAGGAAATGTTAAATGCACAAATTGAGGCAACAAAGAGGTTACAGGCTGACATGATTGCTATCAAACAAGGGGTTTTGACCGCTGGCGGCTTTTCTCCTACTGATGCAGCCCGCGGGGCAATTAATGCCCCCATGACAAAAGTCCTAGATTGGAATGGAACGAGGTACTGGGCACCAAATGCCACCTCTACAACCTCGATGTCGGGTGGCTTCACAAACCAGGCTGTGCACAGAACCACGCCAAATTTTAAAACGAACCAGGCCCCCAAATCCACACCCAGCAGTGGGTCTTCAGTGAGGTCACACTCAACCCAAATCACTAATCTGAGCTCACACTCGTCCGGGTCGTCTCGATCCAGCGGGTCTACAGTTGTTAGCTCATTACCATCCTCTAACAGGACTAGGGACTGGGTCAACCAACAAAATTTTAATTTGGAACCACACATGCCTGGATCCCTTAGGACAGCTTTTGTTACTCCACCATCTAGTACAGCCTCTAGTTCAGGCACTGTCTCAACCGTGCCCAAAAATGTTTTGGACTCCTGGAC-ATCTGCGTTCAATACGCGCAGACAGCCGCTATTCGC-ACACCTTCGTAGAAGGGGGGAGTCAAATGTTTAG

>GU991355|II.12/II.3|CN|2009|SH312

ATGGCTGGAGCTTTTATAGCAGGATTAGCTGGTGATATATTCACAAATACTGTAGGATCTCTAGTTAATGCAGGGGCTAATGCCATTAATCAAAAAGTTGATTTTGAAAATAACAAATATTTGCAAAATGCTTCCTTTAATCATGATAAAGAAATGTTAAATGCACAAATTGAGGCAACAAAGAGGTTACAGGCTGACATGATTGCTATCAAACAGGGGGTTTTGACCGCTGGCGGCTTTTCCCCCACTGATGCAGCTCGCGGGGCAATTAGTGCCCCCATGACAAAAGTCCTAGATTGGAATGGAACGAGGTACTGGGCACCAAATGCCACCTCTACAACCTCGATGTCGGGTGGCTTCACAAACCAGGCTGTGCACAGAACCGCGCCAAATTTTAAAACGAACCAGGCCCCCAAATCCACACCCAGCAGTGGGTCTTCAGTGAGGTCACTCTCAACCCAAATCACTAGTCTGAGCTCACACTCGTCCGGGTCGTCTCGATCCAGCGGGTCTACAGTTGTTAGCTCATTACCATCCTCTAACAGGACTAGGGACTGGGTCAATCAACAAAATTTCAATTTGGAACCACACATGCCTGGGTCCCTTAGGACAGCTTTTGTTACTCCACCATCTAGTACAGTCTCTAGTTCAGGCACTGTCTCAACCGTGCCCAAGAA-GTTTTGGACTCCTGGACAATCTGCGTTTAATACGCGCAGACAGCCGCTATTCGC-ACACCTTCGTAGAAGGGGGGAGTCAAATGTTTAG

>HCU22498|II.3/II.3|US|<1995|MX

ATGGCCGGAGCTTTTATAGCAGGATTGGCTGGGGACATGCTCACAAACACTGTGGGGTCTTTGGTTAATGCAGGGGCTAATGCTATCAATCAAAAAGTTGATTTTGAAAATAATAAATATTTGCGAAATGCTTCTTTTAATCATGATAAGGAGATGCTAAATGCACAAATTGCGGCAACAAAGAGGCTGCAGGCTGACATGATTGCAATCAAACAACCCGTCTTGAC-GCTGGCGGCTTTTCCCCTACTGATGCAGCACGTG--GCAATTAATGCCCCAATGACAAAAGTTTTAGATTGGAGTGGAACAAGGTACTGGGCACCAAACGCCACCTCCACAACTTCAATGTCAGGTGGCTTCACAAGCCAAGTTGTGCACAGAACCACACCAAATTTCAAAACGAACCAGGCCCCCGAATTCACACCCAGCAGTGGGTCTTCAGTGAGATCAAGCTCAACCCAACTCACCAACTTGAGCTCACACTCATCTGGTCGGTCCCGATCTAGCGGGTCTACGGTTGTCAGCTCGCTGCCGTCCTCCAGTAGGACTAGGGATCGGGTCAATCAACAGAATCTCAATTTGGAACCATACATGCCTGGATCTCTCAGGATAGCTTTTGTCACTCCACCATCTAGCACAGCCTCTAGTTCAGGCACAGTCTCAACCGTGCCCAAAAATGTTTTGGACTCCTGGAC-ATCT-CGTTTAACAGGCGCAGACAGCCGCTGTTTGATACCCCTTCGTAGAAGGGGGGAGTCAAATGTTTAG

>JN565063||/II.3|US|2010|Milwaukee009

ATGGCTGGAGCCTTTATAGCAGGATTGGCTGGTGACATGCTCACAAATACTGTAGGATCTTTAGTTAATGCAGGGGCTGGTGCTATTAATCAAAAAGTTGATTTTGAAAATAATAAATATCTACAAAATGCATCCTTTAATCATGATAAGGAGATGTTAAATGCACAAATTGAGGCAACAAAGAGGCTACAGGCTGACATGATTGCTATCAAACAAGGGGTTTTGGCCGCTGGCGGTTTCTCCCCCACTGATGCAGCCCGTGGGGCTATCAACGCCCCCATAACAAAAGTTCTGGACTGGAATGGAACCAGGTACTGGGCACCAAATGCCACCTCCACAACCTCGATGTCAGGTGGCTTCACAGGTCAAGCTGTGCACAGAGCTATACCAAATTTTAAAACCAATCAAGCCCCAAAATCCACACCCAGCAGTGGGTCTTCAGTGAGGTCAAGCTCAACCCAACTCACCAACTTGAGCTCACACTCATCTGGGTCTTCTCGATCCAGCGGATCCACGATGGTCAGCTCGTTACCATCTTCCAGTAGGACTAGAGACTGGGTTAACCAACAGAATTTTAATTTGGAACCATACATGCCTGGATCTCTCAGGACAGCTTTTGTTACTCCACCATCTAGTACAGCCTCTAGCTTAGACACGGTCTCAACCGTGCCCAAGAGTGTTTTGGACTCCTGGAC-ATCTGCGTTTAACACGCGCAGACAGCCGCTTTTCGC-ACACCTTCGTAGAAGGGGGGAGTCAAATGTTTAG

>JN899244||/II.3|US|2004|Glastonbury1164

ATGGCTGGAGCTTTTATAGCAGGATTAGCTGGTGACATACTCACAAATACTGTAGGATCTTTGGTTAATGCAGGGGCTAATGCCATTAATCAAAAAGTTGATTTTGAAAACAATAAATATTTACAAAATGCTTCCTTTAATCATGATAAGGAAATGTTAAATGCACAAATTGAGGCAACAAAGAGGTTACAGGCTGACATGATTGCTATCAAACAAGGGGTTTTGACCGCTGGCGGCTTCTCCCCTACTGATGCAGCCCGCGGGGCAATTAATGCCCCCATGACAAAAGTCCTAGATTGGAATGGGACGAGGTACTGGGCACCAAATGCCACCTCCACAACCTCGATGTCGGGTGGCTTCACAAATCAGGCAGTGCACAGAACCACGCCAAATTTTAAAACGAACCAGGCTCCCAAATCCACACCCAGCAGTGGGTCTTCAGTGAGGTCAAACTCAACCCAAATCACTAGCCTGAGCTCACACTCTTCCGGGTCGTCTCGATCCAGCGGGTCTACAGTAGTTAGCTCATTACCATCCTCTAACAGGACTAGGGACTGGGTCAACCAACAAAATTTCAATTTGGAACCACACATGCCTGGGTCTCTTAGGACAGCTTTTGTTACTCCACCATCTAGTACAGCCTCTAGTTCAGGTACGGTCTCGACCGTGCCCAAAAATGTTTTGGACTCCTGGAC-ATCTGCGTTCAATACGCGCAGACAGCCGCTATTCGC-ACACCTTCGCAGAAGGGGGGAGTCAAATGTTTAG

>JX846924|II.g/II.3|CN|1978|HK71

ATGGCTGGAGCCTTTATAGCAGGATTGGCTGGTGACATGCTCACAAGTACTGTGGGATCTTTAGTTAATGCAGGGGCTAGTGCTATCAATCAAAAAGTTGATTTTGAAAATAATAAATATTTACAAAATGCATCTTTTAATCATGATAAGGAGATGTTAAATGCACAAATTGAGGCAACAAAGAGGCTACAGGCTGACATGATTGCTATCAAACAAGGGGTCTTGACCGCTGGCGGCTTTTCCCCCACTGATGCAGCCCGTGGGGCAATTAATGCCCCCATAACAAGAGTTTTGGACTGGAGTGGAACGAGGTACTGGGCACCAAACGCCACCTCCACAACCTCAATGTCAGGTGGCTTCACAAGCCAAACTGTACACAGAACCACACCAAATTTTAAAACGAACCAGGCCCCCAAGTCCACACCCAGCAGTGGGTCTTCAGTGAGATCAAACTCAACCCAACTCACTAGCTTGAGCTCACACTCATCCGGGTCGTCTCGATCCAGCGGGTCTACGGTTGTTAGCTCATTGCCATCTTCCAACAGGACTAGGGATTGGGTCAATCAACAGAATTTCAATTTGGAACCACACATGCCTGGATCTCTCAGGACAGCTTTTGTCACTCCACCATCTAGTACAGCCTCTAATTCAGACACGGTCTCAACCGTGCCCAAAAGTGTTTTGGACTCCTGGAC-ATCTGCGTTTAATACGCGCAGACAGCCGCTATTCGC-ACACCTTCGCAGAAGGGGGGAGTCAAATGTTTAG

>KF006265||/II.3|US|2002|TCH-104

ATGGCTGGAGCTTTTATAGCAGGATTAGCTGGTGATATACTCACAAATACTGTAGGATCTCTAGTTAATGCAGGGGCTAATGCCATTAATCAAAAAGTTGATTTTGAAAATAATAAATATTTACAAAATGCTTCTTTCAATCATGATAAGGAAATGTTAAATGCACAAATTGAGGCAACAAAGAGGCTACAGGCTGACATGATTGCTATCAAACAAGGGGTTTTGACCGCTGGCGGCTTTTCCCCTACTGATGCAGCCCGCGGGGCAATTAATGCCCCCATGACAAAAGTCCTAGATTGGAATGGAACGAGGTACTGGGCACCAAATGCCACCTCCACAACCTCGATGTCGGGTGGCTTCACAAATCAGGTTGTGCACAGAACCACGCCAAATTTTAAAACGAACCAGGCTCCCAAATCCACACCCAGCAGTGGGTCTTCAGTGAAGTCAAACTCAACCCAAATCACTAGCCTGAGCTCACACTCGTCCGGGTCGTCTCGATCCAGCGGGTCTACAGTTGTTAGCTCATTACCATCCTCTAACAGGACTAGGGACTGGGTCAACCAACAAAATTTTAATTTGGAACCACACATGCCTGGATCTCTTANGACAGCTTTTGTTACTCCACCATCTAGTACAGCCTCTAGTTCACGCACGGTCTCAACCGTGCCCAAAAATGTTTTGGACTCCTGGNC-ATCTGCGTTCAATACGCGCAGACAGCCGCTATTCGC-ACACCTTCGCAGAA-GGGGGAGTCAAATGTTTAG

>KF306213|II.12/II.3|CN|2013|Jingzhou/2013402

ATGGCTGGAGCTTTTATAGCAGGATTGGCTGGTGATATGCTCACAAATACTGTAGGATCTTTAGTTAATGCAGGGGCTAATGCCATTAATCAAACAATTGATTTTGAAAATAATAAATATTTGCAAAATGCCTCTTTTAATCATGATAAGGAGATGTTGAGTGCACAAATTGAGGCAACAAAGAGGTTACAGGCTGACATGATTGCTATCAAGCAAGGGGTTTTGACCGCTGGCGGCTTCTCCCCTACTGATGCAGCCCGCGGGGCAATTAATGCCCCCATGACAAAAGTCCTAGATTGGAATGGAACGAGATACTGGGCACCAGGTGCCACCTCCACAACCTCGATGTCGGGTGGCTTCACGAATCAAACTGCGCACAGACCCACACCAAATTTTAAAACGAACCAGGCCCCCAAACCCACCCCCAACAGTGGGTCCTCAGTGAGGTCAAATTCAACCCAAATTACCAGCCTGAGTTCACACTCGTCCGGGTCGTCTCGATCCAGCGGGTCTACAGTTGTCAGCTCAATACCATCCTCTAACAGGACTAGGGACTGGGTTAACCAACAAAATTTTAATTTGGAACCACACATGCCTGGATCTCTTAGAACAGCTTTTGTCACTCCACCATCTAGTACAGCCTCTAGCTCAGGCACAGTCTCAACTGTGCCCAAAAATGTTTTGGACTCCTGGAC-ATCTGCGTTTAATACGCGCAGACAGCCGCTATTCGC-ACACCTTCGCAGAAGGGGGGAGTCAAATGTTTAG

>KJ145323|II.21/II.3|TW|2013|13-BG-1

ATGGCTGGAGCTTTTATAGCAGGATTGGCTGGTGACATGCTCACAAATACTGTAGGATCTTTAGTTAATGCAGGGGCTAATGCTATTAATCAAACAATTGATTTTGAAAATAATAAATATTTGCAAAATGCCTCTTTTAATCATGATAAGGAGATGTTGAATGCACAAGTTGAGGCAACAAAGAGGTTACAGGCTGACATGATTGCTATCAAACAAGGAGTTTTGACCGCTGGCGGCTTCTCCCCTACTGATGCAGCCCGCGGGGCAATTAATGCTCCCATGACAAAAGTCCTAGATTGGAATGGAACGAGATACTGGGCGCCAGGTGCCACCTCCACAACCTCGATGTCAGGTGGCTTCACAAATCAAACTGTGCGCAGATCCACACCAAATTTTAAAACGAACCAGGCCCCCAAACCCGCACCCAGCAGTGGGTTTTCAGTGAGGTCAAATTCAACCCAAATCACTAGCCTGAGCTCACACTCGTCCGGGTCGTCTCGATCCAGCGGGTCTACAGTTGTCAGCTCAATACCGTCCTTTAACAGGACTAGGGACTGGGTTAACCAACAAAATTTTAATTTGGAACCACACATGCCTGGATTTTTTAGGACAGCCTTTGTCACTCCACCATTTAGTACAGCCTTTAGCTCAGGCACAGTTTCAACTGTGCCCAAAAATGTTTTGGACTCCTGGAC-ATTTGCGTTTAACACGCGCAGACAGCCGCTATTCGC-ACACCTTCGCAGAAGGGGGGAGTCAAATGTTTAG

>KJ194500|II.3/II.3|NL|1995|Amsterdam/1

ATGGCTGGAGCTTTTATAGCAGGATTGGCTGGTGACATGCTCACAAACACTGTGGGGTCTTTGGTTAATGCAGGGGCCAATGCTATTAATCAAAAAGTTGATTTTGAAAATAATAAATATTTGCAAAATGCTTCTTTTAATCATGACAAGGAGATGTTAAGTGCACAAATTGAGGCAACAAAGAGGCTGCAGGCTGACATGATTGCAATCAAACAAGGGGTCTTGACCGCTGGCGGCTTTTCCCCCACTGATGCAGCACGTGGGGCAATTAATGCCCCAATGACAAAAGTTTTAGATTGGAGTGGAACAAGGTACTGGGCACCAAACGCCGCCTCCACAACTTCAATGTCGGGTGGCTTCACAAGCCAAGTTGTGCACAGAACCACACCAAATTCCAAAACGAGCCAGGCCCCCAAATTCACACCCAGCAGTGGGTCTTCAGTGAGATCAAGCTCAACCCAACTTACCAACTTGAGCTCACACTCATCCGGGTCGTCCCGATCTAGCGGGTCTACGGTTGTCAGCTCGCTGCCGTCCTCCAGTAGGACTAGGGACTGGGTCAACCAACAAAATCTCAATTTGGAACCATACATGCCTGGATCTCTCAGGACAGCTTTTGTCACTCCACCATCTAGTACAGCCTCTAGTTCAGGCACAGTCTCAACCGTGCCCAAAAATGTTTTGGACTCCTGGAC-ATCTGCGTTTAACACGCGCAGACAGCCGCTGTTTGC-ACACCTTCGTAGAAGGGGGGAGTCAAATGTTTAG

>KJ194504|II.3/II.3|NL|1994|Amsterdam

ATGGCTGGAGCTTTTGTAGCAGGATTGGCTGGTGACATGCTCACAAACACTGTAGGGTCTTTGGTTAATGCAGGGGCCAATGCTATTAATCAAAAAGTTGATTTTGAAAATAATAAATATTTGCAAAATGCTTCTTTTAATCATGATAAGGAGATGTTAAGTGCACAAATTGAGGCAACAAAGAGGCTGCAGGCTGACATGATTGCAATCAAACAAGGGGTCTTGACCGCTGGCGGCTTTTCCCCCACTGATGCAGCACGTGGGGCAATTAATGCCCCAGTGACAAAAGTTTTAGATTGGAGTGGAACAAGGTACTGGGCACCAAACGCCGCCTCCACAACTTCAATGTCAGGTGGCTTCACAAGCCAAGTTGTGCACAGAACCACACCAAATTTCAAAACGAGCCAGGCCCCCAAATTCACACCCAGCAGTGGGTCTTCAGTGAGATCAAGCTCAACCCAACTCACCAACTTGAGCTCACACTCATCCGGGTCGTCCCGATCTAGCGGGTCTACGATTGTCAGCTCGCTGCCGTCCTCCAGTAGGACTAGGGACTGGGTCAACCAACAAAATCTCAATTTGGAACCACACATGCCTGGATCTCTCAGGACAGCTTTTGTCACTCCACCATCTAGCACAGCCTCTAGTTCAGGCACAGTCTCAACCGTGCCCAAAAATGTTTTGGACTCCTGGAC-ATCTGCGTTTAACACGCGCAGACAGCCGCTGTTTGC-ACACCTTCGTAGAAGGGGGGAGTCAAATGTTTAG

>LN854569|II.21/II.3|NL|2014|Groningen

ATGGCTGGGGCTTTTATAGCAGGATTGGCTGGTGACATGCTCACAAATACTGTAGGATCTTTAGTTAATGCAGGAGCTAATGCTATTAATCAAACAATTGATTTAGAAAATAATAAATATTTGCAAAATGCTTCTTTTAATCATGATAAGGAGATGTTGAATGCACAAATTGAGGCAACAAAGAAGTTACAGGCTGACATGATTGCTATCAAGCAAGGGGTCTTGACCGCTGGCGGCTTCTCCCCTACTGATGCAGCCCGTGGGGCAATTAATGCCCCCATGACAAAAGTCCTAGATTGGAATGGAACGAGACACTGGGCACCAGGTGCCACCTCCACAACCTCGATGTCGGGTGGCTTTACACATCAAACTGTGCACAGATCCACACCAAATTTTAAAACGAACCAGGCTCCCAAATCCACACCCAGCAGTGGGTCTTCAGTGAGGTCAAACTCAACCCAAATCACTAGCCTGAGTTCACACTCGTCCGGGTCGTCTCGATCCAGCGGGTCTACAGTTATCAGATCAATACCATCCTCTGACAGGACTAGGGACTGGGTCAACCAACAAAATTTTAATTTGGAACCACATATGCCTGGATCTCTTAGGACAGCTTTTGTCACTCCACCATCTAGTACAGCCTCTAGCTCAGGCACAGTCTCAACTGTGCCCAAAAATGTTTTGGACTCCTGGAC-ATCTGCGTTCAACACGCGCAGACAGCCGCTATTCGC-ACACCTTCGCAGAAGGGGGGAGTCAAATGTTTAG
